# The effects of coherent breathing and blue light after an acute stress on sleepiness and the stress response in a workplace setting: A randomized double-blind cross-over trial

**DOI:** 10.1371/journal.pone.0357293

**Published:** 2026-09-30

**Authors:** Lucie Ráčková, Madison Diamond, Markéta Makarová, Pavel Morcinek, Inès Butin, Federico Nemmi, Stanislas Boyer, Laure Boyer, Julie Bienertová Vašků, Emma Chabani

**Affiliations:** 1 First Department of Neurology, St. Anne’s University Hospital and Faculty of Medicine, Masaryk University, Brno, Czechia; 2 RECETOX, Faculty of Science, Masaryk University, Brno, Czech Republic; 3 Human design Group, Toulouse, France; 4 Incubator of Kinanthropology Research, Faculty of Sports, Masaryk University, Brno, Czech Republic; 5 Institut de médecine et de physiologie spatiales (MEDES), Centre national d’études spatiales, Toulouse, France; Suez Canal University Faculty of Medicine, EGYPT

## Abstract

This study evaluates the effectiveness of an innovative tertiary stress management intervention for workplace application, combining 0.1 Hz coherent breathing with blue light exposure via the ÉOS device. We hypothesize that the intervention can mitigate acute stress reactions and sleepiness more effectively than passive rest without external stimulation. Using a randomized-controlled crossover double-blind design in a workplace-based laboratory setting, we assessed the effect of an intervention following exposition to Maastricht Acute Stress Test in 29 individuals. Subjective stress (single item scale), sleepiness (Karolinska Sleepiness Scale), positive and negative affect (Positive and Negative Affect Schedule), along with heart rate variability (HRV) and electroencephalography metrics were evaluated using linear mixed-effect models. The intervention significantly reduced subjective stress by 31.23% (CI 21.41% and 41.83%), decreased sleepiness by 0.84 points (SE 0.17), and increased positive affect by 1.85 points (SE 0.54). No significant effect was found on negative affect, HRV, frontal alpha asymmetry or theta/beta ratio. The findings suggest that combining coherent breathing with blue light exposure effectively reduces acute stress and enhances mood without causing sleepiness, making it a promising workplace stress intervention. This study contributes to stress management research by demonstrating the potential of these tertiary stress management interventions to improve well-being and performance in occupational settings. Future research should evaluate the individual effects of each component, examine long-term outcomes, and include diverse populations to strengthen these findings.

## Introduction

The dynamics of stress and work significantly influence individuals’ lives. Work, either in a standard 9-to-5 job, self-employment, or shift work, is a major stress source. Chronic stress with longer-term impact [1] is well known for being related to health issues, productivity losses, absences, early retirement, and substantial economic costs ranging from millions to billions of dollars [2–7]. Acute stress, lasting minutes or hours, increases arousal and influences cognitive functions [8] which is critical for optimal performance. However, inadequate coping and overload can compromise employees’ well-being, performance, and safety [9,10]. This relationship gains added significance when considering high-risk occupations such as first responders, military, firefighters, police, and astronauts [11–14]. Given these considerations, exploring effective stress management solutions applicable at workplace becomes imperative for employers who want to provide resources for active stress management, optimize workers’ performance, achieve a competitive advantage [5] and for those who care about imposing a positive impact on health and society.

According to Lamontagne et al. [15], stress interventions are categorized as primary (preventive, targeting the work environment and worker behaviors), secondary (enhancing self-efficacy through skills training, education, and coaching), or tertiary (reactive, mitigating stress impacts). Most individual interventions fall into the primary or secondary categories, with a gap in tertiary group interventions [5,15–18]. These interventions typically address chronic rather than acute stress [9] and often involve relaxation, mindfulness, or cognitive-behavioral approaches. Cognitive-behavioral interventions are generally more effective than relaxation or mindfulness, both often part of preventive programs [2,6,17,19]. However, there is limited information on the use of relaxation techniques as a tertiary strategy, their impact on the stress response or outcomes relevant for workplace performance, as interventions may inadvertently increase sleepiness or reduce post-relaxation performance readiness.

Interventions using external stimuli, such as blue light, show promise in balancing stress responses and sleepiness [20]. Blue light’s influence on mood and cognitive functions is well-documented, primarily through its modulation of intrinsically photosensitive retinal ganglion cells (ipRGCs), which connect to brain regions regulating circadian rhythms and sleep [21–24]. Although blue light has been shown to increase alertness, its acute effects on post-exposure arousal and behavioral alertness are mixed [23,25,26]. The ipRGCs also influence mood and cognitive functions by connecting to brain areas involved in emotion processing [24,27–30]. Blue light’s effect on mood may involve physiological reactions affecting neurotransmitter and hormone production [31]. Studies have observed accelerated relaxation and decreased heart rate variability ratios with blue light exposure, suggesting its potential for stress management across diverse populations. Minguillon et al. [20] observed accelerated relaxation using blue light in a chromotherapy room, impacting subjective stress and gamma brainwaves. Similarly, Yuda et al. [26,32] found a reduction in the high frequency (HF) HRV band and the low to high frequency (LF/HF) HRV ratio in response to blue light. However, Choi et al. [33] did not find significant effects on most HRV variables in their study. Taken together, it could be assumed that blue light activates brain areas involved in emotion processing and, when combined with relaxation techniques, may expedite acute stress recovery.

A candidate technique that can be combined with blue light is coherent breathing, achieved through slow and controlled breathing at a 0.1 Hz frequency (six respiration cycles a minute with a one-to-one ratio). A review by Sevoz-Couche & Laborde [34] describes that coherent breathing results in in-phase cardiac oscillations and out-of-phase blood pressure oscillations. These physiological modifications lead to an increase in HRV through vagal efferent enhancement with various positive effects on cognition and stress (Sevoz-Couche & Laborde, 2022). A study on air traffic controllers suggests that biofeedback coherent breathing may be effective in preventing fatigue and the related decrease in cognitive function [35]. Furthermore, some evidence suggests that an environmental light has significant impact on the regulation of breathing through direct signalling in the intrinsically photosensitive retinal circuitry to the suprachiasmatic nucleus pathway [36], possibly further aiding with the ease of physiology modulation.

These studies expose several research gaps and limitations in current literature, including a lack of theoretical basis, neutral control groups, and small sample sizes with fewer than 20 participants, which undermine internal validity and robustness [17,23,25,26,34,37,38]. There is also a scarcity of studies on tertiary intervention strategies for mitigating already-occurred stress [9,17]. Furthermore, the research lacks investigations into using external stimuli to enhance relaxation and assessments of both subjective and objective markers of sleepiness and stress [1,39,40]. Addressing these gaps is crucial for enhancing stress management and developing effective workplace interventions to support employee well-being.

The primary objective of this study is to evaluate the effectiveness of an innovative tertiary (reactive) stress management intervention that addresses the identified gaps in the existing literature. The intervention integrates coherent breathing guided by an external blue light stimulus, delivered by the ÉOS device — a multisensory recovery system developed by Human design Group designed for fatigue and stress management in occupational settings, offering calibrated sessions adaptable to the user’s state and recovery goals — in a Research & Development (R&D) laboratory within Human design Group’s office. The study hypothesizes that the applied reactive stress countermeasure will more effectively mitigate acute stress reactions and sleepiness, compared to passive rest without external stimulation. In this randomized-controlled cross-over double blinded design, parameters are measured through both subjective and objective markers. These markers include questionnaires and measurements of heart rate variability using root mean square successive difference (rMSSD), low-frequency band (LF), and electroencephalography (EEG) of frontal alpha asymmetry (FAA) and theta/beta ratio (S1.4) [41].

## Materials and methods

### Participants

The analysed sample consisted of 58 observations from 29 individuals (32 from 16 women, and 26 from 13 men) who underwent both control and experimental conditions. The mean age was 28.72 (SD = 8.56) years with a positive skew towards younger people under 34 years. All participants were based in France, majority of them were of French nationality and all had completed at least high-school education. During the first session, men’s average body mass index (BMI) was 23.32 (SD = 3.46), and woman’s 22.88 (SD = 3.63). Of all participants, 82.76% reported being non-smokers. The data on anthropometrics and potential confounders are available in the supplementary materials (S3.1. Questionnaires).

### Sampling procedure and exclusion criteria

The study was approved by the CER (Research Ethics Committee) at the Université Fédérale, Toulouse Midi-Pyrénées N° 00011835-2023-0328-648- Université Fédérale de Toulouse IRB # 1. We recruited 30 participants, based on an a priori power analysis (see the Sample size and power calculation section below). Invitations for self-selection to participate as a volunteer were sent by e-mail to employees and interns of Human design Group, SpaceshipFR based at the Centre national d’études spatiales (CNES), and ISAE-SUPAERO. The measurement phase was executed at Human design Group’s R&D laboratory. Participants signed the informed consent document at the beginning of the study, before the first measurement.

Exclusion criteria were cardiovascular anomalies detectable by electrocardiography (ECG), cardiovascular diseases, severe physical illnesses (e.g., fibromyalgia), hypertension, endocrine disorders, current or previous psychopathology, substance abuse, heavy smoking (>10 cigarettes/day) or being on any kind of medication known to affect the hypothalamic-pituitary-adrenal axis (more details in Ráčková et al. [41]; based on Smeets et al. [42]).

### Sample size and power calculation

The minimum required number of individuals was 26, given the power = 0.80, alpha = 0.05, effect size = 0.59, and correlation between measures = 0.85 (calculated for repeated measures ANOVA, between factors using G*Power, see S 1.1. Sample size for further details). We aimed to recruit 30 individuals (15 in each group) to account for potential dropouts. The analysed sample consisted of 58 observations from 29 individuals (15 in intervention first – control second, and 14 in control first – intervention second groups).

### Study design

This study was a two-arm, randomized, controlled, crossover design, developed using the Experimental Design Assistant [43]. An AB/BA design with computer-generated 1:1 block randomization was used [44,45], with the algorithm assigning participants to the ÉOS or control intervention. The researcher could not predict group allocations, as they were unaware of the intervention assigned to each participant’s ID. The treatment periods were separated by 3 weeks to prevent carry-over effects [46]. Data analysts were blinded to the order of interventions to minimize bias [47]. The assessment followed several experimental stages, consistent with previous recommendations [39], and the study reporting adhered to the Consolidated Standards of Reporting Trials (CONSORT) statement [48].

### Interventions

Participants were reminded of a scheduled appointment via email one week prior to the experiment and were provided with the following instructions: maintain a regular sleep routine, abstain from alcohol and vigorous physical activity for the 24 hours prior to the test, refrain from consuming food, coffee, or smoking for the 2 hours prior to the test, and bring appropriate clothing and personal hygiene items.

Upon arrival, participants underwent anthropometric measurements and completed demographic questionnaires (only in the first session). Physiological measurement hardware (EEG and ECG) was then mounted. Baseline questionnaires on confounders, Subjective stress, Karolinska Sleepiness Scale (KSS), and Positive and Negative Affect Schedule (PANAS) were administered in both sessions. A 5-minute baseline physiological data collection followed, with participants sitting calmly, knees at a 90° angle, feet flat on the floor, hands on thighs, and eyes closed.

Participants then underwent the Maastricht Acute Stress Task (MAST), involving an anticipation phase and a stress task with cold water immersion, mental arithmetic, and social evaluation [42]. After the MAST, participants completed questionnaires on subjective stress, KSS, and PANAS. They were then transferred to the ÉOS room to recline on a bed with an elevated back and engage in a relaxation intervention. This involved either exposure to the ÉOS program with blue light and sound cues for coherent breathing, or, for the control group, lying on a bed in complete darkness without any additional stimuli. The final round of questionnaires on Subjective stress, KSS, and PANAS was then administered. The experiment concluded with the removal of physiological devices, and participants were allowed to leave.

All experimental manipulations and measurement were conducted by 2 deliverers (Lucie Ráčková and Inès Butin) with proper training. Training on EEG was delivered by Emma Chabani with Ph.D. in neuroscience and prior experience in EEG measurement. Training on HRV, anthropometry and questionnaires was delivered by Lucie Ráčková, Ph.D. candidate in environmental physiology and MSc. in Anthropology with previous experience in these methods. Training on the MAST was delivered by the authors of the method [42]. More details on the experimental manipulation, including time taken, are detailed in previously published research protocol [49].

## Methods

### ÉOS system

The ÉOS device delivers multisensory recovery sessions through two main components: a LED lighting system emitting calibrated wavelengths of light according to the session scenario and recovery goal, and a spatialized sound environment providing auditory guidance for coherent breathing, meditation, or relaxation. Sessions are calibrated based on the user’s state (fatigue or stress), the selected duration, and relaxation preferences, with or without sleep. In the present study, all participants underwent both the experimental and control conditions as part of the crossover design. In the experimental condition, a single standardized 15-minute relaxation scenario without sleep was assigned by the researchers, during which coherent breathing was guided at 5.5 cycles per minute (0.1 Hz) through synchronized light pulses and auditory cues. Participants did not select or modify any session parameters. The specific parameters of the light and sound stimuli are proprietary.

### Anthropometry

Upon arrival to their first trial, participants’ weight was measured using the Mi Body Composition Scale 2 (Xiaomi Communications Co., Ltd.), and their height was measured using a wall-mounted retractable measuring tape (Yuecoom) with precision to 0.1 cm. The measurement followed the definition from Anthropometry Procedures Manual of National Health and Nutrition Examination Survey [50]. Circumferences were measured using the measuring tape included in the Nox A1s Polysomnograph (Nox Medial USA, Alpharetta, United States) with precision to 0.1 cm. Definitions of measurements followed the WHO STEPwise Approach to Surveillance (STEPS) and can be found in the Waist Circumference and Waist-Hip Ratio report of the WHO Expert Consultation [51].

### Questionnaires

Over the course of the study, participants were administered several questionnaires:

Demographic and confounders were assessed using a form from Laborde et al. [52], modified by requirements from the ethics committee. We asked specifically about: age, gender, smoking, habitual alcohol intake, weight, height, waist-hip ratio, use of cardioactive medication and contraceptives, the prior night’s sleep routine, any intensive physical training 24h prior to the experiment, intake of food or caffeinated drinks 2h before the experiment, alcohol intake 24h prior to the experiment, and the necessity to go to the bathroom.

Subjective stress is a simple scale ranging from 1 to 10 where participants rated how stressed they felt at that moment. This approach has been used in several studies with good sensitivity [53].

Positive and Negative Affect Schedule (PANAS) is a scale evaluating a person’s affect using a 20-item 5-point Likert scale, providing overall scoring for both Positive (PA) and Negative Affect (NA) [54]. We used the French version; the psychometric properties are tested elsewhere [55].

Karolinska Sleepiness Scale (KSS) is a single question 9-point Likert scale measuring current sleepiness level, previously used in studies of shift work, attention, and performance [56], validated in a study using EEG data [57]. We used a French translation of this item.

### Physiological measurements

Participants’ physiological signals were measured using the following devices:

Electrocardiography (ECG) – our study used bipolar ECG electrodes from the Nox A1s Polysomnograph (Nox Medial USA, Alpharetta, United States) with ±1024mV input range, < 3 µVrms noise, 512 kHz sampling rate, 24-bit AD.

Electroencephalography (EEG) – our study used 8 unipolar EEG electrodes from the Nox A1s Polysomnograph (Nox Medial USA, Alpharetta, United States) with ±3.2mV input range < 1 µVrms noise, 512 kHz sampling rate. Electrodes were placed in the areas close to Fp1, Fp2, F3, F4, CZ, and PZ on the scalp and, according to the 10–20 international system, at E1 and E2 around the eyes as a polysomnographic montage. The left/right mastoid (M1 and M2) electrodes were used as lateral reference for the right/left electrodes respectively, while an electrode placed on Fpz was used as a ground.

### Data preprocessing

#### ECG.

As a first step, R-peaks were identified and manually corrected in the ECG time-series using R-DECO [58,59]. R-DECO is an open-source project developed in Matlab specifically for semi-automatic detection of R-peaks and hence, the calculation of R-R intervals. First, an enveloping procedure is used to flatten the ECG signal while enhancing the QRS complexes [60].

Then, a rule-based algorithm along with the Pan-Tompkins algorithm is used to detect the R-peaks in the flattened time-series [58,61]. A first automatic correction of the peaks is performed using a search-back procedure [62]. Using this processing method, the author reported about 99% of correctly detected peaks [58]. However, erroneous or mis-positioned peaks can be present after processing, especially for recording with a high level of noise. For this reason, R-DECO includes a graphical user interface that can be used to correct for algorithm errors by manually adding, removing or adjusting the R-peaks. Quality control of R-peak detection was ensured through systematic visual inspection of the entire ECG time series for each participant by a trained operator, who manually corrected any flagged or visually identified anomalies. A final review of the corrected series was performed to confirm the absence of residual errors prior to HRV feature extraction. This procedure was applied uniformly across all participants and sessions.

The corrected R-R intervals series was then used to calculate HRV features using an in-house script and dedicated python libraries. The root means squared of successive differences (RMSSD) was calculated using the *hrv_time* function of the *neurokit2* library [63]. The power densities of low frequencies, high frequencies, and their ratio were calculated using the *hrvanalysis* library [64,65]. The features of interest were calculated for each period of interest by segmenting the time R-R intervals time series based on the timestamp at the beginning and end of each period (e.g., baseline, MAST instructions, MAST task, first half of ÉOS, second half of ÉOS, post ÉOS).

#### EEG.

EEG data were processed in Python using the *mne* library [66,67]. First, raw data were filtered using a digital band-pass filter (0.1–70 Hz) to remove physiologically implausible frequencies, combined with a notch filter at 50 Hz and 100 Hz to remove noise related to alternating current. Then, power spectral density was calculated using the multitaper method [68] over the entire signal length using 2-second windows with an overlap of one second. The integral over the different EEG rhythms was used to calculate the relative power spectral density (PSD) of each rhythm (alpha: 8–13 Hz, beta: 13–30 Hz, delta: 1–4 Hz, gamma: 30–70 Hz, theta: 4–8 Hz). Then, the values from the 2-second windows were averaged over the period of interest in order to have one value for each rhythm for each period of interest.

Pre-processed data served as a base for calculation of the metrics of interest. First, FAA was calculated by subtracting the natural log of left hemisphere alpha power (F3_M2) from the natural log of right hemisphere alpha power (F4_M1) [69] for each experiment phase and used as a predictor value, in line with recommendations of [70]. Second, the theta/beta ratio was calculated for each period by dividing PSD of the theta power calculated under FP1 by PSD of the beta power calculated under FP1 (resulting in Theta/Beta FP1 variable) and PSD of the theta power calculated under FP2 by PSD of the beta power calculated under FP2 (resulting in Theta/Beta FP2 variable).

### Data analysis

Anonymized data used as input for the analysis script, which allowed us to generate the supplementary material file with RMarkdown is available on Zenodo (https://doi.org/10.5281/zenodo.20596627 or https://zenodo.org/records/20596627). Code for analysis is in the Supplementary Materials.

### Software

An a priori power analysis was performed using G*Power 3.1.9.7 [71]. All other analyses were conducted using RStudio version 2023.12.1.402 [72]. Used packages including citations are in S 8. Package information.

### Repeated measures ANOVA

Repeated measures ANOVA (rmANOVA) was used to examine the assumptions of the study, e.g., correct randomization and absence of carry-over effects. We checked the assumptions of the rmANOVA by investigating for sphericity using Mauchly’s test implemented in the *anova_test* function from the *rstatix* library in R [73] and for normality using the Shapiro-Wilk test using the *shapiro_test* function from the *rstatix* library [73] and Q-Q plots using the *ggqqplot* function within the *ggpubr* library in R [74]. Significant outliers were identified using the *identify_outliers* function from the *rstatix* library in R [73] and were removed from analysis. However, remaining violations of normality were described in the supplementary material and not further addressed here as recent studies showed that rmANOVA is generally robust to them [75].

### Linear mixed-effect models

Linear Mixed-Effect Models (LMEM) were checked for normal distribution of residuals and random effects deviation by plotting residuals and random effects along with the models. Some dependent variables were log-transformed to improve homoskedasticity. Since log-transformation changes the scale of the data, we report confidence intervals (CI) instead of standard errors (SE) for the transformed variables. Some of the models were also improved by identifying outliers using the *identify_outliers* function from the *rstatix* library in R [73] and retaining only those within the range defined by 1.5 times the interquartile range above the third quartile and below the first quartile. Due to the repeated measures design, participant ID was selected as a random effect in all models. Model comparison was conducted by first modelling full models with all of the predictors which we defined as critical during the literature review for this study. We also checked for multicollinearity by examining strong correlations within predictors. Given the limited sample size and Occam’s razor principle, we removed non-significant predictors which did not influence model fit. Successive models differ only in fixed effects. Model comparison was guided by Bayesian Information Criterion (BIC), also considering Akaike Information Criterion (AIC), and then by Likelihood Ratio Tests (LRTs) in case of nested models comparison [76]. All models which are considered for inclusion are reported in the Summary subsection of the respective dependent variable.

## Results

### Participant flow and recruitment

The flow of participants is detailed in the CONSORT diagram (S 1.3. Participant flow). Invitations for participation were sent in March 2023, the measurement phase was executed from May to June 2023.

### Baseline data and study assumptions

We aimed to assess the differences between intervention sequences (ÉOS:Control and Control:ÉOS) and between sessions during non-intervention phases (baseline and post-MAST). We conducted rmANOVA tests with Bonferroni correction for variables measured at baseline and after MAST (Subjective stress, PA, and NA), and paired t-tests for baseline-only variables (KSS). Results showed no significant differences in baseline values between groups or sessions (S4. Study assumptions). Instances of violated normality assumptions are described in S4. as well. Descriptive statistics of baseline data by intervention, session, sex, and intervention sequence are described in S3.1.Questionnaires.

For physiological variables (rMSSD, LF, FAA, Theta/Beta FP1, and Theta/Beta FP2), measurements were taken during baseline, MAST instructions (ECG and EEG), and MAST performance (ECG only due to EEG noise). rmANOVA results did not reject the null hypothesis for any physiological variable concerning grouping. The only exception was FAA, which was lower in the second session during MAST instruction with a large effect size (generalized eta square = 0.36; [77]). Detailed rmANOVA results are in S4. Study assumptions, descriptive statistics of baseline data by period, by period and sex, and by sequence are in SS3.2. Physiological data.

### Experiment results

To assess the effectiveness of the blue light and coherent breathing intervention compared to a neutral condition following induced stress, we conducted a series of analyses targeting positive and negative emotions, sleepiness, and subjective stress. Boxplots visualizing differences in post-relaxation subjective values for ÉOS and Control interventions within the first and second interventions are displayed in Fig 1. We employed LMEM where the physiological metrics were included as fixed factors along with intervention type and post-stress values of the given subjective metrics to examine their interrelationship with the outcomes of interest. Additionally, we constructed models with physiological metrics as dependent variables, which are all listed and described in S5.1.1.-S5.1.6. Baseline and post-stress phases are assumed to be without significant difference (see previous section *Baseline data and study assumptions)*. The experimental phase starts with either the ÉOS or Control intervention. Our analysis uses the period after intervention as the reference level for testing changes between the two groups during all experimental phases.

**Fig 1:**
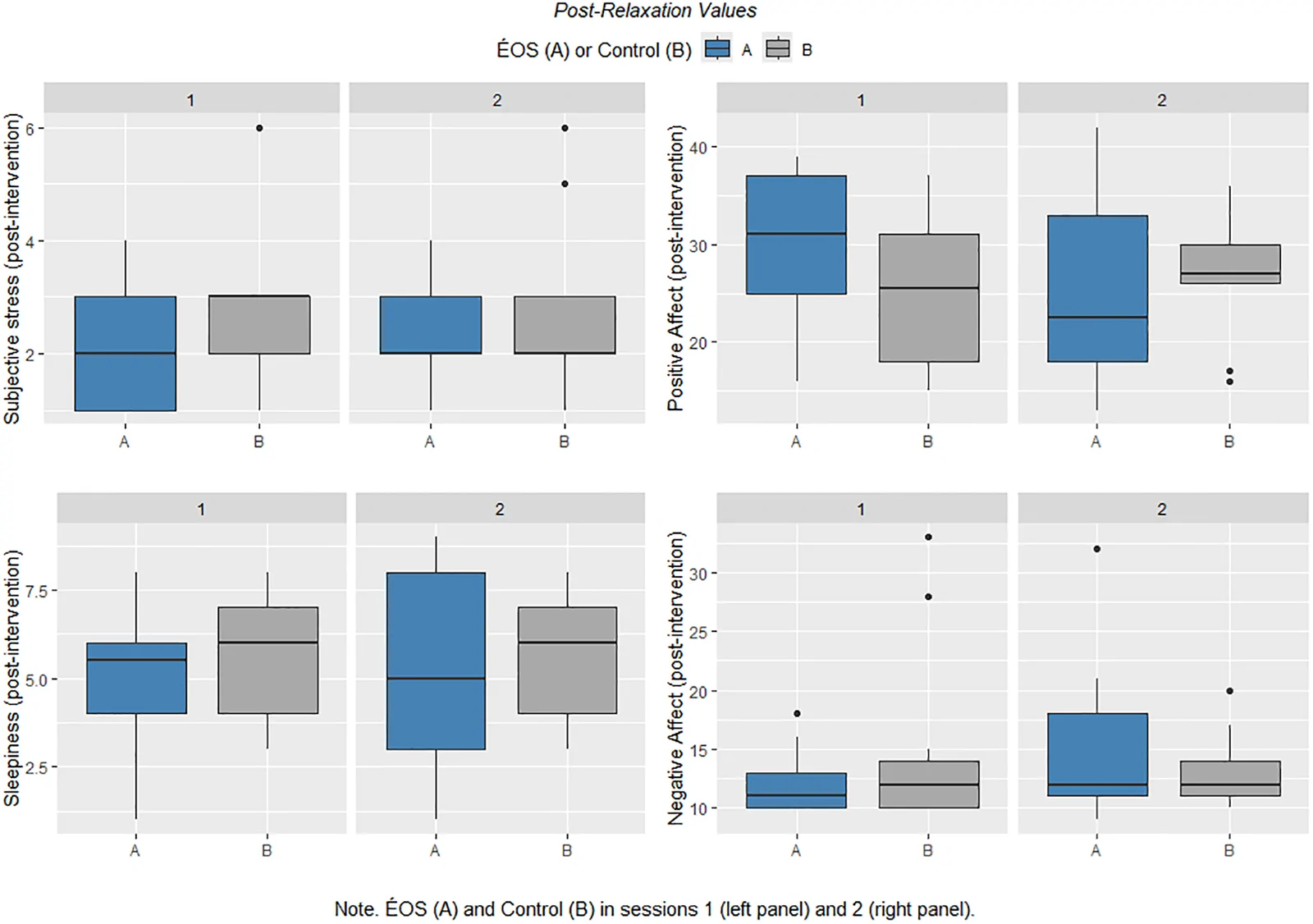
Post-relaxation values by intervention and session. Boxplots of subjective stress, positive affect, negative affect, and sleepiness following the ÉOS (A) and Control (B) interventions, in sessions 1 (left panels) and 2 (right panels).

### Subjective stress

Subjective stress was log-transformed and modelled using LMEM. A model incorporating the post-stress task subjective stress score alongside intervention type, HRV, and EEG data without outliers revealed a significant effect of both intervention type and post-stress subjective stress, along with Theta/Beta FP1 (S5.2.1.7.). A simplified model involving only these predictors (SS5.2.1.8.) indicated that attribution to a control group increased post-relaxation subjective stress by 31.23% (CI 21.41% and 41.83%); each point increase in post-stress subjective stress led to an 8.39% rise in post-relaxation values (CI 6.88% and 15.45%; p < 0.001), and Theta/Beta FP1 decreased subjective stress by 1.43% with borderline significance (CI −3.11% and 0.071%; p = 0.061). However, because the normality of residuals was violated, we advise caution in interpreting these estimates, as they may be less precise (Table 1).

**Table 1:** Linear mixed-effects model (LMEM) of log-transformed subjective stress.

Fixed Effects						
	Est/Beta	SE	95%CI		t	p
Intercept	0.275	0.135	0.007	0.544	2.033	0.045
Control	0.272	0.039	0.194	0.350	6.931	0.000
Pre-ÉOS stress	0.105	0.019	0.067	0.144	5.409	0.000
Theta/Beta FP1	−0.015	0.008	−0.032	0.001	−1.896	0.061
Random Effects						
						S.D.
Participant (Intercept)						0.397
Residual						0.213

*Note.* LMEM (log(Post-ÉOS stress_ij_) = β_0_ + β_1_ ⋅ ÉOS_Control_ij_ + β_2_ ⋅ Pre-ÉOS stress_ij_ + β_3_ ⋅ Theta Beta FP1_ij_ + u_j_ + ϵ_ij_). Log(POST_EOS_stress_ij_) represents the natural logarithm of the post-intervention stress level; the fixed effects (𝛽_0_, 𝛽_1_, 𝛽_2_, 𝛽_3_) account for the influence of intervention type, pre-ÉOS subjective stress, and Theta/Beta FP1 on the log-transformed subjective stress measure; the random effect 𝑢_𝑗_ allows for individual-specific deviations from the overall intercept, capturing variability between subjects; the residual term 𝜖_𝑖𝑗_ captures the within-subject variability, but the test revealed a violation to the assumption of their normal distribution.

A model substituting post-stress phase subjective stress with baseline subjective stress values revealed a significant effect only of Theta/BetaFP1 and had lower performance on BIC; thus, we omitted it from final analysis (S5.2.1. Subjective stress).

### Positive affect

Positive affect, modelled without transformation, was analysed using a LMEM. The full model with the baseline positive affect on the data without EEG outliers unveiled a significant relationship with baseline positive affect, intervention type, and Theta/Beta FP2 (S5.2.2.2. and S5.2.2.3.). The full model using post-stress positive affect found a significant effect of positive affect and intervention type (S5.2.2.4.). A simplified analysis focusing solely on significant predictors (S5.2.2.5.) showed a decrease in post-relaxation positive affect by 0.73 (SE = 0.06, p < 0.001) points, while the control intervention decreased positive affect by 1.85 (SE = 0.54, p < 0.001) points (Table 2). However, because the normality of residuals was violated, we advise caution in interpreting these estimates, as they may be less precise.

**Table 2:** Linear mixed-effects model (LMEM) of positive affect.

Fixed Effects						
	Est/Beta	SE	95%CI		t	p
(Intercept)	6.130	2.04	2.095	10.165	3.006	0.003
Control	−1.845	0.535	−2.903	−0.787	−3.450	0.008
Pre-ÉOS PA	0.733	0.064	0.607	0.859	11.526	0.000
Random Effects						
						S.D.
Participant (Intercept)						4.024
Residual						3.351

*Note.* LMEM (Post-ÉOS PA_ij_ = β_0_ + β_1_ ⋅ ÉOS_Control_ij_ + β_2_ ⋅ Pre-ÉOS PA_ij_ + u_j_ + ϵ_ij_). Post-ÉOS PA_ij_ represents the post-intervention stress level; the fixed effects (𝛽_0_, 𝛽_1_, 𝛽_2_) account for the influence of intervention type, and pre-ÉOS positive affect on the positive affect; the random effect 𝑢_𝑗_ allows for individual-specific deviations from the overall intercept, capturing variability between subjects; the residual term 𝜖_𝑖𝑗_ captures the within-subject variability, but the test revealed a violation to the assumption of their normal distribution.

### Negative affect

Negative affect was log-transformed and modelled using LMEM. The full model examining log-transformed negative affect on the data with no EEG outliers identified significant associations with post-stress negative affect Theta/Beta ratios (Table 3; S5.2.3.7.). A simplified model comprising solely significant predictors and excluding outliers (S5.2.3.8.) revealed statistically significant relationships for post-stress negative affect (1.05%, CI 0.64% and 1.45%, p < 0.001), Theta/Beta FP1 (−1.18%, CI −1.74% and −0.61%, p < 0.001), and Theta/Beta FP2 (0.70%, CI 0.31% and 1.20%, p = 0.005). The effect sizes are marginal, and the violation of residual normality is significant (p < 0.001).

**Table 3:** Linear mixed-effects model (LMEM) of log-transformed negative affect.

Fixed Effects						
	Est/Beta	SE	95%CI		t	p
(Intercept)	2.325	0.054	2.219	2.431	43.443	0.000
Pre-ÉOS NA	0.010	0.002	0.006	0.014	5.166	0.000
Theta/Beta FP1	−0.012	0.003	−0.018	−0.006	−4.093	0.000
Theta/Beta FP2	0.007	0.002	0.002	0.012	2.847	0.005
Random Effects						
						S.D.
Participant (Intercept)						0.142
Residual						0.063

*Note.* LMEM (log(Post-ÉOS NA_ij_) = β_0_ + β_1_ ⋅ Pre-ÉOS NA _ij_ + β_2_ ⋅ Theta Beta FP1 _ij_ + β_3_ ⋅ Theta Beta FP2_ij_ + u_j_ + ϵ_ij_). Log(Post-ÉOS NA_ij_) represents the natural logarithm of the post-intervention negative affect level; the fixed effects (𝛽_0_, 𝛽_1_, 𝛽_2_, 𝛽_3_) account for the influence of pre-ÉOS negative affect, Theta/Beta FP1, and Theta/Beta FP2 on the log-transformed subjective stress measure; the random effect 𝑢_𝑗_ allows for individual-specific deviations from the overall intercept, capturing variability between subjects; the residual term 𝜖_𝑖𝑗_ captures the within-subject variability, but the test revealed a violation to the assumption of their normal distribution.

A model with baseline negative affect instead of post-stress negative affect on the data without outliers showed similar results (S5.2.3.4.), but the model with post-stress negative affect had higher performance on AIC, BIC, and log likelihood and, thus, we excluded the model with baseline negative affect from final analysis.

### Subjective sleepiness

Sleepiness was analysed utilizing a LMEM. Findings from the full model (S5.2.4.2.) revealed significant effect of intervention, baseline sleepiness, and HRV variables. A simpler model (S5.2.4.3.) utilizing only those significant predictors revealed a decrease in subjective sleepiness by 0.84 points (SE = 0.17, p < 0.001) compared to the control condition, and an increase by 0.59 points (SE = 0.07, p < 0.001) with each point in baseline sleepiness. Moreover, each ms^2^ of LF exhibited a positive association with sleepiness, increasing it by 0.0002 points (SE = 0.003, p = 0.002), whereas each ms of rMSSD was inversely related to sleepiness (p = 0.009), decreasing it by 0.009 points (SE = 0.003) per ms (Table 4). Normality of the models’ residuals was violated.

**Table 4:** Linear mixed-effects model (LMEM) of sleepiness.

Fixed Effects						
	Est/Beta	SE	95%CI		t	p
(Intercept)	2.314	0.467	1.390	3.238	4.953	0.000
Control	0.842	0.165	0.516	1.169	5.102	0.000
Baseline KSS	0.594	0.064	0.467	0.720	9.286	0.000
HRV rMSSD	−0.009	0.004	−0.016	−0.002	−2.673	0.009
HRVLF	0.000	0.000	0.000	0.000	3.126	0.002
Random Effects						
						S.D.
Participant (Intercept)						0.155
Residual						0.894

*Note.* LMEM (Post-ÉOS KSS_ij_ = β_0_ + β_1_ ⋅ ÉOS_Control_ij_ + β_2_ ⋅ Baseline KSS_ij_ + β_3_ ⋅ HRV rMSSD_ij_ + β_4_ ⋅ HRV lf_ij_ + u_j_ + ϵ_ij_). Post-ÉOS KSS_ij_ represents the post-intervention subjective sleepiness level; the fixed effects (𝛽_0_, 𝛽_1_, 𝛽_2,_ 𝛽_3,_ 𝛽_4_) account for the influence of intervention type, baseline sleepiness, rMSSD, and LF values on subjective sleepiness; the random effect 𝑢_𝑗_ allows for individual-specific deviations from the overall intercept, capturing variability between subjects; the residual term 𝜖_𝑖𝑗_ captures the within-subject variability, but the test revealed a violation to the assumption of their normal distribution.

## Discussion

Our results demonstrated that a 0.1 Hz coherent breathing intervention, guided by blue light, was more effective than a control condition in reducing subjective stress (−31.23%, CI 21.41% and 41.83%) and sleepiness (−0.84 points, SE = 0.17), and at promoting positive affect (1.85 points, SE = 0.54) after an episode of multisource acute stress in a workplace laboratory setting. However, there was no significant impact on negative affect. These findings partially support our hypothesis that the intervention would outperform the control condition [41] and suggest that this intervention can effectively enhance mood and reduce stress while preventing sleepiness after an episode of an acute stress.

This adds a novel perspective to previous studies. For instance, Minguillon et al. [20] did not find significant stress reduction differences in their study, perhaps due to their inclusion of blue light in both experimental sessions. Our results align with earlier findings on blue light’s positive effect on mood in controlled experiments with blue [78] or blue-enriched light [79,80], and its use in treating seasonal affective disorder [81], although the latter report non-uniform results. It should be noted that these types of exposures are recommended during daytime or complemented with sufficient daytime external light exposure when used at night [82] to prevent negative effects on circadian rhythm and sleep [83].

Regarding sleepiness, our intervention was effective in sleepiness reduction. This indicates that the intervention may effectively prevent the induction of sleepiness in post-stress relaxation measures. This mirrors findings from previous studies using blue light [23,25,84]. To our knowledge, there are no experimental studies on subjective sleepiness after coherent breathing interventions for direct comparison. Additionally, a meta-analysis on slow-paced breathing suggests there is no significant effect on subjective stress [85], with some studies even reporting negative effects on positive arousal [86]. Our results indicate that combining blue-light guidance with coherent breathing may offset these negative effects.

Another important finding is that, during the model selection phase, subjective measures taken post-stress had higher predictive power than baseline values (all models within the selection procedure are detailed in S5. Experimental phase modelling), highlighting the importance of individual reactivity to stress in determining the intervention’s effectiveness. This suggests that those more impacted by stress may benefit more from reactive countermeasures.

The lack of association between HRV and emotions or subjective stress in our study contrasts with extensive literature [85,87–89]. Notwithstanding, such a comparison is complicated due to the differences in study design, type of stimuli, and analyses conducted (detailed in reviews by Danilenko [90] and Katsuura and Lee [91]). However, we found a significant negative relationship with rMSSD and subjective sleepiness as well as a positive relationship with LF and subjective sleepiness, indicating evidence of physiological arousal coupling with subjective experience. Altogether, it seems like the specific combination of different stimuli introduces another level of interaction with potentially different effects than blue light and coherent breathing individually, which would deserve further investigation in a larger study.

As for the selected EEG variables of FAA and theta/beta ratios, our study identified significant but marginal effects of theta/beta ratio on negative affect. Inclusion of theta/beta ratios tended to improve the model’s performance, according to BIC criteria. Theta/beta ratio has been extensively linked to attention deficit and hyperactivity disorder [92,93]. Our results, thus, may suggest that individuals with hemispheric asymmetry in the theta/beta ratio may be more prone to express negative emotions. The lack of association of FAA with variables is in alignment with some previous studies which did not find an association between alpha asymmetry and stress response either [94,95]. However, other research found such evidence [96–98], suggesting the need of further investigation of this relationship.

Our study faced some limitations. The normality of residuals was violated in all models. This means there are possible prediction errors and reduced estimate precision [99], especially with a relatively small sample size compared to the number of predictors [76]. Our study also did not account for multiple hypotheses testing and has to be considered separately. While a cross-over design enhances statistical power and estimate precision [47,100], some effects might remain undetected due to their smaller size or untested hidden factors. Future studies should replicate our design with a larger, presumably more ethnically diverse population as there is some incidence that the effects may not be dependent on ethnic origin [101]. Another potential next step may involve the comparison of different light conditions (darkness, natural, and artificial light), and in more stratified grouping (coherent breathing without light guidance, or light without breathing modulation) to isolate and delineate the specific effects of each intervention type. Assessing long-term effects of these in-office interventions and including cognitive tests post-intervention could further elucidate the practical impact of these stress countermeasures. Notably, two recent studies further support these future directions. Canazei et al. [102] demonstrated that light-guided resonant breathing significantly enhanced autonomic recovery (RMSSD) following acute stress induction in a simulated office environment, corroborating our findings and underlining the potential of light-guided breathing as an occupational stress recovery strategy. DeGraves et al. [103] showed that coherent breathing is a feasible and effective stress management strategy for long-term care workers over an 8-week period, suggesting applicability beyond acute stress contexts.

## Conclusion

In conclusion, our study demonstrates that a 0.1 Hz coherent breathing intervention, guided by blue light, effectively reduces subjective stress and sleepiness while promoting positive affect in a workplace laboratory setting. These findings align with and expand upon previous research, suggesting the potential for such interventions to provide short-term stress relief and mood enhancement without inducing sleepiness. The lack of association between HRV and emotional measures highlights the complexity of these interactions and the need for further investigation. Future research should explore the long-term effects of these interventions, their application across diverse populations, and their integration with other light and breathing modalities to enhance their effectiveness.

## Supporting information

S1 File EOS_Supplementary. Supplementary file contains details about study design, code for analysis and supplementary analyses.
(DOCX)
