# Supplementary material for: The effects of coherent breathing and blue light after an acute stress on sleepiness and the stress response in a workplace setting: A randomized double-blind cross-over trial
Source: PLoS One. 2026 Sep 30;21(9):e0357293. doi: 10.1371/journal.pone.0357293 (PMC13626309; doi:10.1371/journal.pone.0357293)
Supplement: Supplementary file 1 [file pone.0357293.s001.docx]

title: “EOS analysis”

author: “Lucie Ráčková, Pavel Morcinek, Markéta Makarová”

date: ‘2024-05-03’

output: html_document

# EOS - supplementary

This is the supplementary material for the study: *Effects of coherent breathing and blue light on sleepiness and stress response in a work setting: a randomized controlled cross-over trial.*

R:studio version

$mode [1] "desktop" $version [1] ‘2023.12.1.402’ $long_version [1] "2023.12.1+402" $release_name [1] "Ocean Storm"

## 1. Study design

### 1. 1. Sample size

The effect size was estimated based on the results from an experiment using blue, green and no light by Cajochen et al. (2005) who found a large effect on heart rate and on subjective sleepiness (partial eta squared 0.279 and 0.17, calculated from reported F value using Cohen’s method (Lakens, 2013)). By rule of thumb, a large effect for Cohen’s F in multiple regression is 0.59 (Ellis, 2010; Kotrlik & Williams, 2003). Previous studies also revealed strong positive correlation (r > 0.70) in repeated measures of heart rate and subjective sleepiness (r > 0.60) and PANAS (r > 0.84) (Cleophas et al., 2009; Crawford & Henry, 2004; Kleiger et al., 1991).

Sample size was calculated using G*Power 3.1.9.7 software.

F tests - ANOVA: Repeated measures, between factors

| **Analysis:** | A priori: Compute required sample size |  |  |
| --- | --- | --- | --- |
|  |  |  |  |
| **Input:** | Effect size f | = | 0,59 |
|  | α err prob | = | 0,05 |
|  | Power (1-β err prob) | = | 0,85 |
|  | Number of groups | = | 2 |
|  | Number of measurements | = | 2 |
|  | Corr among rep measures | = | 0,85 |
|  |  |  |  |
| **Output:** | Noncentrality parameter λ | = | 9,7844324 |
|  | Critical F | = | 4,2596773 |
|  | Numerator df | = | 1 |
|  | Denominator df | = | 24 |
|  | Total sample size | = | 26 |
|  | Actual power | = | 0,8509963 |

Distribution plot for given input parameters

Plot of power vs- sample size

*References*

Cajochen, C., Munch, M., Kobialka, S., Krauchi, K., Steiner, R., Oelhafen, P., … & Wirz-Justice, A. (2005). High sensitivity of human melatonin, alertness, thermoregulation, and heart rate to short wavelength light. The journal of clinical endocrinology & metabolism, 90(3), 1311-1316.

Cleophas, T. J., Zwinderman, A. H., & van Ouwerkerk, B. M. (2009). Methods for analysing cardiovascular studies with repeated measures. Netherlands heart journal, 17, 429-433.

Crawford, J. R., & Henry, J. D. (2004). The Positive and Negative Affect Schedule (PANAS): Construct validity, measurement properties and normative data in a large non‐clinical sample. British journal of clinical psychology, 43(3), 245-265.

Ellis, P. D. (2010). The essential guide to effect sizes: Statistical power, meta-analysis, and the interpretation of research results. Cambridge university press.

Kleiger, R. E., Bigger, J. T., Bosner, M. S., Chung, M. K., Cook, J. R., Rolnitzky, L. M., … & Fleiss, J. L. (1991). Stability over time of variables measuring heart rate variability in normal subjects. The American journal of cardiology, 68(6), 626-630.

Kotrlik, J. W. K. J. W., & Williams, H. A. W. H. A. (2003). The incorporation of effect size in information technology, learning, information technology, learning, and performance research and performance research. Information Technology, Learning, and Performance Journal, 21(1), 1.

Lakens, D. (2013). Calculating and reporting effect sizes to facilitate cumulative science: a practical primer for t-tests and ANOVAs. Frontiers in psychology, 4, 62627.

### 1. 2. Study design

The study design was planned using EDA (Experimental Design Assistant: RRID:SCR_017019, [**https://eda.nc3rs.org.uk**](https://eda.nc3rs.org.uk))

Experimental Design schema, accessible also from: <https://eda.nc3rs.org.uk:443/eda/modelPublicExport/index/5C7D8CBB51173BAE455DDEE67E0DDB9B>

### 1. 3. Participant flow

The flow was described using the CONSORT diagram in R software.

### 1. 4. Biomarker selection rationale

We included subjective data on subjective sleepiness, perceived stress, physiological arousal, positive and negative affect, which are crucial for individual stress responses and work performance (Leger & Charles, 2020; Zapf et al., 2021). We complemented subjective markers with objective indicators from heart rate and EEG signals. Specifically, we selected:

- the root mean square successive difference (rMSSD) to assess short-term heart rate variability influenced by *nervus vagus*, and
- the low-frequency band spectral measure, reflecting baroreceptor activity (Pereira et al., 2017; Shaffer & Ginsberg, 2017).
- For neurobiological markers, we used:
- frontal alpha asymmetry (FAA) and
- the theta/beta ratio.
- Changes in EEG asymmetry reflect stress responses, with FAA indicating cognitive processes like approach motivation and emotional states (Goodman et al., 2013; Smith et al., 2017; Zhang et al., 2018). High FAA (right-lateralization) indicates withdrawal-related states like sadness and anxiety, while low FAA (left-lateralization) indicates approach-oriented states like anger and self-control (Mauss & Robinson, 2009; Smith et al., 2017). The theta/beta, used in studies of attention and sleepiness (Fernandez Rojas et al., 2020; Finnigan & Robertson, 2011), indicates a balance between motivational and cognitive control systems, with a higher ratio indicating dominant seeking motivation and lower attentional control (Putman et al., 2014), reflecting poorer adaptive responses in challenging environments (Schutte et al., 2017).

*References*

Fernandez Rojas, R., Debie, E., Fidock, J., Barlow, M., Kasmarik, K., Anavatti, S., Garratt, M., & Abbass, H. (2020). Electroencephalographic Workload Indicators During Teleoperation of an Unmanned Aerial Vehicle Shepherding a Swarm of Unmanned Ground Vehicles in Contested Environments. *Frontiers in Neuroscience*, *14*. <https://doi.org/10.3389/fnins.2020.00040>

Finnigan, S., & Robertson, I. H. (2011). Resting EEG theta power correlates with cognitive performance in healthy older adults. *Psychophysiology*, *48*(8), 1083–1087. <https://doi.org/10.1111/j.1469-8986.2010.01173.x>

Goodman, R. N., Rietschel, J. C., Lo, L.-C., Costanzo, M. E., & Hatfield, B. D. (2013). Stress, emotion regulation and cognitive performance: The predictive contributions of trait and state relative frontal EEG alpha asymmetry. *International Journal of Psychophysiology*, *87*(2), 115–123. <https://doi.org/10.1016/j.ijpsycho.2012.09.008>

Leger, K. A., & Charles, S. T. (2020). Affective recovery from stress and its associations with sleep. *Stress and Health*, *36*(5), 693–699. <https://doi.org/10.1002/smi.2966>

Mauss, I. B., & Robinson, M. D. (2009). Measures of emotion: A review. *Cognition & Emotion*, *23*(2), 209–237. <https://doi.org/10.1080/02699930802204677>

Pereira, T., Almeida, P. R., Cunha, J. P. S., & Aguiar, A. (2017). Heart rate variability metrics for fine-grained stress level assessment. *Computer Methods and Programs in Biomedicine*, *148*, 71–80. <https://doi.org/10.1016/j.cmpb.2017.06.018>

Putman, P., Verkuil, B., Arias-Garcia, E., Pantazi, I., & van Schie, C. (2014). EEG theta/beta ratio as a potential biomarker for attentional control and resilience against deleterious effects of stress on attention. *Cognitive, Affective, & Behavioral Neuroscience*, *14*(2), 782–791. <https://doi.org/10.3758/s13415-013-0238-7>

Schutte, I., Kenemans, J. L., & Schutter, D. J. L. G. (2017). Resting-state theta/beta EEG ratio is associated with reward- and punishment-related reversal learning. *Cognitive, Affective, & Behavioral Neuroscience*, *17*(4), 754–763. <https://doi.org/10.3758/s13415-017-0510-3>

Shaffer, F., & Ginsberg, J. P. (2017). An Overview of Heart Rate Variability Metrics and Norms. *Frontiers in Public Health*, *5*(September), 1–17. <https://doi.org/10.3389/fpubh.2017.00258>

Smith, E. E., Reznik, S. J., Stewart, J. L., & Allen, J. J. B. (2017). Assessing and conceptualizing frontal EEG asymmetry: An updated primer on recording, processing, analyzing, and interpreting frontal alpha asymmetry. *International Journal of Psychophysiology*, *111*, 98–114. <https://doi.org/10.1016/j.ijpsycho.2016.11.005>

Zapf, D., Kern, M., Tschan, F., Holman, D., & Semmer, N. K. (2021). Emotion Work: A Work Psychology Perspective. *Annual Review of Organizational Psychology and Organizational Behavior*, *8*(1), 139–172. <https://doi.org/10.1146/annurev-orgpsych-012420-062451>

Zhang, X., Bachmann, P., Schilling, T. M., Naumann, E., Schächinger, H., & Larra, M. F. (2018). Emotional stress regulation: The role of relative frontal alpha asymmetry in shaping the stress response. *Biological Psychology*, *138*, 231–239. <https://doi.org/10.1016/j.biopsycho.2018.08.007>

## 2. Data loading

The purpose of this analysis is to determine whether the ÉOS intervention was effective in reducing physiological and subjective stress without contributing to sleepiness. The study is blinded for analysis and therefore, we test for differences between groups A and B during the experimental phases (baseline; pre_EOS; EOS_1; EOS_2; post_EOS).

1. First, we load the data and perform an exploratory analysis.
2. Second, we test for study assumptions using repeated measures ANOVA on baseline and pre_EOS values.
3. Third, we perform the analysis using linear mixed-effect models to explore changes between the last 3 phases (EOS_1; EOS_2; post_EOS), using the last phase as a reference.

The data to be loaded into R are

- Data_Cleaned_Questionnair_EOS_LAB.xlsx
- EOSdata.RData
- alpha_asymmetry.xls
- Theta_on_Beta_FP1.xls
- Theta_on_Beta_FP2.xls

```
## New names:
## • `` -> `...1`
## • `` -> `...2`
## • `` -> `...3`
## • `` -> `...4`
## • `` -> `...5`
## • `` -> `...141`
## Warning: There were 12 warnings in `mutate()`.
## The first warning was:
## ℹ In argument: `anthro_weight = .Primitive("as.double")(anthro_weight)`.
## Caused by warning:
## ! NAs introduced by coercion
## ℹ Run `dplyr::last_dplyr_warnings()` to see the 11 remaining warnings.
## New names:
## • `` -> `...1`
```

## 3. Descriptive statistics

The descriptive statistics for subjective, demographic, and physiological data are described for:

- both sexes,
- for women,
- and for men separately.

Tables are provided for subsets of the following:

- first session,
- second session,
- ÉOS intervention,
- and control intervention.

### 3.1. Questionnaires

```
library(knitr)

# Subset the data where info$Session == "1"
subset_data <- info[info$Session == "1", ]

# Create a table for demo_sex
table_demo_sex <- table(subset_data$demo_sex)

# Create a proportion table for demo_sex
prop_table_demo_sex <- prop.table(table_demo_sex)

# Create a table for demo_smoke
table_demo_smoke <- table(subset_data$demo_smoke)

# Create a proportion table for demo_smoke
prop_table_demo_smoke <- prop.table(table_demo_smoke)

knitr::kable(table_demo_sex, caption = "Table for sex (Session 1)")
```

Table for sex (Session 1)

| Var1 | Freq |
| --- | --- |
| Femme | 16 |
| Homme | 13 |
| NA | 0 |

knitr::kable(prop_table_demo_sex, caption = "Proportion table for sex (Session 1)")

Proportion table for sex (Session 1)

| Var1 | Freq |
| --- | --- |
| Femme | 0.5517241 |
| Homme | 0.4482759 |
| NA | 0.0000000 |

knitr::kable(table_demo_smoke, caption = "Table for smoking (Session 1)")

Table for smoking (Session 1)

| Var1 | Freq |
| --- | --- |
| 0 | 24 |
| 1 | 5 |
| NA | 0 |

knitr::kable(prop_table_demo_smoke, caption = "Proportion table for smoking (Session 1)")

Proportion table for smoking (Session 1)

| Var1 | Freq |
| --- | --- |
| 0 | 0.8275862 |
| 1 | 0.1724138 |
| NA | 0.0000000 |

Summary statistics for Intervention

|  | vars | n | mean | sd | median | trimmed | mad | min | max | range | skew | kurtosis | se |
| --- | --- | --- | --- | --- | --- | --- | --- | --- | --- | --- | --- | --- | --- |
| Subject* | 1 | 29 | 15.620690 | 8.9339393 | 16.0000 | 15.64000 | 11.860800 | 1.00000 | 30.000 | 29.00000 | -0.0370590 | -1.3617833 | 1.6589909 |
| EOS_Control* | 2 | 29 | 1.000000 | 0.0000000 | 1.0000 | 1.00000 | 0.000000 | 1.00000 | 1.000 | 0.00000 | NaN | NaN | 0.0000000 |
| Session* | 3 | 29 | 1.517241 | 0.5085476 | 2.0000 | 1.52000 | 0.000000 | 1.00000 | 2.000 | 1.00000 | -0.0654682 | -2.0633373 | 0.0944349 |
| anthro_weight | 4 | 29 | 68.324138 | 14.0690810 | 64.1000 | 67.59600 | 14.232960 | 45.90000 | 100.000 | 54.10000 | 0.5908813 | -0.6974584 | 2.6125627 |
| anthro_height | 5 | 29 | 171.772414 | 10.3417633 | 172.4000 | 171.60800 | 11.564280 | 153.40000 | 191.200 | 37.80000 | -0.0548056 | -1.0211582 | 1.9204172 |
| anthro_waist | 6 | 29 | 78.310345 | 10.2892574 | 77.5000 | 77.86000 | 11.860800 | 63.00000 | 102.100 | 39.10000 | 0.3987986 | -0.7827156 | 1.9106671 |
| baseline_KSS | 7 | 29 | 4.827586 | 1.6917650 | 5.0000 | 4.76000 | 1.482600 | 2.00000 | 8.000 | 6.00000 | 0.3033781 | -1.1306814 | 0.3141529 |
| anthro_hips | 8 | 29 | 99.641379 | 8.1016099 | 99.5000 | 99.26800 | 8.154300 | 82.00000 | 117.600 | 35.60000 | 0.4117867 | -0.1216440 | 1.5044312 |
| baseline_stress_state | 9 | 29 | 3.551724 | 1.7440963 | 3.0000 | 3.48000 | 1.482600 | 1.00000 | 7.000 | 6.00000 | 0.2869897 | -0.8746480 | 0.3238706 |
| POST_EOS_stress | 10 | 29 | 2.068966 | 0.9231559 | 2.0000 | 2.00000 | 1.482600 | 1.00000 | 4.000 | 3.00000 | 0.3968202 | -0.8719488 | 0.1714257 |
| POST_EOS_PA | 11 | 29 | 27.275862 | 8.8110991 | 27.0000 | 27.36000 | 13.343400 | 13.00000 | 42.000 | 29.00000 | -0.0035090 | -1.3983125 | 1.6361800 |
| POST_EOS_NA | 12 | 29 | 13.068965 | 4.7202530 | 12.0000 | 12.28000 | 2.965200 | 9.00000 | 32.000 | 23.00000 | 2.3996777 | 6.3999588 | 0.8765290 |
| POST_EOS_KSS | 13 | 29 | 5.068966 | 2.2349662 | 5.0000 | 5.12000 | 1.482600 | 1.00000 | 9.000 | 8.00000 | -0.1388305 | -0.8876922 | 0.4150228 |
| PRE_EOS_stress | 14 | 29 | 4.206897 | 1.6339984 | 4.0000 | 4.24000 | 1.482600 | 1.00000 | 7.000 | 6.00000 | -0.2265188 | -0.7532889 | 0.3034259 |
| PRE_EOS_PA | 15 | 29 | 28.965517 | 8.1305542 | 27.0000 | 28.96000 | 10.378200 | 13.00000 | 46.000 | 33.00000 | 0.0472501 | -0.9258590 | 1.5098060 |
| PRE_EOS_NA | 16 | 29 | 17.413793 | 6.2821586 | 15.0000 | 16.84000 | 5.930400 | 10.00000 | 34.000 | 24.00000 | 0.8378632 | -0.0839973 | 1.1665676 |
| baseline_PA | 17 | 29 | 31.482759 | 7.2191248 | 32.0000 | 31.48000 | 7.413000 | 17.00000 | 45.000 | 28.00000 | -0.0079415 | -0.7513104 | 1.3405578 |
| baseline_NA | 18 | 29 | 16.448276 | 6.4949772 | 14.0000 | 15.72000 | 4.447800 | 10.00000 | 34.000 | 24.00000 | 1.2572767 | 0.5035706 | 1.2060870 |
| demo_age | 19 | 29 | 28.724138 | 8.5602760 | 26.0000 | 27.84000 | 5.930400 | 19.00000 | 51.000 | 32.00000 | 1.1658740 | 0.2125241 | 1.5896033 |
| demo_sex* | 20 | 29 | 1.448276 | 0.5061202 | 1.0000 | 1.44000 | 0.000000 | 1.00000 | 2.000 | 1.00000 | 0.1973466 | -2.0274399 | 0.0939842 |
| demo_smoke* | 21 | 29 | 1.172414 | 0.3844259 | 1.0000 | 1.12000 | 0.000000 | 1.00000 | 2.000 | 1.00000 | 1.6455195 | 0.7366627 | 0.0713861 |
| BMI | 22 | 29 | 23.042318 | 3.5146116 | 22.5183 | 23.02454 | 3.151376 | 16.43841 | 29.502 | 13.06359 | 0.2694675 | -0.9064309 | 0.6526470 |

Summary statistics for Control

|  | vars | n | mean | sd | median | trimmed | mad | min | max | range | skew | kurtosis | se |
| --- | --- | --- | --- | --- | --- | --- | --- | --- | --- | --- | --- | --- | --- |
| Subject* | 1 | 29 | 15.620690 | 8.9339393 | 16.00000 | 15.640000 | 11.860800 | 1.0000 | 30.00000 | 29.00000 | -0.0370590 | -1.3617833 | 1.6589909 |
| EOS_Control* | 2 | 29 | 2.000000 | 0.0000000 | 2.00000 | 2.000000 | 0.000000 | 2.0000 | 2.00000 | 0.00000 | NaN | NaN | 0.0000000 |
| Session* | 3 | 29 | 1.482759 | 0.5085476 | 1.00000 | 1.480000 | 0.000000 | 1.0000 | 2.00000 | 1.00000 | 0.0654682 | -2.0633373 | 0.0944349 |
| anthro_weight | 4 | 28 | 68.814286 | 13.9945416 | 66.25000 | 68.166667 | 14.010570 | 44.0000 | 99.50000 | 55.50000 | 0.4864212 | -0.6750509 | 2.6447198 |
| anthro_height | 5 | 28 | 171.825000 | 10.3328181 | 173.00000 | 171.729167 | 10.822980 | 153.0000 | 191.40000 | 38.40000 | -0.0878105 | -0.9956795 | 1.9527191 |
| anthro_waist | 6 | 28 | 79.025000 | 10.3073374 | 79.40000 | 78.633333 | 10.304070 | 62.2000 | 106.00000 | 43.80000 | 0.3671086 | -0.2466946 | 1.9479037 |
| baseline_KSS | 7 | 28 | 4.857143 | 1.5566889 | 5.00000 | 4.833333 | 1.482600 | 2.0000 | 8.00000 | 6.00000 | 0.1715747 | -1.0517915 | 0.2941866 |
| anthro_hips | 8 | 28 | 100.064286 | 8.2373681 | 98.90000 | 99.933333 | 8.228430 | 80.5000 | 123.30000 | 42.80000 | 0.3454394 | 0.8516412 | 1.5567162 |
| baseline_stress_state | 9 | 28 | 3.285714 | 1.9409265 | 3.00000 | 3.166667 | 1.482600 | 1.0000 | 7.00000 | 6.00000 | 0.7530011 | -0.8010194 | 0.3668006 |
| POST_EOS_stress | 10 | 28 | 2.750000 | 1.3505829 | 3.00000 | 2.625000 | 1.482600 | 1.0000 | 6.00000 | 5.00000 | 0.9622386 | 0.3789941 | 0.2552362 |
| POST_EOS_PA | 11 | 29 | 25.896552 | 6.5810596 | 27.00000 | 25.920000 | 5.930400 | 15.0000 | 37.00000 | 22.00000 | -0.2480841 | -1.1478918 | 1.2220721 |
| POST_EOS_NA | 12 | 29 | 13.413793 | 5.3151888 | 12.00000 | 12.320000 | 2.965200 | 10.0000 | 33.00000 | 23.00000 | 2.4222272 | 5.4578417 | 0.9870058 |
| POST_EOS_KSS | 13 | 29 | 5.620690 | 1.6564548 | 6.00000 | 5.640000 | 1.482600 | 3.0000 | 8.00000 | 5.00000 | -0.1820310 | -1.2884982 | 0.3075959 |
| PRE_EOS_stress | 14 | 28 | 4.857143 | 1.9760470 | 5.00000 | 4.875000 | 2.223900 | 1.0000 | 8.00000 | 7.00000 | 0.0521427 | -0.9960771 | 0.3734378 |
| PRE_EOS_PA | 15 | 28 | 29.464286 | 7.5153283 | 30.00000 | 29.500000 | 7.413000 | 12.0000 | 46.00000 | 34.00000 | -0.1591795 | -0.4212023 | 1.4202635 |
| PRE_EOS_NA | 16 | 28 | 18.464286 | 7.4955895 | 16.50000 | 17.791667 | 5.189100 | 10.0000 | 36.00000 | 26.00000 | 0.9248292 | -0.2838652 | 1.4165333 |
| baseline_PA | 17 | 28 | 31.571429 | 6.4946050 | 31.00000 | 31.541667 | 6.671700 | 20.0000 | 44.00000 | 24.00000 | -0.0176720 | -0.8382131 | 1.2273650 |
| baseline_NA | 18 | 28 | 15.392857 | 6.1664522 | 14.00000 | 14.458333 | 4.447800 | 10.0000 | 37.00000 | 27.00000 | 1.8239874 | 3.3194305 | 1.1653499 |
| demo_age | 19 | 28 | 29.000000 | 8.8023566 | 26.00000 | 28.083333 | 5.930400 | 19.0000 | 52.00000 | 33.00000 | 1.1455473 | 0.1785190 | 1.6634890 |
| demo_sex* | 20 | 29 | 1.517241 | 0.5744991 | 1.00000 | 1.480000 | 0.000000 | 1.0000 | 3.00000 | 2.00000 | 0.4813523 | -0.9064946 | 0.1066818 |
| demo_smoke* | 21 | 29 | 1.241379 | 0.5109635 | 1.00000 | 1.160000 | 0.000000 | 1.0000 | 3.00000 | 2.00000 | 1.8865275 | 2.7159673 | 0.0948835 |
| BMI | 22 | 28 | 23.191802 | 3.4655556 | 22.48471 | 23.224765 | 3.054597 | 15.7203 | 29.97277 | 14.25247 | 0.0924694 | -0.6878455 | 0.6549285 |

Summary statistics for Session 1 and both sexes

|  | vars | n | mean | sd | median | trimmed | mad | min | max | range | skew | kurtosis | se |
| --- | --- | --- | --- | --- | --- | --- | --- | --- | --- | --- | --- | --- | --- |
| Subject* | 1 | 29 | 15.620690 | 8.9339393 | 16.00000 | 15.640000 | 11.860800 | 1.0000 | 30.00000 | 29.00000 | -0.0370590 | -1.3617833 | 1.6589909 |
| EOS_Control* | 2 | 29 | 1.517241 | 0.5085476 | 2.00000 | 1.520000 | 0.000000 | 1.0000 | 2.00000 | 1.00000 | -0.0654682 | -2.0633373 | 0.0944349 |
| Session* | 3 | 29 | 1.000000 | 0.0000000 | 1.00000 | 1.000000 | 0.000000 | 1.0000 | 1.00000 | 0.00000 | NaN | NaN | 0.0000000 |
| anthro_weight | 4 | 29 | 68.506897 | 14.0843361 | 63.70000 | 67.844000 | 13.639920 | 44.0000 | 99.50000 | 55.50000 | 0.5353132 | -0.6543667 | 2.6153955 |
| anthro_height | 5 | 29 | 171.858621 | 10.2488856 | 173.00000 | 171.732000 | 10.378200 | 153.4000 | 191.40000 | 38.00000 | -0.0674274 | -1.0191932 | 1.9031703 |
| anthro_waist | 6 | 29 | 79.131034 | 10.4464657 | 79.70000 | 78.700000 | 10.971240 | 63.0000 | 106.00000 | 43.00000 | 0.3628548 | -0.4229799 | 1.9398600 |
| baseline_KSS | 7 | 29 | 4.655172 | 1.6961271 | 4.00000 | 4.640000 | 1.482600 | 2.0000 | 8.00000 | 6.00000 | 0.2722045 | -1.1698762 | 0.3149629 |
| anthro_hips | 8 | 29 | 99.789655 | 7.3850401 | 99.30000 | 99.784000 | 8.006040 | 80.5000 | 117.00000 | 36.50000 | -0.0745193 | 0.2107570 | 1.3713675 |
| baseline_stress_state | 9 | 29 | 3.310345 | 1.9105618 | 3.00000 | 3.200000 | 1.482600 | 1.0000 | 7.00000 | 6.00000 | 0.7563787 | -0.5161601 | 0.3547824 |
| POST_EOS_stress | 10 | 28 | 2.321429 | 1.1564176 | 2.00000 | 2.208333 | 1.482600 | 1.0000 | 6.00000 | 5.00000 | 0.9064958 | 1.3505326 | 0.2185424 |
| POST_EOS_PA | 11 | 29 | 27.448276 | 7.7440586 | 28.00000 | 27.560000 | 10.378200 | 15.0000 | 39.00000 | 24.00000 | -0.0974018 | -1.3149356 | 1.4380356 |
| POST_EOS_NA | 12 | 29 | 13.172414 | 5.2445139 | 12.00000 | 12.040000 | 2.965200 | 10.0000 | 33.00000 | 23.00000 | 2.5937983 | 6.3231916 | 0.9738818 |
| POST_EOS_KSS | 13 | 29 | 5.413793 | 2.0092152 | 6.00000 | 5.560000 | 2.965200 | 1.0000 | 8.00000 | 7.00000 | -0.5287495 | -0.6071583 | 0.3731019 |
| PRE_EOS_stress | 14 | 29 | 4.827586 | 1.9285258 | 5.00000 | 4.840000 | 1.482600 | 1.0000 | 8.00000 | 7.00000 | 0.0625613 | -0.9075447 | 0.3581183 |
| PRE_EOS_PA | 15 | 29 | 29.413793 | 7.6695932 | 29.00000 | 29.400000 | 8.895600 | 12.0000 | 46.00000 | 34.00000 | -0.0848911 | -0.5489147 | 1.4242077 |
| PRE_EOS_NA | 16 | 29 | 19.827586 | 7.1617484 | 18.00000 | 19.400000 | 4.447800 | 10.0000 | 36.00000 | 26.00000 | 0.7845960 | -0.3281185 | 1.3299033 |
| baseline_PA | 17 | 29 | 33.000000 | 6.7241569 | 34.00000 | 33.080000 | 7.413000 | 20.0000 | 45.00000 | 25.00000 | -0.1170491 | -0.8424109 | 1.2486446 |
| baseline_NA | 18 | 29 | 16.068966 | 6.3241659 | 14.00000 | 15.280000 | 2.965200 | 10.0000 | 34.00000 | 24.00000 | 1.3952440 | 0.8563116 | 1.1743681 |
| demo_age | 19 | 29 | 28.724138 | 8.5602760 | 26.00000 | 27.840000 | 5.930400 | 19.0000 | 51.00000 | 32.00000 | 1.1658740 | 0.2125241 | 1.5896033 |
| demo_sex* | 20 | 29 | 1.448276 | 0.5061202 | 1.00000 | 1.440000 | 0.000000 | 1.0000 | 2.00000 | 1.00000 | 0.1973466 | -2.0274399 | 0.0939842 |
| demo_smoke* | 21 | 29 | 1.172414 | 0.3844259 | 1.00000 | 1.120000 | 0.000000 | 1.0000 | 2.00000 | 1.00000 | 1.6455195 | 0.7366627 | 0.0713861 |
| BMI | 22 | 29 | 23.075978 | 3.4956171 | 22.59251 | 23.073475 | 3.254773 | 15.7203 | 29.97277 | 14.25247 | 0.1706084 | -0.6871670 | 0.6491198 |

Summary statistics for Session 1 and women

|  | vars | n | mean | sd | median | trimmed | mad | min | max | range | skew | kurtosis | se |
| --- | --- | --- | --- | --- | --- | --- | --- | --- | --- | --- | --- | --- | --- |
| Subject* | 1 | 16 | 14.687500 | 9.7003007 | 13.00000 | 14.571429 | 12.602100 | 1.0000 | 30.00000 | 29.00000 | 0.0981032 | -1.5588708 | 2.4250752 |
| EOS_Control* | 2 | 16 | 1.437500 | 0.5123475 | 1.00000 | 1.428571 | 0.000000 | 1.0000 | 2.00000 | 1.00000 | 0.2287266 | -2.0652902 | 0.1280869 |
| Session* | 3 | 16 | 1.000000 | 0.0000000 | 1.00000 | 1.000000 | 0.000000 | 1.0000 | 1.00000 | 0.00000 | NaN | NaN | 0.0000000 |
| anthro_weight | 4 | 16 | 62.312500 | 12.2880362 | 59.30000 | 61.392857 | 6.078660 | 44.0000 | 93.50000 | 49.50000 | 1.1310219 | 0.6747077 | 3.0720090 |
| anthro_height | 5 | 16 | 164.825000 | 7.3460647 | 165.15000 | 164.592857 | 9.636900 | 153.4000 | 179.50000 | 26.10000 | 0.2220992 | -1.1179520 | 1.8365162 |
| anthro_waist | 6 | 16 | 74.193750 | 8.8959518 | 72.60000 | 73.542857 | 8.302560 | 63.0000 | 94.50000 | 31.50000 | 0.6073974 | -0.5746473 | 2.2239879 |
| baseline_KSS | 7 | 16 | 4.687500 | 1.6214705 | 4.00000 | 4.714286 | 1.482600 | 2.0000 | 7.00000 | 5.00000 | 0.1240427 | -1.4419960 | 0.4053676 |
| anthro_hips | 8 | 16 | 98.431250 | 8.4452526 | 98.00000 | 98.385714 | 7.709520 | 80.5000 | 117.00000 | 36.50000 | 0.1898927 | 0.0420456 | 2.1113132 |
| baseline_stress_state | 9 | 16 | 3.500000 | 1.8973666 | 3.00000 | 3.428571 | 0.741300 | 1.0000 | 7.00000 | 6.00000 | 0.5215562 | -0.7719907 | 0.4743416 |
| POST_EOS_stress | 10 | 15 | 2.533333 | 1.3557637 | 3.00000 | 2.384615 | 1.482600 | 1.0000 | 6.00000 | 5.00000 | 0.8173042 | 0.3878479 | 0.3500567 |
| POST_EOS_PA | 11 | 16 | 26.125000 | 7.6321688 | 25.00000 | 26.000000 | 8.154300 | 15.0000 | 39.00000 | 24.00000 | 0.2308830 | -1.1632573 | 1.9080422 |
| POST_EOS_NA | 12 | 16 | 13.937500 | 6.8553507 | 11.00000 | 12.857143 | 1.482600 | 10.0000 | 33.00000 | 23.00000 | 1.8074881 | 1.8989495 | 1.7138377 |
| POST_EOS_KSS | 13 | 16 | 5.187500 | 1.7969882 | 5.50000 | 5.285714 | 2.223900 | 1.0000 | 8.00000 | 7.00000 | -0.5207806 | -0.3925564 | 0.4492471 |
| PRE_EOS_stress | 14 | 16 | 5.312500 | 1.6620770 | 5.00000 | 5.285714 | 1.482600 | 3.0000 | 8.00000 | 5.00000 | 0.3493430 | -1.1451031 | 0.4155193 |
| PRE_EOS_PA | 15 | 16 | 27.562500 | 8.2540394 | 26.00000 | 27.714286 | 10.378200 | 12.0000 | 41.00000 | 29.00000 | -0.0182682 | -1.2275022 | 2.0635099 |
| PRE_EOS_NA | 16 | 16 | 21.062500 | 8.1850982 | 19.00000 | 20.714286 | 6.671700 | 11.0000 | 36.00000 | 25.00000 | 0.6453896 | -1.1243796 | 2.0462746 |
| baseline_PA | 17 | 16 | 31.812500 | 7.7907531 | 32.50000 | 31.714286 | 9.636900 | 20.0000 | 45.00000 | 25.00000 | -0.0048604 | -1.4172137 | 1.9476883 |
| baseline_NA | 18 | 16 | 17.250000 | 7.4966659 | 14.50000 | 16.500000 | 3.706500 | 11.0000 | 34.00000 | 23.00000 | 1.0629725 | -0.4915165 | 1.8741665 |
| demo_age | 19 | 16 | 29.437500 | 9.6399775 | 27.00000 | 28.642857 | 6.671700 | 19.0000 | 51.00000 | 32.00000 | 1.0779131 | -0.2371369 | 2.4099944 |
| demo_sex* | 20 | 16 | 1.000000 | 0.0000000 | 1.00000 | 1.000000 | 0.000000 | 1.0000 | 1.00000 | 0.00000 | NaN | NaN | 0.0000000 |
| demo_smoke* | 21 | 16 | 1.250000 | 0.4472136 | 1.00000 | 1.214286 | 0.000000 | 1.0000 | 2.00000 | 1.00000 | 1.0481569 | -0.9492187 | 0.1118034 |
| BMI | 22 | 16 | 22.879202 | 3.6269402 | 22.07019 | 22.951994 | 3.003103 | 15.7203 | 29.01902 | 13.29872 | 0.0486455 | -0.9176734 | 0.9067351 |

Summary statistics for Session 1 and men

|  | vars | n | mean | sd | median | trimmed | mad | min | max | range | skew | kurtosis | se |
| --- | --- | --- | --- | --- | --- | --- | --- | --- | --- | --- | --- | --- | --- |
| Subject* | 1 | 13 | 16.769231 | 8.1256163 | 17.00000 | 16.818182 | 8.895600 | 4.0000 | 29.00000 | 25.00000 | -0.1411315 | -1.3292775 | 2.2536405 |
| EOS_Control* | 2 | 13 | 1.615385 | 0.5063697 | 2.00000 | 1.636364 | 0.000000 | 1.0000 | 2.00000 | 1.00000 | -0.4206764 | -1.9562130 | 0.1404417 |
| Session* | 3 | 13 | 1.000000 | 0.0000000 | 1.00000 | 1.000000 | 0.000000 | 1.0000 | 1.00000 | 0.00000 | NaN | NaN | 0.0000000 |
| anthro_weight | 4 | 13 | 76.130769 | 12.6486089 | 76.40000 | 75.972727 | 9.488640 | 54.5000 | 99.50000 | 45.00000 | 0.2287546 | -0.8175812 | 3.5080929 |
| anthro_height | 5 | 13 | 180.515385 | 5.5217519 | 180.00000 | 180.209091 | 5.930400 | 173.0000 | 191.40000 | 18.40000 | 0.5476909 | -0.7434587 | 1.5314584 |
| anthro_waist | 6 | 13 | 85.207692 | 9.1217196 | 86.00000 | 84.700000 | 6.671700 | 70.0000 | 106.00000 | 36.00000 | 0.3591436 | 0.0431801 | 2.5299098 |
| baseline_KSS | 7 | 13 | 4.615385 | 1.8501559 | 5.00000 | 4.545454 | 2.965200 | 2.0000 | 8.00000 | 6.00000 | 0.3781776 | -1.2264567 | 0.5131409 |
| anthro_hips | 8 | 13 | 101.461539 | 5.7110330 | 101.10000 | 101.500000 | 6.226920 | 92.0000 | 110.50000 | 18.50000 | -0.1339639 | -1.3429123 | 1.5839556 |
| baseline_stress_state | 9 | 13 | 3.076923 | 1.9774368 | 2.00000 | 2.909091 | 1.482600 | 1.0000 | 7.00000 | 6.00000 | 0.9765815 | -0.3883119 | 0.5484423 |
| POST_EOS_stress | 10 | 13 | 2.076923 | 0.8623165 | 2.00000 | 2.090909 | 1.482600 | 1.0000 | 3.00000 | 2.00000 | -0.1277740 | -1.7464627 | 0.2391636 |
| POST_EOS_PA | 11 | 13 | 29.076923 | 7.8683071 | 31.00000 | 29.454545 | 8.895600 | 16.0000 | 38.00000 | 22.00000 | -0.4949645 | -1.3141604 | 2.1822757 |
| POST_EOS_NA | 12 | 13 | 12.230769 | 1.9215378 | 12.00000 | 12.090909 | 1.482600 | 10.0000 | 16.00000 | 6.00000 | 0.4788448 | -1.0249630 | 0.5329387 |
| POST_EOS_KSS | 13 | 13 | 5.692308 | 2.2870875 | 6.00000 | 5.909091 | 2.965200 | 1.0000 | 8.00000 | 7.00000 | -0.6152217 | -0.9913341 | 0.6343239 |
| PRE_EOS_stress | 14 | 13 | 4.230769 | 2.1273554 | 4.00000 | 4.181818 | 2.965200 | 1.0000 | 8.00000 | 7.00000 | 0.2016836 | -1.3237739 | 0.5900222 |
| PRE_EOS_PA | 15 | 13 | 31.692308 | 6.4728229 | 33.00000 | 31.454545 | 5.930400 | 20.0000 | 46.00000 | 26.00000 | 0.3518544 | -0.1701737 | 1.7952381 |
| PRE_EOS_NA | 16 | 13 | 18.307692 | 5.6033415 | 18.00000 | 18.000000 | 4.447800 | 10.0000 | 30.00000 | 20.00000 | 0.3086158 | -0.5857817 | 1.5540873 |
| baseline_PA | 17 | 13 | 34.461539 | 5.0434014 | 34.00000 | 34.181818 | 4.447800 | 28.0000 | 44.00000 | 16.00000 | 0.5373286 | -0.9194658 | 1.3987879 |
| baseline_NA | 18 | 13 | 14.615385 | 4.3500663 | 14.00000 | 14.090909 | 2.965200 | 10.0000 | 25.00000 | 15.00000 | 1.0146107 | 0.1281019 | 1.2064913 |
| demo_age | 19 | 13 | 27.846154 | 7.3012117 | 26.00000 | 27.181818 | 5.930400 | 20.0000 | 43.00000 | 23.00000 | 0.8821185 | -0.7568277 | 2.0249918 |
| demo_sex* | 20 | 13 | 2.000000 | 0.0000000 | 2.00000 | 2.000000 | 0.000000 | 2.0000 | 2.00000 | 0.00000 | NaN | NaN | 0.0000000 |
| demo_smoke* | 21 | 13 | 1.076923 | 0.2773501 | 1.00000 | 1.000000 | 0.000000 | 1.0000 | 2.00000 | 1.00000 | 2.8161702 | 6.4437870 | 0.0769231 |
| BMI | 22 | 13 | 23.318165 | 3.4573474 | 22.66271 | 23.228088 | 2.044508 | 17.6544 | 29.97277 | 12.31837 | 0.3469619 | -0.8456720 | 0.9588956 |

Summary statistics for Session 1 and EOS intervention first

|  | vars | n | mean | sd | median | trimmed | mad | min | max | range | skew | kurtosis | se |
| --- | --- | --- | --- | --- | --- | --- | --- | --- | --- | --- | --- | --- | --- |
| Subject* | 1 | 14 | 16.071429 | 8.1661994 | 17.00000 | 16.333333 | 11.119500 | 2.0000 | 27.00000 | 25.00000 | -0.1729748 | -1.5242933 | 2.1825086 |
| EOS_Control* | 2 | 14 | 1.000000 | 0.0000000 | 1.00000 | 1.000000 | 0.000000 | 1.0000 | 1.00000 | 0.00000 | NaN | NaN | 0.0000000 |
| Session* | 3 | 14 | 1.000000 | 0.0000000 | 1.00000 | 1.000000 | 0.000000 | 1.0000 | 1.00000 | 0.00000 | NaN | NaN | 0.0000000 |
| anthro_weight | 4 | 14 | 65.135714 | 11.9209323 | 61.45000 | 63.900000 | 11.934930 | 51.6000 | 93.50000 | 41.90000 | 0.8187947 | -0.2710572 | 3.1860032 |
| anthro_height | 5 | 14 | 169.371429 | 10.0051086 | 170.40000 | 169.708333 | 13.788180 | 153.4000 | 181.30000 | 27.90000 | -0.2330064 | -1.6395737 | 2.6739777 |
| anthro_waist | 6 | 14 | 77.007143 | 10.7782162 | 76.00000 | 76.716667 | 13.491660 | 63.0000 | 94.50000 | 31.50000 | 0.1355636 | -1.6849667 | 2.8805995 |
| baseline_KSS | 7 | 14 | 4.500000 | 1.6052798 | 4.50000 | 4.500000 | 2.223900 | 2.0000 | 7.00000 | 5.00000 | 0.1554040 | -1.4093896 | 0.4290291 |
| anthro_hips | 8 | 14 | 98.900000 | 7.0333273 | 98.30000 | 98.025000 | 7.783650 | 91.3000 | 117.00000 | 25.70000 | 1.0294670 | 0.4839324 | 1.8797358 |
| baseline_stress_state | 9 | 14 | 3.428571 | 2.0272864 | 3.00000 | 3.333333 | 1.482600 | 1.0000 | 7.00000 | 6.00000 | 0.5332680 | -0.9786104 | 0.5418151 |
| POST_EOS_stress | 10 | 14 | 1.928571 | 0.9972490 | 2.00000 | 1.833333 | 1.482600 | 1.0000 | 4.00000 | 3.00000 | 0.5600026 | -1.0626664 | 0.2665260 |
| POST_EOS_PA | 11 | 14 | 30.071429 | 8.0044630 | 31.00000 | 30.500000 | 8.895600 | 16.0000 | 39.00000 | 23.00000 | -0.3974218 | -1.4387759 | 2.1392827 |
| POST_EOS_NA | 12 | 14 | 12.071429 | 2.4950500 | 11.00000 | 11.750000 | 1.482600 | 10.0000 | 18.00000 | 8.00000 | 1.0561034 | -0.0788173 | 0.6668302 |
| POST_EOS_KSS | 13 | 14 | 4.928571 | 2.2348390 | 5.50000 | 5.000000 | 2.223900 | 1.0000 | 8.00000 | 7.00000 | -0.3780817 | -1.0124010 | 0.5972859 |
| PRE_EOS_stress | 14 | 14 | 4.357143 | 1.6919330 | 4.50000 | 4.416667 | 2.223900 | 1.0000 | 7.00000 | 6.00000 | -0.3467200 | -0.9439836 | 0.4521881 |
| PRE_EOS_PA | 15 | 14 | 30.857143 | 6.5967025 | 31.00000 | 31.000000 | 7.413000 | 19.0000 | 41.00000 | 22.00000 | -0.1103557 | -1.3740204 | 1.7630429 |
| PRE_EOS_NA | 16 | 14 | 19.571429 | 6.9250302 | 18.50000 | 19.166667 | 5.930400 | 10.0000 | 34.00000 | 24.00000 | 0.5365585 | -0.7584513 | 1.8507922 |
| baseline_PA | 17 | 14 | 34.785714 | 5.6592820 | 35.00000 | 34.916667 | 5.930400 | 23.0000 | 45.00000 | 22.00000 | -0.1320983 | -0.5556120 | 1.5125067 |
| baseline_NA | 18 | 14 | 17.285714 | 7.3842720 | 14.50000 | 16.500000 | 2.965200 | 10.0000 | 34.00000 | 24.00000 | 1.0977538 | -0.2598816 | 1.9735297 |
| demo_age | 19 | 14 | 31.785714 | 10.0779926 | 28.50000 | 31.250000 | 9.636900 | 19.0000 | 51.00000 | 32.00000 | 0.5221124 | -1.1834358 | 2.6934568 |
| demo_sex* | 20 | 14 | 1.357143 | 0.4972452 | 1.00000 | 1.333333 | 0.000000 | 1.0000 | 2.00000 | 1.00000 | 0.5335519 | -1.8311791 | 0.1328944 |
| demo_smoke* | 21 | 14 | 1.214286 | 0.4258153 | 1.00000 | 1.166667 | 0.000000 | 1.0000 | 2.00000 | 1.00000 | 1.2461089 | -0.4655226 | 0.1138039 |
| BMI | 22 | 14 | 22.658824 | 3.2118667 | 22.13958 | 22.545843 | 2.662984 | 17.6544 | 29.01902 | 11.36462 | 0.4155584 | -0.9127071 | 0.8584075 |

Summary statistics for Session 1 and Control intervention first

|  | vars | n | mean | sd | median | trimmed | mad | min | max | range | skew | kurtosis | se |
| --- | --- | --- | --- | --- | --- | --- | --- | --- | --- | --- | --- | --- | --- |
| Subject* | 1 | 15 | 15.200000 | 9.8648003 | 16.00000 | 15.153846 | 13.343400 | 1.0000 | 30.00000 | 29.00000 | 0.0763511 | -1.5040840 | 2.5470805 |
| EOS_Control* | 2 | 15 | 2.000000 | 0.0000000 | 2.00000 | 2.000000 | 0.000000 | 2.0000 | 2.00000 | 0.00000 | NaN | NaN | 0.0000000 |
| Session* | 3 | 15 | 1.000000 | 0.0000000 | 1.00000 | 1.000000 | 0.000000 | 1.0000 | 1.00000 | 0.00000 | NaN | NaN | 0.0000000 |
| anthro_weight | 4 | 15 | 71.653333 | 15.5822640 | 68.80000 | 71.638462 | 15.270780 | 44.0000 | 99.50000 | 55.50000 | 0.1871161 | -1.0751302 | 4.0233233 |
| anthro_height | 5 | 15 | 174.180000 | 10.2551172 | 173.00000 | 174.169231 | 10.378200 | 157.1000 | 191.40000 | 34.30000 | 0.0399412 | -1.1461322 | 2.6478599 |
| anthro_waist | 6 | 15 | 81.113333 | 10.0828615 | 79.80000 | 80.476923 | 9.340380 | 64.5000 | 106.00000 | 41.50000 | 0.7020927 | 0.2327847 | 2.6033836 |
| baseline_KSS | 7 | 15 | 4.800000 | 1.8205180 | 4.00000 | 4.769231 | 1.482600 | 2.0000 | 8.00000 | 6.00000 | 0.2757836 | -1.3503528 | 0.4700557 |
| anthro_hips | 8 | 15 | 100.620000 | 7.8494040 | 100.00000 | 101.407692 | 8.006040 | 80.5000 | 110.50000 | 30.00000 | -0.8706384 | 0.3329784 | 2.0267074 |
| baseline_stress_state | 9 | 15 | 3.200000 | 1.8593394 | 3.00000 | 3.076923 | 1.482600 | 1.0000 | 7.00000 | 6.00000 | 0.9110141 | -0.3053626 | 0.4800794 |
| POST_EOS_stress | 10 | 14 | 2.714286 | 1.2043876 | 3.00000 | 2.583333 | 0.000000 | 1.0000 | 6.00000 | 5.00000 | 1.0012864 | 1.5713232 | 0.3218861 |
| POST_EOS_PA | 11 | 15 | 25.000000 | 6.8660656 | 26.00000 | 24.846154 | 8.895600 | 15.0000 | 37.00000 | 22.00000 | -0.0407803 | -1.3592874 | 1.7728105 |
| POST_EOS_NA | 12 | 15 | 14.200000 | 6.8473144 | 12.00000 | 13.076923 | 2.965200 | 10.0000 | 33.00000 | 23.00000 | 1.8413087 | 1.9353292 | 1.7679690 |
| POST_EOS_KSS | 13 | 15 | 5.866667 | 1.7265435 | 6.00000 | 5.923077 | 1.482600 | 3.0000 | 8.00000 | 5.00000 | -0.3532474 | -1.2969680 | 0.4457916 |
| PRE_EOS_stress | 14 | 15 | 5.266667 | 2.0862361 | 5.00000 | 5.307692 | 2.965200 | 2.0000 | 8.00000 | 6.00000 | 0.0658502 | -1.5135962 | 0.5386638 |
| PRE_EOS_PA | 15 | 15 | 28.066667 | 8.5562564 | 29.00000 | 27.923077 | 8.895600 | 12.0000 | 46.00000 | 34.00000 | 0.1085579 | -0.6048408 | 2.2092159 |
| PRE_EOS_NA | 16 | 15 | 20.066667 | 7.6107693 | 18.00000 | 19.615385 | 4.447800 | 10.0000 | 36.00000 | 26.00000 | 0.8711773 | -0.4528216 | 1.9650922 |
| baseline_PA | 17 | 15 | 31.333333 | 7.3840239 | 31.00000 | 31.230769 | 7.413000 | 20.0000 | 44.00000 | 24.00000 | 0.1455704 | -1.2032978 | 1.9065468 |
| baseline_NA | 18 | 15 | 14.933333 | 5.1473525 | 14.00000 | 14.384615 | 2.965200 | 10.0000 | 27.00000 | 17.00000 | 1.3292196 | 0.5937657 | 1.3290407 |
| demo_age | 19 | 15 | 25.866667 | 5.8415588 | 24.00000 | 24.923077 | 2.965200 | 20.0000 | 44.00000 | 24.00000 | 1.8873065 | 3.3491029 | 1.5082840 |
| demo_sex* | 20 | 15 | 1.533333 | 0.5163978 | 2.00000 | 1.538461 | 0.000000 | 1.0000 | 2.00000 | 1.00000 | -0.1204928 | -2.1133333 | 0.1333333 |
| demo_smoke* | 21 | 15 | 1.133333 | 0.3518658 | 1.00000 | 1.076923 | 0.000000 | 1.0000 | 2.00000 | 1.00000 | 1.9451862 | 1.9251282 | 0.0908514 |
| BMI | 22 | 15 | 23.465322 | 3.8109079 | 22.59251 | 23.560520 | 2.953039 | 15.7203 | 29.97277 | 14.25247 | -0.0613789 | -0.7937342 | 0.9839722 |

Summary statistics for Session 2 and both sexes

|  | vars | n | mean | sd | median | trimmed | mad | min | max | range | skew | kurtosis | se |
| --- | --- | --- | --- | --- | --- | --- | --- | --- | --- | --- | --- | --- | --- |
| Subject* | 1 | 29 | 15.620690 | 8.9339393 | 16.0000 | 15.640000 | 11.860800 | 1.00000 | 30.000 | 29.00000 | -0.0370590 | -1.3617833 | 1.6589909 |
| EOS_Control* | 2 | 29 | 1.482759 | 0.5085476 | 1.0000 | 1.480000 | 0.000000 | 1.00000 | 2.000 | 1.00000 | 0.0654682 | -2.0633373 | 0.0944349 |
| Session* | 3 | 29 | 2.000000 | 0.0000000 | 2.0000 | 2.000000 | 0.000000 | 2.00000 | 2.000 | 0.00000 | NaN | NaN | 0.0000000 |
| anthro_weight | 4 | 28 | 68.625000 | 13.9828897 | 66.0000 | 67.908333 | 13.862310 | 45.90000 | 100.000 | 54.10000 | 0.5440015 | -0.7285137 | 2.6425178 |
| anthro_height | 5 | 28 | 171.735714 | 10.4280325 | 172.9500 | 171.600000 | 11.045370 | 153.00000 | 191.200 | 38.20000 | -0.0739475 | -1.0004143 | 1.9707129 |
| anthro_waist | 6 | 28 | 78.175000 | 10.1315285 | 78.0000 | 77.645833 | 10.600590 | 62.20000 | 102.100 | 39.90000 | 0.3985197 | -0.6406546 | 1.9146789 |
| baseline_KSS | 7 | 28 | 5.035714 | 1.5270922 | 5.0000 | 4.958333 | 1.482600 | 3.00000 | 8.000 | 5.00000 | 0.3033948 | -1.0416822 | 0.2885933 |
| anthro_hips | 8 | 28 | 99.910714 | 8.9134247 | 98.5500 | 99.395833 | 7.709520 | 82.00000 | 123.300 | 41.30000 | 0.6324249 | 0.2599978 | 1.6844789 |
| baseline_stress_state | 9 | 28 | 3.535714 | 1.7739294 | 3.0000 | 3.500000 | 1.482600 | 1.00000 | 7.000 | 6.00000 | 0.2696393 | -1.2757656 | 0.3352411 |
| POST_EOS_stress | 10 | 29 | 2.482759 | 1.2427127 | 2.0000 | 2.360000 | 1.482600 | 1.00000 | 6.000 | 5.00000 | 1.1159986 | 0.8172805 | 0.2307660 |
| POST_EOS_PA | 11 | 29 | 25.724138 | 7.7731063 | 27.0000 | 25.640000 | 8.895600 | 13.00000 | 42.000 | 29.00000 | 0.0890382 | -0.9810542 | 1.4434296 |
| POST_EOS_NA | 12 | 29 | 13.310345 | 4.8040418 | 12.0000 | 12.560000 | 2.965200 | 9.00000 | 32.000 | 23.00000 | 2.2170697 | 5.4171576 | 0.8920882 |
| POST_EOS_KSS | 13 | 29 | 5.275862 | 1.9620789 | 5.0000 | 5.280000 | 1.482600 | 1.00000 | 9.000 | 8.00000 | 0.0099662 | -0.8543271 | 0.3643489 |
| PRE_EOS_stress | 14 | 28 | 4.214286 | 1.6856067 | 4.0000 | 4.250000 | 1.482600 | 1.00000 | 7.000 | 6.00000 | -0.1460994 | -0.7632230 | 0.3185497 |
| PRE_EOS_PA | 15 | 28 | 29.000000 | 8.0046283 | 29.5000 | 29.041667 | 8.895600 | 13.00000 | 46.000 | 33.00000 | -0.0108628 | -0.8620772 | 1.5127326 |
| PRE_EOS_NA | 16 | 28 | 15.964286 | 6.0521324 | 14.0000 | 15.250000 | 4.447800 | 10.00000 | 31.000 | 21.00000 | 1.1217878 | 0.1872713 | 1.1437455 |
| baseline_PA | 17 | 28 | 30.000000 | 6.6777685 | 29.0000 | 29.958333 | 7.413000 | 17.00000 | 45.000 | 28.00000 | 0.0834751 | -0.5998411 | 1.2619796 |
| baseline_NA | 18 | 28 | 15.785714 | 6.3907175 | 14.0000 | 14.791667 | 4.447800 | 10.00000 | 37.000 | 27.00000 | 1.6314907 | 2.5103320 | 1.2077321 |
| demo_age | 19 | 28 | 29.000000 | 8.8023566 | 26.0000 | 28.083333 | 5.930400 | 19.00000 | 52.000 | 33.00000 | 1.1455473 | 0.1785190 | 1.6634890 |
| demo_sex* | 20 | 29 | 1.517241 | 0.5744991 | 1.0000 | 1.480000 | 0.000000 | 1.00000 | 3.000 | 2.00000 | 0.4813523 | -0.9064946 | 0.1066818 |
| demo_smoke* | 21 | 29 | 1.241379 | 0.5109635 | 1.0000 | 1.160000 | 0.000000 | 1.00000 | 3.000 | 2.00000 | 1.8865275 | 2.7159673 | 0.0948835 |
| BMI | 22 | 28 | 23.156940 | 3.4866165 | 22.4476 | 23.170400 | 3.144815 | 16.43841 | 29.502 | 13.06359 | 0.1969603 | -0.9381591 | 0.6589086 |

Summary statistics for Session 2 and women

|  | vars | n | mean | sd | median | trimmed | mad | min | max | range | skew | kurtosis | se |
| --- | --- | --- | --- | --- | --- | --- | --- | --- | --- | --- | --- | --- | --- |
| Subject* | 1 | 15 | 15.066667 | 9.9172769 | 15.00000 | 15.000000 | 13.343400 | 1.00000 | 30.00000 | 29.00000 | 0.0033907 | -1.6088902 | 2.5606299 |
| EOS_Control* | 2 | 15 | 1.533333 | 0.5163978 | 2.00000 | 1.538461 | 0.000000 | 1.00000 | 2.00000 | 1.00000 | -0.1204928 | -2.1133333 | 0.1333333 |
| Session* | 3 | 15 | 2.000000 | 0.0000000 | 2.00000 | 2.000000 | 0.000000 | 2.00000 | 2.00000 | 0.00000 | NaN | NaN | 0.0000000 |
| anthro_weight | 4 | 15 | 62.340000 | 12.0526701 | 58.80000 | 61.353846 | 5.930400 | 45.90000 | 91.60000 | 45.70000 | 1.1153070 | 0.3936561 | 3.1119860 |
| anthro_height | 5 | 15 | 164.333333 | 7.4805144 | 163.50000 | 164.076923 | 8.006040 | 153.00000 | 179.00000 | 26.00000 | 0.2603372 | -1.1551919 | 1.9314605 |
| anthro_waist | 6 | 15 | 73.626667 | 8.7153778 | 72.60000 | 73.030769 | 9.340380 | 62.20000 | 92.80000 | 30.60000 | 0.5893198 | -0.7332478 | 2.2503009 |
| baseline_KSS | 7 | 15 | 5.000000 | 1.4142136 | 5.00000 | 4.923077 | 1.482600 | 3.00000 | 8.00000 | 5.00000 | 0.4242641 | -0.7333333 | 0.3651484 |
| anthro_hips | 8 | 15 | 98.980000 | 10.7096885 | 95.90000 | 98.415385 | 7.116480 | 82.00000 | 123.30000 | 41.30000 | 0.7895503 | -0.0914179 | 2.7652297 |
| baseline_stress_state | 9 | 15 | 4.133333 | 1.6846647 | 5.00000 | 4.230769 | 1.482600 | 1.00000 | 6.00000 | 5.00000 | -0.3615365 | -1.3977575 | 0.4349786 |
| POST_EOS_stress | 10 | 15 | 2.533333 | 1.0600988 | 2.00000 | 2.461539 | 1.482600 | 1.00000 | 5.00000 | 4.00000 | 0.5904293 | -0.1798372 | 0.2737163 |
| POST_EOS_PA | 11 | 15 | 26.400000 | 7.6232726 | 27.00000 | 26.692308 | 8.895600 | 13.00000 | 36.00000 | 23.00000 | -0.4296681 | -1.1254484 | 1.9683205 |
| POST_EOS_NA | 12 | 15 | 13.933333 | 6.0764965 | 12.00000 | 12.923077 | 2.965200 | 9.00000 | 32.00000 | 23.00000 | 1.7561646 | 2.4343529 | 1.5689446 |
| POST_EOS_KSS | 13 | 15 | 5.200000 | 1.6987390 | 5.00000 | 5.076923 | 1.482600 | 3.00000 | 9.00000 | 6.00000 | 0.6886879 | -0.3839767 | 0.4386125 |
| PRE_EOS_stress | 14 | 15 | 4.466667 | 1.5055453 | 4.00000 | 4.461538 | 1.482600 | 2.00000 | 7.00000 | 5.00000 | 0.0595619 | -0.8313264 | 0.3887301 |
| PRE_EOS_PA | 15 | 15 | 28.666667 | 8.4487249 | 30.00000 | 29.000000 | 8.895600 | 13.00000 | 40.00000 | 27.00000 | -0.3980813 | -1.2371376 | 2.1814514 |
| PRE_EOS_NA | 16 | 15 | 16.133333 | 6.8854574 | 14.00000 | 15.461538 | 5.930400 | 10.00000 | 31.00000 | 21.00000 | 0.8811258 | -0.7837678 | 1.7778175 |
| baseline_PA | 17 | 15 | 31.000000 | 6.5683222 | 33.00000 | 31.384615 | 5.930400 | 17.00000 | 40.00000 | 23.00000 | -0.5166277 | -0.8456705 | 1.6959335 |
| baseline_NA | 18 | 15 | 16.600000 | 7.9803330 | 13.00000 | 15.538462 | 2.965200 | 10.00000 | 37.00000 | 27.00000 | 1.3375592 | 0.5434515 | 2.0605131 |
| demo_age | 19 | 15 | 29.933333 | 9.9747300 | 27.00000 | 29.076923 | 7.413000 | 19.00000 | 52.00000 | 33.00000 | 1.0145782 | -0.3667944 | 2.5754642 |
| demo_sex* | 20 | 15 | 1.000000 | 0.0000000 | 1.00000 | 1.000000 | 0.000000 | 1.00000 | 1.00000 | 0.00000 | NaN | NaN | 0.0000000 |
| demo_smoke* | 21 | 15 | 1.266667 | 0.4577377 | 1.00000 | 1.230769 | 0.000000 | 1.00000 | 2.00000 | 1.00000 | 0.9515396 | -1.1587879 | 0.1181874 |
| BMI | 22 | 15 | 23.021830 | 3.5203216 | 22.24821 | 23.100052 | 2.835149 | 16.43841 | 28.58837 | 12.14996 | 0.1055707 | -1.0866892 | 0.9089431 |

Summary statistics for Session 2 and men

|  | vars | n | mean | sd | median | trimmed | mad | min | max | range | skew | kurtosis | se |
| --- | --- | --- | --- | --- | --- | --- | --- | --- | --- | --- | --- | --- | --- |
| Subject* | 1 | 13 | 16.769231 | 8.1256163 | 17.0000 | 16.818182 | 8.895600 | 4.00000 | 29.000 | 25.00000 | -0.1411315 | -1.3292775 | 2.2536405 |
| EOS_Control* | 2 | 13 | 1.384615 | 0.5063697 | 1.0000 | 1.363636 | 0.000000 | 1.00000 | 2.000 | 1.00000 | 0.4206764 | -1.9562130 | 0.1404417 |
| Session* | 3 | 13 | 2.000000 | 0.0000000 | 2.0000 | 2.000000 | 0.000000 | 2.00000 | 2.000 | 0.00000 | NaN | NaN | 0.0000000 |
| anthro_weight | 4 | 13 | 75.876923 | 12.8099671 | 75.0000 | 75.627273 | 10.971240 | 54.50000 | 100.000 | 45.50000 | 0.2424172 | -0.8898162 | 3.5528456 |
| anthro_height | 5 | 13 | 180.276923 | 5.6447843 | 180.2000 | 179.900000 | 5.040840 | 173.50000 | 191.200 | 17.70000 | 0.6847860 | -0.7294187 | 1.5655815 |
| anthro_waist | 6 | 13 | 83.423077 | 9.3082539 | 82.7000 | 82.990909 | 9.340380 | 69.50000 | 102.100 | 32.60000 | 0.3267311 | -0.8035994 | 2.5816451 |
| baseline_KSS | 7 | 13 | 5.076923 | 1.7059474 | 5.0000 | 5.000000 | 1.482600 | 3.00000 | 8.000 | 5.00000 | 0.1694240 | -1.5250849 | 0.4731447 |
| anthro_hips | 8 | 13 | 100.984615 | 6.5279969 | 100.2000 | 100.672727 | 8.895600 | 92.10000 | 113.300 | 21.20000 | 0.2278922 | -1.2665711 | 1.8105406 |
| baseline_stress_state | 9 | 13 | 2.846154 | 1.6756170 | 2.0000 | 2.636364 | 1.482600 | 1.00000 | 7.000 | 6.00000 | 1.1052585 | 0.3841135 | 0.4647325 |
| POST_EOS_stress | 10 | 13 | 2.461539 | 1.5063966 | 2.0000 | 2.272727 | 1.482600 | 1.00000 | 6.000 | 5.00000 | 1.1480478 | 0.1731751 | 0.4177992 |
| POST_EOS_PA | 11 | 13 | 24.923077 | 8.4799915 | 23.0000 | 24.272727 | 8.895600 | 15.00000 | 42.000 | 27.00000 | 0.5669140 | -0.9866868 | 2.3519265 |
| POST_EOS_NA | 12 | 13 | 12.846154 | 3.0234129 | 12.0000 | 12.454546 | 1.482600 | 10.00000 | 20.000 | 10.00000 | 1.2209748 | 0.2187261 | 0.8385439 |
| POST_EOS_KSS | 13 | 13 | 5.230769 | 2.3149459 | 5.0000 | 5.363636 | 2.965200 | 1.00000 | 8.000 | 7.00000 | -0.2212406 | -1.4107035 | 0.6420505 |
| PRE_EOS_stress | 14 | 13 | 3.923077 | 1.8912755 | 4.0000 | 3.909091 | 1.482600 | 1.00000 | 7.000 | 6.00000 | -0.1033467 | -1.2523629 | 0.5245454 |
| PRE_EOS_PA | 15 | 13 | 29.384615 | 7.7839414 | 26.0000 | 28.727273 | 5.930400 | 20.00000 | 46.000 | 26.00000 | 0.5880340 | -0.9178686 | 2.1588769 |
| PRE_EOS_NA | 16 | 13 | 15.769231 | 5.1986191 | 15.0000 | 15.000000 | 2.965200 | 10.00000 | 30.000 | 20.00000 | 1.3696969 | 1.6191198 | 1.4418375 |
| baseline_PA | 17 | 13 | 28.846154 | 6.8780588 | 28.0000 | 28.272727 | 4.447800 | 19.00000 | 45.000 | 26.00000 | 0.7210531 | 0.0298905 | 1.9076303 |
| baseline_NA | 18 | 13 | 14.846154 | 3.9758889 | 15.0000 | 14.727273 | 5.930400 | 10.00000 | 21.000 | 11.00000 | 0.1047649 | -1.6702308 | 1.1027132 |
| demo_age | 19 | 13 | 27.923077 | 7.4773161 | 26.0000 | 27.181818 | 5.930400 | 20.00000 | 44.000 | 24.00000 | 0.9280689 | -0.6216235 | 2.0738344 |
| demo_sex* | 20 | 13 | 2.000000 | 0.0000000 | 2.0000 | 2.000000 | 0.000000 | 2.00000 | 2.000 | 0.00000 | NaN | NaN | 0.0000000 |
| demo_smoke* | 21 | 13 | 1.076923 | 0.2773501 | 1.0000 | 1.000000 | 0.000000 | 1.00000 | 2.000 | 1.00000 | 2.8161702 | 6.4437870 | 0.0769231 |
| BMI | 22 | 13 | 23.312836 | 3.5839723 | 22.5183 | 23.260928 | 3.054051 | 17.69466 | 29.502 | 11.80734 | 0.2721752 | -1.1265950 | 0.9940151 |

Summary statistics for Session 2 and EOS intervention first

|  | vars | n | mean | sd | median | trimmed | mad | min | max | range | skew | kurtosis | se |
| --- | --- | --- | --- | --- | --- | --- | --- | --- | --- | --- | --- | --- | --- |
| Subject* | 1 | 15 | 15.200000 | 9.8648003 | 16.0000 | 15.153846 | 13.34340 | 1.00000 | 30.000 | 29.00000 | 0.0763511 | -1.5040840 | 2.5470805 |
| EOS_Control* | 2 | 15 | 1.000000 | 0.0000000 | 1.0000 | 1.000000 | 0.00000 | 1.00000 | 1.000 | 0.00000 | NaN | NaN | 0.0000000 |
| Session* | 3 | 15 | 2.000000 | 0.0000000 | 2.0000 | 2.000000 | 0.00000 | 2.00000 | 2.000 | 0.00000 | NaN | NaN | 0.0000000 |
| anthro_weight | 4 | 15 | 71.300000 | 15.6290023 | 67.9000 | 71.046154 | 16.75338 | 45.90000 | 100.000 | 54.10000 | 0.2706703 | -1.2002074 | 4.0353911 |
| anthro_height | 5 | 15 | 174.013333 | 10.4788813 | 173.5000 | 174.053846 | 11.41602 | 156.30000 | 191.200 | 34.90000 | 0.0331782 | -1.1372929 | 2.7056355 |
| anthro_waist | 6 | 15 | 79.526667 | 10.0290625 | 77.5000 | 78.830769 | 7.26474 | 66.00000 | 102.100 | 36.10000 | 0.7264301 | -0.3430770 | 2.5894928 |
| baseline_KSS | 7 | 15 | 5.133333 | 1.7674302 | 5.0000 | 5.076923 | 1.48260 | 3.00000 | 8.000 | 5.00000 | 0.3196348 | -1.4340502 | 0.4563485 |
| anthro_hips | 8 | 15 | 100.333333 | 9.1801546 | 100.2000 | 100.415385 | 9.48864 | 82.00000 | 117.600 | 35.60000 | 0.0273617 | -0.6653132 | 2.3703057 |
| baseline_stress_state | 9 | 15 | 3.666667 | 1.4960265 | 4.0000 | 3.692308 | 1.48260 | 1.00000 | 6.000 | 5.00000 | -0.1814104 | -1.4058548 | 0.3862724 |
| POST_EOS_stress | 10 | 15 | 2.200000 | 0.8618916 | 2.0000 | 2.153846 | 1.48260 | 1.00000 | 4.000 | 3.00000 | 0.2748873 | -0.7809862 | 0.2225395 |
| POST_EOS_PA | 11 | 15 | 24.666667 | 8.9814624 | 22.0000 | 24.230769 | 8.89560 | 13.00000 | 42.000 | 29.00000 | 0.4231755 | -1.1210118 | 2.3190036 |
| POST_EOS_NA | 12 | 15 | 14.000000 | 6.0710084 | 12.0000 | 13.000000 | 2.96520 | 9.00000 | 32.000 | 23.00000 | 1.7554521 | 2.3749534 | 1.5675276 |
| POST_EOS_KSS | 13 | 15 | 5.200000 | 2.3052735 | 5.0000 | 5.230769 | 2.96520 | 1.00000 | 9.000 | 8.00000 | 0.0666075 | -1.1603330 | 0.5952190 |
| PRE_EOS_stress | 14 | 15 | 4.066667 | 1.6242214 | 4.0000 | 4.076923 | 1.48260 | 1.00000 | 7.000 | 6.00000 | -0.0994373 | -0.7994820 | 0.4193722 |
| PRE_EOS_PA | 15 | 15 | 27.200000 | 9.2133444 | 24.0000 | 26.846154 | 8.89560 | 13.00000 | 46.000 | 33.00000 | 0.3624380 | -0.9790278 | 2.3788753 |
| PRE_EOS_NA | 16 | 15 | 15.400000 | 5.0398413 | 14.0000 | 15.000000 | 4.44780 | 10.00000 | 26.000 | 16.00000 | 0.8459209 | -0.4706710 | 1.3012814 |
| baseline_PA | 17 | 15 | 28.400000 | 7.3075304 | 27.0000 | 28.000000 | 5.93040 | 17.00000 | 45.000 | 28.00000 | 0.5077313 | -0.4238932 | 1.8867962 |
| baseline_NA | 18 | 15 | 15.666667 | 5.6904264 | 14.0000 | 15.000000 | 4.44780 | 10.00000 | 30.000 | 20.00000 | 1.1154040 | 0.2428449 | 1.4692618 |
| demo_age | 19 | 15 | 25.866667 | 5.8415588 | 24.0000 | 24.923077 | 2.96520 | 20.00000 | 44.000 | 24.00000 | 1.8873065 | 3.3491029 | 1.5082840 |
| demo_sex* | 20 | 15 | 1.533333 | 0.5163978 | 2.0000 | 1.538461 | 0.00000 | 1.00000 | 2.000 | 1.00000 | -0.1204928 | -2.1133333 | 0.1333333 |
| demo_smoke* | 21 | 15 | 1.133333 | 0.3518658 | 1.0000 | 1.076923 | 0.00000 | 1.00000 | 2.000 | 1.00000 | 1.9451862 | 1.9251282 | 0.0908514 |
| BMI | 22 | 15 | 23.400246 | 3.8524582 | 22.5183 | 23.466406 | 3.10686 | 16.43841 | 29.502 | 13.06359 | 0.0906873 | -1.1659341 | 0.9947004 |

Summary statistics for Session 2 and Control intervention first

|  | vars | n | mean | sd | median | trimmed | mad | min | max | range | skew | kurtosis | se |
| --- | --- | --- | --- | --- | --- | --- | --- | --- | --- | --- | --- | --- | --- |
| Subject* | 1 | 14 | 16.071429 | 8.1661994 | 17.00000 | 16.333333 | 11.119500 | 2.00000 | 27.00000 | 25.00000 | -0.1729748 | -1.5242933 | 2.1825086 |
| EOS_Control* | 2 | 14 | 2.000000 | 0.0000000 | 2.00000 | 2.000000 | 0.000000 | 2.00000 | 2.00000 | 0.00000 | NaN | NaN | 0.0000000 |
| Session* | 3 | 14 | 2.000000 | 0.0000000 | 2.00000 | 2.000000 | 0.000000 | 2.00000 | 2.00000 | 0.00000 | NaN | NaN | 0.0000000 |
| anthro_weight | 4 | 13 | 65.538461 | 11.6482143 | 62.80000 | 64.418182 | 10.378200 | 51.80000 | 91.60000 | 39.80000 | 0.6751773 | -0.5589692 | 3.2306334 |
| anthro_height | 5 | 13 | 169.107692 | 10.1291215 | 172.00000 | 169.445455 | 12.750360 | 153.00000 | 181.50000 | 28.50000 | -0.2708511 | -1.6729211 | 2.8093128 |
| anthro_waist | 6 | 13 | 76.615385 | 10.4255972 | 79.00000 | 76.454545 | 14.084700 | 62.20000 | 92.80000 | 30.60000 | 0.0690946 | -1.6639585 | 2.8915404 |
| baseline_KSS | 7 | 13 | 4.923077 | 1.2557560 | 5.00000 | 4.909091 | 1.482600 | 3.00000 | 7.00000 | 4.00000 | -0.1020557 | -1.3444343 | 0.3482840 |
| anthro_hips | 8 | 13 | 99.423077 | 8.9419567 | 96.00000 | 98.190909 | 6.523440 | 89.10000 | 123.30000 | 34.20000 | 1.3051057 | 1.1862932 | 2.4800526 |
| baseline_stress_state | 9 | 13 | 3.384615 | 2.1031112 | 3.00000 | 3.272727 | 1.482600 | 1.00000 | 7.00000 | 6.00000 | 0.5211141 | -1.4639635 | 0.5832981 |
| POST_EOS_stress | 10 | 14 | 2.785714 | 1.5281246 | 2.00000 | 2.666667 | 1.482600 | 1.00000 | 6.00000 | 5.00000 | 0.8137461 | -0.6755596 | 0.4084085 |
| POST_EOS_PA | 11 | 14 | 26.857143 | 6.3712955 | 27.00000 | 27.000000 | 5.189100 | 16.00000 | 36.00000 | 20.00000 | -0.4418281 | -1.0335457 | 1.7028004 |
| POST_EOS_NA | 12 | 14 | 12.571429 | 2.9797854 | 11.50000 | 12.166667 | 2.223900 | 10.00000 | 20.00000 | 10.00000 | 1.1487539 | 0.2940337 | 0.7963811 |
| POST_EOS_KSS | 13 | 14 | 5.357143 | 1.5984195 | 5.50000 | 5.333333 | 2.223900 | 3.00000 | 8.00000 | 5.00000 | -0.0214168 | -1.4341298 | 0.4271956 |
| PRE_EOS_stress | 14 | 13 | 4.384615 | 1.8045526 | 5.00000 | 4.454546 | 1.482600 | 1.00000 | 7.00000 | 6.00000 | -0.2202880 | -1.0274760 | 0.5004929 |
| PRE_EOS_PA | 15 | 13 | 31.076923 | 6.0340912 | 30.00000 | 31.272727 | 7.413000 | 20.00000 | 40.00000 | 20.00000 | -0.2597335 | -1.3002539 | 1.6735558 |
| PRE_EOS_NA | 16 | 13 | 16.615385 | 7.2057669 | 14.00000 | 15.909091 | 4.447800 | 10.00000 | 31.00000 | 21.00000 | 0.9709660 | -0.5932105 | 1.9985202 |
| baseline_PA | 17 | 13 | 31.846154 | 5.5804145 | 31.00000 | 32.181818 | 4.447800 | 20.00000 | 40.00000 | 20.00000 | -0.3441487 | -0.7128067 | 1.5477285 |
| baseline_NA | 18 | 13 | 15.923077 | 7.3537013 | 14.00000 | 14.545454 | 5.930400 | 10.00000 | 37.00000 | 27.00000 | 1.6896219 | 2.3374676 | 2.0395498 |
| demo_age | 19 | 13 | 32.615385 | 10.3966217 | 30.00000 | 32.090909 | 11.860800 | 19.00000 | 52.00000 | 33.00000 | 0.4450936 | -1.2450099 | 2.8835041 |
| demo_sex* | 20 | 14 | 1.500000 | 0.6504436 | 1.00000 | 1.416667 | 0.000000 | 1.00000 | 3.00000 | 2.00000 | 0.7786893 | -0.6555490 | 0.1738384 |
| demo_smoke* | 21 | 14 | 1.357143 | 0.6333237 | 1.00000 | 1.250000 | 0.000000 | 1.00000 | 3.00000 | 2.00000 | 1.3428099 | 0.5339258 | 0.1692629 |
| BMI | 22 | 13 | 22.876202 | 3.1428852 | 22.37691 | 22.827963 | 3.025957 | 17.69466 | 28.58837 | 10.89371 | 0.2582160 | -1.1211527 | 0.8716795 |

### 3.2. Physiological data

Summary statistics for Session 1 and Baseline phase

|  | vars | n | mean | sd | median | trimmed | mad | min | max | range | skew | kurtosis | se |
| --- | --- | --- | --- | --- | --- | --- | --- | --- | --- | --- | --- | --- | --- |
| Phase* | 1 | 28 | 1.000000 | 0.0000000 | 1.00000 | 1.000000 | 0.0000000 | 1.00000 | 1.0000 | 0.00000 | NaN | NaN | 0.0000000 |
| HRV_RMSSD | 2 | 28 | 50.966519 | 28.7710761 | 46.50073 | 48.282642 | 24.8863191 | 17.78861 | 134.9941 | 117.20553 | 1.0414298 | 0.5158308 | 5.4372223 |
| HRV_lf | 3 | 28 | 1007.265680 | 917.5292387 | 662.15790 | 854.355385 | 410.6490382 | 158.70515 | 3953.2617 | 3794.55653 | 1.7901302 | 2.7648399 | 173.3967276 |
| duration | 4 | 28 | 302.450767 | 0.4024055 | 302.46181 | 302.462662 | 0.4801385 | 301.44027 | 303.2096 | 1.76938 | -0.2139645 | -0.2757838 | 0.0760475 |
| EOS_Control* | 5 | 28 | 1.500000 | 0.5091751 | 1.50000 | 1.500000 | 0.7413000 | 1.00000 | 2.0000 | 1.00000 | 0.0000000 | -2.0701531 | 0.0962250 |
| Subject* | 6 | 28 | 14.500000 | 8.2259751 | 14.50000 | 14.500000 | 10.3782000 | 1.00000 | 28.0000 | 27.00000 | 0.0000000 | -1.3291256 | 1.5545632 |
| Session* | 7 | 28 | 1.000000 | 0.0000000 | 1.00000 | 1.000000 | 0.0000000 | 1.00000 | 1.0000 | 0.00000 | NaN | NaN | 0.0000000 |
| demo_sex* | 8 | 28 | 1.428571 | 0.5039526 | 1.00000 | 1.416667 | 0.0000000 | 1.00000 | 2.0000 | 1.00000 | 0.2733493 | -1.9926658 | 0.0952381 |

Summary statistics for Session 1 and MAST Instruction phase

|  | vars | n | mean | sd | median | trimmed | mad | min | max | range | skew | kurtosis | se |
| --- | --- | --- | --- | --- | --- | --- | --- | --- | --- | --- | --- | --- | --- |
| Phase* | 1 | 28 | 4.000000 | 0.0000000 | 4.00000 | 4.000000 | 0.00000 | 4.00000 | 4.0000 | 0.00000 | NaN | NaN | 0.0000000 |
| HRV_RMSSD | 2 | 28 | 47.707382 | 21.0687977 | 42.92716 | 45.145901 | 15.82220 | 21.60226 | 108.9323 | 87.32999 | 1.2914614 | 1.2227173 | 3.9816285 |
| HRV_lf | 3 | 28 | 1481.770499 | 796.2451835 | 1382.76311 | 1434.240839 | 865.35733 | 319.33507 | 3397.4259 | 3078.09088 | 0.5636804 | -0.5169186 | 150.4761956 |
| duration | 4 | 28 | 391.427974 | 140.5399398 | 350.94255 | 366.291482 | 58.25041 | 256.94495 | 978.7763 | 721.83132 | 2.7975681 | 8.5756156 | 26.5595521 |
| EOS_Control* | 5 | 28 | 1.500000 | 0.5091751 | 1.50000 | 1.500000 | 0.74130 | 1.00000 | 2.0000 | 1.00000 | 0.0000000 | -2.0701531 | 0.0962250 |
| Subject* | 6 | 28 | 14.500000 | 8.2259751 | 14.50000 | 14.500000 | 10.37820 | 1.00000 | 28.0000 | 27.00000 | 0.0000000 | -1.3291256 | 1.5545632 |
| Session* | 7 | 28 | 1.000000 | 0.0000000 | 1.00000 | 1.000000 | 0.00000 | 1.00000 | 1.0000 | 0.00000 | NaN | NaN | 0.0000000 |
| demo_sex* | 8 | 28 | 1.428571 | 0.5039526 | 1.00000 | 1.416667 | 0.00000 | 1.00000 | 2.0000 | 1.00000 | 0.2733493 | -1.9926658 | 0.0952381 |

Summary statistics for Session 1 and MAST Task phase

|  | vars | n | mean | sd | median | trimmed | mad | min | max | range | skew | kurtosis | se |
| --- | --- | --- | --- | --- | --- | --- | --- | --- | --- | --- | --- | --- | --- |
| Phase* | 1 | 28 | 5.000000 | 0.0000000 | 5.00000 | 5.000000 | 0.0000000 | 5.00000 | 5.0000 | 0.000000 | NaN | NaN | 0.0000000 |
| HRV_RMSSD | 2 | 28 | 37.943912 | 20.6658946 | 34.58574 | 34.474062 | 11.6010663 | 19.10044 | 121.8318 | 102.731364 | 2.4963374 | 7.218689 | 3.9054870 |
| HRV_lf | 3 | 28 | 963.202306 | 518.7808982 | 892.61656 | 913.734672 | 401.0511674 | 302.92654 | 2691.6372 | 2388.710701 | 1.3258246 | 2.111574 | 98.0403744 |
| duration | 4 | 28 | 603.841724 | 0.7809757 | 603.53408 | 603.840636 | 0.9258844 | 602.33421 | 605.4392 | 3.105038 | 0.0827539 | -1.062652 | 0.1475905 |
| EOS_Control* | 5 | 28 | 1.500000 | 0.5091751 | 1.50000 | 1.500000 | 0.7413000 | 1.00000 | 2.0000 | 1.000000 | 0.0000000 | -2.070153 | 0.0962250 |
| Subject* | 6 | 28 | 14.500000 | 8.2259751 | 14.50000 | 14.500000 | 10.3782000 | 1.00000 | 28.0000 | 27.000000 | 0.0000000 | -1.329126 | 1.5545632 |
| Session* | 7 | 28 | 1.000000 | 0.0000000 | 1.00000 | 1.000000 | 0.0000000 | 1.00000 | 1.0000 | 0.000000 | NaN | NaN | 0.0000000 |
| demo_sex* | 8 | 28 | 1.428571 | 0.5039526 | 1.00000 | 1.416667 | 0.0000000 | 1.00000 | 2.0000 | 1.000000 | 0.2733493 | -1.992666 | 0.0952381 |

Summary statistics for Session 1, Baseline phase, and Women

|  | vars | n | mean | sd | median | trimmed | mad | min | max | range | skew | kurtosis | se |
| --- | --- | --- | --- | --- | --- | --- | --- | --- | --- | --- | --- | --- | --- |
| Phase* | 1 | 16 | 1.00000 | 0.0000000 | 1.00000 | 1.000000 | 0.0000000 | 1.00000 | 1.0000 | 0.00000 | NaN | NaN | 0.0000000 |
| HRV_RMSSD | 2 | 16 | 50.83292 | 30.5991210 | 46.50073 | 47.150639 | 21.4297191 | 18.22357 | 134.9941 | 116.77057 | 1.2728366 | 1.0839833 | 7.6497802 |
| HRV_lf | 3 | 16 | 588.62146 | 343.1921275 | 510.72643 | 566.342156 | 220.9833812 | 158.70515 | 1330.4480 | 1171.74286 | 0.7654483 | -0.5223323 | 85.7980319 |
| duration | 4 | 16 | 302.31587 | 0.4325407 | 302.22902 | 302.314572 | 0.3451315 | 301.44027 | 303.2096 | 1.76938 | 0.2360098 | -0.1427719 | 0.1081352 |
| EOS_Control* | 5 | 16 | 1.43750 | 0.5123475 | 1.00000 | 1.428571 | 0.0000000 | 1.00000 | 2.0000 | 1.00000 | 0.2287266 | -2.0652902 | 0.1280869 |
| Subject* | 6 | 16 | 8.50000 | 4.7609523 | 8.50000 | 8.500000 | 5.9304000 | 1.00000 | 16.0000 | 15.00000 | 0.0000000 | -1.4262408 | 1.1902381 |
| Session* | 7 | 16 | 1.00000 | 0.0000000 | 1.00000 | 1.000000 | 0.0000000 | 1.00000 | 1.0000 | 0.00000 | NaN | NaN | 0.0000000 |
| demo_sex* | 8 | 16 | 1.00000 | 0.0000000 | 1.00000 | 1.000000 | 0.0000000 | 1.00000 | 1.0000 | 0.00000 | NaN | NaN | 0.0000000 |

Summary statistics for Session 1, MAST Instruction phase, and Women

|  | vars | n | mean | sd | median | trimmed | mad | min | max | range | skew | kurtosis | se |
| --- | --- | --- | --- | --- | --- | --- | --- | --- | --- | --- | --- | --- | --- |
| Phase* | 1 | 16 | 4.00000 | 0.0000000 | 4.00000 | 4.000000 | 0.00000 | 4.00000 | 4.0000 | 0.00000 | NaN | NaN | 0.0000000 |
| HRV_RMSSD | 2 | 16 | 47.36126 | 22.9136014 | 42.88802 | 44.803264 | 17.36427 | 21.60226 | 108.9323 | 87.32999 | 1.2276647 | 0.8417371 | 5.7284004 |
| HRV_lf | 3 | 16 | 1282.08020 | 672.3163927 | 1234.82324 | 1258.256236 | 785.05329 | 319.33507 | 2578.3608 | 2259.02576 | 0.2219064 | -1.2162934 | 168.0790982 |
| duration | 4 | 16 | 399.74741 | 166.0621977 | 350.94255 | 368.588385 | 87.23559 | 256.94495 | 978.7763 | 721.83132 | 2.5458051 | 6.3213739 | 41.5155494 |
| EOS_Control* | 5 | 16 | 1.43750 | 0.5123475 | 1.00000 | 1.428571 | 0.00000 | 1.00000 | 2.0000 | 1.00000 | 0.2287266 | -2.0652902 | 0.1280869 |
| Subject* | 6 | 16 | 8.50000 | 4.7609523 | 8.50000 | 8.500000 | 5.93040 | 1.00000 | 16.0000 | 15.00000 | 0.0000000 | -1.4262408 | 1.1902381 |
| Session* | 7 | 16 | 1.00000 | 0.0000000 | 1.00000 | 1.000000 | 0.00000 | 1.00000 | 1.0000 | 0.00000 | NaN | NaN | 0.0000000 |
| demo_sex* | 8 | 16 | 1.00000 | 0.0000000 | 1.00000 | 1.000000 | 0.00000 | 1.00000 | 1.0000 | 0.00000 | NaN | NaN | 0.0000000 |

Summary statistics for Session 1, MAST Task phase, and Women

|  | vars | n | mean | sd | median | trimmed | mad | min | max | range | skew | kurtosis | se |
| --- | --- | --- | --- | --- | --- | --- | --- | --- | --- | --- | --- | --- | --- |
| Phase* | 1 | 16 | 5.00000 | 0.0000000 | 5.00000 | 5.000000 | 0.0000000 | 5.00000 | 5.0000 | 0.000000 | NaN | NaN | 0.0000000 |
| HRV_RMSSD | 2 | 16 | 40.23563 | 25.9354690 | 32.11731 | 35.916983 | 10.3132855 | 19.10044 | 121.8318 | 102.731364 | 1.9970451 | 3.4144300 | 6.4838673 |
| HRV_lf | 3 | 16 | 816.89918 | 327.4240499 | 851.09596 | 807.137497 | 297.9972960 | 302.92654 | 1467.5353 | 1164.608776 | 0.1012771 | -0.9217946 | 81.8560125 |
| duration | 4 | 16 | 603.83839 | 0.7596899 | 603.49021 | 603.791565 | 0.8304695 | 602.89310 | 605.4392 | 2.546146 | 0.5096873 | -1.0334308 | 0.1899225 |
| EOS_Control* | 5 | 16 | 1.43750 | 0.5123475 | 1.00000 | 1.428571 | 0.0000000 | 1.00000 | 2.0000 | 1.000000 | 0.2287266 | -2.0652902 | 0.1280869 |
| Subject* | 6 | 16 | 8.50000 | 4.7609523 | 8.50000 | 8.500000 | 5.9304000 | 1.00000 | 16.0000 | 15.000000 | 0.0000000 | -1.4262408 | 1.1902381 |
| Session* | 7 | 16 | 1.00000 | 0.0000000 | 1.00000 | 1.000000 | 0.0000000 | 1.00000 | 1.0000 | 0.000000 | NaN | NaN | 0.0000000 |
| demo_sex* | 8 | 16 | 1.00000 | 0.0000000 | 1.00000 | 1.000000 | 0.0000000 | 1.00000 | 1.0000 | 0.000000 | NaN | NaN | 0.0000000 |

Summary statistics for Session 1, Baseline phase, and Men

|  | vars | n | mean | sd | median | trimmed | mad | min | max | range | skew | kurtosis | se |
| --- | --- | --- | --- | --- | --- | --- | --- | --- | --- | --- | --- | --- | --- |
| Phase* | 1 | 12 | 1.000000 | 0.0000000 | 1.00000 | 1.00000 | 0.0000000 | 1.00000 | 1.00000 | 0.000000 | NaN | NaN | 0.0000000 |
| HRV_RMSSD | 2 | 12 | 51.144656 | 27.4767019 | 43.80001 | 49.86744 | 22.2734106 | 17.78861 | 97.27281 | 79.484198 | 0.4507085 | -1.4797143 | 7.9318406 |
| HRV_lf | 3 | 12 | 1565.457973 | 1144.9634178 | 1307.20553 | 1455.45765 | 783.6745110 | 277.65753 | 3953.26169 | 3675.604159 | 0.8538762 | -0.5819739 | 330.5224687 |
| duration | 4 | 12 | 302.630631 | 0.2838659 | 302.63090 | 302.64431 | 0.3458179 | 302.13558 | 302.98888 | 0.853306 | -0.2516703 | -1.4830713 | 0.0819450 |
| EOS_Control* | 5 | 12 | 1.583333 | 0.5149287 | 2.00000 | 1.60000 | 0.0000000 | 1.00000 | 2.00000 | 1.000000 | -0.2966970 | -2.0636905 | 0.1486471 |
| Subject* | 6 | 12 | 6.500000 | 3.6055513 | 6.50000 | 6.50000 | 4.4478000 | 1.00000 | 12.00000 | 11.000000 | 0.0000000 | -1.5016026 | 1.0408330 |
| Session* | 7 | 12 | 1.000000 | 0.0000000 | 1.00000 | 1.00000 | 0.0000000 | 1.00000 | 1.00000 | 0.000000 | NaN | NaN | 0.0000000 |
| demo_sex* | 8 | 12 | 2.000000 | 0.0000000 | 2.00000 | 2.00000 | 0.0000000 | 2.00000 | 2.00000 | 0.000000 | NaN | NaN | 0.0000000 |

Summary statistics for Session 1, MAST Instruction phase, and Men

|  | vars | n | mean | sd | median | trimmed | mad | min | max | range | skew | kurtosis | se |
| --- | --- | --- | --- | --- | --- | --- | --- | --- | --- | --- | --- | --- | --- |
| Phase* | 1 | 12 | 4.000000 | 0.0000000 | 4.00000 | 4.00000 | 0.00000 | 4.00000 | 4.00000 | 0.00000 | NaN | NaN | 0.0000000 |
| HRV_RMSSD | 2 | 12 | 48.168873 | 19.3183261 | 42.92716 | 45.62559 | 13.22177 | 24.19444 | 97.57611 | 73.38167 | 1.2064221 | 0.8753073 | 5.5767204 |
| HRV_lf | 3 | 12 | 1748.024231 | 896.9301465 | 1661.67113 | 1683.78081 | 1121.13572 | 741.05673 | 3397.42595 | 2656.36921 | 0.4368849 | -1.2646670 | 258.9214308 |
| duration | 4 | 12 | 380.335387 | 103.1574520 | 345.86012 | 358.13603 | 47.02854 | 307.76144 | 674.90290 | 367.14146 | 1.8586594 | 2.6274442 | 29.7789913 |
| EOS_Control* | 5 | 12 | 1.583333 | 0.5149287 | 2.00000 | 1.60000 | 0.00000 | 1.00000 | 2.00000 | 1.00000 | -0.2966970 | -2.0636905 | 0.1486471 |
| Subject* | 6 | 12 | 6.500000 | 3.6055513 | 6.50000 | 6.50000 | 4.44780 | 1.00000 | 12.00000 | 11.00000 | 0.0000000 | -1.5016026 | 1.0408330 |
| Session* | 7 | 12 | 1.000000 | 0.0000000 | 1.00000 | 1.00000 | 0.00000 | 1.00000 | 1.00000 | 0.00000 | NaN | NaN | 0.0000000 |
| demo_sex* | 8 | 12 | 2.000000 | 0.0000000 | 2.00000 | 2.00000 | 0.00000 | 2.00000 | 2.00000 | 0.00000 | NaN | NaN | 0.0000000 |

Summary statistics for Session 1, MAST Task phase, and Men

|  | vars | n | mean | sd | median | trimmed | mad | min | max | range | skew | kurtosis | se |
| --- | --- | --- | --- | --- | --- | --- | --- | --- | --- | --- | --- | --- | --- |
| Phase* | 1 | 12 | 5.000000 | 0.0000000 | 5.0000 | 5.00000 | 0.0000000 | 5.00000 | 5.00000 | 0.000000 | NaN | NaN | 0.0000000 |
| HRV_RMSSD | 2 | 12 | 34.888293 | 10.6401347 | 37.0729 | 34.44986 | 11.1476574 | 19.66456 | 54.49635 | 34.831786 | 0.1162592 | -1.2222527 | 3.0715423 |
| HRV_lf | 3 | 12 | 1158.273146 | 664.6533502 | 942.9100 | 1083.08442 | 622.5703934 | 376.79634 | 2691.63724 | 2314.840900 | 0.8370657 | -0.2773948 | 191.8688953 |
| duration | 4 | 12 | 603.846169 | 0.8426462 | 603.9073 | 603.88877 | 0.9664158 | 602.33421 | 604.93214 | 2.597939 | -0.3467081 | -1.3818649 | 0.2432510 |
| EOS_Control* | 5 | 12 | 1.583333 | 0.5149287 | 2.0000 | 1.60000 | 0.0000000 | 1.00000 | 2.00000 | 1.000000 | -0.2966970 | -2.0636905 | 0.1486471 |
| Subject* | 6 | 12 | 6.500000 | 3.6055513 | 6.5000 | 6.50000 | 4.4478000 | 1.00000 | 12.00000 | 11.000000 | 0.0000000 | -1.5016026 | 1.0408330 |
| Session* | 7 | 12 | 1.000000 | 0.0000000 | 1.0000 | 1.00000 | 0.0000000 | 1.00000 | 1.00000 | 0.000000 | NaN | NaN | 0.0000000 |
| demo_sex* | 8 | 12 | 2.000000 | 0.0000000 | 2.0000 | 2.00000 | 0.0000000 | 2.00000 | 2.00000 | 0.000000 | NaN | NaN | 0.0000000 |

Summary statistics for Session 1, Baseline phase, and EOS intervention

|  | vars | n | mean | sd | median | trimmed | mad | min | max | range | skew | kurtosis | se |
| --- | --- | --- | --- | --- | --- | --- | --- | --- | --- | --- | --- | --- | --- |
| Phase* | 1 | 14 | 1.000000 | 0.0000000 | 1.00000 | 1.000000 | 0.0000000 | 1.00000 | 1.00000 | 0.00000 | NaN | NaN | 0.0000000 |
| HRV_RMSSD | 2 | 14 | 43.233183 | 18.6408327 | 46.50073 | 42.221292 | 20.2458688 | 17.78861 | 80.82045 | 63.03184 | 0.2024460 | -1.0811174 | 4.9819721 |
| HRV_lf | 3 | 14 | 657.581403 | 428.8567876 | 464.02987 | 614.420586 | 290.8165347 | 159.91939 | 1673.17322 | 1513.25383 | 0.8795868 | -0.2781088 | 114.6167977 |
| duration | 4 | 14 | 302.476511 | 0.3657413 | 302.42995 | 302.449359 | 0.4426399 | 302.06920 | 303.20965 | 1.14045 | 0.5761561 | -1.1361841 | 0.0977485 |
| EOS_Control* | 5 | 14 | 1.000000 | 0.0000000 | 1.00000 | 1.000000 | 0.0000000 | 1.00000 | 1.00000 | 0.00000 | NaN | NaN | 0.0000000 |
| Subject* | 6 | 14 | 7.500000 | 4.1833001 | 7.50000 | 7.500000 | 5.1891000 | 1.00000 | 14.00000 | 13.00000 | 0.0000000 | -1.4585714 | 1.1180340 |
| Session* | 7 | 14 | 1.000000 | 0.0000000 | 1.00000 | 1.000000 | 0.0000000 | 1.00000 | 1.00000 | 0.00000 | NaN | NaN | 0.0000000 |
| demo_sex* | 8 | 14 | 1.357143 | 0.4972452 | 1.00000 | 1.333333 | 0.0000000 | 1.00000 | 2.00000 | 1.00000 | 0.5335519 | -1.8311791 | 0.1328944 |

Summary statistics for Session 1, Baseline phase, and Control intervention

|  | vars | n | mean | sd | median | trimmed | mad | min | max | range | skew | kurtosis | se |
| --- | --- | --- | --- | --- | --- | --- | --- | --- | --- | --- | --- | --- | --- |
| Phase* | 1 | 14 | 1.00000 | 0.0000000 | 1.00000 | 1.00000 | 0.0000000 | 1.00000 | 1.0000 | 0.000000 | NaN | NaN | 0.0000000 |
| HRV_RMSSD | 2 | 14 | 58.69986 | 35.2552736 | 46.57909 | 55.31411 | 30.9030459 | 23.03449 | 134.9941 | 111.959657 | 0.6618755 | -0.9333101 | 9.4223682 |
| HRV_lf | 3 | 14 | 1356.94996 | 1140.6970517 | 860.93067 | 1240.44438 | 612.7312728 | 158.70515 | 3953.2617 | 3794.556535 | 1.0832109 | -0.1142388 | 304.8641107 |
| duration | 4 | 14 | 302.42502 | 0.4484668 | 302.51513 | 302.45498 | 0.4520225 | 301.44027 | 303.0503 | 1.610029 | -0.5681140 | -0.5253899 | 0.1198578 |
| EOS_Control* | 5 | 14 | 1.00000 | 0.0000000 | 1.00000 | 1.00000 | 0.0000000 | 1.00000 | 1.0000 | 0.000000 | NaN | NaN | 0.0000000 |
| Subject* | 6 | 14 | 7.50000 | 4.1833001 | 7.50000 | 7.50000 | 5.1891000 | 1.00000 | 14.0000 | 13.000000 | 0.0000000 | -1.4585714 | 1.1180340 |
| Session* | 7 | 14 | 1.00000 | 0.0000000 | 1.00000 | 1.00000 | 0.0000000 | 1.00000 | 1.0000 | 0.000000 | NaN | NaN | 0.0000000 |
| demo_sex* | 8 | 14 | 1.50000 | 0.5188745 | 1.50000 | 1.50000 | 0.7413000 | 1.00000 | 2.0000 | 1.000000 | 0.0000000 | -2.1377551 | 0.1386750 |

Summary statistics for Session 2, Baseline phase, and EOS intervention

|  | vars | n | mean | sd | median | trimmed | mad | min | max | range | skew | kurtosis | se |
| --- | --- | --- | --- | --- | --- | --- | --- | --- | --- | --- | --- | --- | --- |
| Phase* | 1 | 14 | 1.00000 | 0.0000000 | 1.00000 | 1.00000 | 0.0000000 | 1.00000 | 1.0000 | 0.000000 | NaN | NaN | 0.0000000 |
| HRV_RMSSD | 2 | 14 | 58.62299 | 44.0028607 | 39.67213 | 53.11991 | 19.7091252 | 22.74951 | 160.5335 | 137.783974 | 1.350793 | 0.3349562 | 11.7602592 |
| HRV_lf | 3 | 14 | 1450.33600 | 1808.1781180 | 1001.60747 | 1074.65811 | 611.8046786 | 111.72968 | 7297.0770 | 7185.347300 | 2.356299 | 4.8788147 | 483.2559294 |
| duration | 4 | 14 | 302.33789 | 0.7729210 | 302.48933 | 302.46461 | 0.4880623 | 300.00000 | 303.1552 | 3.155207 | -1.796788 | 3.1690167 | 0.2065718 |
| EOS_Control* | 5 | 14 | 1.00000 | 0.0000000 | 1.00000 | 1.00000 | 0.0000000 | 1.00000 | 1.0000 | 0.000000 | NaN | NaN | 0.0000000 |
| Subject* | 6 | 14 | 7.50000 | 4.1833001 | 7.50000 | 7.50000 | 5.1891000 | 1.00000 | 14.0000 | 13.000000 | 0.000000 | -1.4585714 | 1.1180340 |
| Session* | 7 | 14 | 2.00000 | 0.0000000 | 2.00000 | 2.00000 | 0.0000000 | 2.00000 | 2.0000 | 0.000000 | NaN | NaN | 0.0000000 |
| demo_sex* | 8 | 14 | 1.50000 | 0.5188745 | 1.50000 | 1.50000 | 0.7413000 | 1.00000 | 2.0000 | 1.000000 | 0.000000 | -2.1377551 | 0.1386750 |

Summary statistics for Session 2, Baseline phase, and Control intervention

|  | vars | n | mean | sd | median | trimmed | mad | min | max | range | skew | kurtosis | se |
| --- | --- | --- | --- | --- | --- | --- | --- | --- | --- | --- | --- | --- | --- |
| Phase* | 1 | 13 | 1.000000 | 0.0000000 | 1.00000 | 1.000000 | 0.0000000 | 1.00000 | 1.00000 | 0.000000 | NaN | NaN | 0.0000000 |
| HRV_RMSSD | 2 | 13 | 41.330034 | 13.7946301 | 41.24059 | 41.035683 | 13.0029690 | 22.04111 | 63.85683 | 41.815715 | 0.1761574 | -1.3646451 | 3.8259420 |
| HRV_lf | 3 | 13 | 743.312749 | 339.6496782 | 840.68592 | 725.572769 | 371.9480369 | 268.27413 | 1413.49115 | 1145.217024 | 0.1562521 | -1.0517925 | 94.2018716 |
| duration | 4 | 13 | 302.346324 | 0.4608665 | 302.30837 | 302.359044 | 0.4085616 | 301.37456 | 303.17816 | 1.803602 | -0.2498469 | -0.4091556 | 0.1278214 |
| EOS_Control* | 5 | 13 | 1.000000 | 0.0000000 | 1.00000 | 1.000000 | 0.0000000 | 1.00000 | 1.00000 | 0.000000 | NaN | NaN | 0.0000000 |
| Subject* | 6 | 13 | 7.000000 | 3.8944405 | 7.00000 | 7.000000 | 4.4478000 | 1.00000 | 13.00000 | 12.000000 | 0.0000000 | -1.4784446 | 1.0801234 |
| Session* | 7 | 13 | 2.000000 | 0.0000000 | 2.00000 | 2.000000 | 0.0000000 | 2.00000 | 2.00000 | 0.000000 | NaN | NaN | 0.0000000 |
| demo_sex* | 8 | 13 | 1.538461 | 0.6602253 | 1.00000 | 1.454546 | 0.0000000 | 1.00000 | 3.00000 | 2.000000 | 0.6737567 | -0.8226490 | 0.1831135 |

Summary statistics for Session 1 and Baseline phase

|  | vars | n | mean | sd | median | trimmed | mad | min | max | range | skew | kurtosis | se |
| --- | --- | --- | --- | --- | --- | --- | --- | --- | --- | --- | --- | --- | --- |
| Phase* | 1 | 28 | 1.0000000 | 0.0000000 | 1.0000000 | 1.0000000 | 0.0000000 | 1.0000000 | 1.000000 | 0.000000 | NaN | NaN | 0.0000000 |
| alpha_asymmetry | 2 | 28 | 0.1930659 | 0.4574121 | 0.1184083 | 0.1603349 | 0.2379261 | -0.4943002 | 1.717371 | 2.211671 | 1.2241383 | 2.2441711 | 0.0864428 |
| duration | 3 | 28 | 302.4507674 | 0.4024055 | 302.4618055 | 302.4626624 | 0.4801385 | 301.4402660 | 303.209646 | 1.769380 | -0.2139645 | -0.2757838 | 0.0760475 |
| EOS_Control* | 4 | 28 | 1.5000000 | 0.5091751 | 1.5000000 | 1.5000000 | 0.7413000 | 1.0000000 | 2.000000 | 1.000000 | 0.0000000 | -2.0701531 | 0.0962250 |
| Subject* | 5 | 28 | 14.5000000 | 8.2259751 | 14.5000000 | 14.5000000 | 10.3782000 | 1.0000000 | 28.000000 | 27.000000 | 0.0000000 | -1.3291256 | 1.5545632 |
| Session* | 6 | 28 | 1.0000000 | 0.0000000 | 1.0000000 | 1.0000000 | 0.0000000 | 1.0000000 | 1.000000 | 0.000000 | NaN | NaN | 0.0000000 |
| demo_sex* | 7 | 28 | 1.4285714 | 0.5039526 | 1.0000000 | 1.4166667 | 0.0000000 | 1.0000000 | 2.000000 | 1.000000 | 0.2733493 | -1.9926658 | 0.0952381 |

Summary statistics for Session 1 and MAST Instruction phase

|  | vars | n | mean | sd | median | trimmed | mad | min | max | range | skew | kurtosis | se |
| --- | --- | --- | --- | --- | --- | --- | --- | --- | --- | --- | --- | --- | --- |
| Phase* | 1 | 28 | 4.0000000 | 0.0000000 | 4.0000000 | 4.0000000 | 0.0000000 | 4.0000000 | 4.000000 | 0.000000 | NaN | NaN | 0.0000000 |
| alpha_asymmetry | 2 | 28 | 0.1944691 | 0.3823356 | 0.2141505 | 0.1514581 | 0.2371054 | -0.5201593 | 1.516174 | 2.036334 | 1.5051107 | 3.609008 | 0.0722546 |
| duration | 3 | 28 | 391.4279736 | 140.5399398 | 350.9425530 | 366.2914818 | 58.2504133 | 256.9449540 | 978.776279 | 721.831325 | 2.7975681 | 8.575616 | 26.5595521 |
| EOS_Control* | 4 | 28 | 1.5000000 | 0.5091751 | 1.5000000 | 1.5000000 | 0.7413000 | 1.0000000 | 2.000000 | 1.000000 | 0.0000000 | -2.070153 | 0.0962250 |
| Subject* | 5 | 28 | 14.5000000 | 8.2259751 | 14.5000000 | 14.5000000 | 10.3782000 | 1.0000000 | 28.000000 | 27.000000 | 0.0000000 | -1.329126 | 1.5545632 |
| Session* | 6 | 28 | 1.0000000 | 0.0000000 | 1.0000000 | 1.0000000 | 0.0000000 | 1.0000000 | 1.000000 | 0.000000 | NaN | NaN | 0.0000000 |
| demo_sex* | 7 | 28 | 1.4285714 | 0.5039526 | 1.0000000 | 1.4166667 | 0.0000000 | 1.0000000 | 2.000000 | 1.000000 | 0.2733493 | -1.992666 | 0.0952381 |

Summary statistics for Session 1, Baseline phase, and Women

|  | vars | n | mean | sd | median | trimmed | mad | min | max | range | skew | kurtosis | se |
| --- | --- | --- | --- | --- | --- | --- | --- | --- | --- | --- | --- | --- | --- |
| Phase* | 1 | 16 | 1.000000 | 0.0000000 | 1.0000000 | 1.0000000 | 0.0000000 | 1.0000000 | 1.000000 | 0.000000 | NaN | NaN | 0.0000000 |
| alpha_asymmetry | 2 | 16 | 0.237813 | 0.5293029 | 0.1072304 | 0.1844241 | 0.3370958 | -0.4943002 | 1.717371 | 2.211671 | 1.2022780 | 1.3124434 | 0.1323257 |
| duration | 3 | 16 | 302.315870 | 0.4325407 | 302.2290175 | 302.3145721 | 0.3451315 | 301.4402660 | 303.209646 | 1.769380 | 0.2360098 | -0.1427719 | 0.1081352 |
| EOS_Control* | 4 | 16 | 1.437500 | 0.5123475 | 1.0000000 | 1.4285714 | 0.0000000 | 1.0000000 | 2.000000 | 1.000000 | 0.2287266 | -2.0652902 | 0.1280869 |
| Subject* | 5 | 16 | 8.500000 | 4.7609523 | 8.5000000 | 8.5000000 | 5.9304000 | 1.0000000 | 16.000000 | 15.000000 | 0.0000000 | -1.4262408 | 1.1902381 |
| Session* | 6 | 16 | 1.000000 | 0.0000000 | 1.0000000 | 1.0000000 | 0.0000000 | 1.0000000 | 1.000000 | 0.000000 | NaN | NaN | 0.0000000 |
| demo_sex* | 7 | 16 | 1.000000 | 0.0000000 | 1.0000000 | 1.0000000 | 0.0000000 | 1.0000000 | 1.000000 | 0.000000 | NaN | NaN | 0.0000000 |

Summary statistics for Session 1, MAST Instruction phase, and Women

|  | vars | n | mean | sd | median | trimmed | mad | min | max | range | skew | kurtosis | se |
| --- | --- | --- | --- | --- | --- | --- | --- | --- | --- | --- | --- | --- | --- |
| Phase* | 1 | 16 | 4.0000000 | 0.0000000 | 4.0000000 | 4.000000 | 0.0000000 | 4.0000000 | 4.000000 | 0.0000000 | NaN | NaN | 0.0000000 |
| alpha_asymmetry | 2 | 16 | 0.0546812 | 0.2411960 | 0.0430025 | 0.068294 | 0.2416881 | -0.5201593 | 0.438942 | 0.9591013 | -0.5120879 | -0.1271139 | 0.0602990 |
| duration | 3 | 16 | 399.7474140 | 166.0621977 | 350.9425530 | 368.588385 | 87.2355850 | 256.9449540 | 978.776279 | 721.8313250 | 2.5458051 | 6.3213739 | 41.5155494 |
| EOS_Control* | 4 | 16 | 1.4375000 | 0.5123475 | 1.0000000 | 1.428571 | 0.0000000 | 1.0000000 | 2.000000 | 1.0000000 | 0.2287266 | -2.0652902 | 0.1280869 |
| Subject* | 5 | 16 | 8.5000000 | 4.7609523 | 8.5000000 | 8.500000 | 5.9304000 | 1.0000000 | 16.000000 | 15.0000000 | 0.0000000 | -1.4262408 | 1.1902381 |
| Session* | 6 | 16 | 1.0000000 | 0.0000000 | 1.0000000 | 1.000000 | 0.0000000 | 1.0000000 | 1.000000 | 0.0000000 | NaN | NaN | 0.0000000 |
| demo_sex* | 7 | 16 | 1.0000000 | 0.0000000 | 1.0000000 | 1.000000 | 0.0000000 | 1.0000000 | 1.000000 | 0.0000000 | NaN | NaN | 0.0000000 |

Summary statistics for Session 1, Baseline phase, and Men

|  | vars | n | mean | sd | median | trimmed | mad | min | max | range | skew | kurtosis | se |
| --- | --- | --- | --- | --- | --- | --- | --- | --- | --- | --- | --- | --- | --- |
| Phase* | 1 | 12 | 1.000000 | 0.0000000 | 1.0000000 | 1.0000000 | 0.0000000 | 1.00000 | 1.0000000 | 0.000000 | NaN | NaN | 0.0000000 |
| alpha_asymmetry | 2 | 12 | 0.133403 | 0.3531571 | 0.1232823 | 0.1308809 | 0.1411033 | -0.48272 | 0.7747467 | 1.257467 | 0.2085643 | -0.6010386 | 0.1019477 |
| duration | 3 | 12 | 302.630631 | 0.2838659 | 302.6309020 | 302.6443105 | 0.3458179 | 302.13558 | 302.9888840 | 0.853306 | -0.2516703 | -1.4830713 | 0.0819450 |
| EOS_Control* | 4 | 12 | 1.583333 | 0.5149287 | 2.0000000 | 1.6000000 | 0.0000000 | 1.00000 | 2.0000000 | 1.000000 | -0.2966970 | -2.0636905 | 0.1486471 |
| Subject* | 5 | 12 | 6.500000 | 3.6055513 | 6.5000000 | 6.5000000 | 4.4478000 | 1.00000 | 12.0000000 | 11.000000 | 0.0000000 | -1.5016026 | 1.0408330 |
| Session* | 6 | 12 | 1.000000 | 0.0000000 | 1.0000000 | 1.0000000 | 0.0000000 | 1.00000 | 1.0000000 | 0.000000 | NaN | NaN | 0.0000000 |
| demo_sex* | 7 | 12 | 2.000000 | 0.0000000 | 2.0000000 | 2.0000000 | 0.0000000 | 2.00000 | 2.0000000 | 0.000000 | NaN | NaN | 0.0000000 |

Summary statistics for Session 1, MAST Instruction phase, and Men

|  | vars | n | mean | sd | median | trimmed | mad | min | max | range | skew | kurtosis | se |
| --- | --- | --- | --- | --- | --- | --- | --- | --- | --- | --- | --- | --- | --- |
| Phase* | 1 | 12 | 4.0000000 | 0.0000000 | 4.0000000 | 4.0000000 | 0.0000000 | 4.0000000 | 4.000000 | 0.00000 | NaN | NaN | 0.0000000 |
| alpha_asymmetry | 2 | 12 | 0.3808531 | 0.4616889 | 0.2695983 | 0.3264938 | 0.0973631 | -0.2108756 | 1.516174 | 1.72705 | 1.262759 | 0.6885091 | 0.1332781 |
| duration | 3 | 12 | 380.3353865 | 103.1574520 | 345.8601245 | 358.1360294 | 47.0285375 | 307.7614420 | 674.902902 | 367.14146 | 1.858659 | 2.6274442 | 29.7789913 |
| EOS_Control* | 4 | 12 | 1.5833333 | 0.5149287 | 2.0000000 | 1.6000000 | 0.0000000 | 1.0000000 | 2.000000 | 1.00000 | -0.296697 | -2.0636905 | 0.1486471 |
| Subject* | 5 | 12 | 6.5000000 | 3.6055513 | 6.5000000 | 6.5000000 | 4.4478000 | 1.0000000 | 12.000000 | 11.00000 | 0.000000 | -1.5016026 | 1.0408330 |
| Session* | 6 | 12 | 1.0000000 | 0.0000000 | 1.0000000 | 1.0000000 | 0.0000000 | 1.0000000 | 1.000000 | 0.00000 | NaN | NaN | 0.0000000 |
| demo_sex* | 7 | 12 | 2.0000000 | 0.0000000 | 2.0000000 | 2.0000000 | 0.0000000 | 2.0000000 | 2.000000 | 0.00000 | NaN | NaN | 0.0000000 |

Summary statistics for Session 1, Baseline phase, and EOS intervention

|  | vars | n | mean | sd | median | trimmed | mad | min | max | range | skew | kurtosis | se |
| --- | --- | --- | --- | --- | --- | --- | --- | --- | --- | --- | --- | --- | --- |
| Phase* | 1 | 14 | 1.0000000 | 0.0000000 | 1.0000000 | 1.0000000 | 0.0000000 | 1.0000000 | 1.000000 | 0.000000 | NaN | NaN | 0.0000000 |
| alpha_asymmetry | 2 | 14 | 0.2183503 | 0.5740310 | 0.1033946 | 0.1528195 | 0.5384002 | -0.4943002 | 1.717371 | 2.211671 | 1.1108405 | 0.7319814 | 0.1534162 |
| duration | 3 | 14 | 302.4765110 | 0.3657413 | 302.4299540 | 302.4493593 | 0.4426399 | 302.0691960 | 303.209646 | 1.140450 | 0.5761561 | -1.1361841 | 0.0977485 |
| EOS_Control* | 4 | 14 | 1.0000000 | 0.0000000 | 1.0000000 | 1.0000000 | 0.0000000 | 1.0000000 | 1.000000 | 0.000000 | NaN | NaN | 0.0000000 |
| Subject* | 5 | 14 | 7.5000000 | 4.1833001 | 7.5000000 | 7.5000000 | 5.1891000 | 1.0000000 | 14.000000 | 13.000000 | 0.0000000 | -1.4585714 | 1.1180340 |
| Session* | 6 | 14 | 1.0000000 | 0.0000000 | 1.0000000 | 1.0000000 | 0.0000000 | 1.0000000 | 1.000000 | 0.000000 | NaN | NaN | 0.0000000 |
| demo_sex* | 7 | 14 | 1.3571429 | 0.4972452 | 1.0000000 | 1.3333333 | 0.0000000 | 1.0000000 | 2.000000 | 1.000000 | 0.5335519 | -1.8311791 | 0.1328944 |

Summary statistics for Session 1, Baseline phase, and Control intervention

|  | vars | n | mean | sd | median | trimmed | mad | min | max | range | skew | kurtosis | se |
| --- | --- | --- | --- | --- | --- | --- | --- | --- | --- | --- | --- | --- | --- |
| Phase* | 1 | 14 | 1.0000000 | 0.0000000 | 1.0000000 | 1.0000000 | 0.0000000 | 1.00000 | 1.000000 | 0.000000 | NaN | NaN | 0.0000000 |
| alpha_asymmetry | 2 | 14 | 0.1677815 | 0.3219586 | 0.1566375 | 0.1678504 | 0.1578114 | -0.48272 | 0.817456 | 1.300176 | 0.2481776 | -0.0251348 | 0.0860470 |
| duration | 3 | 14 | 302.4250239 | 0.4484668 | 302.5151345 | 302.4549811 | 0.4520225 | 301.44027 | 303.050295 | 1.610029 | -0.5681140 | -0.5253899 | 0.1198578 |
| EOS_Control* | 4 | 14 | 1.0000000 | 0.0000000 | 1.0000000 | 1.0000000 | 0.0000000 | 1.00000 | 1.000000 | 0.000000 | NaN | NaN | 0.0000000 |
| Subject* | 5 | 14 | 7.5000000 | 4.1833001 | 7.5000000 | 7.5000000 | 5.1891000 | 1.00000 | 14.000000 | 13.000000 | 0.0000000 | -1.4585714 | 1.1180340 |
| Session* | 6 | 14 | 1.0000000 | 0.0000000 | 1.0000000 | 1.0000000 | 0.0000000 | 1.00000 | 1.000000 | 0.000000 | NaN | NaN | 0.0000000 |
| demo_sex* | 7 | 14 | 1.5000000 | 0.5188745 | 1.5000000 | 1.5000000 | 0.7413000 | 1.00000 | 2.000000 | 1.000000 | 0.0000000 | -2.1377551 | 0.1386750 |

Summary statistics for Session 2, Baseline phase, and EOS intervention

|  | vars | n | mean | sd | median | trimmed | mad | min | max | range | skew | kurtosis | se |
| --- | --- | --- | --- | --- | --- | --- | --- | --- | --- | --- | --- | --- | --- |
| Phase* | 1 | 14 | 1.0000000 | 0.0000000 | 1.0000000 | 1.0000000 | 0.0000000 | 1.0000000 | 1.0000000 | 0.0000000 | NaN | NaN | 0.0000000 |
| alpha_asymmetry | 2 | 14 | 0.0648316 | 0.2258367 | 0.0285497 | 0.0516537 | 0.2718495 | -0.2480003 | 0.5357982 | 0.7837985 | 0.3907741 | -0.8178289 | 0.0603574 |
| duration | 3 | 14 | 302.3378905 | 0.7729210 | 302.4893255 | 302.4646050 | 0.4880623 | 300.0000000 | 303.1552070 | 3.1552070 | -1.7967883 | 3.1690167 | 0.2065718 |
| EOS_Control* | 4 | 14 | 1.0000000 | 0.0000000 | 1.0000000 | 1.0000000 | 0.0000000 | 1.0000000 | 1.0000000 | 0.0000000 | NaN | NaN | 0.0000000 |
| Subject* | 5 | 14 | 7.5000000 | 4.1833001 | 7.5000000 | 7.5000000 | 5.1891000 | 1.0000000 | 14.0000000 | 13.0000000 | 0.0000000 | -1.4585714 | 1.1180340 |
| Session* | 6 | 14 | 2.0000000 | 0.0000000 | 2.0000000 | 2.0000000 | 0.0000000 | 2.0000000 | 2.0000000 | 0.0000000 | NaN | NaN | 0.0000000 |
| demo_sex* | 7 | 14 | 1.5000000 | 0.5188745 | 1.5000000 | 1.5000000 | 0.7413000 | 1.0000000 | 2.0000000 | 1.0000000 | 0.0000000 | -2.1377551 | 0.1386750 |

Summary statistics for Session 2, Baseline phase, and Control intervention

|  | vars | n | mean | sd | median | trimmed | mad | min | max | range | skew | kurtosis | se |
| --- | --- | --- | --- | --- | --- | --- | --- | --- | --- | --- | --- | --- | --- |
| Phase* | 1 | 13 | 1.000000 | 0.0000000 | 1.0000000 | 1.0000000 | 0.0000000 | 1.0000000 | 1.0000000 | 0.000000 | NaN | NaN | 0.0000000 |
| alpha_asymmetry | 2 | 13 | 0.111753 | 0.2720597 | 0.0515026 | 0.1034131 | 0.3195469 | -0.3107137 | 0.6259593 | 0.936673 | 0.2344973 | -1.1766493 | 0.0754558 |
| duration | 3 | 13 | 302.346324 | 0.4608665 | 302.3083690 | 302.3590441 | 0.4085616 | 301.3745620 | 303.1781640 | 1.803602 | -0.2498469 | -0.4091556 | 0.1278214 |
| EOS_Control* | 4 | 13 | 1.000000 | 0.0000000 | 1.0000000 | 1.0000000 | 0.0000000 | 1.0000000 | 1.0000000 | 0.000000 | NaN | NaN | 0.0000000 |
| Subject* | 5 | 13 | 7.000000 | 3.8944405 | 7.0000000 | 7.0000000 | 4.4478000 | 1.0000000 | 13.0000000 | 12.000000 | 0.0000000 | -1.4784446 | 1.0801234 |
| Session* | 6 | 13 | 2.000000 | 0.0000000 | 2.0000000 | 2.0000000 | 0.0000000 | 2.0000000 | 2.0000000 | 0.000000 | NaN | NaN | 0.0000000 |
| demo_sex* | 7 | 13 | 1.538461 | 0.6602253 | 1.0000000 | 1.4545455 | 0.0000000 | 1.0000000 | 3.0000000 | 2.000000 | 0.6737567 | -0.8226490 | 0.1831135 |

Summary statistics for Session 1 and Baseline phase

|  | vars | n | mean | sd | median | trimmed | mad | min | max | range | skew | kurtosis | se |
| --- | --- | --- | --- | --- | --- | --- | --- | --- | --- | --- | --- | --- | --- |
| Phase* | 1 | 28 | 1.000000 | 0.0000000 | 1.000000 | 1.000000 | 0.0000000 | 1.000000 | 1.00000 | 0.00000 | NaN | NaN | 0.0000000 |
| ThBt1 | 2 | 28 | 9.101285 | 6.4790111 | 7.109128 | 8.318740 | 3.6138044 | 1.994695 | 31.47029 | 29.47559 | 1.5794374 | 2.7670203 | 1.2244180 |
| duration | 3 | 28 | 302.450767 | 0.4024055 | 302.461806 | 302.462662 | 0.4801385 | 301.440266 | 303.20965 | 1.76938 | -0.2139645 | -0.2757838 | 0.0760475 |
| EOS_Control* | 4 | 28 | 1.500000 | 0.5091751 | 1.500000 | 1.500000 | 0.7413000 | 1.000000 | 2.00000 | 1.00000 | 0.0000000 | -2.0701531 | 0.0962250 |
| Subject* | 5 | 28 | 14.500000 | 8.2259751 | 14.500000 | 14.500000 | 10.3782000 | 1.000000 | 28.00000 | 27.00000 | 0.0000000 | -1.3291256 | 1.5545632 |
| Session* | 6 | 28 | 1.000000 | 0.0000000 | 1.000000 | 1.000000 | 0.0000000 | 1.000000 | 1.00000 | 0.00000 | NaN | NaN | 0.0000000 |
| demo_sex* | 7 | 28 | 1.428571 | 0.5039526 | 1.000000 | 1.416667 | 0.0000000 | 1.000000 | 2.00000 | 1.00000 | 0.2733493 | -1.9926658 | 0.0952381 |

Summary statistics for Session 1 and MAST Instruction phase

|  | vars | n | mean | sd | median | trimmed | mad | min | max | range | skew | kurtosis | se |
| --- | --- | --- | --- | --- | --- | --- | --- | --- | --- | --- | --- | --- | --- |
| Phase* | 1 | 28 | 4.000000 | 0.0000000 | 4.00000 | 4.000000 | 0.000000 | 4.000000 | 4.00000 | 0.00000 | NaN | NaN | 0.0000000 |
| ThBt1 | 2 | 28 | 12.080090 | 8.9499333 | 10.88155 | 11.079570 | 9.220045 | 2.050244 | 44.13608 | 42.08583 | 1.5984370 | 3.352957 | 1.6913784 |
| duration | 3 | 28 | 391.427974 | 140.5399398 | 350.94255 | 366.291482 | 58.250413 | 256.944954 | 978.77628 | 721.83132 | 2.7975681 | 8.575616 | 26.5595521 |
| EOS_Control* | 4 | 28 | 1.500000 | 0.5091751 | 1.50000 | 1.500000 | 0.741300 | 1.000000 | 2.00000 | 1.00000 | 0.0000000 | -2.070153 | 0.0962250 |
| Subject* | 5 | 28 | 14.500000 | 8.2259751 | 14.50000 | 14.500000 | 10.378200 | 1.000000 | 28.00000 | 27.00000 | 0.0000000 | -1.329126 | 1.5545632 |
| Session* | 6 | 28 | 1.000000 | 0.0000000 | 1.00000 | 1.000000 | 0.000000 | 1.000000 | 1.00000 | 0.00000 | NaN | NaN | 0.0000000 |
| demo_sex* | 7 | 28 | 1.428571 | 0.5039526 | 1.00000 | 1.416667 | 0.000000 | 1.000000 | 2.00000 | 1.00000 | 0.2733493 | -1.992666 | 0.0952381 |

Summary statistics for Session 1, Baseline phase, and Women

|  | vars | n | mean | sd | median | trimmed | mad | min | max | range | skew | kurtosis | se |
| --- | --- | --- | --- | --- | --- | --- | --- | --- | --- | --- | --- | --- | --- |
| Phase* | 1 | 16 | 1.000000 | 0.0000000 | 1.000000 | 1.000000 | 0.0000000 | 1.000000 | 1.00000 | 0.00000 | NaN | NaN | 0.0000000 |
| ThBt1 | 2 | 16 | 8.550123 | 5.2906563 | 7.092225 | 8.220053 | 4.6759732 | 2.081559 | 19.63967 | 17.55811 | 0.6497217 | -0.8496352 | 1.3226641 |
| duration | 3 | 16 | 302.315870 | 0.4325407 | 302.229017 | 302.314572 | 0.3451315 | 301.440266 | 303.20965 | 1.76938 | 0.2360098 | -0.1427719 | 0.1081352 |
| EOS_Control* | 4 | 16 | 1.437500 | 0.5123475 | 1.000000 | 1.428571 | 0.0000000 | 1.000000 | 2.00000 | 1.00000 | 0.2287266 | -2.0652902 | 0.1280869 |
| Subject* | 5 | 16 | 8.500000 | 4.7609523 | 8.500000 | 8.500000 | 5.9304000 | 1.000000 | 16.00000 | 15.00000 | 0.0000000 | -1.4262408 | 1.1902381 |
| Session* | 6 | 16 | 1.000000 | 0.0000000 | 1.000000 | 1.000000 | 0.0000000 | 1.000000 | 1.00000 | 0.00000 | NaN | NaN | 0.0000000 |
| demo_sex* | 7 | 16 | 1.000000 | 0.0000000 | 1.000000 | 1.000000 | 0.0000000 | 1.000000 | 1.00000 | 0.00000 | NaN | NaN | 0.0000000 |

Summary statistics for Session 1, MAST Instruction phase, and Women

|  | vars | n | mean | sd | median | trimmed | mad | min | max | range | skew | kurtosis | se |
| --- | --- | --- | --- | --- | --- | --- | --- | --- | --- | --- | --- | --- | --- |
| Phase* | 1 | 16 | 4.00000 | 0.0000000 | 4.0000 | 4.000000 | 0.000000 | 4.000000 | 4.00000 | 0.00000 | NaN | NaN | 0.0000000 |
| ThBt1 | 2 | 16 | 13.92467 | 10.1502618 | 11.8248 | 12.549473 | 9.022028 | 2.966046 | 44.13608 | 41.17003 | 1.4873726 | 2.176311 | 2.5375655 |
| duration | 3 | 16 | 399.74741 | 166.0621977 | 350.9426 | 368.588385 | 87.235585 | 256.944954 | 978.77628 | 721.83132 | 2.5458051 | 6.321374 | 41.5155494 |
| EOS_Control* | 4 | 16 | 1.43750 | 0.5123475 | 1.0000 | 1.428571 | 0.000000 | 1.000000 | 2.00000 | 1.00000 | 0.2287266 | -2.065290 | 0.1280869 |
| Subject* | 5 | 16 | 8.50000 | 4.7609523 | 8.5000 | 8.500000 | 5.930400 | 1.000000 | 16.00000 | 15.00000 | 0.0000000 | -1.426241 | 1.1902381 |
| Session* | 6 | 16 | 1.00000 | 0.0000000 | 1.0000 | 1.000000 | 0.000000 | 1.000000 | 1.00000 | 0.00000 | NaN | NaN | 0.0000000 |
| demo_sex* | 7 | 16 | 1.00000 | 0.0000000 | 1.0000 | 1.000000 | 0.000000 | 1.000000 | 1.00000 | 0.00000 | NaN | NaN | 0.0000000 |

Summary statistics for Session 1, Baseline phase, and Men

|  | vars | n | mean | sd | median | trimmed | mad | min | max | range | skew | kurtosis | se |
| --- | --- | --- | --- | --- | --- | --- | --- | --- | --- | --- | --- | --- | --- |
| Phase* | 1 | 12 | 1.000000 | 0.0000000 | 1.000000 | 1.000000 | 0.0000000 | 1.000000 | 1.00000 | 0.000000 | NaN | NaN | 0.0000000 |
| ThBt1 | 2 | 12 | 9.836166 | 7.9896982 | 7.546542 | 8.456902 | 2.7003020 | 1.994695 | 31.47029 | 29.475590 | 1.5738468 | 1.695721 | 2.3064272 |
| duration | 3 | 12 | 302.630631 | 0.2838659 | 302.630902 | 302.644311 | 0.3458179 | 302.135578 | 302.98888 | 0.853306 | -0.2516703 | -1.483071 | 0.0819450 |
| EOS_Control* | 4 | 12 | 1.583333 | 0.5149287 | 2.000000 | 1.600000 | 0.0000000 | 1.000000 | 2.00000 | 1.000000 | -0.2966970 | -2.063690 | 0.1486471 |
| Subject* | 5 | 12 | 6.500000 | 3.6055513 | 6.500000 | 6.500000 | 4.4478000 | 1.000000 | 12.00000 | 11.000000 | 0.0000000 | -1.501603 | 1.0408330 |
| Session* | 6 | 12 | 1.000000 | 0.0000000 | 1.000000 | 1.000000 | 0.0000000 | 1.000000 | 1.00000 | 0.000000 | NaN | NaN | 0.0000000 |
| demo_sex* | 7 | 12 | 2.000000 | 0.0000000 | 2.000000 | 2.000000 | 0.0000000 | 2.000000 | 2.00000 | 0.000000 | NaN | NaN | 0.0000000 |

Summary statistics for Session 1, MAST Instruction phase, and Men

|  | vars | n | mean | sd | median | trimmed | mad | min | max | range | skew | kurtosis | se |
| --- | --- | --- | --- | --- | --- | --- | --- | --- | --- | --- | --- | --- | --- |
| Phase* | 1 | 12 | 4.000000 | 0.0000000 | 4.000000 | 4.000000 | 0.000000 | 4.000000 | 4.00000 | 0.00000 | NaN | NaN | 0.0000000 |
| ThBt1 | 2 | 12 | 9.620648 | 6.6762271 | 8.801557 | 9.009312 | 7.058309 | 2.050244 | 23.30441 | 21.25417 | 0.6560189 | -0.8327831 | 1.9272608 |
| duration | 3 | 12 | 380.335387 | 103.1574520 | 345.860124 | 358.136029 | 47.028537 | 307.761442 | 674.90290 | 367.14146 | 1.8586594 | 2.6274442 | 29.7789913 |
| EOS_Control* | 4 | 12 | 1.583333 | 0.5149287 | 2.000000 | 1.600000 | 0.000000 | 1.000000 | 2.00000 | 1.00000 | -0.2966970 | -2.0636905 | 0.1486471 |
| Subject* | 5 | 12 | 6.500000 | 3.6055513 | 6.500000 | 6.500000 | 4.447800 | 1.000000 | 12.00000 | 11.00000 | 0.0000000 | -1.5016026 | 1.0408330 |
| Session* | 6 | 12 | 1.000000 | 0.0000000 | 1.000000 | 1.000000 | 0.000000 | 1.000000 | 1.00000 | 0.00000 | NaN | NaN | 0.0000000 |
| demo_sex* | 7 | 12 | 2.000000 | 0.0000000 | 2.000000 | 2.000000 | 0.000000 | 2.000000 | 2.00000 | 0.00000 | NaN | NaN | 0.0000000 |

Summary statistics for Session 1, Baseline phase, and EOS intervention

|  | vars | n | mean | sd | median | trimmed | mad | min | max | range | skew | kurtosis | se |
| --- | --- | --- | --- | --- | --- | --- | --- | --- | --- | --- | --- | --- | --- |
| Phase* | 1 | 14 | 1.000000 | 0.0000000 | 1.000000 | 1.000000 | 0.0000000 | 1.000000 | 1.00000 | 0.00000 | NaN | NaN | 0.0000000 |
| ThBt1 | 2 | 14 | 7.027446 | 4.1590477 | 6.660075 | 6.553346 | 2.5961551 | 2.081559 | 17.66254 | 15.58098 | 1.0972072 | 0.6199378 | 1.1115522 |
| duration | 3 | 14 | 302.476511 | 0.3657413 | 302.429954 | 302.449359 | 0.4426399 | 302.069196 | 303.20965 | 1.14045 | 0.5761561 | -1.1361841 | 0.0977485 |
| EOS_Control* | 4 | 14 | 1.000000 | 0.0000000 | 1.000000 | 1.000000 | 0.0000000 | 1.000000 | 1.00000 | 0.00000 | NaN | NaN | 0.0000000 |
| Subject* | 5 | 14 | 7.500000 | 4.1833001 | 7.500000 | 7.500000 | 5.1891000 | 1.000000 | 14.00000 | 13.00000 | 0.0000000 | -1.4585714 | 1.1180340 |
| Session* | 6 | 14 | 1.000000 | 0.0000000 | 1.000000 | 1.000000 | 0.0000000 | 1.000000 | 1.00000 | 0.00000 | NaN | NaN | 0.0000000 |
| demo_sex* | 7 | 14 | 1.357143 | 0.4972452 | 1.000000 | 1.333333 | 0.0000000 | 1.000000 | 2.00000 | 1.00000 | 0.5335519 | -1.8311791 | 0.1328944 |

Summary statistics for Session 1, Baseline phase, and Control intervention

|  | vars | n | mean | sd | median | trimmed | mad | min | max | range | skew | kurtosis | se |
| --- | --- | --- | --- | --- | --- | --- | --- | --- | --- | --- | --- | --- | --- |
| Phase* | 1 | 14 | 1.00000 | 0.0000000 | 1.00000 | 1.0000 | 0.0000000 | 1.000000 | 1.00000 | 0.000000 | NaN | NaN | 0.0000000 |
| ThBt1 | 2 | 14 | 11.17512 | 7.7860940 | 8.80929 | 10.2489 | 6.0555930 | 1.994695 | 31.47029 | 29.475590 | 1.122620 | 0.6994705 | 2.0809212 |
| duration | 3 | 14 | 302.42502 | 0.4484668 | 302.51513 | 302.4550 | 0.4520225 | 301.440266 | 303.05030 | 1.610029 | -0.568114 | -0.5253899 | 0.1198578 |
| EOS_Control* | 4 | 14 | 1.00000 | 0.0000000 | 1.00000 | 1.0000 | 0.0000000 | 1.000000 | 1.00000 | 0.000000 | NaN | NaN | 0.0000000 |
| Subject* | 5 | 14 | 7.50000 | 4.1833001 | 7.50000 | 7.5000 | 5.1891000 | 1.000000 | 14.00000 | 13.000000 | 0.000000 | -1.4585714 | 1.1180340 |
| Session* | 6 | 14 | 1.00000 | 0.0000000 | 1.00000 | 1.0000 | 0.0000000 | 1.000000 | 1.00000 | 0.000000 | NaN | NaN | 0.0000000 |
| demo_sex* | 7 | 14 | 1.50000 | 0.5188745 | 1.50000 | 1.5000 | 0.7413000 | 1.000000 | 2.00000 | 1.000000 | 0.000000 | -2.1377551 | 0.1386750 |

Summary statistics for Session 2, Baseline phase, and EOS intervention

|  | vars | n | mean | sd | median | trimmed | mad | min | max | range | skew | kurtosis | se |
| --- | --- | --- | --- | --- | --- | --- | --- | --- | --- | --- | --- | --- | --- |
| Phase* | 1 | 14 | 1.000000 | 0.0000000 | 1.00000 | 1.000000 | 0.0000000 | 1.000000 | 1.00000 | 0.000000 | NaN | NaN | 0.0000000 |
| ThBt1 | 2 | 14 | 7.496142 | 4.4084730 | 6.87167 | 7.133951 | 2.7152667 | 1.167038 | 18.17154 | 17.004498 | 1.012941 | 0.3670973 | 1.1782140 |
| duration | 3 | 14 | 302.337891 | 0.7729210 | 302.48933 | 302.464605 | 0.4880623 | 300.000000 | 303.15521 | 3.155207 | -1.796788 | 3.1690167 | 0.2065718 |
| EOS_Control* | 4 | 14 | 1.000000 | 0.0000000 | 1.00000 | 1.000000 | 0.0000000 | 1.000000 | 1.00000 | 0.000000 | NaN | NaN | 0.0000000 |
| Subject* | 5 | 14 | 7.500000 | 4.1833001 | 7.50000 | 7.500000 | 5.1891000 | 1.000000 | 14.00000 | 13.000000 | 0.000000 | -1.4585714 | 1.1180340 |
| Session* | 6 | 14 | 2.000000 | 0.0000000 | 2.00000 | 2.000000 | 0.0000000 | 2.000000 | 2.00000 | 0.000000 | NaN | NaN | 0.0000000 |
| demo_sex* | 7 | 14 | 1.500000 | 0.5188745 | 1.50000 | 1.500000 | 0.7413000 | 1.000000 | 2.00000 | 1.000000 | 0.000000 | -2.1377551 | 0.1386750 |

Summary statistics for Session 2, Baseline phase, and Control intervention

|  | vars | n | mean | sd | median | trimmed | mad | min | max | range | skew | kurtosis | se |
| --- | --- | --- | --- | --- | --- | --- | --- | --- | --- | --- | --- | --- | --- |
| Phase* | 1 | 13 | 1.000000 | 0.0000000 | 1.000000 | 1.000000 | 0.0000000 | 1.000000 | 1.00000 | 0.000000 | NaN | NaN | 0.0000000 |
| ThBt1 | 2 | 13 | 5.311129 | 2.7624486 | 4.574096 | 5.040173 | 2.1101785 | 1.990954 | 11.61182 | 9.620867 | 0.8526489 | -0.3502027 | 0.7661654 |
| duration | 3 | 13 | 302.346324 | 0.4608665 | 302.308369 | 302.359044 | 0.4085616 | 301.374562 | 303.17816 | 1.803602 | -0.2498469 | -0.4091556 | 0.1278214 |
| EOS_Control* | 4 | 13 | 1.000000 | 0.0000000 | 1.000000 | 1.000000 | 0.0000000 | 1.000000 | 1.00000 | 0.000000 | NaN | NaN | 0.0000000 |
| Subject* | 5 | 13 | 7.000000 | 3.8944405 | 7.000000 | 7.000000 | 4.4478000 | 1.000000 | 13.00000 | 12.000000 | 0.0000000 | -1.4784446 | 1.0801234 |
| Session* | 6 | 13 | 2.000000 | 0.0000000 | 2.000000 | 2.000000 | 0.0000000 | 2.000000 | 2.00000 | 0.000000 | NaN | NaN | 0.0000000 |
| demo_sex* | 7 | 13 | 1.538461 | 0.6602253 | 1.000000 | 1.454546 | 0.0000000 | 1.000000 | 3.00000 | 2.000000 | 0.6737567 | -0.8226490 | 0.1831135 |

Summary statistics for Session 1 and Baseline phase

|  | vars | n | mean | sd | median | trimmed | mad | min | max | range | skew | kurtosis | se |
| --- | --- | --- | --- | --- | --- | --- | --- | --- | --- | --- | --- | --- | --- |
| Phase* | 1 | 28 | 1.000000 | 0.0000000 | 1.000000 | 1.000000 | 0.0000000 | 1.000000 | 1.00000 | 0.00000 | NaN | NaN | 0.0000000 |
| ThBt2 | 2 | 28 | 9.041317 | 6.8001735 | 7.834803 | 8.087396 | 3.4275389 | 2.066457 | 35.76402 | 33.69757 | 2.2200974 | 5.9328844 | 1.2851120 |
| duration | 3 | 28 | 302.450767 | 0.4024055 | 302.461806 | 302.462662 | 0.4801385 | 301.440266 | 303.20965 | 1.76938 | -0.2139645 | -0.2757838 | 0.0760475 |
| EOS_Control* | 4 | 28 | 1.500000 | 0.5091751 | 1.500000 | 1.500000 | 0.7413000 | 1.000000 | 2.00000 | 1.00000 | 0.0000000 | -2.0701531 | 0.0962250 |
| Subject* | 5 | 28 | 14.500000 | 8.2259751 | 14.500000 | 14.500000 | 10.3782000 | 1.000000 | 28.00000 | 27.00000 | 0.0000000 | -1.3291256 | 1.5545632 |
| Session* | 6 | 28 | 1.000000 | 0.0000000 | 1.000000 | 1.000000 | 0.0000000 | 1.000000 | 1.00000 | 0.00000 | NaN | NaN | 0.0000000 |
| demo_sex* | 7 | 28 | 1.428571 | 0.5039526 | 1.000000 | 1.416667 | 0.0000000 | 1.000000 | 2.00000 | 1.00000 | 0.2733493 | -1.9926658 | 0.0952381 |

Summary statistics for Session 1 and MAST Instruction phase

|  | vars | n | mean | sd | median | trimmed | mad | min | max | range | skew | kurtosis | se |
| --- | --- | --- | --- | --- | --- | --- | --- | --- | --- | --- | --- | --- | --- |
| Phase* | 1 | 28 | 4.000000 | 0.0000000 | 4.000000 | 4.000000 | 0.000000 | 4.000000 | 4.00000 | 0.00000 | NaN | NaN | 0.0000000 |
| ThBt2 | 2 | 28 | 13.328507 | 12.5900041 | 9.126606 | 11.329687 | 7.378884 | 2.725678 | 51.91112 | 49.18544 | 1.7608822 | 2.205590 | 2.3792871 |
| duration | 3 | 28 | 391.427974 | 140.5399398 | 350.942553 | 366.291482 | 58.250413 | 256.944954 | 978.77628 | 721.83132 | 2.7975681 | 8.575616 | 26.5595521 |
| EOS_Control* | 4 | 28 | 1.500000 | 0.5091751 | 1.500000 | 1.500000 | 0.741300 | 1.000000 | 2.00000 | 1.00000 | 0.0000000 | -2.070153 | 0.0962250 |
| Subject* | 5 | 28 | 14.500000 | 8.2259751 | 14.500000 | 14.500000 | 10.378200 | 1.000000 | 28.00000 | 27.00000 | 0.0000000 | -1.329126 | 1.5545632 |
| Session* | 6 | 28 | 1.000000 | 0.0000000 | 1.000000 | 1.000000 | 0.000000 | 1.000000 | 1.00000 | 0.00000 | NaN | NaN | 0.0000000 |
| demo_sex* | 7 | 28 | 1.428571 | 0.5039526 | 1.000000 | 1.416667 | 0.000000 | 1.000000 | 2.00000 | 1.00000 | 0.2733493 | -1.992666 | 0.0952381 |

Summary statistics for Session 1, Baseline phase, and Women

|  | vars | n | mean | sd | median | trimmed | mad | min | max | range | skew | kurtosis | se |
| --- | --- | --- | --- | --- | --- | --- | --- | --- | --- | --- | --- | --- | --- |
| Phase* | 1 | 16 | 1.000000 | 0.0000000 | 1.00000 | 1.000000 | 0.0000000 | 1.000000 | 1.0000 | 0.00000 | NaN | NaN | 0.0000000 |
| ThBt2 | 2 | 16 | 8.278038 | 4.3772490 | 8.09604 | 7.992189 | 3.5896669 | 2.066457 | 18.4915 | 16.42505 | 0.7129731 | -0.2586678 | 1.0943122 |
| duration | 3 | 16 | 302.315870 | 0.4325407 | 302.22902 | 302.314572 | 0.3451315 | 301.440266 | 303.2096 | 1.76938 | 0.2360098 | -0.1427719 | 0.1081352 |
| EOS_Control* | 4 | 16 | 1.437500 | 0.5123475 | 1.00000 | 1.428571 | 0.0000000 | 1.000000 | 2.0000 | 1.00000 | 0.2287266 | -2.0652902 | 0.1280869 |
| Subject* | 5 | 16 | 8.500000 | 4.7609523 | 8.50000 | 8.500000 | 5.9304000 | 1.000000 | 16.0000 | 15.00000 | 0.0000000 | -1.4262408 | 1.1902381 |
| Session* | 6 | 16 | 1.000000 | 0.0000000 | 1.00000 | 1.000000 | 0.0000000 | 1.000000 | 1.0000 | 0.00000 | NaN | NaN | 0.0000000 |
| demo_sex* | 7 | 16 | 1.000000 | 0.0000000 | 1.00000 | 1.000000 | 0.0000000 | 1.000000 | 1.0000 | 0.00000 | NaN | NaN | 0.0000000 |

Summary statistics for Session 1, MAST Instruction phase, and Women

|  | vars | n | mean | sd | median | trimmed | mad | min | max | range | skew | kurtosis | se |
| --- | --- | --- | --- | --- | --- | --- | --- | --- | --- | --- | --- | --- | --- |
| Phase* | 1 | 16 | 4.00000 | 0.0000000 | 4.000000 | 4.000000 | 0.000000 | 4.000000 | 4.00000 | 0.00000 | NaN | NaN | 0.0000000 |
| ThBt2 | 2 | 16 | 13.73956 | 11.9237723 | 9.145077 | 12.394386 | 4.644557 | 2.725678 | 43.58591 | 40.86023 | 1.5353798 | 1.063459 | 2.9809431 |
| duration | 3 | 16 | 399.74741 | 166.0621977 | 350.942553 | 368.588385 | 87.235585 | 256.944954 | 978.77628 | 721.83132 | 2.5458051 | 6.321374 | 41.5155494 |
| EOS_Control* | 4 | 16 | 1.43750 | 0.5123475 | 1.000000 | 1.428571 | 0.000000 | 1.000000 | 2.00000 | 1.00000 | 0.2287266 | -2.065290 | 0.1280869 |
| Subject* | 5 | 16 | 8.50000 | 4.7609523 | 8.500000 | 8.500000 | 5.930400 | 1.000000 | 16.00000 | 15.00000 | 0.0000000 | -1.426241 | 1.1902381 |
| Session* | 6 | 16 | 1.00000 | 0.0000000 | 1.000000 | 1.000000 | 0.000000 | 1.000000 | 1.00000 | 0.00000 | NaN | NaN | 0.0000000 |
| demo_sex* | 7 | 16 | 1.00000 | 0.0000000 | 1.000000 | 1.000000 | 0.000000 | 1.000000 | 1.00000 | 0.00000 | NaN | NaN | 0.0000000 |

```
## Warning in FUN(newX[, i], ...): no non-missing arguments to min; returning Inf
## Warning in FUN(newX[, i], ...): no non-missing arguments to max; returning -Inf
```

Summary statistics for Session 1, MAST Task phase, and Women

|  | vars | n | mean | sd | median | trimmed | mad | min | max | range | skew | kurtosis | se |
| --- | --- | --- | --- | --- | --- | --- | --- | --- | --- | --- | --- | --- | --- |
| Phase* | 1 | 16 | 5.0000 | 0.0000000 | 5.0000 | 5.000000 | 0.0000000 | 5.0000 | 5.0000 | 0.000000 | NaN | NaN | 0.0000000 |
| ThBt2 | 2 | 0 | NaN | NA | NA | NaN | NA | Inf | -Inf | -Inf | NA | NA | NA |
| duration | 3 | 16 | 603.8384 | 0.7596899 | 603.4902 | 603.791565 | 0.8304695 | 602.8931 | 605.4392 | 2.546146 | 0.5096873 | -1.033431 | 0.1899225 |
| EOS_Control* | 4 | 16 | 1.4375 | 0.5123475 | 1.0000 | 1.428571 | 0.0000000 | 1.0000 | 2.0000 | 1.000000 | 0.2287266 | -2.065290 | 0.1280869 |
| Subject* | 5 | 16 | 8.5000 | 4.7609523 | 8.5000 | 8.500000 | 5.9304000 | 1.0000 | 16.0000 | 15.000000 | 0.0000000 | -1.426241 | 1.1902381 |
| Session* | 6 | 16 | 1.0000 | 0.0000000 | 1.0000 | 1.000000 | 0.0000000 | 1.0000 | 1.0000 | 0.000000 | NaN | NaN | 0.0000000 |
| demo_sex* | 7 | 16 | 1.0000 | 0.0000000 | 1.0000 | 1.000000 | 0.0000000 | 1.0000 | 1.0000 | 0.000000 | NaN | NaN | 0.0000000 |

Summary statistics for Session 1, Baseline phase, and Men

|  | vars | n | mean | sd | median | trimmed | mad | min | max | range | skew | kurtosis | se |
| --- | --- | --- | --- | --- | --- | --- | --- | --- | --- | --- | --- | --- | --- |
| Phase* | 1 | 12 | 1.000000 | 0.0000000 | 1.000000 | 1.000000 | 0.0000000 | 1.000000 | 1.00000 | 0.000000 | NaN | NaN | 0.0000000 |
| ThBt2 | 2 | 12 | 10.059022 | 9.2411574 | 6.829853 | 8.220686 | 3.9582879 | 2.737386 | 35.76402 | 33.026638 | 1.7291259 | 2.120727 | 2.6676924 |
| duration | 3 | 12 | 302.630631 | 0.2838659 | 302.630902 | 302.644311 | 0.3458179 | 302.135578 | 302.98888 | 0.853306 | -0.2516703 | -1.483071 | 0.0819450 |
| EOS_Control* | 4 | 12 | 1.583333 | 0.5149287 | 2.000000 | 1.600000 | 0.0000000 | 1.000000 | 2.00000 | 1.000000 | -0.2966970 | -2.063690 | 0.1486471 |
| Subject* | 5 | 12 | 6.500000 | 3.6055513 | 6.500000 | 6.500000 | 4.4478000 | 1.000000 | 12.00000 | 11.000000 | 0.0000000 | -1.501603 | 1.0408330 |
| Session* | 6 | 12 | 1.000000 | 0.0000000 | 1.000000 | 1.000000 | 0.0000000 | 1.000000 | 1.00000 | 0.000000 | NaN | NaN | 0.0000000 |
| demo_sex* | 7 | 12 | 2.000000 | 0.0000000 | 2.000000 | 2.000000 | 0.0000000 | 2.000000 | 2.00000 | 0.000000 | NaN | NaN | 0.0000000 |

Summary statistics for Session 1, MAST Instruction phase, and Men

|  | vars | n | mean | sd | median | trimmed | mad | min | max | range | skew | kurtosis | se |
| --- | --- | --- | --- | --- | --- | --- | --- | --- | --- | --- | --- | --- | --- |
| Phase* | 1 | 12 | 4.000000 | 0.0000000 | 4.000000 | 4.000000 | 0.000000 | 4.000000 | 4.00000 | 0.0000 | NaN | NaN | 0.0000000 |
| ThBt2 | 2 | 12 | 12.780435 | 13.9504585 | 9.062386 | 9.839108 | 8.299505 | 3.063016 | 51.91112 | 48.8481 | 1.769137 | 2.279950 | 4.0271505 |
| duration | 3 | 12 | 380.335387 | 103.1574520 | 345.860124 | 358.136029 | 47.028537 | 307.761442 | 674.90290 | 367.1415 | 1.858659 | 2.627444 | 29.7789913 |
| EOS_Control* | 4 | 12 | 1.583333 | 0.5149287 | 2.000000 | 1.600000 | 0.000000 | 1.000000 | 2.00000 | 1.0000 | -0.296697 | -2.063690 | 0.1486471 |
| Subject* | 5 | 12 | 6.500000 | 3.6055513 | 6.500000 | 6.500000 | 4.447800 | 1.000000 | 12.00000 | 11.0000 | 0.000000 | -1.501603 | 1.0408330 |
| Session* | 6 | 12 | 1.000000 | 0.0000000 | 1.000000 | 1.000000 | 0.000000 | 1.000000 | 1.00000 | 0.0000 | NaN | NaN | 0.0000000 |
| demo_sex* | 7 | 12 | 2.000000 | 0.0000000 | 2.000000 | 2.000000 | 0.000000 | 2.000000 | 2.00000 | 0.0000 | NaN | NaN | 0.0000000 |

Summary statistics for Session 1, Baseline phase, and EOS intervention

|  | vars | n | mean | sd | median | trimmed | mad | min | max | range | skew | kurtosis | se |
| --- | --- | --- | --- | --- | --- | --- | --- | --- | --- | --- | --- | --- | --- |
| Phase* | 1 | 14 | 1.000000 | 0.0000000 | 1.000000 | 1.000000 | 0.0000000 | 1.000000 | 1.00000 | 0.00000 | NaN | NaN | 0.0000000 |
| ThBt2 | 2 | 14 | 7.230800 | 3.4609723 | 5.909057 | 7.089072 | 3.9219380 | 2.066457 | 14.09589 | 12.02943 | 0.3499540 | -0.9964177 | 0.9249838 |
| duration | 3 | 14 | 302.476511 | 0.3657413 | 302.429954 | 302.449359 | 0.4426399 | 302.069196 | 303.20965 | 1.14045 | 0.5761561 | -1.1361841 | 0.0977485 |
| EOS_Control* | 4 | 14 | 1.000000 | 0.0000000 | 1.000000 | 1.000000 | 0.0000000 | 1.000000 | 1.00000 | 0.00000 | NaN | NaN | 0.0000000 |
| Subject* | 5 | 14 | 7.500000 | 4.1833001 | 7.500000 | 7.500000 | 5.1891000 | 1.000000 | 14.00000 | 13.00000 | 0.0000000 | -1.4585714 | 1.1180340 |
| Session* | 6 | 14 | 1.000000 | 0.0000000 | 1.000000 | 1.000000 | 0.0000000 | 1.000000 | 1.00000 | 0.00000 | NaN | NaN | 0.0000000 |
| demo_sex* | 7 | 14 | 1.357143 | 0.4972452 | 1.000000 | 1.333333 | 0.0000000 | 1.000000 | 2.00000 | 1.00000 | 0.5335519 | -1.8311791 | 0.1328944 |

Summary statistics for Session 1, Baseline phase, and Control intervention

|  | vars | n | mean | sd | median | trimmed | mad | min | max | range | skew | kurtosis | se |
| --- | --- | --- | --- | --- | --- | --- | --- | --- | --- | --- | --- | --- | --- |
| Phase* | 1 | 14 | 1.00000 | 0.0000000 | 1.000000 | 1.000000 | 0.0000000 | 1.00000 | 1.00000 | 0.000000 | NaN | NaN | 0.0000000 |
| ThBt2 | 2 | 14 | 10.85183 | 8.7751495 | 8.374383 | 9.396125 | 6.2643790 | 3.40816 | 35.76402 | 32.355863 | 1.559503 | 1.8474339 | 2.3452574 |
| duration | 3 | 14 | 302.42502 | 0.4484668 | 302.515134 | 302.454981 | 0.4520225 | 301.44027 | 303.05030 | 1.610029 | -0.568114 | -0.5253899 | 0.1198578 |
| EOS_Control* | 4 | 14 | 1.00000 | 0.0000000 | 1.000000 | 1.000000 | 0.0000000 | 1.00000 | 1.00000 | 0.000000 | NaN | NaN | 0.0000000 |
| Subject* | 5 | 14 | 7.50000 | 4.1833001 | 7.500000 | 7.500000 | 5.1891000 | 1.00000 | 14.00000 | 13.000000 | 0.000000 | -1.4585714 | 1.1180340 |
| Session* | 6 | 14 | 1.00000 | 0.0000000 | 1.000000 | 1.000000 | 0.0000000 | 1.00000 | 1.00000 | 0.000000 | NaN | NaN | 0.0000000 |
| demo_sex* | 7 | 14 | 1.50000 | 0.5188745 | 1.500000 | 1.500000 | 0.7413000 | 1.00000 | 2.00000 | 1.000000 | 0.000000 | -2.1377551 | 0.1386750 |

Summary statistics for Session 2, Baseline phase, and EOS intervention

|  | vars | n | mean | sd | median | trimmed | mad | min | max | range | skew | kurtosis | se |
| --- | --- | --- | --- | --- | --- | --- | --- | --- | --- | --- | --- | --- | --- |
| Phase* | 1 | 14 | 1.000000 | 0.0000000 | 1.000000 | 1.000000 | 0.0000000 | 1.000000 | 1.00000 | 0.000000 | NaN | NaN | 0.0000000 |
| ThBt2 | 2 | 14 | 8.569701 | 5.3830257 | 7.055614 | 8.257592 | 3.9770062 | 1.697933 | 19.18678 | 17.488842 | 0.6202343 | -0.9864085 | 1.4386741 |
| duration | 3 | 14 | 302.337891 | 0.7729210 | 302.489326 | 302.464605 | 0.4880623 | 300.000000 | 303.15521 | 3.155207 | -1.7967883 | 3.1690167 | 0.2065718 |
| EOS_Control* | 4 | 14 | 1.000000 | 0.0000000 | 1.000000 | 1.000000 | 0.0000000 | 1.000000 | 1.00000 | 0.000000 | NaN | NaN | 0.0000000 |
| Subject* | 5 | 14 | 7.500000 | 4.1833001 | 7.500000 | 7.500000 | 5.1891000 | 1.000000 | 14.00000 | 13.000000 | 0.0000000 | -1.4585714 | 1.1180340 |
| Session* | 6 | 14 | 2.000000 | 0.0000000 | 2.000000 | 2.000000 | 0.0000000 | 2.000000 | 2.00000 | 0.000000 | NaN | NaN | 0.0000000 |
| demo_sex* | 7 | 14 | 1.500000 | 0.5188745 | 1.500000 | 1.500000 | 0.7413000 | 1.000000 | 2.00000 | 1.000000 | 0.0000000 | -2.1377551 | 0.1386750 |

Summary statistics for Session 2, Baseline phase, and Control intervention

|  | vars | n | mean | sd | median | trimmed | mad | min | max | range | skew | kurtosis | se |
| --- | --- | --- | --- | --- | --- | --- | --- | --- | --- | --- | --- | --- | --- |
| Phase* | 1 | 13 | 1.000000 | 0.0000000 | 1.000000 | 1.000000 | 0.0000000 | 1.000000 | 1.000000 | 0.000000 | NaN | NaN | 0.0000000 |
| ThBt2 | 2 | 13 | 6.199040 | 2.6563247 | 7.768712 | 6.226455 | 2.6683919 | 2.528002 | 9.568518 | 7.040516 | -0.2571522 | -1.7715099 | 0.7367319 |
| duration | 3 | 13 | 302.346324 | 0.4608665 | 302.308369 | 302.359044 | 0.4085616 | 301.374562 | 303.178164 | 1.803602 | -0.2498469 | -0.4091556 | 0.1278214 |
| EOS_Control* | 4 | 13 | 1.000000 | 0.0000000 | 1.000000 | 1.000000 | 0.0000000 | 1.000000 | 1.000000 | 0.000000 | NaN | NaN | 0.0000000 |
| Subject* | 5 | 13 | 7.000000 | 3.8944405 | 7.000000 | 7.000000 | 4.4478000 | 1.000000 | 13.000000 | 12.000000 | 0.0000000 | -1.4784446 | 1.0801234 |
| Session* | 6 | 13 | 2.000000 | 0.0000000 | 2.000000 | 2.000000 | 0.0000000 | 2.000000 | 2.000000 | 0.000000 | NaN | NaN | 0.0000000 |
| demo_sex* | 7 | 13 | 1.538461 | 0.6602253 | 1.000000 | 1.454546 | 0.0000000 | 1.000000 | 3.000000 | 2.000000 | 0.6737567 | -0.8226490 | 0.1831135 |

## 4. Study assumptions

Randomized cross-over studies are conditioned by the assumption of the absence of carry-over effects and proper randomization. To explore these potential effects, we will test differences in mean values for each variable in the pre-intervention phases (baseline and stress task) between sessions (carry-over effect), and between groups (intervention first and control first groups).

### 4.1. Subjective stress

#### 4.1.1. EOS x Control

```
## # A tibble: 4 × 6
##   EOS_Control TIME        variable     n  mean    sd
##   <chr>       <fct>       <fct>    <dbl> <dbl> <dbl>
## 1 A           Baseline    Stress      28  3.61  1.75
## 2 B           Baseline    Stress      26  3.38  1.98
## 3 A           Post-stress Stress      28  4.21  1.66
## 4 B           Post-stress Stress      26  4.88  2.05
## Warning: Removed 2 rows containing non-finite outside the scale range
## (`stat_boxplot()`).
## Removed 2 rows containing non-finite outside the scale range
## (`stat_boxplot()`).
## Warning: Removed 2 rows containing non-finite outside the scale range
## (`stat_summary()`).
## Notch went outside hinges
## ℹ Do you want `notch = FALSE`?
```

```
## [1] EOS_Control TIME        Subject     Session     Stress      is.outlier
## [7] is.extreme
## <0 rows> (or 0-length row.names)
## # A tibble: 4 × 5
##   EOS_Control TIME        variable statistic       p
##   <chr>       <fct>       <chr>        <dbl>   <dbl>
## 1 A           Baseline    Stress       0.941 0.115
## 2 A           Post-stress Stress       0.948 0.173
## 3 B           Baseline    Stress       0.863 0.00262
## 4 B           Post-stress Stress       0.947 0.196
## Warning: Removed 2 rows containing non-finite outside the scale range
## (`stat_qq()`).
## Warning: Removed 2 rows containing non-finite outside the scale range
## (`stat_qq_line()`).
## Removed 2 rows containing non-finite outside the scale range
## (`stat_qq_line()`).
```

```
## Warning: NA detected in rows: 15,70.
## Removing this rows before the analysis.
## ANOVA Table (type III tests)
##
##             Effect DFn DFd      F        p p<.05   ges
## 1      EOS_Control   1  25  0.368 0.550000       0.002
## 2             TIME   1  25 14.294 0.000868     * 0.066
## 3 EOS_Control:TIME   1  25  4.903 0.036000     * 0.020
## Warning: There were 2 warnings in `mutate()`.
## The first warning was:
## ℹ In argument: `data = map(.data$data, .f, ...)`.
## Caused by warning:
## ! NA detected in rows: 15.
## Removing this rows before the analysis.
## ℹ Run `dplyr::last_dplyr_warnings()` to see the 1 remaining warning.
##          TIME      Effect DFn DFd     F     p p<.05   ges p.adj
## 1    Baseline EOS_Control   1  25 0.928 0.344       0.009 0.688
## 2 Post-stress EOS_Control   1  25 3.331 0.080       0.034 0.160
## # A tibble: 1 × 10
##   .y.    group1  group2    n1    n2 statistic    df       p   p.adj p.adj.signif
## * <chr>  <chr>   <chr>  <int> <int>     <dbl> <dbl>   <dbl>   <dbl> <chr>
## 1 Stress Baseli… Post-…    54    54     -4.16    53 1.17e-4 1.17e-4 ***
## Warning: Removed 2 rows containing non-finite outside the scale range
## (`stat_boxplot()`).
## Warning: Removed 2 rows containing non-finite outside the scale range
## (`stat_boxplot()`).
## Warning: Removed 2 rows containing non-finite outside the scale range
## (`stat_summary()`).
## Notch went outside hinges
## ℹ Do you want `notch = FALSE`?
```

Assumptions for repeated measures ANOVA: the data set has no significant outliers. Normality is violated in EOS_Control B at Baseline timepoint. However, because the repeated measures ANOVA is robust to normality violations, we can proceed with the analysis.

The subjective stress was statistically significantly different between the Baseline and after stress task phases, F(1,25) = 14.29, p = 0.0009, generalized eta squared = 0.07. There was no significant difference between the EOS and Control groups.

Post-hoc testing using one-way ANOVA with Bonferroni adjusted p-value did not reveal significant effects at any timepoint. A pairwise comparison shows that the mean subjective stress was significantly different between the Baseline and Post-stress timepoints (p = 0.0001).

#### 4.1.2. Session

```
## # A tibble: 4 × 6
##   Session TIME        variable     n  mean    sd
##   <fct>   <fct>       <fct>    <dbl> <dbl> <dbl>
## 1 1       Baseline    Stress      28  3.36  1.93
## 2 2       Baseline    Stress      26  3.65  1.79
## 3 1       Post-stress Stress      28  4.86  1.96
## 4 2       Post-stress Stress      26  4.19  1.74
## Warning: Removed 2 rows containing non-finite outside the scale range
## (`stat_boxplot()`).
## Removed 2 rows containing non-finite outside the scale range
## (`stat_boxplot()`).
## Warning: Removed 2 rows containing non-finite outside the scale range
## (`stat_summary()`).
```

```
## [1] Session     TIME        Subject     EOS_Control Stress      is.outlier
## [7] is.extreme
## <0 rows> (or 0-length row.names)
## # A tibble: 4 × 5
##   Session TIME        variable statistic       p
##   <fct>   <fct>       <chr>        <dbl>   <dbl>
## 1 1       Baseline    Stress       0.864 0.00184
## 2 1       Post-stress Stress       0.950 0.202
## 3 2       Baseline    Stress       0.928 0.0709
## 4 2       Post-stress Stress       0.949 0.221
## Warning: Removed 2 rows containing non-finite outside the scale range
## (`stat_qq()`).
## Warning: Removed 2 rows containing non-finite outside the scale range
## (`stat_qq_line()`).
## Removed 2 rows containing non-finite outside the scale range
## (`stat_qq_line()`).
```

```
## Warning: NA detected in rows: 15,70.
## Removing this rows before the analysis.
## ANOVA Table (type III tests)
##
##         Effect DFn DFd      F        p p<.05   ges
## 1      Session   1  25  0.779 0.386000       0.005
## 2         TIME   1  25 14.294 0.000868     * 0.066
## 3 Session:TIME   1  25  3.376 0.078000       0.014
```

Assumptions for repeated measures ANOVA: the data set has no significant outliers. Normality is violated in EOS_Control B at the Time Baseline. However, because repeated measures ANOVA is robust to normality violations, we can proceed with the analysis.

Subjective stress was statistically significantly different between the Baseline and After Stress Task phases, F(1,25) = 14.29, p = 0.0009, generalized eta squared = 0.07. There was no significant difference between Sessions.

### 4.2. Positive Affect

#### 4.2.1. EOS x Control

```
## # A tibble: 4 × 6
##   EOS_Control TIME        variable     n  mean    sd
##   <chr>       <fct>       <fct>    <dbl> <dbl> <dbl>
## 1 A           Baseline    PA          28  31.9  6.93
## 2 B           Baseline    PA          26  31.5  6.73
## 3 A           Post-stress PA          28  29.2  8.17
## 4 B           Post-stress PA          26  29.3  7.64
## Warning: Removed 2 rows containing non-finite outside the scale range
## (`stat_boxplot()`).
## Removed 2 rows containing non-finite outside the scale range
## (`stat_boxplot()`).
## Warning: Removed 2 rows containing non-finite outside the scale range
## (`stat_summary()`).
```

```
## [1] EOS_Control TIME        Subject     Session     PA          is.outlier
## [7] is.extreme
## <0 rows> (or 0-length row.names)
## # A tibble: 4 × 5
##   EOS_Control TIME        variable statistic     p
##   <chr>       <fct>       <chr>        <dbl> <dbl>
## 1 A           Baseline    PA           0.978 0.801
## 2 A           Post-stress PA           0.971 0.599
## 3 B           Baseline    PA           0.971 0.650
## 4 B           Post-stress PA           0.976 0.775
## Warning: Removed 2 rows containing non-finite outside the scale range
## (`stat_qq()`).
## Warning: Removed 2 rows containing non-finite outside the scale range
## (`stat_qq_line()`).
## Removed 2 rows containing non-finite outside the scale range
## (`stat_qq_line()`).
```

```
## Warning: NA detected in rows: 15,70.
## Removing this rows before the analysis.
## ANOVA Table (type III tests)
##
##             Effect DFn DFd          F     p p<.05      ges
## 1      EOS_Control   1  25 9.0500e-04 0.976       6.96e-06
## 2             TIME   1  25 1.0584e+01 0.003     * 2.30e-02
## 3 EOS_Control:TIME   1  25 3.9400e-29 1.000       5.94e-32
## Warning: There were 2 warnings in `mutate()`.
## The first warning was:
## ℹ In argument: `data = map(.data$data, .f, ...)`.
## Caused by warning:
## ! NA detected in rows: 15.
## Removing this rows before the analysis.
## ℹ Run `dplyr::last_dplyr_warnings()` to see the 1 remaining warning.
##          TIME      Effect DFn DFd        F     p p<.05      ges p.adj
## 1    Baseline EOS_Control   1  25 0.000774 0.978       8.24e-06     1
## 2 Post-stress EOS_Control   1  25 0.000740 0.979       6.03e-06     1
## # A tibble: 1 × 10
##   .y.   group1   group2    n1    n2 statistic    df       p   p.adj p.adj.signif
## * <chr> <chr>    <chr>  <int> <int>     <dbl> <dbl>   <dbl>   <dbl> <chr>
## 1 PA    Baseline Post-…    54    54      3.94    53 2.37e-4 2.37e-4 ***
## Warning: Removed 2 rows containing non-finite outside the scale range
## (`stat_boxplot()`).
## Warning: Removed 2 rows containing non-finite outside the scale range
## (`stat_boxplot()`).
## Warning: Removed 2 rows containing non-finite outside the scale range
## (`stat_summary()`).
```

Assumptions for repeated measures ANOVA: the data set has no significant outliers. Normality is not violated. We can proceed with the analysis.

Subjective positive affect was statistically significantly different between the baseline and after stress task phases, F(1,25) = 10.58, p = 0.003, generalized eta squared = 0.02. There was no significant difference between the EOS and Control groups.

Post-hoc testing considering Bonferroni adjusted p-value reveals that the simple main effect of timepoint was non-significant at both timepoints (p.adj = 1.00).

A pairwise comparison showed that the mean positive affect score was significantly different between Baseline and Post-stress timepoints (p = 0.0002).

#### 4.2.2. Session

```
## # A tibble: 4 × 6
##   Session TIME        variable     n  mean    sd
##   <fct>   <fct>       <fct>    <dbl> <dbl> <dbl>
## 1 1       Baseline    PA          28  33.1  6.84
## 2 2       Baseline    PA          26  30.3  6.53
## 3 1       Post-stress PA          28  29.5  7.80
## 4 2       Post-stress PA          26  29.0  8.04
## Warning: Removed 2 rows containing non-finite outside the scale range
## (`stat_boxplot()`).
## Removed 2 rows containing non-finite outside the scale range
## (`stat_boxplot()`).
## Warning: Removed 2 rows containing non-finite outside the scale range
## (`stat_summary()`).
## Notch went outside hinges
## ℹ Do you want `notch = FALSE`?
```

```
## [1] Session     TIME        Subject     EOS_Control PA          is.outlier
## [7] is.extreme
## <0 rows> (or 0-length row.names)
## # A tibble: 4 × 5
##   Session TIME        variable statistic     p
##   <fct>   <fct>       <chr>        <dbl> <dbl>
## 1 1       Baseline    PA           0.976 0.756
## 2 1       Post-stress PA           0.981 0.867
## 3 2       Baseline    PA           0.986 0.967
## 4 2       Post-stress PA           0.973 0.703
## Warning: Removed 2 rows containing non-finite outside the scale range
## (`stat_qq()`).
## Warning: Removed 2 rows containing non-finite outside the scale range
## (`stat_qq_line()`).
## Removed 2 rows containing non-finite outside the scale range
## (`stat_qq_line()`).
```

```
## Warning: NA detected in rows: 15,70.
## Removing this rows before the analysis.
## ANOVA Table (type III tests)
##
##         Effect DFn DFd      F     p p<.05   ges
## 1      Session   1  25  1.456 0.239       0.011
## 2         TIME   1  25 10.584 0.003     * 0.023
## 3 Session:TIME   1  25  2.980 0.097       0.004
```

Assumptions for repeated measures ANOVA: the data set has no significant outliers. Normality is not violated. We can proceed with the analysis.

Positive affect was statistically significantly different between the baseline and after stress task phases, F(1,25) = 10.58, p = 0.003, generalized eta squared = 0.02. There was no significant difference between Sessions.

### 4.3. Negative Affect

#### 4.3.1. EOS x Control

```
## # A tibble: 4 × 6
##   EOS_Control TIME        variable     n  mean    sd
##   <chr>       <fct>       <fct>    <dbl> <dbl> <dbl>
## 1 A           Baseline    NeA         28  16.6  6.53
## 2 B           Baseline    NeA         26  15.6  6.31
## 3 A           Post-stress NeA         28  17.6  6.31
## 4 B           Post-stress NeA         26  18.8  7.60
## Warning: Removed 2 rows containing non-finite outside the scale range
## (`stat_boxplot()`).
## Removed 2 rows containing non-finite outside the scale range
## (`stat_boxplot()`).
## Warning: Removed 2 rows containing non-finite outside the scale range
## (`stat_summary()`).
## Notch went outside hinges
## ℹ Do you want `notch = FALSE`?
```

```
## # A tibble: 9 × 7
##   EOS_Control TIME        Subject Session   NeA is.outlier is.extreme
##   <chr>       <fct>       <chr>   <fct>   <dbl> <lgl>      <lgl>
## 1 A           Baseline    16      1          34 TRUE       FALSE
## 2 A           Baseline    22      2          30 TRUE       FALSE
## 3 A           Baseline    34      1          30 TRUE       FALSE
## 4 A           Post-stress 16      1          34 TRUE       FALSE
## 5 B           Baseline    16      2          37 TRUE       TRUE
## 6 B           Baseline    38      1          26 TRUE       FALSE
## 7 B           Baseline    5       1          27 TRUE       FALSE
## 8 B           Post-stress 22      1          34 TRUE       FALSE
## 9 B           Post-stress 38      1          36 TRUE       FALSE
## # A tibble: 7 × 7
##   EOS_Control TIME        Subject Session   NeA is.outlier is.extreme
##   <chr>       <fct>       <chr>   <fct>   <dbl> <lgl>      <lgl>
## 1 A           Baseline    22      2          30 TRUE       FALSE
## 2 A           Baseline    34      1          30 TRUE       FALSE
## 3 A           Post-stress 34      1          30 TRUE       FALSE
## 4 B           Baseline    38      1          26 TRUE       FALSE
## 5 B           Baseline    5       1          27 TRUE       FALSE
## 6 B           Post-stress 3       1          30 TRUE       FALSE
## 7 B           Post-stress 37      2          30 TRUE       FALSE
## Iteration: 1
## Number of outliers removed: 7
## Current size of data frame: 96 rows
##
## Iteration: 2
## Number of outliers removed: 5
## Current size of data frame: 88 rows
##
## Iteration: 3
## Number of outliers removed: 2
## Current size of data frame: 87 rows
##
## Iteration: 4
## Number of outliers removed: 2
## Current size of data frame: 82 rows
##
## No outliers found. Exiting loop.
```

```
## # A tibble: 4 × 5
##   EOS_Control TIME        variable statistic      p
##   <chr>       <fct>       <chr>        <dbl>  <dbl>
## 1 A           Baseline    NeA          0.940 0.222
## 2 A           Post-stress NeA          0.925 0.108
## 3 B           Baseline    NeA          0.920 0.0757
## 4 B           Post-stress NeA          0.960 0.610
```

```
## ANOVA Table (type III tests)
##
##             Effect DFn DFd     F     p p<.05   ges
## 1      EOS_Control   1  13 0.406 0.535       0.003
## 2             TIME   1  13 6.151 0.028     * 0.025
## 3 EOS_Control:TIME   1  13 0.972 0.342       0.009
##          TIME      Effect DFn DFd     F     p p<.05   ges p.adj
## 1    Baseline EOS_Control   1  17 2.267 0.151       0.022 0.302
## 2 Post-stress EOS_Control   1  15 0.096 0.761       0.001 1.000
```

Assumptions for repeated measures ANOVA: the data set has 9 outliers, 1 of which is extreme, and normality is violated in every measure. After removing the outliers, the normality test does not show statistically significant violations.

Subjective negative affect was statistically significantly different between the baseline and after stress task phases, F(1,13) = 6.151, p = 0.028, generalized eta squared = 0.025. There was no significant difference between the EOS and Control groups.

Post-hoc testing considering Bonferroni adjusted p-value did not reveal any significant effect of either timepoint on negative affect values.

#### 4.3.2. Session

```
## # A tibble: 4 × 6
##   Session TIME        variable     n  mean    sd
##   <fct>   <fct>       <fct>    <dbl> <dbl> <dbl>
## 1 1       Baseline    NeA         22  13.1  2.02
## 2 2       Baseline    NeA         21  13.7  3.02
## 3 1       Post-stress NeA         18  15.6  3.28
## 4 2       Post-stress NeA         21  13.8  2.88
## Notch went outside hinges
## ℹ Do you want `notch = FALSE`?
```

```
## # A tibble: 1 × 7
##   Session TIME        Subject EOS_Control   NeA is.outlier is.extreme
##   <fct>   <fct>       <chr>   <chr>       <dbl> <lgl>      <lgl>
## 1 2       Post-stress 25      A              20 TRUE       FALSE
## # A tibble: 4 × 5
##   Session TIME        variable statistic      p
##   <fct>   <fct>       <chr>        <dbl>  <dbl>
## 1 1       Baseline    NeA          0.915 0.0589
## 2 1       Post-stress NeA          0.939 0.276
## 3 2       Baseline    NeA          0.930 0.137
## 4 2       Post-stress NeA          0.938 0.202
```

```
## ANOVA Table (type III tests)
##
##         Effect DFn DFd     F     p p<.05   ges
## 1      Session   1  13 3.965 0.068       0.026
## 2         TIME   1  13 6.151 0.028     * 0.026
## 3 Session:TIME   1  13 3.114 0.101       0.026
##          TIME  Effect DFn DFd     F     p p<.05      ges p.adj
## 1    Baseline Session   1  17 0.056 0.816       0.000605 1.000
## 2 Post-stress Session   1  15 5.475 0.034     * 0.063000 0.068
```

Assumptions for repeated measures ANOVA: after removing outliers, normality was violated with borderline significance (p = 0.059) in the Baseline timepoint for first session. However, because repeated measures ANOVA is robust to normality violations, we can proceed with the analysis.

Negative affect was statistically significantly different between the baseline and after stress task phases, F(1,13) = 6.151, p = 0.028, generalized eta squared = 0.026.

The post-hoc analysis with the Bonferroni adjusted p-value shows that the simple main effect of Session was not significant at the baseline timepoint (p.adj= 1.00), and was borderline significant at the Post-stress timepoint (p.adj = 0.068).

### 4.4. Sleepiness

#### 4.4.1. EOS x Control

```
## # A tibble: 2 × 6
##   EOS_Control TIME     variable     n  mean    sd
##   <chr>       <fct>    <fct>    <dbl> <dbl> <dbl>
## 1 A           baseline KSS         28  4.79  1.71
## 2 B           baseline KSS         26  4.88  1.61
## Warning: Removed 1 row containing non-finite outside the scale range (`stat_boxplot()`).
## Removed 1 row containing non-finite outside the scale range (`stat_boxplot()`).
## Warning: Removed 1 row containing non-finite outside the scale range
## (`stat_summary()`).
```

```
## [1] EOS_Control  TIME         Subject      Session      baseline_KSS
## [6] KSS          is.outlier   is.extreme
## <0 rows> (or 0-length row.names)
## # A tibble: 2 × 4
##   EOS_Control variable statistic      p
##   <chr>       <chr>        <dbl>  <dbl>
## 1 A           KSS          0.922 0.0389
## 2 B           KSS          0.943 0.157
## Warning: Removed 1 row containing non-finite outside the scale range
## (`stat_qq()`).
## Warning: Removed 1 row containing non-finite outside the scale range (`stat_qq_line()`).
## Removed 1 row containing non-finite outside the scale range (`stat_qq_line()`).
```

```
## Warning in df_KSS$KSS[df_KSS$EOS_Control == "A"] -
## df_KSS$KSS[df_KSS$EOS_Control == : longer object length is not a multiple of
## shorter object length
##
##  Shapiro-Wilk normality test
##
## data:  differ
## W = 0.94257, p-value = 0.141
## Warning: Removed 1 row containing non-finite outside the scale range (`stat_qq()`).
## Removed 1 row containing non-finite outside the scale range (`stat_qq_line()`).
## Removed 1 row containing non-finite outside the scale range (`stat_qq_line()`).
```

```
##
##  One Sample t-test
##
## data:  differ
## t = 0.095783, df = 26, p-value = 0.9244
## alternative hypothesis: true mean is not equal to 0
## 95 percent confidence interval:
##  -0.7577910  0.8318651
## sample estimates:
##  mean of x
## 0.03703704
```

Assumptions for repeated measures ANOVA: the data set has no significant outliers. Normality is violated in EOS_Control A (p = 0.039), but the Q-Q plots seem reasonable. We proceed to test using a one-sample t-test.

Subjective sleepiness was not statistically significantly different between EOS and Control groups, p = 0.924.

#### 4.4.2. Session

```
## # A tibble: 2 × 6
##   Session TIME     variable     n  mean    sd
##   <fct>   <fct>    <fct>    <dbl> <dbl> <dbl>
## 1 1       baseline KSS         28  4.64  1.73
## 2 2       baseline KSS         26  5.04  1.56
## Warning: Removed 1 row containing non-finite outside the scale range (`stat_boxplot()`).
## Removed 1 row containing non-finite outside the scale range (`stat_boxplot()`).
## Warning: Removed 1 row containing non-finite outside the scale range
## (`stat_summary()`).
```

```
## [1] Session      TIME         Subject      EOS_Control  baseline_KSS
## [6] KSS          is.outlier   is.extreme
## <0 rows> (or 0-length row.names)
## # A tibble: 2 × 5
##   Session TIME     variable statistic      p
##   <fct>   <fct>    <chr>        <dbl>  <dbl>
## 1 1       baseline KSS          0.921 0.0372
## 2 2       baseline KSS          0.921 0.0486
## Warning: Removed 1 row containing non-finite outside the scale range
## (`stat_qq()`).
## Warning: Removed 1 row containing non-finite outside the scale range (`stat_qq_line()`).
## Removed 1 row containing non-finite outside the scale range (`stat_qq_line()`).
```

```
## Warning in df_KSS$KSS[df_KSS$Session == "1"] - df_KSS$KSS[df_KSS$Session == :
## longer object length is not a multiple of shorter object length
##
##  Shapiro-Wilk normality test
##
## data:  differ
## W = 0.93972, p-value = 0.1199
## Warning: Removed 1 row containing non-finite outside the scale range (`stat_qq()`).
## Removed 1 row containing non-finite outside the scale range (`stat_qq_line()`).
## Removed 1 row containing non-finite outside the scale range (`stat_qq_line()`).
```

```
##
##  One Sample t-test
##
## data:  differ
## t = -0.71168, df = 26, p-value = 0.483
## alternative hypothesis: true mean is not equal to 0
## 95 percent confidence interval:
##  -1.2960877  0.6294211
## sample estimates:
##  mean of x
## -0.3333333
```

The subjective sleepiness was not statistically significantly different between Sessions, p = 0.483.

### 4.5. rMSSD

#### 4.5.1. EOS x Control

```
## 'data.frame':    165 obs. of  5 variables:
##  $ Subject    : chr  "1" "1" "1" "1" ...
##  $ Session    : Factor w/ 2 levels "1","2": 1 1 1 2 2 2 1 1 1 1 ...
##  $ Phase      : Factor w/ 6 levels "Baseline","EOS_1",..: 1 4 5 1 4 5 1 4 5 1 ...
##  $ EOS_Control: chr  "B" "B" "B" "A" ...
##  $ HRV_RMSSD  : num  23.2 31.7 27.7 33.4 27.5 ...
## # A tibble: 6 × 6
##   Phase            EOS_Control variable      n  mean    sd
##   <fct>            <chr>       <fct>     <dbl> <dbl> <dbl>
## 1 Baseline         A           HRV_RMSSD    28  50.9  34.1
## 2 Baseline         B           HRV_RMSSD    27  50.3  28.1
## 3 Mast_Instruction A           HRV_RMSSD    28  45.8  19.4
## 4 Mast_Instruction B           HRV_RMSSD    27  46.3  22.1
## 5 Mast_Task        A           HRV_RMSSD    28  37.8  17.8
## 6 Mast_Task        B           HRV_RMSSD    27  38.1  20.0
```

```
## # A tibble: 11 × 7
##    Phase            EOS_Control Subject Session HRV_RMSSD is.outlier is.extreme
##    <fct>            <chr>       <chr>   <fct>       <dbl> <lgl>      <lgl>
##  1 Baseline         A           15      2           147.  TRUE       TRUE
##  2 Baseline         A           33      2           161.  TRUE       TRUE
##  3 Baseline         B           33      1           135.  TRUE       FALSE
##  4 Mast_Instruction A           15      2           104.  TRUE       FALSE
##  5 Mast_Instruction A           38      2            99.4 TRUE       FALSE
##  6 Mast_Instruction B           3       1            97.6 TRUE       FALSE
##  7 Mast_Instruction B           33      1           109.  TRUE       FALSE
##  8 Mast_Task        A           11      1            75.0 TRUE       FALSE
##  9 Mast_Task        A           15      2            88.6 TRUE       TRUE
## 10 Mast_Task        A           38      2            79.1 TRUE       FALSE
## 11 Mast_Task        B           33      1           122.  TRUE       TRUE
## # A tibble: 4 × 7
##   Phase            EOS_Control Subject Session HRV_RMSSD is.outlier is.extreme
##   <fct>            <chr>       <chr>   <fct>       <dbl> <lgl>      <lgl>
## 1 Mast_Task        A           11      1            75.0 TRUE       FALSE
## 2 Mast_Task        A           38      2            79.1 TRUE       TRUE
## 3 Baseline         B           15      1            86.6 TRUE       FALSE
## 4 Mast_Instruction B           38      1            82.0 TRUE       FALSE
## Iteration: 1
## Number of outliers removed: 4
## Current size of data frame: 148 rows
##
## Iteration: 2
## Number of outliers removed: 1
## Current size of data frame: 147 rows
##
## No outliers found. Exiting loop.
```

```
## # A tibble: 6 × 5
##   Phase            EOS_Control variable  statistic      p
##   <fct>            <chr>       <chr>         <dbl>  <dbl>
## 1 Baseline         A           HRV_RMSSD     0.939 0.157
## 2 Baseline         B           HRV_RMSSD     0.928 0.112
## 3 Mast_Instruction A           HRV_RMSSD     0.979 0.842
## 4 Mast_Instruction B           HRV_RMSSD     0.935 0.125
## 5 Mast_Task        A           HRV_RMSSD     0.929 0.0842
## 6 Mast_Task        B           HRV_RMSSD     0.961 0.408
```

```
## ANOVA Table (type III tests)
##
##              Effect DFn DFd     F        p p<.05      ges
## 1       EOS_Control   1  21 0.099 0.756000       0.000272
## 2             Phase   2  42 9.112 0.000517     * 0.064000
## 3 EOS_Control:Phase   2  42 0.978 0.385000       0.004000
## # A tibble: 2 × 9
##   EOS_Control Effect   DFn   DFd     F        p `p<.05`   ges    p.adj
##   <chr>       <chr>  <dbl> <dbl> <dbl>    <dbl> <chr>   <dbl>    <dbl>
## 1 A           Phase      2    46  9.67 0.000313 *       0.089 0.000626
## 2 B           Phase      2    42  3.98 0.026    *       0.042 0.052
```

Assumptions for repeated measures ANOVA: the data set has 11 outliers, 3 of which are extreme, and normality is violated in every combination. After removing the outliers, the normality test does not show statistically significant violations.

The rMSSD was statistically significantly different between phases, F(2, 42) = 9.112, p = 0.0005, generalized eta squared = 0.064. There was no significant relationship between the EOS and Control groups.

The post-hoc effect showed a statistically significant effect of Phase in groups A and B with p.adj = 0.0006 and 0.0520, respectively.

#### 4.5.2. Session

```
## Notch went outside hinges
## ℹ Do you want `notch = FALSE`?
```

```
## [1] Session     Phase       Subject     EOS_Control HRV_RMSSD   is.outlier
## [7] is.extreme
## <0 rows> (or 0-length row.names)
## # A tibble: 6 × 5
##   Session Phase            variable  statistic      p
##   <fct>   <fct>            <chr>         <dbl>  <dbl>
## 1 1       Baseline         HRV_RMSSD     0.899 0.0287
## 2 2       Baseline         HRV_RMSSD     0.944 0.197
## 3 1       Mast_Instruction HRV_RMSSD     0.969 0.617
## 4 2       Mast_Instruction HRV_RMSSD     0.957 0.364
## 5 1       Mast_Task        HRV_RMSSD     0.948 0.211
## 6 2       Mast_Task        HRV_RMSSD     0.949 0.241
```

```
## ANOVA Table (type III tests)
##
##          Effect DFn DFd     F        p p<.05      ges
## 1         Phase   2  42 9.112 0.000517     * 0.063000
## 2       Session   1  21 0.316 0.580000       0.000855
## 3 Phase:Session   2  42 0.318 0.730000       0.001000
```

After removing outliers, the rMSSD was statistically significantly different between phases, F(2, 42) = 9.112, p = 0.0005, generalized eta squared = 0.063. There was no significant relationship with Session.

### 4.6. LF

#### 4.6.1. EOS x Control

```
## 'data.frame':    165 obs. of  5 variables:
##  $ Subject    : chr  "1" "1" "1" "1" ...
##  $ Session    : Factor w/ 2 levels "1","2": 1 1 1 2 2 2 1 1 1 1 ...
##  $ Phase      : Factor w/ 6 levels "Baseline","EOS_1",..: 1 4 5 1 4 5 1 4 5 1 ...
##  $ EOS_Control: chr  "B" "B" "B" "A" ...
##  $ HRV_lf     : num  666 1494 991 401 1127 ...
## # A tibble: 6 × 6
##   Phase            EOS_Control variable     n  mean    sd
##   <fct>            <chr>       <fct>    <dbl> <dbl> <dbl>
## 1 Baseline         A           HRV_lf      28 1054. 1351.
## 2 Baseline         B           HRV_lf      27 1061.  895.
## 3 Mast_Instruction A           HRV_lf      28 1534.  790.
## 4 Mast_Instruction B           HRV_lf      27 1485.  766.
## 5 Mast_Task        A           HRV_lf      28 1047.  645.
## 6 Mast_Task        B           HRV_lf      27 1066.  545.
```

```
## # A tibble: 7 × 7
##   Phase            EOS_Control Subject Session HRV_lf is.outlier is.extreme
##   <fct>            <chr>       <chr>   <fct>    <dbl> <lgl>      <lgl>
## 1 Baseline         A           15      2        7297. TRUE       TRUE
## 2 Baseline         A           29      2        2739. TRUE       FALSE
## 3 Baseline         B           15      1        3953. TRUE       TRUE
## 4 Baseline         B           8       1        3427. TRUE       TRUE
## 5 Mast_Instruction B           3       1        3397. TRUE       FALSE
## 6 Mast_Task        A           15      2        3193. TRUE       FALSE
## 7 Mast_Task        B           29      1        2692. TRUE       FALSE
## # A tibble: 5 × 7
##   Phase     EOS_Control Subject Session HRV_lf is.outlier is.extreme
##   <fct>     <chr>       <chr>   <fct>    <dbl> <lgl>      <lgl>
## 1 Baseline  A           29      2        2739. TRUE       FALSE
## 2 Baseline  B           28      1        2153. TRUE       FALSE
## 3 Baseline  B           29      1        1675. TRUE       FALSE
## 4 Baseline  B           3       1        1707. TRUE       FALSE
## 5 Mast_Task B           29      1        2692. TRUE       FALSE
## Notch went outside hinges
## ℹ Do you want `notch = FALSE`?
```

```
## Iteration: 1
## Number of outliers removed: 5
## Current size of data frame: 154 rows
##
## Iteration: 2
## Number of outliers removed: 3
## Current size of data frame: 153 rows
##
## Iteration: 3
## Number of outliers removed: 3
## Current size of data frame: 153 rows
##
## Maximum iterations reached. Exiting loop.
## # A tibble: 3 × 7
##   Phase    EOS_Control Subject Session HRV_lf is.outlier is.extreme
##   <fct>    <chr>       <chr>   <fct>    <dbl> <lgl>      <lgl>
## 1 Baseline B           28      1        2153. TRUE       FALSE
## 2 Baseline B           29      1        1675. TRUE       FALSE
## 3 Baseline B           3       1        1707. TRUE       FALSE
## Notch went outside hinges
## ℹ Do you want `notch = FALSE`?
```

```
## [1] Phase       EOS_Control Subject     Session     HRV_lf      is.outlier
## [7] is.extreme
## <0 rows> (or 0-length row.names)
## # A tibble: 6 × 5
##   Phase            EOS_Control variable statistic      p
##   <fct>            <chr>       <chr>        <dbl>  <dbl>
## 1 Baseline         A           HRV_lf       0.933 0.0935
## 2 Baseline         B           HRV_lf       0.970 0.718
## 3 Mast_Instruction A           HRV_lf       0.955 0.316
## 4 Mast_Instruction B           HRV_lf       0.954 0.337
## 5 Mast_Task        A           HRV_lf       0.945 0.157
## 6 Mast_Task        B           HRV_lf       0.916 0.0372
```

```
## ANOVA Table (type III tests)
##
##              Effect DFn DFd      F        p p<.05      ges
## 1             Phase   2  40 21.408 4.77e-07     * 0.208000
## 2       EOS_Control   1  20  0.043 8.38e-01       0.000117
## 3 Phase:EOS_Control   2  40  1.917 1.60e-01       0.009000
## # A tibble: 2 × 9
##   EOS_Control Effect   DFn   DFd     F            p `p<.05`   ges       p.adj
##   <chr>       <chr>  <dbl> <dbl> <dbl>        <dbl> <chr>   <dbl>       <dbl>
## 1 A           Phase      2    48  24.9 0.0000000385 *       0.212 0.000000077
## 2 B           Phase      2    40  12.2 0.0000737    *       0.177 0.000147
```

Assumptions for repeated measures ANOVA: the dataset had seven outliers, three of them extreme. After removing outliers, normality test shows statistically significant violation in MAST_Task EOS_Control B. However, repeated measures ANOVA is robust towards normality violations and we may proceed with analysis.

The rMSSD was statistically significantly different between phases, F(2, 40) = 21.408, p < 0.001, generalized eta squared = 0.208. No significant relationship with EOS or Control group.

Post-hoc analysis with Bonferroni adjusted p-values revealed the effect of phase was significant in both groups (p.adj < 0.000, and 0.0002, respectively).

#### 4.6.2. Session

```
## Notch went outside hinges
## ℹ Do you want `notch = FALSE`?
```

```
## # A tibble: 6 × 7
##   Session Phase     Subject EOS_Control HRV_lf is.outlier is.extreme
##   <fct>   <fct>     <chr>   <chr>        <dbl> <lgl>      <lgl>
## 1 1       Baseline  15      B            3953. TRUE       TRUE
## 2 1       Baseline  8       B            3427. TRUE       FALSE
## 3 2       Baseline  15      A            7297. TRUE       TRUE
## 4 2       Baseline  29      A            2739. TRUE       FALSE
## 5 1       Mast_Task 29      B            2692. TRUE       TRUE
## 6 2       Mast_Task 15      A            3193. TRUE       FALSE
## # A tibble: 3 × 7
##   Session Phase     Subject EOS_Control HRV_lf is.outlier is.extreme
##   <fct>   <fct>     <chr>   <chr>        <dbl> <lgl>      <lgl>
## 1 1       Baseline  28      B            2153. TRUE       FALSE
## 2 2       Baseline  29      A            2739. TRUE       FALSE
## 3 1       Mast_Task 29      B            2692. TRUE       TRUE
## # A tibble: 2 × 7
##   Session Phase     Subject EOS_Control HRV_lf is.outlier is.extreme
##   <fct>   <fct>     <chr>   <chr>        <dbl> <lgl>      <lgl>
## 1 1       Baseline  28      B            2153. TRUE       FALSE
## 2 1       Mast_Task 3       B            1699. TRUE       FALSE
## # A tibble: 6 × 5
##   Session Phase            variable statistic      p
##   <fct>   <fct>            <chr>        <dbl>  <dbl>
## 1 1       Baseline         HRV_lf       0.896 0.0125
## 2 2       Baseline         HRV_lf       0.939 0.137
## 3 1       Mast_Instruction HRV_lf       0.957 0.331
## 4 2       Mast_Instruction HRV_lf       0.928 0.0887
## 5 1       Mast_Task        HRV_lf       0.947 0.186
## 6 2       Mast_Task        HRV_lf       0.941 0.142
```

```
## ANOVA Table (type III tests)
##
##          Effect DFn DFd      F        p p<.05   ges
## 1         Phase   2  42 21.999 2.91e-07     * 0.177
## 2       Session   1  21  0.773 3.89e-01       0.002
## 3 Phase:Session   2  42  0.573 5.68e-01       0.003
## # A tibble: 3 × 9
##   Phase            Effect    DFn   DFd     F     p `p<.05`     ges p.adj
##   <fct>            <chr>   <dbl> <dbl> <dbl> <dbl> <chr>     <dbl> <dbl>
## 1 Baseline         Session     1    23 0.045 0.833 ""      0.00034 1
## 2 Mast_Instruction Session     1    22 0.05  0.825 ""      0.00039 1
## 3 Mast_Task        Session     1    24 5.70  0.025 "*"     0.024   0.075
```

In data without outliers, the LF was statistically significantly different between phases, F(2, 42) = 21.999, p < 0.001, generalized eta squared = 0.177. No significant relationship with Sessions.

### 4.7. Alpha asymmetry

#### 4.7.1. EOS x Control

```
## 'data.frame':    110 obs. of  5 variables:
##  $ Subject        : chr  "1" "1" "1" "1" ...
##  $ Session        : Factor w/ 2 levels "1","2": 1 1 2 2 1 1 1 1 2 2 ...
##  $ Phase          : Factor w/ 6 levels "Baseline","EOS_1",..: 1 4 1 4 1 4 1 4 1 4 ...
##  $ EOS_Control    : chr  "B" "B" "A" "A" ...
##  $ alpha_asymmetry: num  -0.0834 -0.1449 0.5358 -0.0748 0.0613 ...
## # A tibble: 4 × 6
##   Phase            EOS_Control variable            n  mean    sd
##   <fct>            <chr>       <fct>           <dbl> <dbl> <dbl>
## 1 Baseline         A           alpha_asymmetry    28 0.142 0.435
## 2 Baseline         B           alpha_asymmetry    27 0.141 0.295
## 3 Mast_Instruction A           alpha_asymmetry    28 0.052 0.284
## 4 Mast_Instruction B           alpha_asymmetry    27 0.059 0.384
```

```
## # A tibble: 6 × 7
##   Phase        EOS_Control Subject Session alpha_asymmetry is.outlier is.extreme
##   <fct>        <chr>       <chr>   <fct>             <dbl> <lgl>      <lgl>
## 1 Baseline     A           4       1                 1.72  TRUE       TRUE
## 2 Baseline     B           33      1                 0.817 TRUE       FALSE
## 3 Mast_Instru… A           16      1                -0.520 TRUE       FALSE
## 4 Mast_Instru… A           37      1                 1.05  TRUE       TRUE
## 5 Mast_Instru… A           8       2                -0.462 TRUE       FALSE
## 6 Mast_Instru… B           15      1                 1.52  TRUE       TRUE
## # A tibble: 4 × 7
##   Phase        EOS_Control Subject Session alpha_asymmetry is.outlier is.extreme
##   <fct>        <chr>       <chr>   <fct>             <dbl> <lgl>      <lgl>
## 1 Baseline     A           37      1                 0.691 TRUE       FALSE
## 2 Mast_Instru… A           16      1                -0.520 TRUE       FALSE
## 3 Mast_Instru… A           8       2                -0.462 TRUE       FALSE
## 4 Baseline     B           38      1                 0.697 TRUE       FALSE
## [1] Phase           EOS_Control     Subject         Session
## [5] alpha_asymmetry is.outlier      is.extreme
## <0 rows> (or 0-length row.names)
## Notch went outside hinges
## ℹ Do you want `notch = FALSE`?
```

```
## # A tibble: 4 × 5
##   Phase            EOS_Control variable        statistic        p
##   <fct>            <chr>       <chr>               <dbl>    <dbl>
## 1 Baseline         A           alpha_asymmetry     0.850 0.000955
## 2 Baseline         B           alpha_asymmetry     0.978 0.818
## 3 Mast_Instruction A           alpha_asymmetry     0.874 0.00297
## 4 Mast_Instruction B           alpha_asymmetry     0.826 0.000399
```

```
## ANOVA Table (type III tests)
##
##              Effect DFn DFd     F     p p<.05      ges
## 1             Phase   1  26 1.401 0.247       1.60e-02
## 2       EOS_Control   1  26 0.003 0.956       2.91e-05
## 3 Phase:EOS_Control   1  26 0.020 0.889       1.15e-04
```

Assumptions for repeated measures ANOVA: the data set had six outliers, three were extreme. After removing the outliers, the normality test shows statistically significant violations in the majority of subgroups. However, repeated measures ANOVA is robust to normality violations and we may proceed with the analysis.

The repeated measures ANOVA did not reveal any statistically significant differences between Phases nor between EOS and Control groups.

#### 4.7.2. Session

```
## Notch went outside hinges
## ℹ Do you want `notch = FALSE`?
```

```
## # A tibble: 4 × 7
##   Session Phase        Subject EOS_Control alpha_asymmetry is.outlier is.extreme
##   <fct>   <fct>        <chr>   <chr>                 <dbl> <lgl>      <lgl>
## 1 2       Mast_Instru… 18      B                    -0.486 TRUE       FALSE
## 2 2       Mast_Instru… 20      B                    -0.368 TRUE       FALSE
## 3 2       Mast_Instru… 32      B                     0.258 TRUE       FALSE
## 4 2       Mast_Instru… 40      B                     0.301 TRUE       FALSE
## # A tibble: 3 × 7
##   Session Phase        Subject EOS_Control alpha_asymmetry is.outlier is.extreme
##   <fct>   <fct>        <chr>   <chr>                 <dbl> <lgl>      <lgl>
## 1 2       Mast_Instru… 15      A                     0.150 TRUE       FALSE
## 2 2       Mast_Instru… 24      B                    -0.303 TRUE       FALSE
## 3 2       Mast_Instru… 37      B                    -0.330 TRUE       FALSE
## # A tibble: 1 × 7
##   Session Phase        Subject EOS_Control alpha_asymmetry is.outlier is.extreme
##   <fct>   <fct>        <chr>   <chr>                 <dbl> <lgl>      <lgl>
## 1 2       Mast_Instru… 26      B                    -0.267 TRUE       FALSE
## [1] Session         Phase           Subject         EOS_Control
## [5] alpha_asymmetry is.outlier      is.extreme
## <0 rows> (or 0-length row.names)
## # A tibble: 4 × 5
##   Session Phase            variable        statistic     p
##   <fct>   <fct>            <chr>               <dbl> <dbl>
## 1 1       Baseline         alpha_asymmetry     0.938 0.162
## 2 2       Baseline         alpha_asymmetry     0.969 0.564
## 3 1       Mast_Instruction alpha_asymmetry     0.949 0.234
## 4 2       Mast_Instruction alpha_asymmetry     0.972 0.834
```

```
## ANOVA Table (type III tests)
##
##          Effect DFn DFd     F     p p<.05   ges
## 1         Phase   1  13 0.142 0.712       0.003
## 2       Session   1  13 4.966 0.044     * 0.081
## 3 Phase:Session   1  13 3.098 0.102       0.043
## # A tibble: 2 × 9
##   Phase            Effect    DFn   DFd      F     p `p<.05`   ges p.adj
##   <fct>            <chr>   <dbl> <dbl>  <dbl> <dbl> <chr>   <dbl> <dbl>
## 1 Baseline         Session     1    21  0.158 0.695 ""      0.002 1
## 2 Mast_Instruction Session     1    16 14.6   0.002 "*"     0.357 0.004
```

Assumptions for repeated measures ANOVA: the data without outliers had additional three outliers. After removing the outliers, the normality test did not show significant departures violations of normality.

The alpha asymmetry was statistically significantly different between sessions, F(1, 13) = 4.966, p = 0.044, generalized eta squared = 0.081. No significant relationship with Phases was found.

The post-hoc analysis revealed that the main effect of Session was not significant during the baseline timepoint (p.adj=1.00), but became significant during the instructions for stress task timepoint (p.adj = 0.004, generalized eta squared = 0.357).

### 4.8. Theta/Beta FP1

#### 4.8.1. EOS x Control

```
## 'data.frame':    110 obs. of  5 variables:
##  $ Subject    : chr  "1" "1" "1" "1" ...
##  $ Session    : Factor w/ 2 levels "1","2": 1 1 2 2 1 1 1 1 2 2 ...
##  $ Phase      : Factor w/ 6 levels "Baseline","EOS_1",..: 1 4 1 4 1 4 1 4 1 4 ...
##  $ EOS_Control: chr  "B" "B" "A" "A" ...
##  $ ThBt1      : num  3.07 6.95 5.16 5.24 12.7 ...
## # A tibble: 4 × 6
##   Phase            EOS_Control variable     n  mean    sd
##   <fct>            <chr>       <fct>    <dbl> <dbl> <dbl>
## 1 Baseline         A           ThBt1       28  7.26  4.21
## 2 Baseline         B           ThBt1       27  8.35  6.54
## 3 Mast_Instruction A           ThBt1       28  9.42  6.36
## 4 Mast_Instruction B           ThBt1       27 10.6   8.88
```

```
## # A tibble: 7 × 7
##   Phase            EOS_Control Subject Session ThBt1 is.outlier is.extreme
##   <fct>            <chr>       <chr>   <fct>   <dbl> <lgl>      <lgl>
## 1 Baseline         A           29      2        18.2 TRUE       FALSE
## 2 Baseline         A           37      1        17.7 TRUE       FALSE
## 3 Baseline         A           9       2        14.7 TRUE       FALSE
## 4 Baseline         B           29      1        31.5 TRUE       TRUE
## 5 Baseline         B           9       1        19.6 TRUE       FALSE
## 6 Mast_Instruction A           9       2        28.2 TRUE       FALSE
## 7 Mast_Instruction B           9       1        44.1 TRUE       TRUE
## # A tibble: 5 × 7
##   Phase    EOS_Control Subject Session ThBt1 is.outlier is.extreme
##   <fct>    <chr>       <chr>   <fct>   <dbl> <lgl>      <lgl>
## 1 Baseline A           29      2        18.2 TRUE       FALSE
## 2 Baseline A           37      1        17.7 TRUE       FALSE
## 3 Baseline A           9       2        14.7 TRUE       FALSE
## 4 Baseline B           12      1        16.8 TRUE       FALSE
## 5 Baseline B           9       1        19.6 TRUE       FALSE
## [1] Phase       EOS_Control Subject     Session     ThBt1       is.outlier
## [7] is.extreme
## <0 rows> (or 0-length row.names)
```

```
## # A tibble: 4 × 5
##   Phase            EOS_Control variable statistic      p
##   <fct>            <chr>       <chr>        <dbl>  <dbl>
## 1 Baseline         A           ThBt1        0.980 0.878
## 2 Baseline         B           ThBt1        0.936 0.135
## 3 Mast_Instruction A           ThBt1        0.956 0.317
## 4 Mast_Instruction B           ThBt1        0.892 0.0122
```

```
## ANOVA Table (type III tests)
##
##              Effect DFn DFd     F     p p<.05     ges
## 1       EOS_Control   1  21 1.883 0.184       2.1e-02
## 2             Phase   1  21 6.702 0.017     * 4.9e-02
## 3 EOS_Control:Phase   1  21 0.008 0.930       1.9e-05
## # A tibble: 2 × 9
##   EOS_Control Effect   DFn   DFd     F     p `p<.05`   ges p.adj
##   <chr>       <chr>  <dbl> <dbl> <dbl> <dbl> <chr>   <dbl> <dbl>
## 1 A           Phase      1    23  6.75 0.016 *       0.063 0.032
## 2 B           Phase      1    23  5.84 0.024 *       0.041 0.048
```

Assumptions for repeated measures ANOVA: the data set had seven outliers, two of which were extreme. After removing the outliers, the normality test shows statistically significant violations in the Mast_Instruction, EOS_Control B subgroup. However, repeated measures ANOVA is robust towards normality violations and we may proceed with the analysis.

The Theta/Beta FP1 was statistically significantly different between phases, F(1, 21) = 6.702, p = 0.017, generalized eta squared = 0.049. No significant relationship with EOS or Control group was seen.

The post-hoc analysis with Bonferroni adjusted p-values revealed that the effect of phase was significant in both intervention groups (p.adj = 0.032 and p = 0.048).

#### 4.8.2. Session

```
## Notch went outside hinges
## ℹ Do you want `notch = FALSE`?
```

```
## # A tibble: 1 × 7
##   Session Phase    Subject EOS_Control ThBt1 is.outlier is.extreme
##   <fct>   <fct>    <chr>   <chr>       <dbl> <lgl>      <lgl>
## 1 1       Baseline 33      B            15.5 TRUE       FALSE
## [1] Session     Phase       Subject     EOS_Control ThBt1       is.outlier
## [7] is.extreme
## <0 rows> (or 0-length row.names)
## # A tibble: 4 × 5
##   Session Phase            variable statistic      p
##   <fct>   <fct>            <chr>        <dbl>  <dbl>
## 1 1       Baseline         ThBt1        0.959 0.451
## 2 2       Baseline         ThBt1        0.977 0.810
## 3 1       Mast_Instruction ThBt1        0.920 0.0517
## 4 2       Mast_Instruction ThBt1        0.948 0.208
```

```
## ANOVA Table (type III tests)
##
##          Effect DFn DFd     F     p p<.05   ges
## 1         Phase   1  20 8.283 0.009     * 0.065
## 2       Session   1  20 3.538 0.075       0.036
## 3 Phase:Session   1  20 3.068 0.095       0.007
## # A tibble: 2 × 9
##   Session Effect   DFn   DFd     F     p `p<.05`   ges p.adj
##   <fct>   <chr>  <dbl> <dbl> <dbl> <dbl> <chr>   <dbl> <dbl>
## 1 1       Phase      1    21 12.2  0.002 "*"     0.087 0.004
## 2 2       Phase      1    24  3.21 0.086 ""      0.037 0.172
```

Assumptions for repeated measures ANOVA: the data set had several outliers. After removing the outliers, the normality test did not show significant departures violations of normality.

The alpha asymmetry was statistically significantly different between Phases, F(1, 20) = 8.283, p = 0.009, eta2 = 0.065. No significant relationship with Sessions was seen.

The post-hoc analysis revealed that the main effect of Phase was significant during the first session (p.adj=0.004, eta2 = 0.087), but became insignificant during the second session (p.adj = 0.172, eta2 = 0.357).

### 4.9. Theta/Beta FP2

#### 4.9.1. EOS x Control

```
## 'data.frame':    110 obs. of  5 variables:
##  $ Subject    : chr  "1" "1" "1" "1" ...
##  $ Session    : Factor w/ 2 levels "1","2": 1 1 2 2 1 1 1 1 2 2 ...
##  $ Phase      : Factor w/ 6 levels "Baseline","EOS_1",..: 1 4 1 4 1 4 1 4 1 4 ...
##  $ EOS_Control: chr  "B" "B" "A" "A" ...
##  $ ThBt2      : num  4.42 7.25 2.55 3.65 11.81 ...
## # A tibble: 4 × 6
##   Phase            EOS_Control variable     n  mean    sd
##   <fct>            <chr>       <fct>    <dbl> <dbl> <dbl>
## 1 Baseline         A           ThBt2       28  7.9   4.49
## 2 Baseline         B           ThBt2       27  8.61  6.88
## 3 Mast_Instruction A           ThBt2       28 12.6  11.8
## 4 Mast_Instruction B           ThBt2       27 11.9   7.66
## Notch went outside hinges
## ℹ Do you want `notch = FALSE`?
## Notch went outside hinges
## ℹ Do you want `notch = FALSE`?
```

```
## # A tibble: 8 × 7
##   Phase            EOS_Control Subject Session ThBt2 is.outlier is.extreme
##   <fct>            <chr>       <chr>   <fct>   <dbl> <lgl>      <lgl>
## 1 Baseline         A           3       2        19.2 TRUE       FALSE
## 2 Baseline         A           9       2        16.8 TRUE       FALSE
## 3 Baseline         B           29      1        35.8 TRUE       TRUE
## 4 Baseline         B           3       1        18.0 TRUE       FALSE
## 5 Baseline         B           9       1        18.5 TRUE       FALSE
## 6 Mast_Instruction A           11      1        43.6 TRUE       FALSE
## 7 Mast_Instruction A           37      1        51.9 TRUE       TRUE
## 8 Mast_Instruction B           9       1        40.1 TRUE       TRUE
## # A tibble: 5 × 7
##   Phase            EOS_Control Subject Session ThBt2 is.outlier is.extreme
##   <fct>            <chr>       <chr>   <fct>   <dbl> <lgl>      <lgl>
## 1 Baseline         A           3       2        19.2 TRUE       FALSE
## 2 Baseline         A           9       2        16.8 TRUE       FALSE
## 3 Baseline         B           3       1        18.0 TRUE       FALSE
## 4 Baseline         B           9       1        18.5 TRUE       FALSE
## 5 Mast_Instruction B           33      1        21.2 TRUE       FALSE
## [1] Phase       EOS_Control Subject     Session     ThBt2       is.outlier
## [7] is.extreme
## <0 rows> (or 0-length row.names)
## Notch went outside hinges
## ℹ Do you want `notch = FALSE`?
## Notch went outside hinges
## ℹ Do you want `notch = FALSE`?
```

```
## # A tibble: 4 × 5
##   Phase            EOS_Control variable statistic      p
##   <fct>            <chr>       <chr>        <dbl>  <dbl>
## 1 Baseline         A           ThBt2        0.946 0.184
## 2 Baseline         B           ThBt2        0.906 0.0285
## 3 Mast_Instruction A           ThBt2        0.925 0.0681
## 4 Mast_Instruction B           ThBt2        0.970 0.673
```

```
## ANOVA Table (type III tests)
##
##              Effect DFn DFd      F        p p<.05   ges
## 1       EOS_Control   1  21  1.207 0.284000       0.012
## 2             Phase   1  21 18.836 0.000288     * 0.117
## 3 EOS_Control:Phase   1  21  1.838 0.190000       0.007
## # A tibble: 2 × 9
##   EOS_Control Effect   DFn   DFd     F        p `p<.05`   ges   p.adj
##   <chr>       <chr>  <dbl> <dbl> <dbl>    <dbl> <chr>   <dbl>   <dbl>
## 1 A           Phase      1    23  7.70 0.011    *       0.055 0.022
## 2 B           Phase      1    22 22.3  0.000105 *       0.202 0.00021
```

Assumptions for repeated measures ANOVA: the data set had eight outliers, three of which were extreme. After removing the outliers, the normality test shows statistically significant violations in the Baseline, EOS_Control B subgroup. However, repeated measures ANOVA is robust towards normality violations and we may proceed with the analysis.

The Theta/Beta FP1 was statistically significantly different between phases, F(1, 21) = 18.836, p = 0.0003, generalized eta squared = 0.117. No significant relationship with EOS or Control group was seen.

The post-hoc analysis with Bonferroni adjusted p-values revealed the effect of phase was significant in both intervention groups (p.adj = 0.022 and p.adj = 0.0002).

#### 4.9.2. Session

```
## Notch went outside hinges
## ℹ Do you want `notch = FALSE`?
## Notch went outside hinges
## ℹ Do you want `notch = FALSE`?
```

```
## [1] Session     Phase       Subject     EOS_Control ThBt2       is.outlier
## [7] is.extreme
## <0 rows> (or 0-length row.names)
## # A tibble: 4 × 5
##   Session Phase            variable statistic      p
##   <fct>   <fct>            <chr>        <dbl>  <dbl>
## 1 1       Baseline         ThBt2        0.950 0.257
## 2 2       Baseline         ThBt2        0.929 0.0828
## 3 1       Mast_Instruction ThBt2        0.922 0.0753
## 4 2       Mast_Instruction ThBt2        0.964 0.481
```

```
## ANOVA Table (type III tests)
##
##          Effect DFn DFd      F        p p<.05   ges
## 1         Phase   1  21 18.836 0.000288     * 0.119
## 2       Session   1  21  2.144 0.158000       0.020
## 3 Phase:Session   1  21  5.766 0.026000     * 0.018
## # A tibble: 2 × 9
##   Session Effect   DFn   DFd     F         p `p<.05`   ges    p.adj
##   <fct>   <chr>  <dbl> <dbl> <dbl>     <dbl> <chr>   <dbl>    <dbl>
## 1 1       Phase      1    21  6.16 0.022     *       0.06  0.044
## 2 2       Phase      1    24 23.5  0.0000611 *       0.156 0.000122
```

Assumptions for repeated measures ANOVA: the data set has no significant outliers. Normality is not violated. We can proceed with the analysis.

Theta/Beta FP2 was statistically significantly different between Phases, F(1, 21) = 18.836, p = 0.0003, generalized eta squared = 0.119. There was a statistically significant interaction between Phase and Session.

The post-hoc analysis revealed that the main effect of Phase was significant for both sessions (p.adj = 0.044, generalized eta squared = 0.060; p.adj = 0.0001, generalized eta squared = 0.156).

## 5. Experimental phase modelling

The goal of this study is to assess the effectiveness of the blue light and coherent breathing intervention and compare it to a neutral condition following exposure to a multisource stress.

The study is controlled, randomized, and double-blinded (to the analyst and to the researchers).

First, physiological indicators related to the stress response and emotion processing are analyzed:

- **root Mean Square Successive Differences (rMSSD)** is a suitable metric to study the variability between successive peaks heart rate signal, especially suitable for short recordings (5-minute intervals). It is linked to *nervus vagus* activity. Higher values (in ms) are related to calmer states;
- **Low Frequency Heart Rate Variability (LF)** is a frequency-based variability metric, also suitable for short recordings. It reflects baroreceptor activity during rest, dynamics of blood pressure and of the autonomic nervous system. During slow respiration (<7s periods), vagal activity can induce heart rate oscillations projecting to the LF band. Higher values (ms^2) relate to higher baroreceptor activity;
- **frontal Alpha Symmetry** is a metric reflecting relative difference between left and right alpha activity over the frontal regions of the brain. It is calculated from the alpha power spectrum (8-13 Hz; generally associated with attentive states) by log-transforming values from the right side and subtracting from them log-transformed values from left side. Higher scores reflect relatively greater left frontal activity and may be associated with approach-oriented reactions. Lower activity may be related to worse emotion regulation;
- **Theta/Beta Ratio** is a metric reflecting cognitive demand, calculated by dividing the absolute theta power (4-7 Hz) by the absolute beta power (13-30Hz). It reflects the relationship of internal slow and fast frequency activity. Higher values are related to poorer cognitive efficiency.

### 5.1. Physiological variables and demographics

#### 5.1.1. rMSSD

##### 5.1.1.1. Boxplots rMSSD

```
fulldata$Phase <- factor(fulldata$Phase, levels=c("Baseline", "Mast_Instruction", "Mast_Task", "EOS_1", "EOS_2", "Post_EOS"), ordered = T)

cbPalette <- c("#999999", "#E69F00", "#56B4E9", "#009E73", "#F0E442", "#0072B2", "#D55E00", "#CC79A7")


ggplot(fulldata, aes(x=(Phase), y = HRV_RMSSD, fill = (Phase)))+
  geom_boxplot()+
  scale_fill_manual(values=cbPalette) +
  facet_wrap(~EOS_Control)+
  theme(legend.position="none")+
  xlab("Phase") +
  ylab("rMSSD (ms)")
```

```
ggplot(fulldata4to6, aes(x=factor(EOS_Control), y = HRV_RMSSD, fill = factor(EOS_Control)))+
  geom_boxplot()+
    facet_wrap(~Session)+
    theme(legend.position="none")+
  scale_fill_manual(
    values = c("steelblue", "slategrey")
  ) +
    xlab("EOS or Control") +
    ylab("rMSSD (ms)")
```

##### 5.1.1.2. Full model rMSSD

```
mod1 <- lme(log(HRV_RMSSD) ~ EOS_Control + Phase + BMI + demo_age + demo_sex, random = ~ 1 | Subject, data = fulldata4to6, na.action = na.omit)

summary(mod1)
## Linear mixed-effects model fit by REML
##   Data: fulldata4to6
##        AIC      BIC    logLik
##   209.7763 237.1671 -95.88816
##
## Random effects:
##  Formula: ~1 | Subject
##         (Intercept)  Residual
## StdDev:   0.3564992 0.3431744
##
## Fixed effects:  log(HRV_RMSSD) ~ EOS_Control + Phase + BMI + demo_age + demo_sex
##                   Value Std.Error  DF   t-value p-value
## (Intercept)    3.579418 0.4954845 129  7.224077  0.0000
## EOS_ControlB  -0.055650 0.0547322 129 -1.016769  0.3112
## PhaseEOS_1     0.562586 0.0660439 129  8.518356  0.0000
## PhaseEOS_2     0.464806 0.0660439 129  7.037837  0.0000
## BMI            0.029411 0.0217216 129  1.354020  0.1781
## demo_age      -0.014776 0.0089548 129 -1.650038  0.1014
## demo_sexHomme -0.222241 0.1479896  26 -1.501737  0.1452
##  Correlation:
##               (Intr) EOS_CB PEOS_1 PEOS_2 BMI    demo_g
## EOS_ControlB  -0.050
## PhaseEOS_1    -0.067  0.000
## PhaseEOS_2    -0.067  0.000  0.500
## BMI           -0.836 -0.001  0.000  0.000
## demo_age      -0.221  0.004  0.000  0.000 -0.310
## demo_sexHomme -0.075 -0.020  0.000  0.000 -0.105  0.102
##
## Standardized Within-Group Residuals:
##           Min            Q1           Med            Q3           Max
## -3.6495112733 -0.5275687268 -0.0007013824  0.5398927144  3.0207301471
##
## Number of Observations: 162
## Number of Groups: 28
par(mfrow=c(1, 3))

plot(mod1$residuals)

qqnorm(mod1$residuals)

qqline(mod1$residuals, col="red")

hist(mod1$residuals)
```

plot(mod1, resid(., type = "p") ~ fitted(.) | EOS_Control, abline = 0)

plot(mod1, Subject ~ resid(.))

plot(mod1, HRV_RMSSD ~ fitted(.) | Subject, abline = c(0,1))

```
lillie.test(mod1$residuals)
##
##  Lilliefors (Kolmogorov-Smirnov) normality test
##
## data:  mod1$residuals
## D = 0.046388, p-value = 0.08984
((exp(coef(mod1)["PhaseEOS_1"]) - 1) * 100)[1, 1]
## [1] 75.52052
((exp(coef(mod1)["PhaseEOS_2"]) - 1) * 100)[1, 1]
## [1] 59.17061
intervals(mod1)
## Approximate 95% confidence intervals
##
##  Fixed effects:
##                     lower        est.       upper
## (Intercept)    2.59908997  3.57941822 4.559746466
## EOS_ControlB  -0.16393894 -0.05564998 0.052638973
## PhaseEOS_1     0.43191622  0.56258577 0.693255315
## PhaseEOS_2     0.33413694  0.46480649 0.595476033
## BMI           -0.01356523  0.02941147 0.072388161
## demo_age      -0.03249305 -0.01477576 0.002941533
## demo_sexHomme -0.52643841 -0.22224147 0.081955482
##
##  Random Effects:
##   Level: Subject
##                     lower      est.     upper
## sd((Intercept)) 0.2558172 0.3564992 0.4968065
##
##  Within-group standard error:
##     lower      est.     upper
## 0.3039932 0.3431744 0.3874055
```

The mixed-effects model analysis revealed significant positive effects of PhaseEOS_1 and PhaseEOS_2 on log(HRV_RMSSD) where values increased compared to the post-intervention phase by 75.52 % and by 59.12 %, respectively. Conversely, none of the other predictor variables, including intervention type, BMI, age, nor sex, displayed statistically significant effects on log(HRV_RMSSD). Variability in intercepts among subjects had a SD of 0.356; similarly, residual variability had a SD of 0.343. Correlation analyses showed low associations between predictor variables. Standardized within-group residuals exhibited acceptable distributional properties, with no significant departures from normality according to the Lilliefors (Kolmogorov-Smirnov) test (p = 0.08984).

##### 5.1.1.3. Simple model rMSSD

```
mod2 <- lme(log(HRV_RMSSD) ~ EOS_Control, random = ~ 1 | Subject, data = fulldata4to6, na.action = na.omit)

summary(mod2)
## Linear mixed-effects model fit by REML
##   Data: fulldata4to6
##        AIC      BIC    logLik
##   254.5698 266.9448 -123.2849
##
## Random effects:
##  Formula: ~1 | Subject
##         (Intercept)  Residual
## StdDev:   0.3477712 0.4419462
##
## Fixed effects:  log(HRV_RMSSD) ~ EOS_Control
##                  Value  Std.Error  DF  t-value p-value
## (Intercept)   4.078509 0.08151475 136 50.03400  0.0000
## EOS_ControlB -0.050489 0.06922784 136 -0.72931  0.4671
##  Correlation:
##              (Intr)
## EOS_ControlB -0.412
##
## Standardized Within-Group Residuals:
##        Min         Q1        Med         Q3        Max
## -3.5134712 -0.6235130  0.1682707  0.6311679  1.7550306
##
## Number of Observations: 165
## Number of Groups: 28
par(mfrow=c(1, 3))

plot(mod2$residuals)

qqnorm(mod2$residuals)

qqline(mod2$residuals, col="red")

hist(mod2$residuals)
```

plot(mod2, resid(., type = "p") ~ fitted(.) | EOS_Control, abline = 0)

plot(mod2, Subject ~ resid(.))

plot(mod2, HRV_RMSSD ~ fitted(.) | Subject, abline = c(0,1))

```
lillie.test(mod2$residuals)
##
##  Lilliefors (Kolmogorov-Smirnov) normality test
##
## data:  mod2$residuals
## D = 0.086067, p-value = 3.658e-06
((exp(coef(mod2)["EOS_ControlB"]) - 1) * 100)[1, 1]
## [1] -4.923524
```

A simplified model using only significant predictors on data without outliers has worse AIC and BIC performance, as well as lower p-values in the Lilliefors (Kolmogorov-Smirnov) normality test. This indicates worse performance compared to the full model on the data without outliers.

##### 5.1.1.4. Model comparison rMSSD

```
# Function to extract model information
extract_model_info <- function(model) {
  AIC <- AIC(model)
  BIC <- BIC(model)
  logLik <- logLik(model)
  fixed_effects <- paste(names(fixef(model)), collapse = ", ")
  random_effects <- paste(names(ranef(model)), collapse = ", ")
  return(c(AIC, BIC, logLik, fixed_effects, random_effects))
}

# Extract relevant information from each model
model_names <- c("Model 1", "Model 2")
model_info <- sapply(list(mod1, mod2), extract_model_info)

# Create a data frame to store the information
model_comparison <- data.frame(Model = model_names,
                                Fixed_Effects_added = model_info[4,],
                                Random_Effects = model_info[5,],
                                Model_fit_AIC = model_info[1,],
                                Model_fit_BIC = model_info[2,],
                                Model_fit_logLik = model_info[3,])


# Print the table
print(model_comparison)
##     Model
## 1 Model 1
## 2 Model 2
##                                                               Fixed_Effects_added
## 1 (Intercept), EOS_ControlB, PhaseEOS_1, PhaseEOS_2, BMI, demo_age, demo_sexHomme
## 2                                                       (Intercept), EOS_ControlB
##   Random_Effects    Model_fit_AIC    Model_fit_BIC  Model_fit_logLik
## 1    (Intercept) 209.776311338363 237.167137390636 -95.8881556691813
## 2    (Intercept)  254.56977773596 266.944778539187  -123.28488886798
```

The full model (5.1.1.2.) shows better performance based on the BIC criterion.

#### 5.1.2. LF

##### 5.1.2.1. Boxplots LF

```
ggplot(fulldata, aes(x=(Phase), y = HRV_lf, fill = (Phase)))+
  geom_boxplot()+
  scale_fill_manual(values=cbPalette) +
    facet_wrap(~EOS_Control)+
    theme(legend.position="none")+
    xlab("Phase") +
    ylab("LF (ms^2)")
```

```
ggplot(fulldata4to6, aes(x=factor(EOS_Control), y = HRV_lf, fill = factor(EOS_Control)))+
  geom_boxplot()+
    facet_wrap(~Session)+
    theme(legend.position="none")+
  scale_fill_manual(
    values = c("steelblue", "slategrey")
  ) +
    xlab("EOS or Control") +
    ylab("LF (ms^2)")
```

##### 5.1.2.2. Full model LF

```
mod1 <- lme(log(HRV_lf) ~ EOS_Control + Phase + BMI + demo_age + demo_sex, random = ~ 1 | Subject, data = fulldata4to6, na.action = na.omit)

summary(mod1)
## Linear mixed-effects model fit by REML
##   Data: fulldata4to6
##        AIC      BIC    logLik
##   413.5972 440.9881 -197.7986
##
## Random effects:
##  Formula: ~1 | Subject
##         (Intercept)  Residual
## StdDev:   0.4378274 0.7048202
##
## Fixed effects:  log(HRV_lf) ~ EOS_Control + Phase + BMI + demo_age + demo_sex
##                   Value Std.Error  DF   t-value p-value
## (Intercept)    8.194824 0.6921890 129 11.838998  0.0000
## EOS_ControlB  -0.673159 0.1119756 129 -6.011656  0.0000
## PhaseEOS_1     0.614860 0.1356427 129  4.532937  0.0000
## PhaseEOS_2     0.194508 0.1356427 129  1.433970  0.1540
## BMI            0.003172 0.0300549 129  0.105532  0.9161
## demo_age      -0.035416 0.0123333 129 -2.871615  0.0048
## demo_sexHomme  0.233553 0.2030504  26  1.150223  0.2605
##  Correlation:
##               (Intr) EOS_CB PEOS_1 PEOS_2 BMI    demo_g
## EOS_ControlB  -0.081
## PhaseEOS_1    -0.098  0.000
## PhaseEOS_2    -0.098  0.000  0.500
## BMI           -0.834  0.002  0.000  0.000
## demo_age      -0.227  0.012  0.000  0.000 -0.299
## demo_sexHomme -0.069 -0.027  0.000  0.000 -0.106  0.097
##
## Standardized Within-Group Residuals:
##         Min          Q1         Med          Q3         Max
## -2.66840793 -0.48904435  0.07213004  0.62749033  1.84056401
##
## Number of Observations: 162
## Number of Groups: 28
par(mfrow=c(1, 3))

plot(mod1$residuals)

qqnorm(mod1$residuals)

qqline(mod1$residuals, col="red")

hist(mod1$residuals)
```

plot(mod1, resid(., type = "p") ~ fitted(.) | EOS_Control, abline = 0)

plot(mod1, Subject ~ resid(.))

plot(mod1, HRV_lf ~ fitted(.) | Subject, abline = c(0,1))

```
lillie.test(mod1$residuals)
##
##  Lilliefors (Kolmogorov-Smirnov) normality test
##
## data:  mod1$residuals
## D = 0.049176, p-value = 0.05688
((exp(coef(mod1)["EOS_ControlB"]) - 1) * 100)[1, 1]
## [1] -48.99052
((exp(coef(mod1)["PhaseEOS_1"]) - 1) * 100)[1, 1]
## [1] 84.93974
((exp(coef(mod1)["demo_age"]) - 1) * 100)[1, 1]
## [1] -3.479666
```

The mixed-effects model analysis revealed significant positive effects of intervention type on log(HRV_lf), with group B decreasing values by 48.99 %. PhaseEOS_1 increased by 84.94 % and age decreased by 3.48 %. None of the other predictor variables, including PhaseEOS_2, sex, and BMI, revealed significant effects on log(HRV_lf). The standard deviation (SD) of the intercept (0.438) and residual (0.705) suggest considerable variability among individuals that is not explained by the fixed effects. Correlation analysis showed low associations between predictor variables. The Lilliefors (Kolmogorov-Smirnov) normality test on the residuals yielded a p-value of 0.057, suggesting borderline evidence against the null hypothesis of normality. However, this p-value should be interpreted with caution as it is close to the conventional significance threshold of 0.05.

##### 5.1.2.3. Simple model LF

```
mod2 <- lme(log(HRV_lf) ~ EOS_Control + Phase + demo_age, random = ~ 1 | Subject, data = fulldata4to6, na.action = na.omit)

summary(mod2)
## Linear mixed-effects model fit by REML
##   Data: fulldata4to6
##        AIC      BIC    logLik
##   404.4282 425.8219 -195.2141
##
## Random effects:
##  Formula: ~1 | Subject
##         (Intercept)  Residual
## StdDev:   0.4283855 0.7051691
##
## Fixed effects:  log(HRV_lf) ~ EOS_Control + Phase + demo_age
##                  Value Std.Error  DF   t-value p-value
## (Intercept)   8.382431 0.3607550 130 23.235802  0.0000
## EOS_ControlB -0.669141 0.1119694 130 -5.976108  0.0000
## PhaseEOS_1    0.614860 0.1357099 130  4.530694  0.0000
## PhaseEOS_2    0.194508 0.1357099 130  1.433261  0.1542
## demo_age     -0.035914 0.0115730 130 -3.103299  0.0023
##  Correlation:
##              (Intr) EOS_CB PEOS_1 PEOS_2
## EOS_ControlB -0.161
## PhaseEOS_1   -0.188  0.000
## PhaseEOS_2   -0.188  0.000  0.500
## demo_age     -0.926  0.015  0.000  0.000
##
## Standardized Within-Group Residuals:
##         Min          Q1         Med          Q3         Max
## -2.74671433 -0.47282024  0.04829749  0.66497844  1.78264627
##
## Number of Observations: 162
## Number of Groups: 28
par(mfrow=c(1, 3))

plot(mod2$residuals)

qqnorm(mod2$residuals)

qqline(mod2$residuals, col="red")

hist(mod2$residuals)
```

plot(mod2, resid(., type = "p") ~ fitted(.) | EOS_Control, abline = 0)

plot(mod2, Subject ~ resid(.))

plot(mod2, HRV_lf ~ fitted(.) | Subject, abline = c(0,1))

```
lillie.test(mod2$residuals)
##
##  Lilliefors (Kolmogorov-Smirnov) normality test
##
## data:  mod2$residuals
## D = 0.059304, p-value = 0.008021
((exp(coef(mod2)["EOS_ControlB"]) - 1) * 100)[1, 1]
## [1] -48.78517
((exp(coef(mod2)["PhaseEOS_2"]) - 1) * 100)[1, 1]
## [1] 21.47127
((exp(coef(mod2)["demo_age"]) - 1) * 100)[1, 1]
## [1] -3.527711
intervals(mod2)
## Approximate 95% confidence intervals
##
##  Fixed effects:
##                    lower        est.       upper
## (Intercept)   7.66872007  8.38243060  9.09614113
## EOS_ControlB -0.89065916 -0.66914112 -0.44762308
## PhaseEOS_1    0.34637414  0.61485986  0.88334557
## PhaseEOS_2   -0.07397813  0.19450758  0.46299329
## demo_age     -0.05881012 -0.03591438 -0.01301865
##
##  Random Effects:
##   Level: Subject
##                     lower      est.     upper
## sd((Intercept)) 0.2840166 0.4283855 0.6461388
##
##  Within-group standard error:
##     lower      est.     upper
## 0.6245670 0.7051691 0.7961731
```

A simplified model using only significant predictors on data without outliers has poorer AIC and BIC performance, as well as lower p-values in the Lilliefors (Kolmogorov-Smirnov) normality test. This indicates a worse performance compared to the full model.

##### 5.1.2.4. Model comparison LF

```
anova(mod1,mod2)
## Warning in anova.lme(mod1, mod2): fitted objects with different fixed effects.
## REML comparisons are not meaningful.
##      Model df      AIC      BIC    logLik   Test L.Ratio p-value
## mod1     1  9 413.5972 440.9881 -197.7986
## mod2     2  7 404.4282 425.8219 -195.2141 1 vs 2 5.16906  0.0754
```

The simple model (5.1.2.3.) shows better performance based on the BIC criterion.

#### 5.1.3. Frontal alpha asymmetry

##### 5.1.3.1. Boxplots Frontal alpha asymmetry

```
ggplot(fulldata, aes(x=(Phase), y = alpha_asymmetry, fill = (Phase)))+
  geom_boxplot()+
    scale_fill_manual(values=cbPalette) +
    facet_wrap(~EOS_Control)+
    theme(legend.position="none")+
    xlab("Phase") +
    ylab("Alpha asymmetry")
## Warning: Removed 55 rows containing non-finite outside the scale range
## (`stat_boxplot()`).
```

```
ggplot(fulldata4to6, aes(x=factor(EOS_Control), y = alpha_asymmetry, fill = factor(EOS_Control)))+
  geom_boxplot()+
    facet_wrap(~Session)+
    theme(legend.position="none")+
  scale_fill_manual(
    values = c("steelblue", "slategrey")
  ) +
    xlab("EOS or Control") +
    ylab("Alpha asymmetry")
```

##### 5.1.3.2. Full model Frontal alpha asymmetry

```
mod1 <- lme(alpha_asymmetry ~ EOS_Control + Phase + BMI + demo_age + demo_sex, random = ~ 1 | Subject, data = fulldata4to6, na.action = na.omit)

summary(mod1)
## Linear mixed-effects model fit by REML
##   Data: fulldata4to6
##        AIC      BIC    logLik
##   84.23738 111.6282 -33.11869
##
## Random effects:
##  Formula: ~1 | Subject
##         (Intercept)  Residual
## StdDev:  0.09219863 0.2553195
##
## Fixed effects:  alpha_asymmetry ~ EOS_Control + Phase + BMI + demo_age + demo_sex
##                     Value  Std.Error  DF    t-value p-value
## (Intercept)    0.20379524 0.18799848 129  1.0840260  0.2804
## EOS_ControlB  -0.04737897 0.04038298 129 -1.1732411  0.2429
## PhaseEOS_1     0.01737335 0.04913627 129  0.3535749  0.7242
## PhaseEOS_2    -0.05127810 0.04913627 129 -1.0435897  0.2986
## BMI           -0.01976955 0.00804749 129 -2.4566111  0.0154
## demo_age       0.00868671 0.00329972 129  2.6325618  0.0095
## demo_sexHomme  0.09249004 0.05412217  26  1.7089123  0.0994
##  Correlation:
##               (Intr) EOS_CB PEOS_1 PEOS_2 BMI    demo_g
## EOS_ControlB  -0.111
## PhaseEOS_1    -0.131  0.000
## PhaseEOS_2    -0.131  0.000  0.500
## BMI           -0.830  0.004  0.000  0.000
## demo_age      -0.237  0.017  0.000  0.000 -0.285
## demo_sexHomme -0.063 -0.031  0.000  0.000 -0.107  0.091
##
## Standardized Within-Group Residuals:
##         Min          Q1         Med          Q3         Max
## -4.77471751 -0.46755488 -0.03002987  0.45494561  4.15724580
##
## Number of Observations: 162
## Number of Groups: 28
par(mfrow=c(1, 3))

plot(mod1)
```

plot(mod1$fitted, mod1$residuals)

qqnorm(mod1$residuals)

qqline(mod1$residuals, col="red")

hist(mod1$residuals)

plot(mod1, resid(., type = "p") ~ fitted(.) | EOS_Control, abline = 0)

plot(mod1, Subject ~ resid(.))

plot(mod1, alpha_asymmetry ~ fitted(.) | Subject, abline = c(0,1))

```
lillie.test(mod1$residuals)
##
##  Lilliefors (Kolmogorov-Smirnov) normality test
##
## data:  mod1$residuals
## D = 0.064375, p-value = 0.002523
```

The linear mixed-effects model analysis revealed significant associations between demographic predictor variables and alpha asymmetry. Whereas EOS_ControlB, PhaseEOS_1, and PhaseEOS_2 phases did not exhibit statistically significant effects on alpha asymmetry, BMI displayed a significant negative effect (p = 0.015), indicating that higher BMI values were associated with decreased alpha asymmetry. Conversely, age demonstrated a significant positive effect (p = 0.01), suggesting that older participants tended to exhibit higher alpha asymmetry. Sex had borderline statistically significant effects (p = 0.1) on alpha asymmetry, indicating an increase of alpha asymmetry in men. Variability in intercepts among subjects was characterized by a SD of 0.0922, whereas residual variability had a SD of 0.255. Correlation analysis revealed low associations between predictor variables, indicating minimal multi-co-linearity issues. Moreover, standardized within-group residuals exhibited acceptable distributional properties, but the Lilliefors (Kolmogorov-Smirnov) normality test on residuals yielded a p-value of 0.003, indicating significant evidence against the null hypothesis of normality. This suggests potential deviations from normality in the residuals, which should be considered when interpreting the results of the model.

##### 5.1.3.3. Full model without outiers Frontal alpha asymmetry

```
# Histogram of logarithm of Alpha asymmetry  in fulldata4to6
hist(fulldata4to6$alpha_asymmetry)
```

```
 # Calculate quartiles and IQR
quartiles <- quantile(fulldata4to6$alpha_asymmetry, probs = c(0.25, 0.75), na.rm = FALSE)
IQR_val <- IQR(fulldata4to6$alpha_asymmetry)

 # Calculate lower and upper bounds for outlier detection
Lower <- quartiles[1] - 1.5 * IQR_val
Upper <- quartiles[2] + 1.5 * IQR_val

 # Subset data to remove outliers
data_no_outlier <- subset(fulldata4to6, alpha_asymmetry > Lower & alpha_asymmetry < Upper)

 # Print dimensions of data_no_outlier
dim(data_no_outlier)
## [1] 160  40
 # Histogram of logarithm of alpha_asymmetry in data_no_outlier
hist(data_no_outlier$alpha_asymmetry)
```

```
ggplot(data_no_outlier, aes(x=(Phase), y = alpha_asymmetry, fill = (Phase)))+
  geom_boxplot()+
    facet_wrap(~EOS_Control)+
    theme(legend.position="none")+
    xlab("Phase") +
    ylab("Alpha asymmetry")
```

```
ggplot(data_no_outlier, aes(x=factor(EOS_Control), y = alpha_asymmetry, fill = factor(EOS_Control)))+
  geom_boxplot()+
    facet_wrap(~Session)+
    theme(legend.position="none")+
  scale_fill_manual(
    values = c("steelblue", "slategrey")
  ) +
    xlab("EOS or Control") +
    ylab("Alpha asymmetry")
```

```
mod1.1 <- lme(alpha_asymmetry ~ EOS_Control + BMI + demo_age + demo_sex, random = ~ 1 | Subject, data = data_no_outlier, na.action = na.omit)

summary(mod1.1)
## Linear mixed-effects model fit by REML
##   Data: data_no_outlier
##        AIC      BIC   logLik
##   1.757563 22.92473 6.121218
##
## Random effects:
##  Formula: ~1 | Subject
##         (Intercept)  Residual
## StdDev:   0.1088867 0.1950958
##
## Fixed effects:  alpha_asymmetry ~ EOS_Control + BMI + demo_age + demo_sex
##                     Value  Std.Error  DF    t-value p-value
## (Intercept)    0.21850627 0.17823852 126  1.2259206  0.2225
## EOS_ControlB  -0.02155975 0.03157040 126 -0.6829102  0.4959
## BMI           -0.01923759 0.00776508 126 -2.4774479  0.0146
## demo_age       0.00752615 0.00319108 126  2.3584933  0.0199
## demo_sexHomme  0.06640128 0.05262208  26  1.2618522  0.2182
##  Correlation:
##               (Intr) EOS_CB BMI    demo_g
## EOS_ControlB  -0.087
## BMI           -0.839  0.009
## demo_age      -0.231 -0.002 -0.296
## demo_sexHomme -0.074 -0.023 -0.102  0.095
##
## Standardized Within-Group Residuals:
##         Min          Q1         Med          Q3         Max
## -2.58545262 -0.49553272 -0.05599594  0.46881043  2.57863667
##
## Number of Observations: 157
## Number of Groups: 28
par(mfrow=c(1, 3))

plot(mod1.1)
```

plot(mod1.1$residuals, mod1.1$fitted)

qqnorm(mod1.1$residuals)

qqline(mod1.1$residuals, col="red")

hist(mod1.1$residuals)

plot(mod1.1, resid(., type = "p") ~ fitted(.) | EOS_Control, abline = 0)

plot(mod1.1, Subject ~ resid(.))

plot(mod1.1, alpha_asymmetry ~ fitted(.) | Subject, abline = c(0,1))

```
lillie.test(mod1.1$residuals)
##
##  Lilliefors (Kolmogorov-Smirnov) normality test
##
## data:  mod1.1$residuals
## D = 0.062227, p-value = 0.005185
```

The model without outliers in alpha asymmetry has slightly different results. The SD for the intercept slightly increased for intercepts to 0.108 and decreased for residuals to 0.197. EOS_ControlB, PhaseEOS_1, PhaseEOS_2, and sex did not exhibit statistically significant effects on alpha asymmetry. BMI displayed a significant negative effect, indicating that higher BMI values were associated with decreased alpha asymmetry. Conversely, age demonstrated a significant positive effect, suggesting that older participants tended to exhibit higher alpha asymmetry. Correlation analysis revealed low associations between predictor variables, indicating minimal multi-co-linearity issues. Moreover, standardized within-group residuals exhibited acceptable distributional properties, but the Lilliefors (Kolmogorov-Smirnov) normality test on residuals yielded a p-value of 0.005, indicating significant evidence against the null hypothesis of normality. This suggests potential deviations from normality in the residuals, which should be considered when interpreting the results of the model.

##### 5.1.3.4. Simple model without outliers Frontal alpha asymmetry

```
mod2 <- lme(alpha_asymmetry ~ BMI + demo_age, random = ~ 1 | Subject, data = data_no_outlier, na.action = na.omit)

summary(mod2)
## Linear mixed-effects model fit by REML
##   Data: data_no_outlier
##        AIC      BIC   logLik
##   -9.35905 5.825713 9.679525
##
## Random effects:
##  Formula: ~1 | Subject
##         (Intercept)  Residual
## StdDev:   0.1114252 0.1945836
##
## Fixed effects:  alpha_asymmetry ~ BMI + demo_age
##                   Value  Std.Error  DF   t-value p-value
## (Intercept)  0.22434735 0.17943885 127  1.250272  0.2135
## BMI         -0.01818539 0.00783168 127 -2.322028  0.0218
## demo_age     0.00714357 0.00322052 127  2.218139  0.0283
##  Correlation:
##          (Intr) BMI
## BMI      -0.856
## demo_age -0.226 -0.290
##
## Standardized Within-Group Residuals:
##         Min          Q1         Med          Q3         Max
## -2.58176601 -0.45103124 -0.05870981  0.43934941  2.70296500
##
## Number of Observations: 157
## Number of Groups: 28
par(mfrow=c(1, 3))

plot(mod2)
```

plot(mod2$residuals, mod2$fitted)

qqnorm(mod2$residuals)

qqline(mod2$residuals, col="red")

hist(mod2$residuals)

plot(mod2, resid(., type = "p") ~ fitted(.) | EOS_Control, abline = 0)

plot(mod2, Subject ~ resid(.))

plot(mod2, alpha_asymmetry ~ fitted(.) | Subject, abline = c(0,1))

```
lillie.test(mod2$residuals)
##
##  Lilliefors (Kolmogorov-Smirnov) normality test
##
## data:  mod2$residuals
## D = 0.064511, p-value = 0.003086
intervals(mod2)
## Approximate 95% confidence intervals
##
##  Fixed effects:
##                     lower        est.        upper
## (Intercept) -0.1307297469  0.22434735  0.579424443
## BMI         -0.0336828843 -0.01818539 -0.002687903
## demo_age     0.0007707331  0.00714357  0.013516407
##
##  Random Effects:
##   Level: Subject
##                      lower      est.     upper
## sd((Intercept)) 0.07268175 0.1114252 0.1708209
##
##  Within-group standard error:
##     lower      est.     upper
## 0.1723053 0.1945836 0.2197423
```

The simplified model using only significant predictors on data without outliers has better AIC, BIC, and log likelihood performance, but lower p-values in the Lilliefors (Kolmogorov-Smirnov) normality test.

Since both models have residuals violating normality, we will use this with better performance on other criteria.

##### 5.1.3.5. Model comparison

```
# Extract relevant information from each model
model_names <- c("Model 1", "Model 2", "Model 3")
model_info <- sapply(list(mod1, mod1.1, mod2), extract_model_info)

# Create a data frame to store the information
model_comparison <- data.frame(Model = model_names,
                                Fixed_Effects_added = model_info[4,],
                                Random_Effects = model_info[5,],
                                Model_fit_AIC = model_info[1,],
                                Model_fit_BIC = model_info[2,],
                                Model_fit_logLik = model_info[3,])


# Print the table
print(model_comparison)
##     Model
## 1 Model 1
## 2 Model 2
## 3 Model 3
##                                                               Fixed_Effects_added
## 1 (Intercept), EOS_ControlB, PhaseEOS_1, PhaseEOS_2, BMI, demo_age, demo_sexHomme
## 2                         (Intercept), EOS_ControlB, BMI, demo_age, demo_sexHomme
## 3                                                      (Intercept), BMI, demo_age
##   Random_Effects     Model_fit_AIC    Model_fit_BIC  Model_fit_logLik
## 1    (Intercept)  84.2373800646762 111.628206116949 -33.1186900323381
## 2    (Intercept)  1.75756342684247 22.9247270727664  6.12121828657877
## 3    (Intercept) -9.35904975589375  5.8257132561744  9.67952487794687
anova(mod1.1, mod2)
## Warning in anova.lme(mod1.1, mod2): fitted objects with different fixed
## effects. REML comparisons are not meaningful.
##        Model df       AIC       BIC   logLik   Test  L.Ratio p-value
## mod1.1     1  7  1.757563 22.924727 6.121218
## mod2       2  5 -9.359050  5.825713 9.679525 1 vs 2 7.116613  0.0285
```

The simple model (5.1.3.4.) shows better performance based on the BIC criterion.

#### 5.1.4. Theta/Beta FP1

##### 5.1.4.1. Boxplots Frontal Theta/Beta FP1

```
ggplot(fulldata, aes(x=(Phase), y = ThBt1, fill = (Phase)))+
  geom_boxplot()+
  scale_fill_manual(values=cbPalette) +
    facet_wrap(~EOS_Control)+
    theme(legend.position="none")+
    xlab("Phase") +
    ylab("Theta/Beta left hemisphere")
## Warning: Removed 55 rows containing non-finite outside the scale range
## (`stat_boxplot()`).
```

```
ggplot(fulldata4to6, aes(x=factor(EOS_Control), y = ThBt1, fill = factor(EOS_Control)))+
  geom_boxplot()+
    facet_wrap(~Session)+
    theme(legend.position="none")+
  scale_fill_manual(
    values = c("steelblue", "slategrey")
  ) +
    xlab("EOS or Control") +
    ylab("Theta/Beta left hemisphere")
```

##### 5.1.4.2. Full model Frontal Theta/Beta FP1

```
mod1 <- lme(log(ThBt1) ~ EOS_Control + Phase + BMI + demo_age + demo_sex, random = ~ 1 | Subject, data = fulldata4to6, na.action = na.omit)

summary(mod1)
## Linear mixed-effects model fit by REML
##   Data: fulldata4to6
##        AIC     BIC    logLik
##   296.4372 323.828 -139.2186
##
## Random effects:
##  Formula: ~1 | Subject
##         (Intercept) Residual
## StdDev:   0.5391039 0.444398
##
## Fixed effects:  log(ThBt1) ~ EOS_Control + Phase + BMI + demo_age + demo_sex
##                    Value Std.Error  DF    t-value p-value
## (Intercept)    1.4975657 0.7312372 129  2.0479889  0.0426
## EOS_ControlB  -0.0286921 0.0709382 129 -0.4044665  0.6865
## PhaseEOS_1     0.1607433 0.0855244 129  1.8795019  0.0624
## PhaseEOS_2     0.0501445 0.0855244 129  0.5863180  0.5587
## BMI            0.0267628 0.0320997 129  0.8337389  0.4060
## demo_age      -0.0020814 0.0132725 129 -0.1568199  0.8756
## demo_sexHomme -0.0818130 0.2196049  26 -0.3725463  0.7125
##  Correlation:
##               (Intr) EOS_CB PEOS_1 PEOS_2 BMI    demo_g
## EOS_ControlB  -0.041
## PhaseEOS_1    -0.058  0.000
## PhaseEOS_2    -0.058  0.000  0.500
## BMI           -0.836 -0.002  0.000  0.000
## demo_age      -0.221  0.001  0.000  0.000 -0.312
## demo_sexHomme -0.076 -0.018  0.000  0.000 -0.105  0.102
##
## Standardized Within-Group Residuals:
##         Min          Q1         Med          Q3         Max
## -2.88230510 -0.60478970  0.06847031  0.46090705  2.48899293
##
## Number of Observations: 162
## Number of Groups: 28
par(mfrow=c(1, 3))

plot(mod1)
```

plot(mod1$fitted, mod1$residuals)

qqnorm(mod1$residuals)

qqline(mod1$residuals, col="red")

hist(mod1$residuals)

plot(mod1, resid(., type = "p") ~ fitted(.) | EOS_Control, abline = 0)

plot(mod1, Subject ~ resid(.))

plot(mod1, ThBt1 ~ fitted(.) | Subject, abline = c(0,1))

```
lillie.test(mod1$residuals)
##
##  Lilliefors (Kolmogorov-Smirnov) normality test
##
## data:  mod1$residuals
## D = 0.078643, p-value = 5.192e-05
((exp(coef(mod1)["PhaseEOS_1"]) - 1) * 100)[1, 1]
## [1] 17.43835
```

The linear mixed-effects model analysis revealed significant associations between phase and log(Theta/Beta FP1). Whereas EOS_ControlB and PhaseEOS_2 did not exhibit statistically significant effects on alpha asymmetry, PhaseEOS_1 displayed a significant positive effect. We will construct a simpler model including phase as the only predictor.

##### 5.1.4.3. Simple model Frontal Theta/Beta FP1

```
mod1.1 <- lme(log(ThBt1) ~ Phase, random = ~ 1 | Subject, data = fulldata4to6, na.action = na.omit)

summary(mod1.1)
## Linear mixed-effects model fit by REML
##   Data: fulldata4to6
##        AIC      BIC    logLik
##   276.7211 292.1591 -133.3606
##
## Random effects:
##  Formula: ~1 | Subject
##         (Intercept)  Residual
## StdDev:   0.4937767 0.4457152
##
## Fixed effects:  log(ThBt1) ~ Phase
##                 Value  Std.Error  DF   t-value p-value
## (Intercept) 1.9960270 0.11106799 135 17.971217  0.0000
## PhaseEOS_1  0.1668371 0.08499455 135  1.962915  0.0517
## PhaseEOS_2  0.0454482 0.08499455 135  0.534718  0.5937
##  Correlation:
##            (Intr) PEOS_1
## PhaseEOS_1 -0.383
## PhaseEOS_2 -0.383  0.500
##
## Standardized Within-Group Residuals:
##         Min          Q1         Med          Q3         Max
## -2.89795482 -0.62096580  0.07889271  0.48334662  2.47027362
##
## Number of Observations: 165
## Number of Groups: 28
par(mfrow=c(1, 3))

plot(mod1.1)
```

plot(mod1.1$fitted, mod1.1$residuals)

qqnorm(mod1.1$residuals)

qqline(mod1.1$residuals, col="red")

hist(mod1.1$residuals)

plot(mod1.1, resid(., type = "p") ~ fitted(.) | EOS_Control, abline = 0)

plot(mod1.1, Subject ~ resid(.))

plot(mod1.1, ThBt1 ~ fitted(.) | Subject, abline = c(0,1))

```
lillie.test(mod1.1$residuals)
##
##  Lilliefors (Kolmogorov-Smirnov) normality test
##
## data:  mod1.1$residuals
## D = 0.078852, p-value = 3.914e-05
((exp(coef(mod1.1)["PhaseEOS_1"]) - 1) * 100)[1, 1]
## [1] 18.15618
```

The simplified model revealed significant associations with PhaseEOS_1 increasing values by 18.16 %. Demographic predictor variables and Theta/Beta FP1. However, the residual plot shows some outliers and normality is seriously violated (p < 0.001). We opted to search and remove outliers from data and re-running the analysis.

##### 5.1.4.4. Full model without outliers Frontal Theta/Beta FP1

```
# Histogram of logarithm of ThBt1 in fulldata4to6
hist(log(fulldata4to6$ThBt1))
```

```
# Calculate quartiles and IQR
quartiles <- quantile(fulldata4to6$ThBt1, probs = c(0.25, 0.75), na.rm = FALSE)
IQR_val <- IQR(fulldata4to6$ThBt1)

# Calculate lower and upper bounds for outlier detection
Lower <- quartiles[1] - 1.5 * IQR_val
Upper <- quartiles[2] + 1.5 * IQR_val

# Subset data to remove outliers
data_no_outlier <- subset(fulldata4to6, ThBt1 > Lower & ThBt1 < Upper)

# Print dimensions of data_no_outlier
dim(data_no_outlier)
## [1] 148  40
# Histogram of logarithm of ThBt1 in data_no_outlier
hist(log(data_no_outlier$ThBt1))
```

```
ggplot(data_no_outlier, aes(x=(Phase), y = ThBt1, fill = (Phase)))+
  geom_boxplot()+
    facet_wrap(~EOS_Control)+
    theme(legend.position="none")+
    xlab("Phase") +
    ylab("Theta/Beta left hemisphere")
```

```
ggplot(data_no_outlier, aes(x=factor(EOS_Control), y = ThBt1, fill = factor(EOS_Control)))+
  geom_boxplot()+
    facet_wrap(~Session)+
    theme(legend.position="none")+
  scale_fill_manual(
    values = c("steelblue", "slategrey")
  ) +
    xlab("EOS or Control") +
    ylab("Theta/Beta left hemisphere")
```

```
mod2 <- lme(log(ThBt1) ~ EOS_Control + Phase + BMI + demo_age + demo_sex, random = ~ 1 | Subject, data = data_no_outlier, na.action = na.omit)

summary(mod2)
## Linear mixed-effects model fit by REML
##   Data: data_no_outlier
##        AIC      BIC    logLik
##   206.5274 232.8727 -94.26372
##
## Random effects:
##  Formula: ~1 | Subject
##         (Intercept)  Residual
## StdDev:   0.2478407 0.3833166
##
## Fixed effects:  log(ThBt1) ~ EOS_Control + Phase + BMI + demo_age + demo_sex
##                    Value Std.Error  DF    t-value p-value
## (Intercept)    1.2736706 0.4346121 113  2.9305919  0.0041
## EOS_ControlB  -0.1003608 0.0643491 113 -1.5596299  0.1216
## PhaseEOS_1     0.1557898 0.0783629 113  1.9880555  0.0492
## PhaseEOS_2     0.0712829 0.0783072 113  0.9102986  0.3646
## BMI            0.0286775 0.0179849 113  1.5945323  0.1136
## demo_age      -0.0026699 0.0073103 113 -0.3652236  0.7156
## demo_sexHomme  0.0309826 0.1178316  25  0.2629397  0.7948
##  Correlation:
##               (Intr) EOS_CB PEOS_1 PEOS_2 BMI    demo_g
## EOS_ControlB  -0.052
## PhaseEOS_1    -0.091  0.000
## PhaseEOS_2    -0.126  0.009  0.500
## BMI           -0.853 -0.009 -0.006  0.031
## demo_age      -0.310 -0.018  0.014  0.010 -0.183
## demo_sexHomme -0.021 -0.011  0.001 -0.010 -0.121  0.034
##
## Standardized Within-Group Residuals:
##         Min          Q1         Med          Q3         Max
## -2.55371119 -0.70987093  0.08877249  0.66128865  2.10556703
##
## Number of Observations: 145
## Number of Groups: 27
par(mfrow=c(1, 3))

plot(mod2)
```

plot(mod2$fitted, mod2$residuals)

qqnorm(mod2$residuals)

qqline(mod2$residuals, col="red")

hist(mod2$residuals)

plot(mod2, resid(., type = "p") ~ fitted(.) | EOS_Control, abline = 0)

plot(mod2, Subject ~ resid(.))

plot(mod2, ThBt1 ~ fitted(.) | Subject, abline = c(0,1))

```
lillie.test(mod2$residuals)
##
##  Lilliefors (Kolmogorov-Smirnov) normality test
##
## data:  mod2$residuals
## D = 0.047457, p-value = 0.1155
((exp(coef(mod2)["PhaseEOS_1"]) - 1) * 100)[1, 1]
## [1] 16.85805
```

The full model on data without outliers revealed significant associations between PhaseEOS_2 and log(Theta/Beta FP1), similar to the model with outliers. However, compared to the model with outliers, this model has better AIC, BIC and log likelihood criteria, as well as the Liliefors (Kolmogorov-Smirnov) normality test on the residuals which did not show signs of normality violation.

We proceed to test the hypothesis on a simpler model involving only phase as a predictor and on data without outliers.

##### 5.1.4.5. Simple model without outliers Frontal Theta/Beta FP1

```
# Fit linear mixed-effects model

mod2.1 <- lme(log(ThBt1) ~ Phase, random = ~ 1 | Subject, data = data_no_outlier, na.action = na.omit)

summary(mod2.1)
## Linear mixed-effects model fit by REML
##   Data: data_no_outlier
##        AIC      BIC    logLik
##   185.7556 200.6393 -87.87781
##
## Random effects:
##  Formula: ~1 | Subject
##         (Intercept)  Residual
## StdDev:   0.2355318 0.3864383
##
## Fixed effects:  log(ThBt1) ~ Phase
##                 Value  Std.Error  DF   t-value p-value
## (Intercept) 1.8200735 0.07230998 119 25.170434  0.0000
## PhaseEOS_1  0.1634463 0.07817610 119  2.090745  0.0387
## PhaseEOS_2  0.0630681 0.07805557 119  0.807989  0.4207
##  Correlation:
##            (Intr) PEOS_1
## PhaseEOS_1 -0.541
## PhaseEOS_2 -0.553  0.501
##
## Standardized Within-Group Residuals:
##        Min         Q1        Med         Q3        Max
## -2.7297945 -0.6688449  0.1040754  0.6267018  2.0098212
##
## Number of Observations: 148
## Number of Groups: 27
par(mfrow=c(1, 3))

plot(mod2.1)
```

plot(mod2.1$fitted, mod2.1$residuals)

qqnorm(mod2.1$residuals)

qqline(mod2.1$residuals, col="red")

hist(mod2.1$residuals)

plot(mod2.1, resid(., type = "p") ~ fitted(.) | EOS_Control, abline = 0)

plot(mod2.1, Subject ~ resid(.))

plot(mod2.1, ThBt1 ~ fitted(.) | Subject, abline = c(0,1))

```
lillie.test(mod2.1$residuals)
##
##  Lilliefors (Kolmogorov-Smirnov) normality test
##
## data:  mod2.1$residuals
## D = 0.049727, p-value = 0.07429
((exp(coef(mod2.1)["PhaseEOS_1"]) - 1) * 100)[1, 1]
## [1] 17.75621
intervals(mod2.1)
## Approximate 95% confidence intervals
##
##  Fixed effects:
##                    lower       est.     upper
## (Intercept)  1.676892531 1.82007351 1.9632545
## PhaseEOS_1   0.008649806 0.16344629 0.3182428
## PhaseEOS_2  -0.091489762 0.06306806 0.2176259
##
##  Random Effects:
##   Level: Subject
##                     lower      est.     upper
## sd((Intercept)) 0.1557511 0.2355318 0.3561788
##
##  Within-group standard error:
##     lower      est.     upper
## 0.3405029 0.3864383 0.4385706
```

The simple model of the dataset without outliers indicates the impact of each phase on log(ThBt1), with PhaseEOS_1 demonstrating a statistically significant positive effect increasing values by 17.76%, whereas PhaseEOS_2 does not show a statistically significant effect. The variability in intercepts among subjects is a SD of 0.236. The residual variability is a SD of 0.386, representing the dispersion of data around the fixed effects estimates. The correlation matrix shows that PhaseEOS_1 and PhaseEOS_2 are correlated at 0.501, suggesting some degree of association between the two phases in influencing log(ThBt1). The standardized within-group residuals exhibit acceptable distributional properties, with values ranging from -2.730 to 2.010, indicating that the model adequately captures the variability in log(ThBt1) within each subject. The quartiles suggest a relatively symmetrical distribution around the median. The Lilliefors (Kolmogorov-Smirnot) normality test yielded a p-value of 0.07, which exceeds the conventional significance level of 0.05, but still lies within the borderline significance level of 0.1. Therefore, there is insufficient evidence to reject the null hypothesis that the residuals are normally distributed, but there might be some departure from normality in the data.

##### 5.1.4.6. Model comparison

```
# Extract relevant information from each model
model_names <- c("Model 1", "Model 2", "Model 3", "Model 4")
model_info <- sapply(list(mod1, mod1.1, mod2, mod2.1), extract_model_info)

# Create a data frame to store the information
model_comparison <- data.frame(Model = model_names,
                                Fixed_Effects_added = model_info[4,],
                                Random_Effects = model_info[5,],
                                Model_fit_AIC = model_info[1,],
                                Model_fit_BIC = model_info[2,],
                                Model_fit_logLik = model_info[3,])


# Print the table
print(model_comparison)
##     Model
## 1 Model 1
## 2 Model 2
## 3 Model 3
## 4 Model 4
##                                                               Fixed_Effects_added
## 1 (Intercept), EOS_ControlB, PhaseEOS_1, PhaseEOS_2, BMI, demo_age, demo_sexHomme
## 2                                             (Intercept), PhaseEOS_1, PhaseEOS_2
## 3 (Intercept), EOS_ControlB, PhaseEOS_1, PhaseEOS_2, BMI, demo_age, demo_sexHomme
## 4                                             (Intercept), PhaseEOS_1, PhaseEOS_2
##   Random_Effects    Model_fit_AIC    Model_fit_BIC  Model_fit_logLik
## 1    (Intercept) 296.437214364928 323.828040417201 -139.218607182464
## 2    (Intercept) 276.721145073091 292.159126749253 -133.360572536546
## 3    (Intercept) 206.527438175439 232.872721341854 -94.2637190877196
## 4    (Intercept) 185.755623433188  200.63929214529 -87.8778117165938
```

The simple model without EEG outliers (5.1.4.5.) shows better performance based on the BIC criterion.

#### 5.1.5. Theta/Beta FP2

##### 5.1.5.1. Boxplots Frontal Theta/Beta FP2

```
ggplot(fulldata, aes(x=(Phase), y = ThBt2, fill = (Phase)))+
  geom_boxplot()+
  scale_fill_manual(values=cbPalette) +
  facet_wrap(~EOS_Control)+
  theme(legend.position="none")+
  xlab("Phase") +
  ylab("Theta/Beta rigth hemisphere")
## Warning: Removed 55 rows containing non-finite outside the scale range
## (`stat_boxplot()`).
```

```
ggplot(fulldata4to6, aes(x=factor(EOS_Control), y = ThBt2, fill = factor(EOS_Control)))+
  geom_boxplot()+
    facet_wrap(~Session)+
    theme(legend.position="none")+
  scale_fill_manual(
    values = c("steelblue", "slategrey")
  ) +
    xlab("EOS or Control") +
    ylab("Theta/Beta right hemisphere")
```

##### 5.1.5.2. Full model Frontal Theta/Beta FP2

```
mod1 <- lme(log(ThBt2) ~ EOS_Control + Phase + BMI + demo_age + demo_sex, random = ~ 1 | Subject, data = fulldata4to6, na.action = na.omit)

summary(mod1)
## Linear mixed-effects model fit by REML
##   Data: fulldata4to6
##        AIC      BIC    logLik
##   285.7406 313.1315 -133.8703
##
## Random effects:
##  Formula: ~1 | Subject
##         (Intercept)  Residual
## StdDev:   0.5380002 0.4270834
##
## Fixed effects:  log(ThBt2) ~ EOS_Control + Phase + BMI + demo_age + demo_sex
##                    Value Std.Error  DF    t-value p-value
## (Intercept)    1.6871029 0.7259199 129  2.3240898  0.0217
## EOS_ControlB   0.0495549 0.0681872 129  0.7267468  0.4687
## PhaseEOS_1     0.1633665 0.0821922 129  1.9876144  0.0490
## PhaseEOS_2     0.0834255 0.0821922 129  1.0150045  0.3120
## BMI            0.0177829 0.0318726 129  0.5579377  0.5779
## demo_age      -0.0008537 0.0131902 129 -0.0647213  0.9485
## demo_sexHomme  0.0158606 0.2183096  26  0.0726519  0.9426
##  Correlation:
##               (Intr) EOS_CB PEOS_1 PEOS_2 BMI    demo_g
## EOS_ControlB  -0.039
## PhaseEOS_1    -0.057  0.000
## PhaseEOS_2    -0.057  0.000  0.500
## BMI           -0.835 -0.003  0.000  0.000
## demo_age      -0.222  0.000  0.000  0.000 -0.312
## demo_sexHomme -0.077 -0.018  0.000  0.000 -0.105  0.103
##
## Standardized Within-Group Residuals:
##         Min          Q1         Med          Q3         Max
## -2.92883836 -0.50152733 -0.05725695  0.62404423  2.54172556
##
## Number of Observations: 162
## Number of Groups: 28
par(mfrow=c(1, 3))

plot(mod1)
```

plot(mod1$fitted, mod1$residuals)

qqnorm(mod1$residuals)

qqline(mod1$residuals, col="red")

hist(mod1$residuals)

plot(mod1, resid(., type = "p") ~ fitted(.) | EOS_Control, abline = 0)

plot(mod1, Subject ~ resid(.))

plot(mod1, ThBt2 ~ fitted(.) | Subject, abline = c(0,1))

```
lillie.test(mod1$residuals)
##
##  Lilliefors (Kolmogorov-Smirnov) normality test
##
## data:  mod1$residuals
## D = 0.065279, p-value = 0.002028
((exp(coef(mod1)["PhaseEOS_1"]) - 1) * 100)[1, 1]
## [1] 17.74681
```

The linear mixed-effects model revealed significant fixed effects of PhaseEOS_1 on log-transformed Theta/Beta FP2 values, increasing them by 17.75%. Other predictors were not statistically significant. The random effects indicate variability among subjects with a SD of 0.538, and a residual variability SD of 0.427. Correlation analysis shows low associations between predictor variables. The standardized within-group residuals display acceptable distributional properties, but the Lilliefors (Holmogorov-Smirnov) test reveals a statistically significant violation from normality (p = 0.002).

We will construct a second model, only considering the phase variable.

##### 5.1.5.3. Simple model Frontal Theta/Beta FP2

```
mod1.1 <- lme(log(ThBt2) ~ Phase, random = ~ 1 | Subject, data = fulldata4to6, na.action = na.omit)

summary(mod1.1)
## Linear mixed-effects model fit by REML
##   Data: fulldata4to6
##      AIC     BIC   logLik
##   263.61 279.048 -126.805
##
## Random effects:
##  Formula: ~1 | Subject
##         (Intercept)  Residual
## StdDev:   0.5024791 0.4237744
##
## Fixed effects:  log(ThBt2) ~ Phase
##                 Value Std.Error  DF   t-value p-value
## (Intercept) 2.1055957 0.1108950 135 18.987287  0.0000
## PhaseEOS_1  0.1675830 0.0808106 135  2.073775  0.0400
## PhaseEOS_2  0.0793713 0.0808106 135  0.982189  0.3278
##  Correlation:
##            (Intr) PEOS_1
## PhaseEOS_1 -0.364
## PhaseEOS_2 -0.364  0.500
##
## Standardized Within-Group Residuals:
##        Min         Q1        Med         Q3        Max
## -2.8867324 -0.5007890 -0.0489496  0.6602786  2.6557489
##
## Number of Observations: 165
## Number of Groups: 28
par(mfrow=c(1, 3))

plot(mod1.1)
```

plot(mod1.1$fitted, mod1.1$residuals)

qqnorm(mod1.1$residuals)

qqline(mod1.1$residuals, col="red")

hist(mod1.1$residuals)

plot(mod1.1, resid(., type = "p") ~ fitted(.) | EOS_Control, abline = 0)

plot(mod1.1, Subject ~ resid(.))

plot(mod1.1, ThBt2 ~ fitted(.) | Subject, abline = c(0,1))

```
lillie.test(mod1.1$residuals)
##
##  Lilliefors (Kolmogorov-Smirnov) normality test
##
## data:  mod1.1$residuals
## D = 0.060602, p-value = 0.005338
((exp(coef(mod1.1)["PhaseEOS_1"]) - 1) * 100)[1, 1]
## [1] 18.24434
```

The simpler model shows improvement in AIC and BIC, but normality of residuals is still violated.

We opted for searching and removing outliers from the data and re-running the analysis.

##### 5.1.5.4. Full model without outliers Frontal Theta/Beta FP1

```
# Histogram of logarithm of ThBt2 in data_no_outlier
hist(log(data_no_outlier$ThBt2))
```

```
# Calculate quartiles and IQR
quartiles <- quantile(data_no_outlier$ThBt2, probs = c(0.25, 0.75), na.rm = FALSE)
IQR_val <- IQR(data_no_outlier$ThBt2)

# Calculate lower and upper bounds for outlier detection
Lower <- quartiles[1] - 1.5 * IQR_val
Upper <- quartiles[2] + 1.5 * IQR_val

# Subset data to remove outliers
data_no_outlier <- subset(data_no_outlier, ThBt2 > Lower & ThBt2 < Upper)

# Print dimensions of data_no_outlier
dim(data_no_outlier)
## [1] 143  40
# Histogram of logarithm of ThBt2 in data_no_outlier
hist(log(data_no_outlier$ThBt2))
```

```
ggplot(fulldata4to6, aes(x=factor(EOS_Control), y = ThBt2, fill = factor(EOS_Control)))+
  geom_boxplot()+
    facet_wrap(~Session)+
    theme(legend.position="none")+
  scale_fill_manual(
    values = c("steelblue", "slategrey")
  ) +
    xlab("EOS or Control") +
    ylab("Theta/Beta right hemisphere")
```

```
mod2 <- lme(log(ThBt2) ~ EOS_Control + Phase + BMI + demo_age + demo_sex, random = ~ 1 | Subject, data = data_no_outlier, na.action = na.omit)

summary(mod2)
## Linear mixed-effects model fit by REML
##   Data: data_no_outlier
##        AIC      BIC    logLik
##   218.8766 244.9572 -100.4383
##
## Random effects:
##  Formula: ~1 | Subject
##         (Intercept)  Residual
## StdDev:   0.2781785 0.4051655
##
## Fixed effects:  log(ThBt2) ~ EOS_Control + Phase + BMI + demo_age + demo_sex
##                    Value Std.Error  DF   t-value p-value
## (Intercept)    1.8267008 0.4793402 109  3.810865  0.0002
## EOS_ControlB  -0.0046592 0.0692482 109 -0.067283  0.9465
## PhaseEOS_1     0.0387632 0.0851568 109  0.455198  0.6499
## PhaseEOS_2     0.0328655 0.0828107 109  0.396876  0.6922
## BMI            0.0108807 0.0199299 109  0.545945  0.5862
## demo_age      -0.0039644 0.0080991 109 -0.489490  0.6255
## demo_sexHomme  0.0955533 0.1304941  25  0.732243  0.4708
##  Correlation:
##               (Intr) EOS_CB PEOS_1 PEOS_2 BMI    demo_g
## EOS_ControlB  -0.046
## PhaseEOS_1    -0.098  0.023
## PhaseEOS_2    -0.123  0.010  0.488
## BMI           -0.853 -0.009  0.015  0.032
## demo_age      -0.302 -0.026 -0.008  0.009 -0.191
## demo_sexHomme -0.026 -0.010  0.012 -0.011 -0.114  0.030
##
## Standardized Within-Group Residuals:
##          Min           Q1          Med           Q3          Max
## -3.144117405 -0.423904486 -0.006304835  0.639074774  1.936981979
##
## Number of Observations: 141
## Number of Groups: 27
par(mfrow=c(1, 3))

plot(mod2)
```

plot(mod2$fitted, mod2$residuals)

qqnorm(mod2$residuals)

qqline(mod2$residuals, col="red")

hist(mod2$residuals)

plot(mod2, resid(., type = "p") ~ fitted(.) | EOS_Control, abline = 0)

plot(mod2, Subject ~ resid(.))

plot(mod2, ThBt2 ~ fitted(.) | Subject, abline = c(0,1))

```
lillie.test(mod2$residuals)
##
##  Lilliefors (Kolmogorov-Smirnov) normality test
##
## data:  mod2$residuals
## D = 0.069704, p-value = 0.002108
((exp(coef(mod2)["PhaseEOS_1"]) - 1) * 100)[1, 1]
## [1] 3.952431
```

The full model without outliers has improved AIC, BIC, and log likelihood criteria. However, this model did not reveal any statistically significant effects of predictors. Normality of residuals is still violated (p = 0.002).

We can try modelling with a simpler model involving only phase.

##### 5.1.5.5. Simple model without outliers Frontal Theta/Beta FP2

```
mod2.1 <- lme(log(ThBt2) ~ Phase, random = ~ 1 | Subject, data = data_no_outlier, na.action = na.omit)

summary(mod2.1)
## Linear mixed-effects model fit by REML
##   Data: data_no_outlier
##        AIC      BIC    logLik
##   193.2089 207.9171 -91.60447
##
## Random effects:
##  Formula: ~1 | Subject
##         (Intercept)  Residual
## StdDev:   0.2716906 0.3991238
##
## Fixed effects:  log(ThBt2) ~ Phase
##                 Value  Std.Error  DF   t-value p-value
## (Intercept) 2.0107385 0.07836848 114 25.657490  0.0000
## PhaseEOS_1  0.0319411 0.08347761 114  0.382631  0.7027
## PhaseEOS_2  0.0289253 0.08067693 114  0.358532  0.7206
##  Correlation:
##            (Intr) PEOS_1
## PhaseEOS_1 -0.499
## PhaseEOS_2 -0.528  0.485
##
## Standardized Within-Group Residuals:
##         Min          Q1         Med          Q3         Max
## -3.20324481 -0.45178823  0.03897096  0.62803094  1.97572596
##
## Number of Observations: 143
## Number of Groups: 27
par(mfrow=c(1, 3))

plot(mod2.1)
```

plot(mod2.1$fitted, mod2.1$residuals)

qqnorm(mod2.1$residuals)

qqline(mod2.1$residuals, col="red")

hist(mod2.1$residuals)

plot(mod2.1, resid(., type = "p") ~ fitted(.) | EOS_Control, abline = 0)

plot(mod2.1, Subject ~ resid(.))

plot(mod2.1, ThBt2 ~ fitted(.) | Subject, abline = c(0,1))

```
lillie.test(mod2.1$residuals)
##
##  Lilliefors (Kolmogorov-Smirnov) normality test
##
## data:  mod2.1$residuals
## D = 0.070419, p-value = 0.0016
((exp(coef(mod2.1)["PhaseEOS_1"]) - 1) * 100)[1, 1]
## [1] 3.245674
intervals(mod2.1)
## Approximate 95% confidence intervals
##
##  Fixed effects:
##                  lower       est.     upper
## (Intercept)  1.8554912 2.01073854 2.1659859
## PhaseEOS_1  -0.1334274 0.03194115 0.1973097
## PhaseEOS_2  -0.1308951 0.02892527 0.1887456
##
##  Random Effects:
##   Level: Subject
##                     lower      est.     upper
## sd((Intercept)) 0.1811075 0.2716906 0.4075799
##
##  Within-group standard error:
##     lower      est.     upper
## 0.3503652 0.3991238 0.4546679
```

The simpler model above has better AIC and BIC criteria, but also did not reveal any significant relationships and the Lilliefors (Kolmogorov-Smirnov) normality test again shows evidence for significant violations of normality in residuals (p = 0.002).

##### 5.1.5.6. Model comparison

```
# Extract relevant information from each model
model_names <- c("Model 1", "Model 2", "Model 3", "Model 4")
model_info <- sapply(list(mod1, mod1.1, mod2, mod2.1), extract_model_info)

# Create a data frame to store the information
model_comparison <- data.frame(Model = model_names,
                                Fixed_Effects_added = model_info[4,],
                                Random_Effects = model_info[5,],
                                Model_fit_AIC = model_info[1,],
                                Model_fit_BIC = model_info[2,],
                                Model_fit_logLik = model_info[3,])


# Print the table
print(model_comparison)
##     Model
## 1 Model 1
## 2 Model 2
## 3 Model 3
## 4 Model 4
##                                                               Fixed_Effects_added
## 1 (Intercept), EOS_ControlB, PhaseEOS_1, PhaseEOS_2, BMI, demo_age, demo_sexHomme
## 2                                             (Intercept), PhaseEOS_1, PhaseEOS_2
## 3 (Intercept), EOS_ControlB, PhaseEOS_1, PhaseEOS_2, BMI, demo_age, demo_sexHomme
## 4                                             (Intercept), PhaseEOS_1, PhaseEOS_2
##   Random_Effects    Model_fit_AIC    Model_fit_BIC  Model_fit_logLik
## 1    (Intercept) 285.740647599612 313.131473651885 -133.870323799806
## 2    (Intercept) 263.610041382961 279.048023059123 -126.805020691481
## 3    (Intercept) 218.876612585656 244.957170785214 -100.438306292828
## 4    (Intercept) 193.208935029929 207.917147142975 -91.6044675149645
```

The simple model without EEG outliers (5.1.5.5.) shows better performance based on the BIC criterion.

#### 5.1.6. Summary

- Full model for log rMSSD was selected based on BIC. rMSSD was influenced by the two intervention phase (75.52% and 59.12% increase compared to post-relaxation period, respectively), 5.1.1.2.
- Simple model for LF was selected based on BIC. Results show that LF was significantly affected by intervention type (-48.99 %), first half of relaxation (84.94 %), and by age (-3.48 %/year), 5.1.2.3.
- No EEG variables were associated with the intervention type.
- Simple model without outliers was selected for frontal alpha asymmetry based on BIC. Alpha asymmetry was rather influenced by BMI and age 5.1.3.4.
- Simple model without outliers was selected for Theta/Beta FP1 based on BIC. It showed, at the beginning of intervention the values were higher by 17.76 %, possibly residual effect after stress task, 5.1.4.5.
- Simple model without outliers was selected for Theta/Beta FP2 based on BIC. The model did not reveal any statistically significant relationship with predictors, 5.1.5.5.

The models exhibit acceptable distributional properties indicated by standardized within-group residuals. However, the normality of residuals was evidenced in model for rMSSD, but violated for all others (in models for LF, Alpha asymmetry, Theta/Beta FP1 and Theta/Beta FP2). Therefore, the reliability and confidence intervals derived from these models may be affected.

### 5.2. Subjective and physiological values

Physiological values are then analyzed in relation to subjective values:

- **subjective stress;**
- **positive affect;**
- **negative affect;**
- **sleepiness.**

#### 5.2.1. Subjective stress

##### 5.2.1.1. Boxplots Frontal Subjective stress

```
ggplot(fulldata4to6, aes(x=factor(EOS_Control), y = POST_EOS_stress, fill = factor(EOS_Control)))+
  geom_boxplot()+
    facet_wrap(~Session)+
    theme(legend.position="none")+
  scale_fill_manual(
    values = c("steelblue", "slategrey")
  ) +
    xlab("EOS or Control") +
    ylab("Subjective stress (post-intervention)")
## Warning: Removed 3 rows containing non-finite outside the scale range
## (`stat_boxplot()`).
```

##### 5.2.1.2. Full model Subjective stress - baseline

```
# baseline

mod1 <- lme(log(POST_EOS_stress) ~ EOS_Control + Phase + baseline_stress_state + HRV_RMSSD + HRV_lf + alpha_asymmetry + ThBt1 + ThBt2, random = ~ 1 | Subject, data = fulldata4to6, na.action = na.omit)

summary(mod1)
## Linear mixed-effects model fit by REML
##   Data: fulldata4to6
##        AIC      BIC    logLik
##   172.4429 208.4903 -74.22146
##
## Random effects:
##  Formula: ~1 | Subject
##         (Intercept)  Residual
## StdDev:   0.4750309 0.2373405
##
## Fixed effects:  log(POST_EOS_stress) ~ EOS_Control + Phase + baseline_stress_state +      HRV_RMSSD + HRV_lf + alpha_asymmetry + ThBt1 + ThBt2
##                            Value  Std.Error  DF   t-value p-value
## (Intercept)            0.7177682 0.13936409 122  5.150309  0.0000
## EOS_ControlB           0.2730849 0.04536099 122  6.020259  0.0000
## PhaseEOS_1             0.0194557 0.05612832 122  0.346629  0.7295
## PhaseEOS_2             0.0070731 0.05298173 122  0.133501  0.8940
## baseline_stress_state -0.0136949 0.02091056 122 -0.654926  0.5137
## HRV_RMSSD             -0.0006282 0.00112901 122 -0.556377  0.5790
## HRV_lf                 0.0000003 0.00001760 122  0.016569  0.9868
## alpha_asymmetry       -0.0779159 0.08381596 122 -0.929607  0.3544
## ThBt1                 -0.0053532 0.00502464 122 -1.065396  0.2888
## ThBt2                  0.0038944 0.00483511 122  0.805451  0.4221
##  Correlation:
##                       (Intr) EOS_CB PEOS_1 PEOS_2 bsln__ HRV_RM HRV_lf alph_s
## EOS_ControlB          -0.248
## PhaseEOS_1             0.119 -0.135
## PhaseEOS_2             0.044 -0.014  0.605
## baseline_stress_state -0.588  0.151 -0.060 -0.032
## HRV_RMSSD             -0.239 -0.174 -0.336 -0.416 -0.023
## HRV_lf                -0.119  0.485 -0.164  0.063  0.109 -0.571
## alpha_asymmetry        0.065  0.048  0.078  0.178 -0.068 -0.163  0.003
## ThBt1                 -0.088 -0.077  0.019  0.040  0.026  0.091 -0.128 -0.045
## ThBt2                 -0.117  0.062 -0.146 -0.109  0.061 -0.081  0.178  0.078
##                       ThBt1
## EOS_ControlB
## PhaseEOS_1
## PhaseEOS_2
## baseline_stress_state
## HRV_RMSSD
## HRV_lf
## alpha_asymmetry
## ThBt1
## ThBt2                 -0.785
##
## Standardized Within-Group Residuals:
##         Min          Q1         Med          Q3         Max
## -2.80730659 -0.58492922 -0.02357931  0.51558695  2.91152234
##
## Number of Observations: 159
## Number of Groups: 28
par(mfrow=c(1, 3))

plot(mod1)
```

plot(mod1$fitted, mod1$residuals)

qqnorm(mod1$residuals)

qqline(mod1$residuals, col="red")

hist(mod1$residuals)

plot(mod1, resid(., type = "p") ~ fitted(.) | EOS_Control, abline = 0)

plot(mod1, Subject ~ resid(.))

plot(mod1, POST_EOS_stress ~ fitted(.) | Subject, abline = c(0,1))

```
lillie.test(mod1$residuals)
##
##  Lilliefors (Kolmogorov-Smirnov) normality test
##
## data:  mod1$residuals
## D = 0.10604, p-value = 3.359e-09
((exp(coef(mod1)["PhaseEOS_1"]) - 1) * 100)[1, 1]
## [1] 1.964618
```

The linear mixed-effects model analysis revealed significant influence on log-transformed post-relaxation subjective stress from the intervention type. No other variables exhibited statistically significant effects. Moreover, the Lilliefors (Kolmogorov-Smirnov) normality test on residuals indicated a significant evidence against the null hypothesis of normality (p < 0.001). This suggests potential deviations from normality in the residuals which should be considered when interpreting the model results.

We will try to run the model through data without EEG outliers.

##### 5.2.1.3. Full model Subjective stress - baseline without EEG outliers

```
# without outliers
 # Calculate quartiles and IQR for alpha asymmetry

quartiles <- quantile(fulldata4to6$alpha_asymmetry, probs = c(0.25, 0.75), na.rm = FALSE)

IQR_val <- IQR(fulldata4to6$alpha_asymmetry)

 # Calculate lower and upper bounds for outlier detection

Lower <- quartiles[1] - 1.5 * IQR_val

Upper <- quartiles[2] + 1.5 * IQR_val

# Subset data to remove outliers

data_no_outlier <- subset(fulldata4to6, alpha_asymmetry > Lower & alpha_asymmetry < Upper)

# Calculate quartiles and IQR for Theta/Beta FP1

quartiles <- quantile(fulldata4to6$ThBt1, probs = c(0.25, 0.75), na.rm = FALSE)

IQR_val <- IQR(fulldata4to6$ThBt1)

# Calculate lower and upper bounds for outlier detection

Lower <- quartiles[1] - 1.5 * IQR_val

Upper <- quartiles[2] + 1.5 * IQR_val

# Subset data to remove outliers

data_no_outlier <- subset(data_no_outlier, ThBt1 > Lower & ThBt1 < Upper)

# Calculate quartiles and IQR for Theta/Beta FP2

quartiles <- quantile(data_no_outlier$ThBt2, probs = c(0.25, 0.75), na.rm = FALSE)

IQR_val <- IQR(data_no_outlier$ThBt2)

# Calculate lower and upper bounds for outlier detection

Lower <- quartiles[1] - 1.5 * IQR_val

Upper <- quartiles[2] + 1.5 * IQR_val

# Subset data to remove outliers

data_no_outlier <- subset(data_no_outlier, ThBt2 > Lower & ThBt2 < Upper)

# Print dimensions of data_no_outlier

dim(data_no_outlier)
## [1] 140  40
# baseline

mod2 <- lme(log(POST_EOS_stress) ~ EOS_Control + Phase + baseline_stress_state + HRV_RMSSD + HRV_lf + alpha_asymmetry + ThBt1 + ThBt2, random = ~ 1 | Subject, data = data_no_outlier, na.action = na.omit)

summary(mod2)
## Linear mixed-effects model fit by REML
##   Data: data_no_outlier
##        AIC      BIC    logLik
##   158.6446 192.5843 -67.32228
##
## Random effects:
##  Formula: ~1 | Subject
##         (Intercept)  Residual
## StdDev:   0.4616239 0.2376086
##
## Fixed effects:  log(POST_EOS_stress) ~ EOS_Control + Phase + baseline_stress_state +      HRV_RMSSD + HRV_lf + alpha_asymmetry + ThBt1 + ThBt2
##                            Value  Std.Error DF   t-value p-value
## (Intercept)            0.7458962 0.16227489 99  4.596498  0.0000
## EOS_ControlB           0.3230262 0.05178209 99  6.238184  0.0000
## PhaseEOS_1             0.0408157 0.06239332 99  0.654168  0.5145
## PhaseEOS_2             0.0380880 0.05776130 99  0.659404  0.5112
## baseline_stress_state  0.0065580 0.02221122 99  0.295257  0.7684
## HRV_RMSSD             -0.0011594 0.00135104 99 -0.858177  0.3929
## HRV_lf                 0.0000034 0.00002163 99  0.155465  0.8768
## alpha_asymmetry       -0.0516260 0.12464439 99 -0.414186  0.6796
## ThBt1                 -0.0223404 0.01071512 99 -2.084946  0.0396
## ThBt2                  0.0062954 0.00881872 99  0.713871  0.4770
##  Correlation:
##                       (Intr) EOS_CB PEOS_1 PEOS_2 bsln__ HRV_RM HRV_lf alph_s
## EOS_ControlB          -0.265
## PhaseEOS_1             0.108 -0.123
## PhaseEOS_2             0.001  0.061  0.575
## baseline_stress_state -0.577  0.210 -0.046  0.004
## HRV_RMSSD             -0.144 -0.305 -0.280 -0.420 -0.062
## HRV_lf                -0.143  0.541 -0.221  0.087  0.121 -0.621
## alpha_asymmetry        0.042  0.128  0.115  0.168 -0.008 -0.176  0.056
## ThBt1                 -0.246 -0.023 -0.050 -0.085  0.056  0.043 -0.106 -0.214
## ThBt2                 -0.267  0.075 -0.030  0.070  0.050 -0.131  0.206  0.175
##                       ThBt1
## EOS_ControlB
## PhaseEOS_1
## PhaseEOS_2
## baseline_stress_state
## HRV_RMSSD
## HRV_lf
## alpha_asymmetry
## ThBt1
## ThBt2                 -0.502
##
## Standardized Within-Group Residuals:
##         Min          Q1         Med          Q3         Max
## -2.80359815 -0.50036285  0.01367489  0.59233877  2.68189250
##
## Number of Observations: 135
## Number of Groups: 27
par(mfrow=c(1, 3))

plot(mod2)
```

plot(mod2$fitted, mod2$residuals)

qqnorm(mod2$residuals)

qqline(mod2$residuals, col="red")

hist(mod2$residuals)

plot(mod2, resid(., type = "p") ~ fitted(.) | EOS_Control, abline = 0)

plot(mod2, Subject ~ resid(.))

plot(mod2, POST_EOS_stress ~ fitted(.) | Subject, abline = c(0,1))

```
lillie.test(mod2$residuals)
##
##  Lilliefors (Kolmogorov-Smirnov) normality test
##
## data:  mod2$residuals
## D = 0.10486, p-value = 1.37e-07
((exp(coef(mod2)["PhaseEOS_1"]) - 1) * 100)[1, 1]
## [1] 4.166011
```

This model revealed a significant relationship with intervention type and with Theta/Beta FP1. We will construct a simplified model including only those predictors and data without EEG outliers.

##### 5.2.1.4. Simple model Subjective stress- baseline without EEG outliers

```
# baseline

mod2.1 <- lme(log(POST_EOS_stress) ~ EOS_Control + ThBt1, random = ~ 1 | Subject, data = data_no_outlier, na.action = na.omit)

summary(mod2.1)
## Linear mixed-effects model fit by REML
##   Data: data_no_outlier
##        AIC      BIC    logLik
##   89.85808 104.3473 -39.92904
##
## Random effects:
##  Formula: ~1 | Subject
##         (Intercept)  Residual
## StdDev:   0.4552799 0.2314304
##
## Fixed effects:  log(POST_EOS_stress) ~ EOS_Control + ThBt1
##                   Value  Std.Error  DF   t-value p-value
## (Intercept)   0.7791917 0.11162590 108  6.980384  0.0000
## EOS_ControlB  0.3293768 0.04064381 108  8.103985  0.0000
## ThBt1        -0.0218733 0.00842877 108 -2.595075  0.0108
##  Correlation:
##              (Intr) EOS_CB
## EOS_ControlB -0.211
## ThBt1        -0.564  0.089
##
## Standardized Within-Group Residuals:
##          Min           Q1          Med           Q3          Max
## -2.949936560 -0.610668202 -0.004745734  0.544398546  2.915228219
##
## Number of Observations: 137
## Number of Groups: 27
par(mfrow=c(1, 3))

plot(mod2.1)
```

plot(mod2.1$fitted, mod2.1$residuals)

qqnorm(mod2.1$residuals)

qqline(mod2.1$residuals, col="red")

hist(mod2.1$residuals)

plot(mod2.1, resid(., type = "p") ~ fitted(.) | EOS_Control, abline = 0)

plot(mod2.1, Subject ~ resid(.))

plot(mod2.1, POST_EOS_stress ~ fitted(.) | Subject, abline = c(0,1))

```
lillie.test(mod2.1$residuals)
##
##  Lilliefors (Kolmogorov-Smirnov) normality test
##
## data:  mod2.1$residuals
## D = 0.1122, p-value = 6.934e-09
((exp(coef(mod2.1)["EOS_ControlB"]) - 1) * 100)[1, 1]
## [1] 39.01015
((exp(coef(mod2.1)["ThBt1"]) - 1) * 100)[1, 1]
## [1] -2.163581
```

The simple model on data without EEG outliers revealed significant associations between intervention type and Theta/Beta FP1 ration on log-transformed post-relaxation subjective stress. The Control group displayed a significant increase by 39.01%. The Theta/Beta ratio in the left frontoparietal hemisphere had a negative relationship (-2.16 %). Variability in intercepts among subjects was characterized by a SD of 0.455, whereas residual variability had a SD of 0.231. Correlation analysis revealed low associations between predictor variables. Moreover, standardized within-group residuals exhibited acceptable distributional properties, but the Lilliefors (Kolmogorov-Smirnov) normality test on residuals yielded a p-value of 6.93 e-09, indicating significant evidence against the null hypothesis of normality. This suggests potential deviations from normality in the residuals, which should be considered when interpreting the model results.

##### 5.2.1.5. Full model Subjective stress - post-stress

```
# post-stress

mod3 <- lme(log(POST_EOS_stress) ~ EOS_Control + PRE_EOS_stress + Phase + HRV_RMSSD + HRV_lf + alpha_asymmetry + ThBt1 + ThBt2, random = ~ 1 | Subject, data = data_no_outlier, na.action = na.omit)

summary(mod3)
## Linear mixed-effects model fit by REML
##   Data: data_no_outlier
##        AIC      BIC   logLik
##   132.2854 166.2252 -54.1427
##
## Random effects:
##  Formula: ~1 | Subject
##         (Intercept)  Residual
## StdDev:   0.4069998 0.2148958
##
## Fixed effects:  log(POST_EOS_stress) ~ EOS_Control + PRE_EOS_stress + Phase +      HRV_RMSSD + HRV_lf + alpha_asymmetry + ThBt1 + ThBt2
##                       Value  Std.Error DF   t-value p-value
## (Intercept)      0.19725592 0.15899597 99  1.240635  0.2177
## EOS_ControlB     0.26732744 0.04675845 99  5.717200  0.0000
## PRE_EOS_stress   0.10992794 0.02024498 99  5.429887  0.0000
## PhaseEOS_1      -0.00232337 0.05691543 99 -0.040821  0.9675
## PhaseEOS_2       0.00178336 0.05261378 99  0.033895  0.9730
## HRV_RMSSD       -0.00015063 0.00122988 99 -0.122475  0.9028
## HRV_lf           0.00000574 0.00001941 99  0.295934  0.7679
## alpha_asymmetry -0.12067913 0.11327707 99 -1.065345  0.2893
## ThBt1           -0.01998821 0.00967834 99 -2.065252  0.0415
## ThBt2            0.01020351 0.00799310 99  1.276540  0.2048
##  Correlation:
##                 (Intr) EOS_CB PRE_EO PEOS_1 PEOS_2 HRV_RM HRV_lf alph_s ThBt1
## EOS_ControlB     0.003
## PRE_EOS_stress  -0.667 -0.205
## PhaseEOS_1       0.169 -0.083 -0.142
## PhaseEOS_2       0.087  0.085 -0.125  0.583
## HRV_RMSSD       -0.261 -0.319  0.144 -0.298 -0.430
## HRV_lf          -0.090  0.513  0.034 -0.220  0.082 -0.607
## alpha_asymmetry  0.109  0.152 -0.113  0.129  0.179 -0.190  0.053
## ThBt1           -0.227 -0.043  0.045 -0.053 -0.090  0.052 -0.112 -0.217
## ThBt2           -0.283  0.044  0.096 -0.041  0.056 -0.112  0.203  0.163 -0.499
##
## Standardized Within-Group Residuals:
##         Min          Q1         Med          Q3         Max
## -2.63810149 -0.38301939 -0.02322545  0.48387724  2.25634754
##
## Number of Observations: 135
## Number of Groups: 27
par(mfrow=c(1, 3))

plot(mod3)
```

plot(mod3$fitted, mod3$residuals)

qqnorm(mod3$residuals)

qqline(mod3$residuals, col="red")

hist(mod3$residuals)

plot(mod3, resid(., type = "p") ~ fitted(.) | EOS_Control, abline = 0)

plot(mod3, Subject ~ resid(.))

plot(mod3, POST_EOS_stress ~ fitted(.) | Subject, abline = c(0,1))

```
lillie.test(mod3$residuals)
##
##  Lilliefors (Kolmogorov-Smirnov) normality test
##
## data:  mod3$residuals
## D = 0.10168, p-value = 4.101e-07
((exp(coef(mod3)["EOS_ControlB"]) - 1) * 100)[1, 1]
## [1] 30.64682
((exp(coef(mod3)["PRE_EOS_stress"]) - 1) * 100)[1, 1]
## [1] 11.61976
((exp(coef(mod3)["alpha_asymmetry"]) - 1) * 100)[1, 1]
## [1] -11.36817
```

The model involving intervention type, phase, post-stress subjective stress and all physiology revealed significant relationships with intervention type, with post-stress subjective stress, and with Theta/Beta FP1. Residuals have violated normality (p < 0.001). We will construct a simpler model only using significant predictors.

##### 5.2.1.6. Simple model Subjective stress - post-stress

```
# post-stress

mod3.1 <- lme(log(POST_EOS_stress) ~ EOS_Control + PRE_EOS_stress + ThBt1, random = ~ 1 | Subject, data = data_no_outlier, na.action = na.omit)

summary(mod3.1)
## Linear mixed-effects model fit by REML
##   Data: data_no_outlier
##        AIC     BIC    logLik
##   72.36112 89.6123 -30.18056
##
## Random effects:
##  Formula: ~1 | Subject
##         (Intercept)  Residual
## StdDev:   0.3970767 0.2134044
##
## Fixed effects:  log(POST_EOS_stress) ~ EOS_Control + PRE_EOS_stress + ThBt1
##                      Value  Std.Error  DF   t-value p-value
## (Intercept)     0.27533878 0.13541208 105  2.033340  0.0445
## EOS_ControlB    0.27175878 0.03920899 105  6.931032  0.0000
## PRE_EOS_stress  0.10512999 0.01943578 105  5.409096  0.0000
## ThBt1          -0.01544841 0.00814975 105 -1.895569  0.0608
##  Correlation:
##                (Intr) EOS_CB PRE_EO
## EOS_ControlB    0.030
## PRE_EOS_stress -0.666 -0.245
## ThBt1          -0.510  0.011  0.097
##
## Standardized Within-Group Residuals:
##         Min          Q1         Med          Q3         Max
## -2.70081326 -0.42131913 -0.02128555  0.48357287  2.27824373
##
## Number of Observations: 135
## Number of Groups: 27
par(mfrow=c(1, 3))

plot(mod3.1)
```

plot(mod3.1$fitted, mod3.1$residuals)

qqnorm(mod3.1$residuals)

qqline(mod3.1$residuals, col="red")

hist(mod3.1$residuals)

plot(mod3.1, resid(., type = "p") ~ fitted(.) | EOS_Control, abline = 0)

plot(mod3.1, Subject ~ resid(.))

plot(mod3.1, POST_EOS_stress ~ fitted(.) | Subject, abline = c(0,1))

```
lillie.test(mod3.1$residuals)
##
##  Lilliefors (Kolmogorov-Smirnov) normality test
##
## data:  mod3.1$residuals
## D = 0.088862, p-value = 2.297e-05
((exp(coef(mod3.1)["EOS_ControlB"]) - 1) * 100)[1, 1]
## [1] 31.22704
((exp(coef(mod3.1)["PRE_EOS_stress"]) - 1) * 100)[1, 1]
## [1] 11.0855
((exp(coef(mod3.1)["ThBt1"]) - 1) * 100)[1, 1]
## [1] -1.532969
```

The model with intervention type, alpha asymmetry, and post-stress subjective stress state performed better on AIC, BIC, and log likelihood, but still had no normal residuals (p < 0.001). It revealed significant associations with intervention type (p < 0.001) and post-stress subjective stress (p < 0.001). Alpha asymmetry is borderline significant (p = 0.061).

##### 5.2.1.7. Full model Subjective stress - post-stress without EEG outliers

```
# no EEG outliers

# post-stress

mod4 <- lme(log(POST_EOS_stress) ~ EOS_Control + PRE_EOS_stress + Phase + HRV_RMSSD + HRV_lf + alpha_asymmetry + ThBt1 + ThBt2, random = ~ 1 | Subject, data = data_no_outlier, na.action = na.omit)

summary(mod4)
## Linear mixed-effects model fit by REML
##   Data: data_no_outlier
##        AIC      BIC   logLik
##   132.2854 166.2252 -54.1427
##
## Random effects:
##  Formula: ~1 | Subject
##         (Intercept)  Residual
## StdDev:   0.4069998 0.2148958
##
## Fixed effects:  log(POST_EOS_stress) ~ EOS_Control + PRE_EOS_stress + Phase +      HRV_RMSSD + HRV_lf + alpha_asymmetry + ThBt1 + ThBt2
##                       Value  Std.Error DF   t-value p-value
## (Intercept)      0.19725592 0.15899597 99  1.240635  0.2177
## EOS_ControlB     0.26732744 0.04675845 99  5.717200  0.0000
## PRE_EOS_stress   0.10992794 0.02024498 99  5.429887  0.0000
## PhaseEOS_1      -0.00232337 0.05691543 99 -0.040821  0.9675
## PhaseEOS_2       0.00178336 0.05261378 99  0.033895  0.9730
## HRV_RMSSD       -0.00015063 0.00122988 99 -0.122475  0.9028
## HRV_lf           0.00000574 0.00001941 99  0.295934  0.7679
## alpha_asymmetry -0.12067913 0.11327707 99 -1.065345  0.2893
## ThBt1           -0.01998821 0.00967834 99 -2.065252  0.0415
## ThBt2            0.01020351 0.00799310 99  1.276540  0.2048
##  Correlation:
##                 (Intr) EOS_CB PRE_EO PEOS_1 PEOS_2 HRV_RM HRV_lf alph_s ThBt1
## EOS_ControlB     0.003
## PRE_EOS_stress  -0.667 -0.205
## PhaseEOS_1       0.169 -0.083 -0.142
## PhaseEOS_2       0.087  0.085 -0.125  0.583
## HRV_RMSSD       -0.261 -0.319  0.144 -0.298 -0.430
## HRV_lf          -0.090  0.513  0.034 -0.220  0.082 -0.607
## alpha_asymmetry  0.109  0.152 -0.113  0.129  0.179 -0.190  0.053
## ThBt1           -0.227 -0.043  0.045 -0.053 -0.090  0.052 -0.112 -0.217
## ThBt2           -0.283  0.044  0.096 -0.041  0.056 -0.112  0.203  0.163 -0.499
##
## Standardized Within-Group Residuals:
##         Min          Q1         Med          Q3         Max
## -2.63810149 -0.38301939 -0.02322545  0.48387724  2.25634754
##
## Number of Observations: 135
## Number of Groups: 27
par(mfrow=c(1, 3))

plot(mod4)
```

plot(mod4$fitted, mod4$residuals)

qqnorm(mod4$residuals)

qqline(mod4$residuals, col="red")

hist(mod4$residuals)

plot(mod4, resid(., type = "p") ~ fitted(.) | EOS_Control, abline = 0)

plot(mod4, Subject ~ resid(.))

plot(mod4, POST_EOS_stress ~ fitted(.) | Subject, abline = c(0,1))

```
lillie.test(mod4$residuals)
##
##  Lilliefors (Kolmogorov-Smirnov) normality test
##
## data:  mod4$residuals
## D = 0.10168, p-value = 4.101e-07
((exp(coef(mod4)["EOS_ControlB"]) - 1) * 100)[1, 1]
## [1] 30.64682
((exp(coef(mod4)["PRE_EOS_stress"]) - 1) * 100)[1, 1]
## [1] 11.61976
((exp(coef(mod4)["alpha_asymmetry"]) - 1) * 100)[1, 1]
## [1] -11.36817
```

The full linear mixed-effect model involving intervention type, phase, post-stress subjective stress, and all physiology (without EEG outliers) revealed a significant relationship with intervention type, post-stress subjective stress, and Theta/Beta ratio in the left frontal hemisphere. Residuals are not normal (p < 0.001).

We will run a simplified model with significant predictors.

##### 5.2.1.8. Simple model Subjective stress - post-stress without EEG outliers

```
# no EEG outliers

# post-stress

mod4.1 <- lme(log(POST_EOS_stress) ~ EOS_Control + PRE_EOS_stress + ThBt1, random = ~ 1 | Subject, data = data_no_outlier, na.action = na.omit)

summary(mod4.1)
## Linear mixed-effects model fit by REML
##   Data: data_no_outlier
##        AIC     BIC    logLik
##   72.36112 89.6123 -30.18056
##
## Random effects:
##  Formula: ~1 | Subject
##         (Intercept)  Residual
## StdDev:   0.3970767 0.2134044
##
## Fixed effects:  log(POST_EOS_stress) ~ EOS_Control + PRE_EOS_stress + ThBt1
##                      Value  Std.Error  DF   t-value p-value
## (Intercept)     0.27533878 0.13541208 105  2.033340  0.0445
## EOS_ControlB    0.27175878 0.03920899 105  6.931032  0.0000
## PRE_EOS_stress  0.10512999 0.01943578 105  5.409096  0.0000
## ThBt1          -0.01544841 0.00814975 105 -1.895569  0.0608
##  Correlation:
##                (Intr) EOS_CB PRE_EO
## EOS_ControlB    0.030
## PRE_EOS_stress -0.666 -0.245
## ThBt1          -0.510  0.011  0.097
##
## Standardized Within-Group Residuals:
##         Min          Q1         Med          Q3         Max
## -2.70081326 -0.42131913 -0.02128555  0.48357287  2.27824373
##
## Number of Observations: 135
## Number of Groups: 27
par(mfrow=c(1, 3))

plot(mod4.1)
```

plot(mod4.1$fitted, mod4.1$residuals)

qqnorm(mod4.1$residuals)

qqline(mod4.1$residuals, col="red")

hist(mod4.1$residuals)

plot(mod4.1, resid(., type = "p") ~ fitted(.) | EOS_Control, abline = 0)

plot(mod4.1, Subject ~ resid(.))

plot(mod4.1, POST_EOS_stress ~ fitted(.) | Subject, abline = c(0,1))

```
lillie.test(mod4.1$residuals)
##
##  Lilliefors (Kolmogorov-Smirnov) normality test
##
## data:  mod4.1$residuals
## D = 0.088862, p-value = 2.297e-05
round(summary(mod4.1)$tTable[, c(1, 5)], 8)
##                      Value    p-value
## (Intercept)     0.27533878 0.04454072
## EOS_ControlB    0.27175878 0.00000000
## PRE_EOS_stress  0.10512999 0.00000040
## ThBt1          -0.01544841 0.06076683
intervals <- intervals(mod4.1)

# Define the transformation function
transform_func <- function(x) {
  (exp(x) - 1) * 100
}

# Apply the transformation to confidence intervals
transformed_intervals <- transform_func(intervals$fixed)

# Print the transformed confidence intervals
print(transformed_intervals)
##                     lower      est.       upper
## (Intercept)     0.6865084 31.697676 72.26019800
## EOS_ControlB   21.4114000 31.227042 41.83623973
## PRE_EOS_stress  6.8859755 11.085500 15.45002322
## ThBt1          -3.1113548 -1.532969  0.07112904
## attr(,"label")
## [1] "Fixed effects:"
```

The simple model on the data without EEG outliers revealed a significant influence of the intervention type and post-stress subjective stress and a borderline significant influence of Theta/Beta FP1. In this model, the Control group increased subjective stress by 31.23% (CI 21.41 % and 41.83 %), and post-stress subjective stress significantly increased post-relaxation subjective stress by 11.09% (CI 6.88 % and 15.45 %). The Theta/Beta ratio in the left hemisphere had a negative association (-1.53 %; CI -3.11% and 0.071 %) with borderline significance (p = 0.06). Variability in intercepts among subjects was characterized by a SD of 0.397, while residual variability had a SD of 0.213. Correlation analysis revealed low associations between predictor variables. Moreover, standardized within-group residuals exhibited acceptable distributional properties, but the Lilliefors (Kolmogorov-Smirnov) normality test on residuals yielded a p-value of 2.297 e-05, indicating significant evidence against the null hypothesis of normality. This suggests potential deviations from normality in the residuals, which should be considered when interpreting the results of the model.

The model with outliers also had alpha asymmetry with borderline significance; we will try incorporating it in the model.

##### 5.2.1.9. Simple model Subjective stress - post-stress without EEG outliers and alpha asymmetry

```
# post-stress

mod4.2 <- lme(log(POST_EOS_stress) ~ EOS_Control + PRE_EOS_stress + alpha_asymmetry + ThBt1, random = ~ 1 | Subject, data = data_no_outlier, na.action = na.omit)

summary(mod4.2)
## Linear mixed-effects model fit by REML
##   Data: data_no_outlier
##        AIC      BIC    logLik
##   75.22704 95.29978 -30.61352
##
## Random effects:
##  Formula: ~1 | Subject
##         (Intercept)  Residual
## StdDev:   0.3985052 0.2124828
##
## Fixed effects:  log(POST_EOS_stress) ~ EOS_Control + PRE_EOS_stress + alpha_asymmetry +      ThBt1
##                       Value  Std.Error  DF   t-value p-value
## (Intercept)      0.25762535 0.13595822 104  1.894886  0.0609
## EOS_ControlB     0.26367223 0.03952995 104  6.670189  0.0000
## PRE_EOS_stress   0.10733873 0.01945103 104  5.518408  0.0000
## alpha_asymmetry -0.14278564 0.10772069 104 -1.325517  0.1879
## ThBt1           -0.01373408 0.00822323 104 -1.670156  0.0979
##  Correlation:
##                 (Intr) EOS_CB PRE_EO alph_s
## EOS_ControlB     0.046
## PRE_EOS_stress  -0.668 -0.255
## alpha_asymmetry  0.102  0.156 -0.090
## ThBt1           -0.515 -0.015  0.110 -0.160
##
## Standardized Within-Group Residuals:
##         Min          Q1         Med          Q3         Max
## -2.64832403 -0.42088013 -0.04356169  0.51048101  2.25849242
##
## Number of Observations: 135
## Number of Groups: 27
par(mfrow=c(1, 3))

plot(mod4.2)
```

plot(mod4.2$fitted, mod4.2$residuals)

qqnorm(mod4.2$residuals)

qqline(mod4.2$residuals, col="red")

hist(mod4.2$residuals)

plot(mod4.2, resid(., type = "p") ~ fitted(.) | EOS_Control, abline = 0)

plot(mod4.2, Subject ~ resid(.))

plot(mod4.2, POST_EOS_stress ~ fitted(.) | Subject, abline = c(0,1))

```
lillie.test(mod4.2$residuals)
##
##  Lilliefors (Kolmogorov-Smirnov) normality test
##
## data:  mod4.2$residuals
## D = 0.08574, p-value = 5.568e-05
round(summary(mod4.2)$tTable[, c(1, 5)], 8)
##                       Value    p-value
## (Intercept)      0.25762535 0.06088471
## EOS_ControlB     0.26367223 0.00000000
## PRE_EOS_stress   0.10733873 0.00000025
## alpha_asymmetry -0.14278564 0.18790404
## ThBt1           -0.01373408 0.09789562
((exp(coef(mod4.2)["EOS_ControlB"]) - 1) * 100)[1, 1]
## [1] 30.17015
((exp(coef(mod4.2)["PRE_EOS_stress"]) - 1) * 100)[1, 1]
## [1] 11.33113
((exp(coef(mod4.2)["alpha_asymmetry"]) - 1) * 100)[1, 1]
## [1] -13.30601
((exp(coef(mod4.2)["ThBt1"]) - 1) * 100)[1, 1]
## [1] -1.36402
```

The model with alpha asymmetry again showed a significant effect of intervention type and post-stress subjective stress. The Theta/Beta ratio had small and borderline significant effect (-1.36 %, p = 0.1). Alpha asymmetry had a more pronounced effect (-13.31 %) on post-relaxation stress, but the effect was not statistically significant (p = 0.19).

The model has poorer performance characteristics; we will use the above model with intervention type, post-stress subjective stress, and Theta/Beta ratio in the left hemisphere (*5.2.1.8. Simple model Subjective stress - post-stress without EEG outliers*).

##### 5.2.1.10. Model comparison

```
# Extract relevant information from each model
model_names <- c("Model 1", "Model 2", "Model 3", "Model 4", "Model 5", "Model 6", "Model 7", "Model 8")
model_info <- sapply(list(mod1, mod2, mod2.1, mod3, mod3.1, mod4, mod4.1, mod4.2), extract_model_info)

# Create a data frame to store the information
model_comparison <- data.frame(Model = model_names,
                                Fixed_Effects_added = model_info[4,],
                                Random_Effects = model_info[5,],
                                Model_fit_AIC = model_info[1,],
                                Model_fit_BIC = model_info[2,],
                                Model_fit_logLik = model_info[3,])


# Print the table
print(model_comparison)
##     Model
## 1 Model 1
## 2 Model 2
## 3 Model 3
## 4 Model 4
## 5 Model 5
## 6 Model 6
## 7 Model 7
## 8 Model 8
##                                                                                                          Fixed_Effects_added
## 1 (Intercept), EOS_ControlB, PhaseEOS_1, PhaseEOS_2, baseline_stress_state, HRV_RMSSD, HRV_lf, alpha_asymmetry, ThBt1, ThBt2
## 2 (Intercept), EOS_ControlB, PhaseEOS_1, PhaseEOS_2, baseline_stress_state, HRV_RMSSD, HRV_lf, alpha_asymmetry, ThBt1, ThBt2
## 3                                                                                           (Intercept), EOS_ControlB, ThBt1
## 4        (Intercept), EOS_ControlB, PRE_EOS_stress, PhaseEOS_1, PhaseEOS_2, HRV_RMSSD, HRV_lf, alpha_asymmetry, ThBt1, ThBt2
## 5                                                                           (Intercept), EOS_ControlB, PRE_EOS_stress, ThBt1
## 6        (Intercept), EOS_ControlB, PRE_EOS_stress, PhaseEOS_1, PhaseEOS_2, HRV_RMSSD, HRV_lf, alpha_asymmetry, ThBt1, ThBt2
## 7                                                                           (Intercept), EOS_ControlB, PRE_EOS_stress, ThBt1
## 8                                                          (Intercept), EOS_ControlB, PRE_EOS_stress, alpha_asymmetry, ThBt1
##   Random_Effects    Model_fit_AIC    Model_fit_BIC  Model_fit_logLik
## 1    (Intercept) 172.442918913849 208.490274585195 -74.2214594569247
## 2    (Intercept) 158.644556981461 192.584321829088 -67.3222784907304
## 3    (Intercept) 89.8580839190271 104.347282918782 -39.9290419595135
## 4    (Intercept) 132.285395472371 166.225160319998 -54.1426977361854
## 5    (Intercept) 72.3611175138329 89.6123014530398 -30.1805587569165
## 6    (Intercept) 132.285395472371 166.225160319998 -54.1426977361854
## 7    (Intercept) 72.3611175138329 89.6123014530398 -30.1805587569165
## 8    (Intercept) 75.2270406520563 95.2997818052454 -30.6135203260282
```

Model “mod3.1” (5.2.1.6.) and “mod4.1” (5.2.1.8.) have both the lowest BIC.

#### 5.2.2. Positive Affect

##### 5.2.2.1. Boxplots Positive affect

```
ggplot(fulldata4to6, aes(x=factor(EOS_Control), y = POST_EOS_PA, fill = factor(EOS_Control)))+
  geom_boxplot()+
    facet_wrap(~Session)+
    theme(legend.position="none")+
  scale_fill_manual(
    values = c("steelblue", "slategrey")
  ) +
    xlab("EOS or Control") +
    ylab("Positive Affect (post-intervention)")
```

##### 5.2.2.2. Full model Positive affect - baseline and without EEG outliers

```
# baseline

mod1 <- lme(POST_EOS_PA ~ EOS_Control + Phase + baseline_PA + HRV_RMSSD + HRV_lf + alpha_asymmetry + ThBt1 + ThBt2, random = ~ 1 | Subject, data = data_no_outlier, na.action = na.omit)

summary(mod1)
## Linear mixed-effects model fit by REML
##   Data: data_no_outlier
##        AIC      BIC    logLik
##   858.6842 892.9086 -417.3421
##
## Random effects:
##  Formula: ~1 | Subject
##         (Intercept) Residual
## StdDev:    4.139914 4.032532
##
## Fixed effects:  POST_EOS_PA ~ EOS_Control + Phase + baseline_PA + HRV_RMSSD +      HRV_lf + alpha_asymmetry + ThBt1 + ThBt2
##                     Value Std.Error  DF   t-value p-value
## (Intercept)      6.058996  3.259005 102  1.859155  0.0659
## EOS_ControlB    -1.305505  0.840844 102 -1.552611  0.1236
## PhaseEOS_1      -0.213656  1.031354 102 -0.207161  0.8363
## PhaseEOS_2       0.239641  0.949279 102  0.252445  0.8012
## baseline_PA      0.606856  0.082610 102  7.346046  0.0000
## HRV_RMSSD       -0.024636  0.019484 102 -1.264395  0.2090
## HRV_lf           0.000361  0.000335 102  1.077433  0.2838
## alpha_asymmetry  1.797503  1.957299 102  0.918359  0.3606
## ThBt1           -0.053864  0.175306 102 -0.307258  0.7593
## ThBt2            0.323306  0.142248 102  2.272831  0.0251
##  Correlation:
##                 (Intr) EOS_CB PEOS_1 PEOS_2 bsl_PA HRV_RM HRV_lf alph_s ThBt1
## EOS_ControlB    -0.117
## PhaseEOS_1       0.105 -0.110
## PhaseEOS_2       0.029  0.044  0.576
## baseline_PA     -0.843 -0.020 -0.064 -0.046
## HRV_RMSSD       -0.220 -0.318 -0.279 -0.397  0.099
## HRV_lf          -0.073  0.538 -0.232  0.048  0.002 -0.582
## alpha_asymmetry  0.074  0.127  0.112  0.140 -0.040 -0.247  0.091
## ThBt1           -0.223 -0.005 -0.058 -0.072  0.061  0.062 -0.115 -0.211
## ThBt2           -0.153  0.055 -0.024  0.043 -0.032 -0.087  0.164  0.188 -0.535
##
## Standardized Within-Group Residuals:
##         Min          Q1         Med          Q3         Max
## -2.08079185 -0.48973411 -0.03020737  0.46701176  2.45374616
##
## Number of Observations: 138
## Number of Groups: 27
par(mfrow=c(1, 3))

plot(mod1)
```

plot(mod1$fitted, mod1$residuals)

qqnorm(mod1$residuals)

qqline(mod1$residuals, col="red")

hist(mod1$residuals)

plot(mod1, resid(., type = "p") ~ fitted(.) | EOS_Control, abline = 0)

plot(mod1, Subject ~ resid(.))

plot(mod1, POST_EOS_PA ~ fitted(.) | Subject, abline = c(0,1))

```
lillie.test(mod1$residuals)
##
##  Lilliefors (Kolmogorov-Smirnov) normality test
##
## data:  mod1$residuals
## D = 0.050398, p-value = 0.08702
```

The linear mixed-effects model analysis on the data without EEG outliers revealed a significant influence on post-relaxation subjective positive affect from baseline positive affect and Theta/Beta FP2. Intervention type was only borderline significant (p = 0.124). No other variables exhibited statistically significant effects. The Lilliefors (Kolmogorov-Smirnov) normality test on residuals yielded a p-value of 0.087, indicating borderline evidence against the null hypothesis of normality. This suggests potential deviations from normality in the residuals, which should be considered when interpreting the results of the model.

We will try to run the model using only those significant variables.

##### 5.2.2.3. Simple model Positive affect - baseline without EEG outliers

```
# baseline

mod1.1 <- lme(POST_EOS_PA ~ EOS_Control + baseline_PA + ThBt2, random = ~ 1 | Subject, data = fulldata4to6, na.action = na.omit)

summary(mod1.1)
## Linear mixed-effects model fit by REML
##   Data: fulldata4to6
##        AIC      BIC    logLik
##   968.2813 986.6568 -478.1406
##
## Random effects:
##  Formula: ~1 | Subject
##         (Intercept) Residual
## StdDev:    4.287798 3.848618
##
## Fixed effects:  POST_EOS_PA ~ EOS_Control + baseline_PA + ThBt2
##                  Value Std.Error  DF   t-value p-value
## (Intercept)   6.900578 2.6268182 131  2.626972  0.0096
## EOS_ControlB -1.858818 0.6155800 131 -3.019620  0.0030
## baseline_PA   0.619037 0.0765927 131  8.082189  0.0000
## ThBt2         0.080228 0.0440136 131  1.822808  0.0706
##  Correlation:
##              (Intr) EOS_CB bsl_PA
## EOS_ControlB -0.107
## baseline_PA  -0.920  0.012
## ThBt2        -0.125 -0.073 -0.060
##
## Standardized Within-Group Residuals:
##         Min          Q1         Med          Q3         Max
## -2.24557709 -0.51742364 -0.01759033  0.57798676  2.40196811
##
## Number of Observations: 162
## Number of Groups: 28
par(mfrow=c(1, 3))

plot(mod1.1)
```

plot(mod1.1$fitted, mod1.1$residuals)

qqnorm(mod1.1$residuals)

qqline(mod1.1$residuals, col="red")

hist(mod1.1$residuals)

plot(mod1.1, resid(., type = "p") ~ fitted(.) | EOS_Control, abline = 0)

plot(mod1.1, Subject ~ resid(.))

plot(mod1.1, POST_EOS_PA ~ fitted(.) | Subject, abline = c(0,1))

```
lillie.test(mod1.1$residuals)
##
##  Lilliefors (Kolmogorov-Smirnov) normality test
##
## data:  mod1.1$residuals
## D = 0.049635, p-value = 0.05258
```

The simpler model revealed a significant influence of the intervention type (-1.86, p = 0.007) and baseline values of positive affect (0.62, p < 0.001) and a borderline significant influence of Theta/Beta FP2 (0.08, p = 0.071). No other variables exhibited statistically significant effects. The Lilliefors (Kolmogorov-Smirnov) normality test on residuals yielded a p-value of 0.053, indicating violation of the null hypothesis of normality. This suggests potential deviations from normality in the residuals, which should be considered when interpreting the results of the model.

##### 5.2.2.4. Full model Positive affect - post-stress without EEG outliers

```
# post-stress

mod2 <- lme((POST_EOS_PA) ~ EOS_Control + PRE_EOS_PA + HRV_RMSSD + Phase + HRV_lf + alpha_asymmetry + ThBt1 + ThBt2, random = ~ 1 | Subject, data = data_no_outlier, na.action = na.omit)


summary(mod2)
## Linear mixed-effects model fit by REML
##   Data: data_no_outlier
##        AIC      BIC    logLik
##   831.7707 865.9951 -403.8854
##
## Random effects:
##  Formula: ~1 | Subject
##         (Intercept) Residual
## StdDev:    4.145685 3.555069
##
## Fixed effects:  (POST_EOS_PA) ~ EOS_Control + PRE_EOS_PA + HRV_RMSSD + Phase +      HRV_lf + alpha_asymmetry + ThBt1 + ThBt2
##                     Value Std.Error  DF   t-value p-value
## (Intercept)      5.136582 2.7163414 102  1.890993  0.0615
## EOS_ControlB    -2.260772 0.7519448 102 -3.006566  0.0033
## PRE_EOS_PA       0.725498 0.0756809 102  9.586275  0.0000
## HRV_RMSSD        0.005492 0.0179526 102  0.305921  0.7603
## PhaseEOS_1      -0.199149 0.9141986 102 -0.217840  0.8280
## PhaseEOS_2      -0.218120 0.8445842 102 -0.258258  0.7967
## HRV_lf          -0.000135 0.0003027 102 -0.447203  0.6557
## alpha_asymmetry  1.433544 1.7520142 102  0.818226  0.4151
## ThBt1            0.042549 0.1561810 102  0.272434  0.7858
## ThBt2            0.106364 0.1291369 102  0.823655  0.4121
##  Correlation:
##                 (Intr) EOS_CB PRE_EO HRV_RM PEOS_1 PEOS_2 HRV_lf alph_s ThBt1
## EOS_ControlB    -0.023
## PRE_EOS_PA      -0.807 -0.144
## HRV_RMSSD       -0.337 -0.343  0.240
## PhaseEOS_1       0.099 -0.102 -0.050 -0.285
## PhaseEOS_2       0.064  0.058 -0.088 -0.412  0.580
## HRV_lf           0.050  0.552 -0.155 -0.599 -0.221  0.064
## alpha_asymmetry  0.088  0.133 -0.055 -0.255  0.118  0.150  0.097
## ThBt1           -0.262 -0.022  0.099  0.081 -0.060 -0.080 -0.131 -0.211
## ThBt2           -0.040  0.084 -0.189 -0.132 -0.018  0.062  0.194  0.192 -0.536
##
## Standardized Within-Group Residuals:
##        Min         Q1        Med         Q3        Max
## -2.0501921 -0.6823689  0.0262225  0.5468070  2.4468689
##
## Number of Observations: 138
## Number of Groups: 27
par(mfrow=c(1, 3))

plot(mod2)
```

plot(mod2$fitted, mod2$residuals)

qqnorm(mod2$residuals)

qqline(mod2$residuals, col="red")

hist(mod2$residuals)

plot(mod2, resid(., type = "p") ~ fitted(.) | EOS_Control, abline = 0)

plot(mod2, Subject ~ resid(.))

plot(mod2, POST_EOS_PA ~ fitted(.) | Subject, abline = c(0,1))

```
lillie.test(mod2$residuals)
##
##  Lilliefors (Kolmogorov-Smirnov) normality test
##
## data:  mod2$residuals
## D = 0.05134, p-value = 0.07566
```

This model did not reveal any significant relationship with any of the physiological variables; only with post-stress positive affect and with intervention type. We will use a simplified model with only those predictors but on all data.

##### 5.2.2.5. Simple model Positive affect - post-stress

```
# post-stress

mod2.1 <- lme((POST_EOS_PA) ~ EOS_Control + PRE_EOS_PA, random = ~ 1 | Subject, data = fulldata4to6, na.action = na.omit)

summary(mod2.1)
## Linear mixed-effects model fit by REML
##   Data: fulldata4to6
##        AIC      BIC    logLik
##   922.5139 937.8584 -456.2569
##
## Random effects:
##  Formula: ~1 | Subject
##         (Intercept) Residual
## StdDev:    4.024206 3.351037
##
## Fixed effects:  (POST_EOS_PA) ~ EOS_Control + PRE_EOS_PA
##                  Value Std.Error  DF   t-value p-value
## (Intercept)   6.129865 2.0399405 132  3.004923  0.0032
## EOS_ControlB -1.844847 0.5347999 132 -3.449601  0.0008
## PRE_EOS_PA    0.732719 0.0635727 132 11.525678  0.0000
##  Correlation:
##              (Intr) EOS_CB
## EOS_ControlB -0.118
## PRE_EOS_PA   -0.910 -0.005
##
## Standardized Within-Group Residuals:
##         Min          Q1         Med          Q3         Max
## -2.19861637 -0.74503367  0.01544959  0.53499502  2.50405951
##
## Number of Observations: 162
## Number of Groups: 28
par(mfrow=c(1, 3))

plot(mod2.1)
```

plot(mod2.1$fitted, mod2.1$residuals)

qqnorm(mod2.1$residuals)

qqline(mod2.1$residuals, col="red")

hist(mod2.1$residuals)

plot(mod2.1, resid(., type = "p") ~ fitted(.) | EOS_Control, abline = 0)

plot(mod2.1, Subject ~ resid(.))

plot(mod2.1, POST_EOS_PA ~ fitted(.) | Subject, abline = c(0,1))

```
lillie.test(mod2.1$residuals)
##
##  Lilliefors (Kolmogorov-Smirnov) normality test
##
## data:  mod2.1$residuals
## D = 0.062262, p-value = 0.004144
intervals(mod2.1)
## Approximate 95% confidence intervals
##
##  Fixed effects:
##                   lower       est.      upper
## (Intercept)   2.0946611  6.1298651 10.1650690
## EOS_ControlB -2.9027336 -1.8448465 -0.7869594
## PRE_EOS_PA    0.6069655  0.7327186  0.8584717
##
##  Random Effects:
##   Level: Subject
##                    lower     est.    upper
## sd((Intercept)) 2.984858 4.024206 5.425462
##
##  Within-group standard error:
##    lower     est.    upper
## 2.971309 3.351037 3.779294
```

In this model, post-stress positive affect increased post-relaxation affect (0.733, p < 0.001) and intervention type had a significant influence (-1.845, p = 0.001). The normality of residuals is violated (p = 0.004). This model has worse AIC, BIC, log likelihood and lower p-value of the residual normality test compared to the full model without outliers.

We also included baseline positive affect and Theta/Beta FP2 which were significant in the baseline models.

##### 5.2.2.6. Simple model Positive affect - post-stress including baseline values and EEG without outliers

```
# post-stress

mod2.2 <- lme((POST_EOS_PA) ~ EOS_Control + PRE_EOS_PA + baseline_PA + ThBt2, random = ~ 1 | Subject, data = data_no_outlier, na.action = na.omit)

summary(mod2.2)
## Linear mixed-effects model fit by REML
##   Data: data_no_outlier
##        AIC      BIC    logLik
##   806.3023 826.5348 -396.1512
##
## Random effects:
##  Formula: ~1 | Subject
##         (Intercept) Residual
## StdDev:    3.967418 3.502297
##
## Fixed effects:  (POST_EOS_PA) ~ EOS_Control + PRE_EOS_PA + baseline_PA + ThBt2
##                  Value Std.Error  DF   t-value p-value
## (Intercept)   3.786179 2.6444044 107  1.431770  0.1551
## EOS_ControlB -2.114547 0.6124432 107 -3.452642  0.0008
## PRE_EOS_PA    0.621777 0.1056061 107  5.887702  0.0000
## baseline_PA   0.133001 0.1078066 107  1.233698  0.2200
## ThBt2         0.143149 0.1066612 107  1.342087  0.1824
##  Correlation:
##              (Intr) EOS_CB PRE_EO bsl_PA
## EOS_ControlB -0.061
## PRE_EOS_PA   -0.127 -0.079
## baseline_PA  -0.496  0.037 -0.737
## ThBt2        -0.303  0.001 -0.211  0.157
##
## Standardized Within-Group Residuals:
##         Min          Q1         Med          Q3         Max
## -2.08411302 -0.61136062  0.01348527  0.48792910  2.48950340
##
## Number of Observations: 138
## Number of Groups: 27
par(mfrow=c(1, 3))

plot(mod2.2)
```

plot(mod2.2$fitted, mod2.2$residuals)

qqnorm(mod2.2$residuals)

qqline(mod2.2$residuals, col="red")

hist(mod2.2$residuals)

plot(mod2.2, resid(., type = "p") ~ fitted(.) | EOS_Control, abline = 0)

plot(mod2.2, Subject ~ resid(.))

plot(mod2.2, POST_EOS_PA ~ fitted(.) | Subject, abline = c(0,1))

```
lillie.test(mod2.2$residuals)
##
##  Lilliefors (Kolmogorov-Smirnov) normality test
##
## data:  mod2.2$residuals
## D = 0.059709, p-value = 0.01878
intervals(mod2.2)
## Approximate 95% confidence intervals
##
##  Fixed effects:
##                    lower       est.      upper
## (Intercept)  -1.45604406  3.7861793  9.0284026
## EOS_ControlB -3.32864450 -2.1145473 -0.9004500
## PRE_EOS_PA    0.41242543  0.6217772  0.8311289
## baseline_PA  -0.08071324  0.1330008  0.3467148
## ThBt2        -0.06829475  0.1431485  0.3545918
##
##  Random Effects:
##   Level: Subject
##                   lower     est.    upper
## sd((Intercept)) 2.88685 3.967418 5.452449
##
##  Within-group standard error:
##    lower     est.    upper
## 3.066442 3.502297 4.000102
```

In this model, post-stress positive affect increased post-relaxation affect (0.622, p < 0.001) and intervention type had significant influence (-2.115, p = 0.001), but baseline positive affect had a weak (0.133, p = 0.220) and non-significant effect. The normality of residuals is violated (p = 0.02). This model has the best AIC, BIC, log likelihood among the models.

##### 5.2.2.7. Model comparison

```
# Extract relevant information from each model
model_names <- c("Model 1", "Model 2", "Model 3", "Model 4", "Model 5")
model_info <- sapply(list(mod1, mod1.1, mod2, mod2.1, mod2.2), extract_model_info)

# Create a data frame to store the information
model_comparison <- data.frame(Model = model_names,
                                Fixed_Effects_added = model_info[4,],
                                Random_Effects = model_info[5,],
                                Model_fit_AIC = model_info[1,],
                                Model_fit_BIC = model_info[2,],
                                Model_fit_logLik = model_info[3,])


# Print the table
print(model_comparison)
##     Model
## 1 Model 1
## 2 Model 2
## 3 Model 3
## 4 Model 4
## 5 Model 5
##                                                                                                Fixed_Effects_added
## 1 (Intercept), EOS_ControlB, PhaseEOS_1, PhaseEOS_2, baseline_PA, HRV_RMSSD, HRV_lf, alpha_asymmetry, ThBt1, ThBt2
## 2                                                                    (Intercept), EOS_ControlB, baseline_PA, ThBt2
## 3  (Intercept), EOS_ControlB, PRE_EOS_PA, HRV_RMSSD, PhaseEOS_1, PhaseEOS_2, HRV_lf, alpha_asymmetry, ThBt1, ThBt2
## 4                                                                            (Intercept), EOS_ControlB, PRE_EOS_PA
## 5                                                        (Intercept), EOS_ControlB, PRE_EOS_PA, baseline_PA, ThBt2
##   Random_Effects    Model_fit_AIC    Model_fit_BIC  Model_fit_logLik
## 1    (Intercept) 858.684220368348 892.908583535383 -417.342110184174
## 2    (Intercept) 968.281269365622 986.656839563784 -478.140634682811
## 3    (Intercept) 831.770709564207 865.995072731243 -403.885354782104
## 4    (Intercept) 922.513851779796 937.858372790898 -456.256925889898
## 5    (Intercept) 806.302330859446 826.534774756999 -396.151165429723
```

Model “mod2.2” (5.2.2.6.) has lowest BIC.However, because in this model Baseline PA and theta/beta FP2 are non-significant, we opted for “mod2.1” (5.2.2.5.) with lower number but all significant predictors.

#### 5.2.3. Negative Affect

##### 5.2.3.1. Boxplots Negative Affect

```
ggplot(fulldata4to6, aes(x=factor(EOS_Control), y = POST_EOS_NA, fill = factor(EOS_Control)))+
  geom_boxplot()+
    facet_wrap(~Session)+
    theme(legend.position="none")+
  scale_fill_manual(
    values = c("steelblue", "slategrey")
  ) +
    xlab("EOS or Control") +
    ylab("Negative Affect (post-intervention)")
```

##### 5.2.3.2. Full model Negative affect - baseline

```
# baseline

mod1 <- lme(log(POST_EOS_NA) ~ EOS_Control + Phase + baseline_NA + HRV_RMSSD  + HRV_lf + alpha_asymmetry + ThBt1 + ThBt2, random = ~ 1 | Subject, data = data_no_outlier, na.action = na.omit)


summary(mod1)
## Linear mixed-effects model fit by REML
##   Data: data_no_outlier
##         AIC       BIC   logLik
##   -37.99584 -3.771474 30.99792
##
## Random effects:
##  Formula: ~1 | Subject
##         (Intercept)  Residual
## StdDev:    0.262855 0.1034113
##
## Fixed effects:  log(POST_EOS_NA) ~ EOS_Control + Phase + baseline_NA + HRV_RMSSD +      HRV_lf + alpha_asymmetry + ThBt1 + ThBt2
##                      Value  Std.Error  DF   t-value p-value
## (Intercept)      2.6117734 0.09128708 102 28.610549  0.0000
## EOS_ControlB     0.0176036 0.02215034 102  0.794733  0.4286
## PhaseEOS_1       0.0053698 0.02718031 102  0.197561  0.8438
## PhaseEOS_2      -0.0059861 0.02511486 102 -0.238350  0.8121
## baseline_NA     -0.0004069 0.00359648 102 -0.113130  0.9101
## HRV_RMSSD        0.0010782 0.00054980 102  1.961037  0.0526
## HRV_lf          -0.0000174 0.00000908 102 -1.916073  0.0582
## alpha_asymmetry -0.0533765 0.05375201 102 -0.993015  0.3231
## ThBt1           -0.0056977 0.00464828 102 -1.225774  0.2231
## ThBt2           -0.0069733 0.00385591 102 -1.808463  0.0735
##  Correlation:
##                 (Intr) EOS_CB PEOS_1 PEOS_2 bsl_NA HRV_RM HRV_lf alph_s ThBt1
## EOS_ControlB    -0.213
## PhaseEOS_1       0.079 -0.109
## PhaseEOS_2       0.021  0.052  0.591
## baseline_NA     -0.690  0.136 -0.018 -0.028
## HRV_RMSSD       -0.241 -0.301 -0.308 -0.435  0.161
## HRV_lf          -0.001  0.533 -0.223  0.066 -0.098 -0.590
## alpha_asymmetry  0.039  0.126  0.137  0.179  0.001 -0.267  0.083
## ThBt1           -0.133 -0.031 -0.059 -0.080 -0.047  0.061 -0.118 -0.199
## ThBt2           -0.210  0.075 -0.028  0.066  0.031 -0.110  0.185  0.184 -0.512
##
## Standardized Within-Group Residuals:
##          Min           Q1          Med           Q3          Max
## -3.112515475 -0.407612038 -0.001373042  0.507097017  2.800146719
##
## Number of Observations: 138
## Number of Groups: 27
par(mfrow=c(1, 3))

plot(mod1)
```

plot(mod1$fitted, mod1$residuals)

qqnorm(mod1$residuals)

qqline(mod1$residuals, col="red")

hist(mod1$residuals)

plot(mod1, resid(., type = "p") ~ fitted(.) | EOS_Control, abline = 0)

plot(mod1, Subject ~ resid(.))

plot(mod1, ThBt2 ~ fitted(.) | Subject, abline = c(0,1))

```
lillie.test(mod1$residuals)
##
##  Lilliefors (Kolmogorov-Smirnov) normality test
##
## data:  mod1$residuals
## D = 0.15893, p-value < 2.2e-16
((exp(coef(mod1)["HRV_RMSSD"]) - 1) * 100)[1, 1]
## [1] 0.1078766
((exp(coef(mod1)["HRV_lf"]) - 1) * 100)[1, 1]
## [1] -0.00174059
((exp(coef(mod1)["ThBt2"]) - 1) * 100)[1, 1]
## [1] -0.694902
```

In the full model, significant predictors were HRV variables. Theta/Beta FP2 was borderline significant (p = 0.074) There was no influence of baseline values nor of intervention type. We will try a simpler model using only the significant predictors.

The linear mixed-effects model analysis revealed a significant influence of HRV variables rMSSD and LF and of Theta/Beta FP2. The Lilliefors (Kolmogorov-Smirnov) normality test on residuals indicates significant evidence (p < 0.001) against the null hypothesis of normality.

We will try to run a simpler model including only the significant predictors.

##### 5.2.3.3. Simple model Negative affect - baseline

```
mod1.1 <- lme(log(POST_EOS_NA) ~ HRV_RMSSD  + HRV_lf + ThBt2, random = ~ 1 | Subject, data = data_no_outlier, na.action = na.omit)


summary(mod1.1)
## Linear mixed-effects model fit by REML
##   Data: data_no_outlier
##         AIC       BIC   logLik
##   -89.71432 -72.23839 50.85716
##
## Random effects:
##  Formula: ~1 | Subject
##         (Intercept)  Residual
## StdDev:   0.2584693 0.1022897
##
## Fixed effects:  log(POST_EOS_NA) ~ HRV_RMSSD + HRV_lf + ThBt2
##                  Value  Std.Error  DF  t-value p-value
## (Intercept)  2.5938815 0.06202155 110 41.82226  0.0000
## HRV_RMSSD    0.0010826 0.00043679 110  2.47844  0.0147
## HRV_lf      -0.0000211 0.00000702 110 -3.01202  0.0032
## ThBt2       -0.0088755 0.00323326 110 -2.74507  0.0071
##  Correlation:
##           (Intr) HRV_RM HRV_lf
## HRV_RMSSD -0.270
## HRV_lf    -0.010 -0.639
## ThBt2     -0.451 -0.066  0.108
##
## Standardized Within-Group Residuals:
##        Min         Q1        Med         Q3        Max
## -3.1718220 -0.4232072 -0.0183035  0.5780356  2.8736371
##
## Number of Observations: 140
## Number of Groups: 27
par(mfrow=c(1, 3))

plot(mod1.1)
```

plot(mod1.1$fitted, mod1.1$residuals)

qqnorm(mod1.1$residuals)

qqline(mod1.1$residuals, col="red")

hist(mod1.1$residuals)

plot(mod1.1, resid(., type = "p") ~ fitted(.) | EOS_Control, abline = 0)

plot(mod1.1, Subject ~ resid(.))

plot(mod1.1, POST_EOS_NA ~ fitted(.) | Subject, abline = c(0,1))

```
lillie.test(mod1.1$residuals)
##
##  Lilliefors (Kolmogorov-Smirnov) normality test
##
## data:  mod1.1$residuals
## D = 0.1451, p-value = 1.228e-15
((exp(coef(mod1.1)["HRV_RMSSD"]) - 1) * 100)[1, 1]
## [1] 0.1083153
((exp(coef(mod1.1)["HRV_lf"]) - 1) * 100)[1, 1]
## [1] -0.002113753
((exp(coef(mod1.1)["ThBt2"]) - 1) * 100)[1, 1]
## [1] -0.8836234
```

In this model, negative affect seems to be statistically significantly influenced by Theta/Beta FP2 (- 0.884 %, p = 0.007) and by rMSSD (0.108 %, p = 0.02) and marginally influenced by LF (-0.002 %, p = 0.007). Normality of residuals is violated.

The residual plot shows some outliers that we may try to omit from analysis.

##### 5.2.3.4. Full model Negative affect - baseline without outliers

```
# Histogram of logarithm of ThBt1 in fulldata4to6

hist(log(fulldata4to6$POST_EOS_NA))
```

```
# Calculate quartiles and IQR

quartiles <- quantile(fulldata4to6$POST_EOS_NA, probs = c(0.25, 0.75), na.rm = FALSE)

IQR_val <- IQR(fulldata4to6$POST_EOS_NA)


# Calculate lower and upper bounds for outlier detection

Lower <- quartiles[1] - 1.5 * IQR_val

Upper <- quartiles[2] + 1.5 * IQR_val


# Subset data to remove outliers

data_no_outlier <- subset(data_no_outlier, POST_EOS_NA > Lower & POST_EOS_NA < Upper)


# Print dimensions of data_no_outlier

dim(data_no_outlier)
## [1] 128  40
# Histogram of logarithm of POST_EOS_NA in data_no_outlier

hist(log(data_no_outlier$POST_EOS_NA))
```

```
# Fit linear mixed-effects model

mod2 <- lme(log(POST_EOS_NA ) ~ EOS_Control + Phase + baseline_NA + HRV_RMSSD  + HRV_lf + alpha_asymmetry + ThBt1 + ThBt2, random = ~ 1 | Subject, data = data_no_outlier, na.action = na.omit)

summary(mod2)
## Linear mixed-effects model fit by REML
##   Data: data_no_outlier
##         AIC       BIC   logLik
##   -120.9431 -87.90004 72.47156
##
## Random effects:
##  Formula: ~1 | Subject
##         (Intercept)   Residual
## StdDev:    0.150473 0.07063897
##
## Fixed effects:  log(POST_EOS_NA) ~ EOS_Control + Phase + baseline_NA + HRV_RMSSD +      HRV_lf + alpha_asymmetry + ThBt1 + ThBt2
##                      Value  Std.Error DF  t-value p-value
## (Intercept)      2.4455938 0.06358882 91 38.45949  0.0000
## EOS_ControlB    -0.0107258 0.01581993 91 -0.67799  0.4995
## PhaseEOS_1       0.0145412 0.01929966 91  0.75344  0.4531
## PhaseEOS_2       0.0085353 0.01793535 91  0.47590  0.6353
## baseline_NA      0.0049478 0.00268736 91  1.84115  0.0689
## HRV_RMSSD        0.0001837 0.00042472 91  0.43248  0.6664
## HRV_lf          -0.0000047 0.00000655 91 -0.71812  0.4745
## alpha_asymmetry -0.0319883 0.03873036 91 -0.82592  0.4110
## ThBt1           -0.0111586 0.00339298 91 -3.28873  0.0014
## ThBt2            0.0037827 0.00283651 91  1.33356  0.1857
##  Correlation:
##                 (Intr) EOS_CB PEOS_1 PEOS_2 bsl_NA HRV_RM HRV_lf alph_s ThBt1
## EOS_ControlB    -0.218
## PhaseEOS_1       0.060 -0.101
## PhaseEOS_2       0.008  0.056  0.584
## baseline_NA     -0.745  0.145  0.013 -0.001
## HRV_RMSSD       -0.150 -0.303 -0.268 -0.410  0.038
## HRV_lf          -0.052  0.502 -0.230  0.064 -0.022 -0.618
## alpha_asymmetry  0.030  0.133  0.124  0.171 -0.021 -0.200  0.062
## ThBt1           -0.171  0.018 -0.104 -0.140  0.006  0.043 -0.090 -0.199
## ThBt2           -0.246  0.045  0.002  0.112  0.069 -0.169  0.209  0.213 -0.505
##
## Standardized Within-Group Residuals:
##         Min          Q1         Med          Q3         Max
## -2.79867780 -0.49655758 -0.01849345  0.58199725  2.17992059
##
## Number of Observations: 126
## Number of Groups: 26
par(mfrow=c(1, 3))

plot(mod2)
```

plot(mod2$fitted, mod2$residuals)

qqnorm(mod2$residuals)

qqline(mod2$residuals, col="red")

hist(mod2$residuals)

plot(mod2, resid(., type = "p") ~ fitted(.) | EOS_Control, abline = 0)

plot(mod2, Subject ~ resid(.))

plot(mod2, POST_EOS_NA ~ fitted(.) | Subject, abline = c(0,1))

```
lillie.test(mod2$residuals)
##
##  Lilliefors (Kolmogorov-Smirnov) normality test
##
## data:  mod2$residuals
## D = 0.11045, p-value = 6.954e-08
```

This model has better performance on AIC and BIC criteria. Both models violate normality of residuals. Deleting the outliers from negative affect completely changed the predictors. The significant predictors are Theta/Beta FP1 (p = 0.001), and baseline negative affect was borderline significant (p = 0.069).

In the former model, there was a significant relationship with Theta/Beta FP1 and rMSSD. We will try adding them to the model along with baseline negative affect and Theta/Beta FP2.

##### 5.2.3.5. Simple model Negative affect - baseline without outliers and including ThBt FP1, FP2, and rMSSD

```
# baseline

mod2.1 <- lme(log(POST_EOS_NA) ~ baseline_NA + ThBt1 + ThBt2 + HRV_RMSSD, random = ~ 1 | Subject, data = data_no_outlier, na.action = na.omit)

summary(mod2.1)
## Linear mixed-effects model fit by REML
##   Data: data_no_outlier
##        AIC       BIC   logLik
##   -175.476 -155.9055 94.73802
##
## Random effects:
##  Formula: ~1 | Subject
##         (Intercept)   Residual
## StdDev:   0.1504024 0.06948506
##
## Fixed effects:  log(POST_EOS_NA) ~ baseline_NA + ThBt1 + ThBt2 + HRV_RMSSD
##                  Value  Std.Error DF  t-value p-value
## (Intercept)  2.4413372 0.06106621 96 39.97853  0.0000
## baseline_NA  0.0048633 0.00260743 96  1.86519  0.0652
## ThBt1       -0.0115075 0.00322327 96 -3.57014  0.0006
## ThBt2        0.0044828 0.00265210 96  1.69028  0.0942
## HRV_RMSSD    0.0000830 0.00026802 96  0.30976  0.7574
##  Correlation:
##             (Intr) bsl_NA ThBt1  ThBt2
## baseline_NA -0.737
## ThBt1       -0.153 -0.017
## ThBt2       -0.284  0.102 -0.471
## HRV_RMSSD   -0.249  0.032 -0.154  0.022
##
## Standardized Within-Group Residuals:
##         Min          Q1         Med          Q3         Max
## -3.05319832 -0.51107669  0.03489518  0.53585790  2.25010751
##
## Number of Observations: 126
## Number of Groups: 26
par(mfrow=c(1, 3))

plot(mod2.1)
```

plot(mod2.1$fitted, mod2.1$residuals)

qqnorm(mod2.1$residuals)

qqline(mod2.1$residuals, col="red")

hist(mod2.1$residuals)

plot(mod2.1, resid(., type = "p") ~ fitted(.) | EOS_Control, abline = 0)

plot(mod2.1, Subject ~ resid(.))

plot(mod2.1, POST_EOS_NA ~ fitted(.) | Subject, abline = c(0,1))

```
lillie.test(mod2.1$residuals)
##
##  Lilliefors (Kolmogorov-Smirnov) normality test
##
## data:  mod2.1$residuals
## D = 0.1195, p-value = 2.612e-09
```

Significance has been found in Theta/Beta FP1 (p = 0.001), borderline significant in Theta/Beta FP2 (p = 0.09) and baseline negative affect (p = 0.065), but no significance was wound in rMSSD. This model has better performance on AIC and BIC. Normality of residuals is violated (p < 0.001). The next model will omit rMSSD.

##### 5.2.3.6. Simple model Negative affect - baseline without outliers and including ThBt FP1, and FP2

```
mod2.2 <- lme(log(POST_EOS_NA) ~ baseline_NA + ThBt1 + ThBt2, random = ~ 1 | Subject, data = data_no_outlier, na.action = na.omit)

summary(mod2.2)
## Linear mixed-effects model fit by REML
##   Data: data_no_outlier
##         AIC       BIC   logLik
##   -191.9964 -175.1722 101.9982
##
## Random effects:
##  Formula: ~1 | Subject
##         (Intercept)   Residual
## StdDev:   0.1508489 0.06911702
##
## Fixed effects:  log(POST_EOS_NA) ~ baseline_NA + ThBt1 + ThBt2
##                  Value  Std.Error DF  t-value p-value
## (Intercept)  2.4470608 0.05902798 97 41.45594  0.0000
## baseline_NA  0.0047763 0.00259771 97  1.83867  0.0690
## ThBt1       -0.0113591 0.00316857 97 -3.58494  0.0005
## ThBt2        0.0044676 0.00263821 97  1.69341  0.0936
##  Correlation:
##             (Intr) bsl_NA ThBt1
## baseline_NA -0.753
## ThBt1       -0.200 -0.012
## ThBt2       -0.287  0.101 -0.473
##
## Standardized Within-Group Residuals:
##         Min          Q1         Med          Q3         Max
## -3.05741210 -0.50567543  0.02385515  0.56288708  2.24532542
##
## Number of Observations: 126
## Number of Groups: 26
par(mfrow=c(1, 3))

plot(mod2.2)
```

plot(mod2.2$fitted, mod2.2$residuals)

qqnorm(mod2.2$residuals)

qqline(mod2.2$residuals, col="red")

hist(mod2.2$residuals)

plot(mod2.2, resid(., type = "p") ~ fitted(.) | EOS_Control, abline = 0)

plot(mod2.2, Subject ~ resid(.))

plot(mod2.2, POST_EOS_NA ~ fitted(.) | Subject, abline = c(0,1))

```
lillie.test(mod2.2$residuals)
##
##  Lilliefors (Kolmogorov-Smirnov) normality test
##
## data:  mod2.2$residuals
## D = 0.12358, p-value = 5.418e-10
((exp(coef(mod2.2)["baseline_NA"]) - 1) * 100)[1, 1]
## [1] 0.4787753
((exp(coef(mod2.2)["ThBt1"]) - 1) * 100)[1, 1]
## [1] -1.129487
((exp(coef(mod2.2)["ThBt2"]) - 1) * 100)[1, 1]
## [1] 0.4477554
```

A significant relationship was found with the Theta/Beta ratio in FP1 (-1.13%, p = 0.001), a borderline significant relationship with Theta/Beta in FP2 (0.45 %, p = 0.094) and with baseline negative affect (0.48%, p = 0.069). This model has better performance on AIC and BIC. Effect sizes are very small and normality of residuals is violated (p < 0.001).

##### 5.2.3.7. Full model Negative affect - post-stress

```
# post-stress

mod3 <- lme(log(POST_EOS_NA) ~  EOS_Control + Phase + PRE_EOS_NA + HRV_RMSSD  + HRV_lf + alpha_asymmetry + ThBt1 + ThBt2, random = ~ 1 | Subject, data = data_no_outlier, na.action = na.omit)

summary(mod3)
## Linear mixed-effects model fit by REML
##   Data: data_no_outlier
##         AIC       BIC   logLik
##   -141.1597 -108.1166 82.57983
##
## Random effects:
##  Formula: ~1 | Subject
##         (Intercept)   Residual
## StdDev:   0.1418792 0.06417864
##
## Fixed effects:  log(POST_EOS_NA) ~ EOS_Control + Phase + PRE_EOS_NA + HRV_RMSSD +      HRV_lf + alpha_asymmetry + ThBt1 + ThBt2
##                      Value  Std.Error DF  t-value p-value
## (Intercept)      2.3106915 0.05859425 91 39.43547  0.0000
## EOS_ControlB    -0.0044663 0.01437315 91 -0.31074  0.7567
## PhaseEOS_1       0.0058920 0.01762076 91  0.33438  0.7389
## PhaseEOS_2       0.0047839 0.01632987 91  0.29295  0.7702
## PRE_EOS_NA       0.0107532 0.00210580 91  5.10645  0.0000
## HRV_RMSSD        0.0000990 0.00038668 91  0.25612  0.7984
## HRV_lf           0.0000012 0.00000606 91  0.20174  0.8406
## alpha_asymmetry -0.0419930 0.03532664 91 -1.18871  0.2376
## ThBt1           -0.0118111 0.00308797 91 -3.82489  0.0002
## ThBt2            0.0067596 0.00265481 91  2.54617  0.0126
##  Correlation:
##                 (Intr) EOS_CB PEOS_1 PEOS_2 PRE_EO HRV_RM HRV_lf alph_s ThBt1
## EOS_ControlB    -0.214
## PhaseEOS_1       0.137 -0.115
## PhaseEOS_2       0.043  0.050  0.586
## PRE_EOS_NA      -0.742  0.142 -0.092 -0.048
## HRV_RMSSD       -0.101 -0.313 -0.266 -0.410 -0.026
## HRV_lf          -0.201  0.523 -0.241  0.054  0.182 -0.612
## alpha_asymmetry  0.062  0.127  0.130  0.175 -0.064 -0.199  0.049
## ThBt1           -0.137  0.011 -0.101 -0.139 -0.037  0.044 -0.095 -0.196
## ThBt2           -0.367  0.069 -0.021  0.098  0.242 -0.175  0.247  0.193 -0.499
##
## Standardized Within-Group Residuals:
##         Min          Q1         Med          Q3         Max
## -2.71396550 -0.51218216  0.02820183  0.54186165  2.16998370
##
## Number of Observations: 126
## Number of Groups: 26
par(mfrow=c(1, 3))

plot(mod3)
```

plot(mod3$fitted, mod3$residuals)

qqnorm(mod3$residuals)

qqline(mod3$residuals, col="red")

hist(mod3$residuals)

plot(mod3, resid(., type = "p") ~ fitted(.) | EOS_Control, abline = 0)

plot(mod3, Subject ~ resid(.))

plot(mod3, POST_EOS_NA ~ fitted(.) | Subject, abline = c(0,1))

```
lillie.test(mod3$residuals)
##
##  Lilliefors (Kolmogorov-Smirnov) normality test
##
## data:  mod3$residuals
## D = 0.11091, p-value = 5.924e-08
intervals(mod3)
## Approximate 95% confidence intervals
##
##  Fixed effects:
##                         lower          est.         upper
## (Intercept)      2.194301e+00  2.310692e+00  2.427082e+00
## EOS_ControlB    -3.301682e-02 -4.466330e-03  2.408416e-02
## PhaseEOS_1      -2.910943e-02  5.892039e-03  4.089351e-02
## PhaseEOS_2      -2.765339e-02  4.783892e-03  3.722118e-02
## PRE_EOS_NA       6.570251e-03  1.075317e-02  1.493608e-02
## HRV_RMSSD       -6.690588e-04  9.903804e-05  8.671348e-04
## HRV_lf          -1.080777e-05  1.221712e-06  1.325119e-05
## alpha_asymmetry -1.121650e-01 -4.199299e-02  2.817903e-02
## ThBt1           -1.794500e-02 -1.181113e-02 -5.677257e-03
## ThBt2            1.486129e-03  6.759575e-03  1.203302e-02
##
##  Random Effects:
##   Level: Subject
##                     lower      est.     upper
## sd((Intercept)) 0.1052092 0.1418792 0.1913303
##
##  Within-group standard error:
##      lower       est.      upper
## 0.05548652 0.06417864 0.07423242
((exp(coef(mod3)["PRE_EOS_NA"]) - 1) * 100)[1, 1]
## [1] 1.081119
((exp(coef(mod3)["ThBt1"]) - 1) * 100)[1, 1]
## [1] -1.174165
((exp(coef(mod3)["ThBt2"]) - 1) * 100)[1, 1]
## [1] 0.6782473
```

In this model, a significant relationship was found with both Theta/Beta ratios (p = 0.001, and p = 0.013, respectively) and post-stress negative affect (p < 0.001). Normality of residuals is violated. We will use a simpler model with only the significant predictors.

##### 5.2.3.8. Simple model Negative affect - post-stress

```
# post-stress with EEG

mod3.1 <- lme(log(POST_EOS_NA) ~ PRE_EOS_NA + ThBt1 + ThBt2, random = ~ 1 | Subject, data = data_no_outlier, na.action = na.omit)

summary(mod3.1)
## Linear mixed-effects model fit by REML
##   Data: data_no_outlier
##         AIC       BIC   logLik
##   -212.7698 -195.9457 112.3849
##
## Random effects:
##  Formula: ~1 | Subject
##         (Intercept)   Residual
## StdDev:   0.1421039 0.06298694
##
## Fixed effects:  log(POST_EOS_NA) ~ PRE_EOS_NA + ThBt1 + ThBt2
##                  Value  Std.Error DF  t-value p-value
## (Intercept)  2.3247299 0.05351174 97 43.44337  0.0000
## PRE_EOS_NA   0.0104266 0.00201819 97  5.16630  0.0000
## ThBt1       -0.0118374 0.00289180 97 -4.09344  0.0001
## ThBt2        0.0070157 0.00246413 97  2.84712  0.0054
##  Correlation:
##            (Intr) PRE_EO ThBt1
## PRE_EOS_NA -0.737
## ThBt1      -0.184 -0.035
## ThBt2      -0.381  0.235 -0.468
##
## Standardized Within-Group Residuals:
##         Min          Q1         Med          Q3         Max
## -2.79846908 -0.55617580  0.04535611  0.55145678  2.13920520
##
## Number of Observations: 126
## Number of Groups: 26
par(mfrow=c(1, 3))

plot(mod3.1)
```

plot(mod3.1$fitted, mod3.1$residuals)

qqnorm(mod3.1$residuals)

qqline(mod3.1$residuals, col="red")

hist(mod3.1$residuals)

plot(mod3.1, resid(., type = "p") ~ fitted(.) | EOS_Control, abline = 0)

plot(mod3.1, Subject ~ resid(.))

plot(mod3.1, ThBt2 ~ fitted(.) | Subject, abline = c(0,1))

```
lillie.test(mod3.1$residuals)
##
##  Lilliefors (Kolmogorov-Smirnov) normality test
##
## data:  mod3.1$residuals
## D = 0.11052, p-value = 6.787e-08
intervals <- intervals(mod3.1)

# Define the transformation function
transform_func <- function(x) {
  (exp(x) - 1) * 100
}

# Apply the transformation to confidence intervals
transformed_intervals <- transform_func(intervals$fixed)

# Print the transformed confidence intervals
print(transformed_intervals)
##                   lower        est.        upper
## (Intercept) 819.3750404 922.3918587 1036.9518062
## PRE_EOS_NA    0.6441676   1.0481109    1.4536755
## ThBt1        -1.7423266  -1.1767625   -0.6079431
## ThBt2         0.2127314   0.7040333    1.1977438
## attr(,"label")
## [1] "Fixed effects:"
```

The simple model on the data without EEG or negative affect outliers revealed a significant influence of the post-stress negative affect (1.05 %, CI 0.66% and 1.45 %), Theta/Beta FP1(-1.18 %, CI -1.74 % and -0.61 %), and FP2 (0.70 %, CI 0.21 % and -0.61 %). The effect sizes are marginal and the normality of residuals is violated (p < 0.001). Variability in intercepts among subjects was characterized by a SD of 0.142, whereas residual variability had a SD of 0.063. Correlation analysis revealed low to medium associations between predictor variables. Moreover, standardized within-group residuals exhibited acceptable distributional properties, but the Lilliefors (Kolmogorov-Smirnov) normality test on residuals yielded a p-value of 6.787 e-08, indicating significant evidence against the null hypothesis of normality. This suggests potential deviations from normality in the residuals, which should be considered when interpreting the results of the model.

##### 5.2.3.9. Model comparison

```
# Extract relevant information from each model
model_names <- c("Model 1", "Model 2", "Model 3", "Model 4", "Model 5", "Model 6", "Model 7")
model_info <- sapply(list(mod1, mod1.1, mod2, mod2.1, mod2.2, mod3, mod3.1), extract_model_info)

# Create a data frame to store the information
model_comparison <- data.frame(Model = model_names,
                                Fixed_Effects_added = model_info[4,],
                                Random_Effects = model_info[5,],
                                Model_fit_AIC = model_info[1,],
                                Model_fit_BIC = model_info[2,],
                                Model_fit_logLik = model_info[3,])


# Print the table
print(model_comparison)
##     Model
## 1 Model 1
## 2 Model 2
## 3 Model 3
## 4 Model 4
## 5 Model 5
## 6 Model 6
## 7 Model 7
##                                                                                                Fixed_Effects_added
## 1 (Intercept), EOS_ControlB, PhaseEOS_1, PhaseEOS_2, baseline_NA, HRV_RMSSD, HRV_lf, alpha_asymmetry, ThBt1, ThBt2
## 2                                                                            (Intercept), HRV_RMSSD, HRV_lf, ThBt2
## 3 (Intercept), EOS_ControlB, PhaseEOS_1, PhaseEOS_2, baseline_NA, HRV_RMSSD, HRV_lf, alpha_asymmetry, ThBt1, ThBt2
## 4                                                                (Intercept), baseline_NA, ThBt1, ThBt2, HRV_RMSSD
## 5                                                                           (Intercept), baseline_NA, ThBt1, ThBt2
## 6  (Intercept), EOS_ControlB, PhaseEOS_1, PhaseEOS_2, PRE_EOS_NA, HRV_RMSSD, HRV_lf, alpha_asymmetry, ThBt1, ThBt2
## 7                                                                            (Intercept), PRE_EOS_NA, ThBt1, ThBt2
##   Random_Effects     Model_fit_AIC     Model_fit_BIC Model_fit_logLik
## 1    (Intercept) -37.9958370199858 -3.77147385295039 30.9979185099929
## 2    (Intercept) -89.7143166991307 -72.2383873847144 50.8571583495653
## 3    (Intercept) -120.943121862847 -87.9000395695707 72.4715609314235
## 4    (Intercept) -175.476032284109 -155.905498464931 94.7380161420543
## 5    (Intercept) -191.996367174646 -175.172240906247 101.998183587323
## 6    (Intercept)  -141.15966026453 -108.116577971254  82.579830132265
## 7    (Intercept) -212.769814243165 -195.945687974766 112.384907121583
```

Model “mod3.1” (5.2.3.8.) has the lowest BIC.

#### 5.2.4. Sleepiness

##### 5.2.4.1. Boxplots Sleepiness

```
ggplot(fulldata4to6, aes(x=factor(EOS_Control), y = POST_EOS_KSS, fill = factor(EOS_Control)))+
  geom_boxplot()+
    facet_wrap(~Session)+
    theme(legend.position="none")+
  scale_fill_manual(
    values = c("steelblue", "slategrey")
  ) +
    xlab("EOS or Control") +
    ylab("Sleepiness (post-intervention)")
```

In group A, the variation is higher, suggesting a non-uniform effect.

##### 5.2.4.2. Full model Sleepiness - without EEG outliers

```
# EEG without outliers
quartiles <- quantile(fulldata4to6$alpha_asymmetry, probs = c(0.25, 0.75), na.rm = FALSE)

IQR_val <- IQR(fulldata4to6$alpha_asymmetry)

 # Calculate lower and upper bounds for outlier detection

Lower <- quartiles[1] - 1.5 * IQR_val

Upper <- quartiles[2] + 1.5 * IQR_val

# Subset data to remove outliers

data_no_outlier <- subset(fulldata4to6, alpha_asymmetry > Lower & alpha_asymmetry < Upper)

# Calculate quartiles and IQR for Theta/Beta FP1

quartiles <- quantile(fulldata4to6$ThBt1, probs = c(0.25, 0.75), na.rm = FALSE)

IQR_val <- IQR(fulldata4to6$ThBt1)

# Calculate lower and upper bounds for outlier detection

Lower <- quartiles[1] - 1.5 * IQR_val

Upper <- quartiles[2] + 1.5 * IQR_val

# Subset data to remove outliers

data_no_outlier <- subset(data_no_outlier, ThBt1 > Lower & ThBt1 < Upper)

# Calculate quartiles and IQR for Theta/Beta FP2

quartiles <- quantile(data_no_outlier$ThBt2, probs = c(0.25, 0.75), na.rm = FALSE)

IQR_val <- IQR(data_no_outlier$ThBt2)

# Calculate lower and upper bounds for outlier detection

Lower <- quartiles[1] - 1.5 * IQR_val

Upper <- quartiles[2] + 1.5 * IQR_val

# Subset data to remove outliers

data_no_outlier <- subset(data_no_outlier, ThBt2 > Lower & ThBt2 < Upper)

# Print dimensions of data_no_outlier

dim(data_no_outlier)
## [1] 140  40
# baseline

mod1 <- lme((POST_EOS_KSS) ~ EOS_Control + Phase + baseline_KSS + HRV_RMSSD  + HRV_lf + alpha_asymmetry + ThBt1 + ThBt2, random = ~ 1 | Subject, data = data_no_outlier, na.action = na.omit)

summary(mod1)
## Linear mixed-effects model fit by REML
##   Data: data_no_outlier
##        AIC      BIC    logLik
##   478.1572 512.3815 -227.0786
##
## Random effects:
##  Formula: ~1 | Subject
##         (Intercept)  Residual
## StdDev:    1.536089 0.8305775
##
## Fixed effects:  (POST_EOS_KSS) ~ EOS_Control + Phase + baseline_KSS + HRV_RMSSD +      HRV_lf + alpha_asymmetry + ThBt1 + ThBt2
##                      Value Std.Error  DF   t-value p-value
## (Intercept)      2.7528058 0.5610489 102  4.906535  0.0000
## EOS_ControlB     0.7725675 0.1756362 102  4.398681  0.0000
## PhaseEOS_1      -0.0399112 0.2170404 102 -0.183888  0.8545
## PhaseEOS_2       0.0962138 0.2001106 102  0.480803  0.6317
## baseline_KSS     0.5438577 0.0649272 102  8.376422  0.0000
## HRV_RMSSD       -0.0092465 0.0042830 102 -2.158882  0.0332
## HRV_lf           0.0001704 0.0000719 102  2.370759  0.0196
## alpha_asymmetry  0.2398697 0.4286218 102  0.559630  0.5770
## ThBt1            0.0150522 0.0370480 102  0.406289  0.6854
## ThBt2           -0.0390495 0.0307173 102 -1.271254  0.2065
##  Correlation:
##                 (Intr) EOS_CB PEOS_1 PEOS_2 bs_KSS HRV_RM HRV_lf alph_s ThBt1
## EOS_ControlB    -0.170
## PhaseEOS_1       0.058 -0.108
## PhaseEOS_2       0.004  0.053  0.586
## baseline_KSS    -0.602  0.021  0.040 -0.008
## HRV_RMSSD       -0.138 -0.329 -0.303 -0.426 -0.053
## HRV_lf          -0.075  0.551 -0.228  0.060 -0.024 -0.583
## alpha_asymmetry -0.027  0.129  0.134  0.167  0.131 -0.269  0.081
## ThBt1           -0.238 -0.019 -0.057 -0.079  0.040  0.064 -0.123 -0.194
## ThBt2           -0.298  0.069 -0.024  0.059  0.099 -0.114  0.181  0.193 -0.509
##
## Standardized Within-Group Residuals:
##         Min          Q1         Med          Q3         Max
## -1.94306495 -0.62690164  0.02193966  0.54089059  3.32341534
##
## Number of Observations: 138
## Number of Groups: 27
par(mfrow=c(1, 3))

plot(mod1)
```

plot(mod1$fitted, mod1$residuals)

qqnorm(mod1$residuals)

qqline(mod1$residuals, col="red")

hist(mod1$residuals)

plot(mod1, resid(., type = "p") ~ fitted(.) | EOS_Control, abline = 0)

plot(mod1, Subject ~ resid(.))

plot(mod1, POST_EOS_KSS ~ fitted(.) | Subject, abline = c(0,1))

```
lillie.test(mod1$residuals)
##
##  Lilliefors (Kolmogorov-Smirnov) normality test
##
## data:  mod1$residuals
## D = 0.049622, p-value = 0.09738
intervals(mod1)
## Approximate 95% confidence intervals
##
##  Fixed effects:
##                         lower          est.         upper
## (Intercept)      1.639968e+00  2.7528057787  3.8656435527
## EOS_ControlB     4.241939e-01  0.7725674761  1.1209410071
## PhaseEOS_1      -4.704099e-01 -0.0399112097  0.3905874467
## PhaseEOS_2      -3.007046e-01  0.0962138497  0.4931322646
## baseline_KSS     4.150749e-01  0.5438576722  0.6726404817
## HRV_RMSSD       -1.774186e-02 -0.0092465229 -0.0007511846
## HRV_lf           2.783941e-05  0.0001704278  0.0003130162
## alpha_asymmetry -6.102997e-01  0.2398696816  1.0900390749
## ThBt1           -5.843233e-02  0.0150521709  0.0885366696
## ThBt2           -9.997705e-02 -0.0390494675  0.0218781150
##
##  Random Effects:
##   Level: Subject
##                    lower     est.   upper
## sd((Intercept)) 1.140931 1.536089 2.06811
##
##  Within-group standard error:
##     lower      est.     upper
## 0.7239153 0.8305775 0.9529554
```

The full model revealed a significant influence of the intervention type (control increasing sleepiness by 0.77 points, p < 0.001), baseline sleepiness (increasing by 0.54 points, p < 0.001), rMSSD (decreasing by 0.009 points/ms, p =0.033), and LF (increasing by 0.0002 points/ms^2, p = 0.020). The assumption of the normality of residuals is not violated, but still borderline significant (p = 0.10). The variability in intercepts among subjects was characterized by a SD of 1.54, wherea residual variability had a SD of 0.83. Correlation analysis revealed low to medium associations between predictor variables suggesting potential multi-co-linearity.

We will perform a simpler model with those selected predictors.

##### 5.2.4.3. Simple model Sleepiness

```
# baseline

mod2 <- lme((POST_EOS_KSS) ~ EOS_Control + baseline_KSS + HRV_RMSSD + HRV_lf, random = ~ 1 | Subject, data = fulldata4to6, na.action = na.omit)

summary(mod2)
## Linear mixed-effects model fit by REML
##   Data: fulldata4to6
##        AIC      BIC    logLik
##   547.6747 569.0684 -266.8373
##
## Random effects:
##  Formula: ~1 | Subject
##         (Intercept)  Residual
## StdDev:    1.554978 0.8939276
##
## Fixed effects:  (POST_EOS_KSS) ~ EOS_Control + baseline_KSS + HRV_RMSSD + HRV_lf
##                   Value Std.Error  DF   t-value p-value
## (Intercept)   2.3139735 0.4671684 130  4.953189  0.0000
## EOS_ControlB  0.8421665 0.1650668 130  5.101975  0.0000
## baseline_KSS  0.5935541 0.0639162 130  9.286443  0.0000
## HRV_RMSSD    -0.0092064 0.0034449 130 -2.672514  0.0085
## HRV_lf        0.0001879 0.0000601 130  3.126146  0.0022
##  Correlation:
##              (Intr) EOS_CB bs_KSS HRV_RM
## EOS_ControlB -0.171
## baseline_KSS -0.631 -0.028
## HRV_RMSSD    -0.263 -0.236 -0.016
## HRV_lf       -0.033  0.492 -0.040 -0.628
##
## Standardized Within-Group Residuals:
##          Min           Q1          Med           Q3          Max
## -2.208910005 -0.635473708  0.006885959  0.561371334  2.196888330
##
## Number of Observations: 162
## Number of Groups: 28
par(mfrow=c(1, 3))

plot(mod2)
```

plot(mod2$fitted, mod2$residuals)

qqnorm(mod2$residuals)

qqline(mod2$residuals, col="red")

hist(mod2$residuals)

plot(mod2, resid(., type = "p") ~ fitted(.) | EOS_Control, abline = 0)

plot(mod2, Subject ~ resid(.))

plot(mod2, POST_EOS_KSS ~ fitted(.) | Subject, abline = c(0,1))

```
lillie.test(mod2$residuals)
##
##  Lilliefors (Kolmogorov-Smirnov) normality test
##
## data:  mod2$residuals
## D = 0.051497, p-value = 0.03784
intervals(mod2)
## Approximate 95% confidence intervals
##
##  Fixed effects:
##                      lower          est.         upper
## (Intercept)   1.389737e+00  2.3139735371  3.2382103865
## EOS_ControlB  5.156016e-01  0.8421665034  1.1687313843
## baseline_KSS  4.671035e-01  0.5935540514  0.7200045850
## HRV_RMSSD    -1.602167e-02 -0.0092064327 -0.0023911908
## HRV_lf        6.897118e-05  0.0001878554  0.0003067396
##
##  Random Effects:
##   Level: Subject
##                    lower     est.    upper
## sd((Intercept)) 1.169504 1.554978 2.067506
##
##  Within-group standard error:
##     lower      est.     upper
## 0.7915748 0.8939276 1.0095148
```

The model with heart physiology shows a significant association with intervention type (control increasing sleepiness by 0.84 points), baseline sleepiness (increasing by 0.60 points, p <0.001), rMSSD (decreasing by 0.009 points/ms, p = 009), and LF (increasing by 0.0002 points/ms^2, p = 0.002). However residuals are not normal, and AIC, BIC, and log likelihood are worse than they were in the full model. We will therefore use the full model to interpret coefficients.

##### 5.2.4.4. Model comparison

```
# Function to extract model information
extract_model_info <- function(model) {
  AIC <- AIC(model)
  BIC <- BIC(model)
  logLik <- logLik(model)
  fixed_effects <- paste(names(fixef(model)), collapse = ", ")
  random_effects <- paste(names(ranef(model)), collapse = ", ")
  return(c(AIC, BIC, logLik, fixed_effects, random_effects))
}

# Extract relevant information from each model
model_names <- c("Model 1", "Model 2")
model_info <- sapply(list(mod1, mod2), extract_model_info)

# Create a data frame to store the information
model_comparison <- data.frame(Model = model_names,
                                Fixed_Effects_added = model_info[4,],
                                Random_Effects = model_info[5,],
                                Model_fit_AIC = model_info[1,],
                                Model_fit_BIC = model_info[2,],
                                Model_fit_logLik = model_info[3,])


# Print the table
print(model_comparison)
##     Model
## 1 Model 1
## 2 Model 2
##                                                                                                 Fixed_Effects_added
## 1 (Intercept), EOS_ControlB, PhaseEOS_1, PhaseEOS_2, baseline_KSS, HRV_RMSSD, HRV_lf, alpha_asymmetry, ThBt1, ThBt2
## 2                                                        (Intercept), EOS_ControlB, baseline_KSS, HRV_RMSSD, HRV_lf
##   Random_Effects    Model_fit_AIC    Model_fit_BIC  Model_fit_logLik
## 1    (Intercept) 478.157171670957 512.381534837993 -227.078585835479
## 2    (Intercept) 547.674696900868 569.068417538306 -266.837348450434
```

Full model “mod1” (5.2.4.2.) as lower BIC, however there are too many insignificant predictors, thus we opted to include the simple model “mod2” (5.2.4.3.) for final reporting. #### 5.2.5. Summary

The following models were selected based on BIC: the model for post-relaxation subjective stress with post-stress subjective stress, intervention type, and Theta/Beta FP1 (without outliers), 5.2.1.8. The model for post-relaxation positive affect with post-stress positive affect and intervention type, 5.2.2.5. The model for post-relaxation negative affect with post-stress negative affect and Theta/Beta ratios in both frontal lobes (without outliers), 5.2.3.8. The model for post-relaxation subjective sleepiness with intervention type, baseline negative affect and all HRV variables (without outliers), 5.2.4.3.

The model for post-relaxation subjective stress involving baseline subjective stress did not reveal its significant influence. However, it indicated a significant influence of intervention type and Theta/Beta FP1. The model violated normality of residuals. The second model involving post-stress subjective stress instead of baseline revealed a significant influence of intervention type and of post-stress subjective stress and a borderline significance of Theta/Beta FP1. The model has also violated normality of residuals.

The models for post-relaxation positive affect involving baseline positive affect revealed their significant influence, including intervention type and Theta/Beta FP2. The normality of residuals in the model is violated, which should be considered when interpreting the results of the model. In a model which included post-stress positive affect along baseline positive affect, the influence of the latter became non-significant and weak. Therefore a model with only post-stress positive affect was used, including intervention type and Theta/Beta FP2 as significant predictors.

The models for post-relaxation negative affect including either baseline or post-stress negative affect revealed statistically significant but marginal effect sizes. Better model performance exhibited a model with post-stress negative affect.

The model for subjective sleepiness revealed a significant impact of the intervention type (control increased by 0.84 points), baseline sleepiness (increased by 0.60 points), rMSSD (decreasing effect), and LF (increasing effect). The assumption of residuals normality holds for the full, but not for the simplified, model. Correlation analysis in the full model might suggest the potential of multi-co-linearity in some variables.

## 6. Final models selection and reasoning

In the publication, we involved the simple model for log-transformed subjective stress including post-stress subjective stress (AIC = 72.361, BIC = 89.61, log likelihood = -30.18) instead of baseline subjective stress (AIC = 89.89, BIC = 104.35, log likelihood = -39.93 due to its better model performance. Both models have violated normality of residuals. We will use the model on the data without EEG outliers with intervention type, post-stress subjective stress, and Theta/Beta ratio described above.

For positive affect, the best performance was the model involving baseline, post-stress positive affect, and Theta/Beta FP2 (AIC = 806.30, BIC = 826.53, log likelihood = -396.15). The normality of residuals was violated. However, because some variables were not significant, we opted for simpler model involving only post-stress positive affect (AIC = 922.51, BIC = 937.86, log likelihood = -456.26).

The best performing model for negative affect involved post-stress negative affect, Theta/Beta FP1 and FP2 (AIC = -212.77, BIC = -195.95, log likelihood = -122.38). The normality of residuals was violated.

For the sleepiness, the best performing model was the full model (AIC = 478.16, BIC = 512.38, log likelihood -227.08) compared to the simpler model (AIC = 547.67, BIC = 569.07, log Likelihood -266.84). The normality of residuals was not violated in the full model (p = 0.097), but was below the threshold in the simpler model (p = 0.038). However, because majority of variables in full model were not significant, we opted for the simpler model (AIC = 547.68, BIC = 569.07, log likelihood = -266.84).

## 7. Package information

library(grateful)
pkgs <- cite_packages(output = "table", out.dir = ".")
knitr::kable(pkgs)

| Package | Version | Citation |
| --- | --- | --- |
| base | 4.2.3 | @base |
| car | 3.1.2 | @car |
| consort | 1.2.1 | @consort |
| contrast | 0.24.2 | @contrast |
| DiagrammeR | 1.0.11 | @DiagrammeR |
| ez | 4.4.0 | @ez |
| foreign | 0.8.84 | @foreign |
| ggpubr | 0.6.0 | @ggpubr |
| glm2 | 1.2.1 | @glm2 |
| gridExtra | 2.3 | @gridExtra |
| knitr | 1.46 | @knitr2014; @knitr2015; @knitr2024 |
| lme4 | 1.1.35.3 | @lme4 |
| lmtest | 0.9.40 | @lmtest |
| MASS | 7.3.58.2 | @MASS |
| nlme | 3.1.162 | @nlme2000; @nlme2023 |
| nortest | 1.0.4 | @nortest |
| openxlsx | 4.2.5.2 | @openxlsx |
| ordinal | 2023.12.4 | @ordinal |
| psych | 2.4.3 | @psych |
| rmarkdown | 2.26 | @rmarkdown2018; @rmarkdown2020; @rmarkdown2024 |
| rstatix | 0.7.2 | @rstatix |
| rticles | 0.27 | @rticles |
| tidyverse | 2.0.0 | @tidyverse |
