# Supplementary material for: The effects of coherent breathing and blue light after an acute stress on sleepiness and the stress response in a workplace setting: A randomized double-blind cross-over trial
Source: PLoS One. 2026 Sep 30;21(9):e0357293. doi: 10.1371/journal.pone.0357293 (PMC13626309; doi:10.1371/journal.pone.0357293)
Supplement: Supplementary file 2 [file pone.0357293.s003.docx]

**Response to Reviewers**

Manuscript ID: PONE-D-25-23417

Title: The effects of coherent breathing and blue light after an acute stress on sleepiness and the stress response in a workplace setting: a randomized double-blind cross-over trial

We thank the Academic Editor and both reviewers for their careful reading of our manuscript and their constructive comments. We have addressed all points raised and provide detailed responses below. Reviewer comments are shown in blue italics; our responses are in black. Manuscript changes are shown with added text in green bold and deleted text in orange strikethrough; unchanged surrounding text is shown in dark grey.

**Legend: added text** ~~deleted text~~ unchanged text

**Response to Academic Editor Requirements**

**Editor requirement 1 — Abbreviations**

*Kindly write the full length word of all abbreviations for the first mention in the text beginning from the abstract.*

**Response:**

All abbreviations have been spelled out at first mention throughout the manuscript, including in the abstract. Commonly used abbreviations such as SD, CI, and WHO have been retained without expansion as they are standard in the field. Abbreviations that appeared only once in the text (e.g., SCN, HPA) have been replaced by their full form without introducing the abbreviation. A detailed list of all changes is provided in the responses to Reviewer 2 below.

**Editor requirement 2 — Figure quality**

*Kindly enhance the quality of the figures.*

**Response:**

Figures have been exported from RStudio as EPS files with a width of 850 pixels, above the required minimum of 789 pixels. The manuscript now displays TIFF versions at 850 pixels width, in line with PLOS ONE figure guidelines.

**Editor requirement 3 — Funding Statement**

*Please provide an amended Funding Statement that declares all funding or sources of support received during this study.*

**Response:**

An updated Funding Statement is provided in the cover letter accompanying this submission, as requested.

**Editor requirement 4 — Competing Interests Statement**

*Please provide an amended Competing Interests Statement declaring commercial affiliations.*

**Response:**

An updated Competing Interests Statement is provided in the cover letter. We also note that all statistical analyses were performed independently by researchers at RECETOX, Masaryk University, who have no commercial affiliation with Human Design Group.

**Editor requirement 5 — Deposited protocol**

*If applicable, we recommend that you deposit your laboratory protocols.*

**Response:**

The study protocol has already been deposited on Protocols Exchange: https://dx.doi.org/10.21203/rs.3.pex-2297/v1. We would appreciate it if the editorial team could link it to the published article.

**Response to Reviewer 1**

**Reviewer 1 — Comment 1: Abstract length (lines 23–28)**

*Lines 23–28 is too long a sentence, please consider breaking up to improve readability.*

**Response:**

We thank the reviewer for this suggestion. The long sentence has been divided into two shorter, clearer sentences. The revised abstract now reads:

This study evaluates the effectiveness of an innovative tertiary stress management intervention for workplace application, combining 0.1 Hz coherent breathing with blue light exposure via the ÉOS device. **We hypothesize that the intervention can mitigate acute stress reactions and sleepiness more effectively than passive rest without external stimulation**~~The hypothesis was that the intervention will mitigate acute stress reactions and sleepiness more compared to passive rest without external stimulation~~. **Using** ~~Through~~ a randomized-controlled crossover double-blind design in a workplace-based laboratory setting, **we assessed the effect of an intervention following** ~~after~~ exposition to the Maastricht Acute Stress Test **in 29 individuals. S**~~on s~~ubjective stress (single item scale), sleepiness (Karolinska Sleepiness Scale), positive and negative affect (Positive and Negative Affect Schedule), along with heart rate variability (HRV) and electroencephalography metrics were **evaluated**~~assessed~~ using linear mixed-effect models ~~on 29 individuals~~. [...] This study contributes to stress management research by demonstrating **the potential of these tertiary stress management interventions** ~~its potential~~ to improve well-being and performance in occupational settings.

**Reviewer 1 — Comment 2: Description of the ÉOS system**

*In the methodology or introduction please include a brief line on what the EOS system is as the reader may not be familiar with such a device.*

**Response:**

We have addressed this at two levels. First, the description of the ÉOS device has been expanded in the Introduction where it is first mentioned, replacing the previous shorter formulation:

The intervention integrates coherent breathing guided by an external blue light stimulus, delivered by the ÉOS device **— a multisensory recovery system developed by Human Design Group designed for fatigue and stress management in occupational settings, offering calibrated sessions adaptable to the user’s state and recovery goals — in a Research & Development (R&D) laboratory within Human Design Group’s office.**~~in a Research & Development (R&D) laboratory within Human design Group’s offices.~~

Second, a dedicated subsection titled 'ÉOS system' has been added at the beginning of the Methods section, providing a full technical description:

**ÉOS system**

**The ÉOS device delivers multisensory recovery sessions through two main components: a LED lighting system emitting calibrated wavelengths of light according to the session scenario and recovery goal, and a spatialized sound environment providing auditory guidance for coherent breathing, meditation, or relaxation. Sessions are calibrated based on the user’s state (fatigue or stress), the selected duration, and relaxation preferences, with or without sleep. In the present study, all participants underwent both the experimental and control conditions as part of the crossover design. In the experimental condition, a single standardized 15-minute relaxation scenario without sleep was assigned by the researchers, during which coherent breathing was guided at 5.5 cycles per minute (0.1 Hz) through synchronized light pulses and auditory cues. Participants did not select or modify any session parameters. The specific parameters of the light and sound stimuli are proprietary.**

**Reviewer 1 — Comment 3: Quality control for R-peak detection (R-DECO)**

*Please include information as to whether there was any quality control in place associated with the manual adjustment of adding, removing or adjusting the R peaks on the R-DECO system.*

**Response:**

A description of the quality control procedure has been added directly after the existing description of the automated detection algorithm in the ECG preprocessing section:

However, erroneous or mis-positioned peaks can be present after processing, especially for recording with a high level of noise. For this reason, R-DECO includes a graphical user interface that can be used to correct for algorithm errors by manually adding, removing or adjusting the R-peaks. **Quality control of R-peak detection was ensured through systematic visual inspection of the entire ECG time series for each participant by a trained operator, who manually corrected any flagged or visually identified anomalies. A final review of the corrected series was performed to confirm the absence of residual errors prior to HRV feature extraction. This procedure was applied uniformly across all participants and sessions.**

**Reviewer 1 — Concern 1: Isolation of coherent breathing vs. blue light effects**

*I find a significant limitation of this study is there is no isolation of the effect of coherent breathing compared to the effect of the blue light.*

**Response:**

We fully acknowledge this limitation. The combined nature of the intervention does not allow us to isolate the independent contribution of coherent breathing versus blue light exposure. This is explicitly stated as a direction for future research in the Discussion section. To make this recommendation even more explicit, we have added the words “isolate and” in the relevant sentence:

Another potential next step may involve the comparison of different light conditions (darkness, natural, and artificial light), and in more stratified grouping (coherent breathing without light guidance, or light without breathing modulation) to **isolate and** delineate the specific effects of each intervention type.

**Reviewer 1 — Concern 2: Log-transformed vs. non-transformed data**

*I do have a concern with the way log-transformed data has been used and displayed alongside non-log transformed data. Perhaps this requires the inclusion of a table with log transformed results and non-log transformed results?*

**Response:**

We thank the reviewer for raising this methodological point and are happy to clarify. The figures present boxplots of non-transformed raw data in all instances, ensuring direct visual comparability across outcomes. Log-transformation was applied selectively to certain variables (specifically negative affect) solely to meet the homoskedasticity assumption required for valid Linear Mixed-Effects Modeling (LMEM). Running LMEM on heteroscedastic data violates the assumption of constant residual variance, leading to distorted standard errors, misleading p-values, and invalid hypothesis testing.

Presenting two parallel tables — one with transformed and one with non-transformed results — would be methodologically misleading, as coefficients are not directly comparable across scales: non-transformed coefficients represent absolute unit-based changes, while log-transformed coefficients represent relative changes. Comparing AIC/BIC across these models would also be invalid. To facilitate interpretation, we converted log-transformed coefficients to percentage changes throughout the Results section, allowing readers to interpret all outcomes on an intuitive scale. Readers are consistently reminded in the text which models used transformed data. No change to the manuscript text was required for this point.

**Response to Reviewer 2**

**Reviewer 2 — Comment 1: Abstract line 21**

*In the abstract, line 21 you could re-word this sentence to be a bit clearer and more fluent.*

**Response:**

The sentence has been reworded as shown above in the revised abstract (Reviewer 1, Comment 1).

**Reviewer 2 — Comment 2: Abstract line 23**

*In the abstract, line 23 could be cleaned up a bit for better understanding.*

**Response:**

The sentence has been restructured for clarity and directness, as shown above in the revised abstract (Reviewer 1, Comment 1).

**Reviewer 2 — Comment 3: Line 91 — Suprachiasmatic Nucleus**

*In line 91, be sure to spell out the entire name for the pathway as follows Suprachiasmatic Nucleus (SCN) Pathway.*

**Response:**

As this term appeared only once in the manuscript, we replaced the abbreviation with the full term and removed the acronym to avoid introducing an abbreviation used only once:

...through direct signalling in the intrinsically photosensitive retinal circuitry to the **suprachiasmatic nucleus** ~~SCN~~ pathway [36]...

**Reviewer 2 — Comment 4: Description of the ÉOS device (line 104)**

*Be sure to explain what an EOS device is in your introduction. See line 104.*

**Response:**

Please see Reviewer 1, Comment 2 above. A contextual description has been added in the Introduction and a dedicated subsection has been added in the Methods section.

**Reviewer 2 — Comment 5: Line 109 — Electroencephalography**

*Line 109, be sure to spell out Electroencephalography (EEG).*

**Response:**

Electroencephalography (EEG) has been spelled out at first mention in the Introduction:

...and **electroencephalography (**EEG**)** measurements (frontal alpha asymmetry...

**Reviewer 2 — Comment 6: Lines 125, 265 — ‘a priori’ spelling**

*Lines 125, 265 check spelling formats for 'a-priori' as it should be 'a priori'.*

**Response:**

'A-priori' has been corrected to 'a priori' (without hyphen) at all occurrences in the manuscript:

...based on an a~~-~~priori power analysis...

**Reviewer 2 — Comment 7: Line 133 — Electrocardiography**

*Line 133, be sure to spell out Electrocardiography (ECG).*

**Response:**

Electrocardiography (ECG) has been spelled out at first mention in the exclusion criteria section:

...detectable by **electrocardiography (**ECG**)**, cardiovascular diseases...

**Reviewer 2 — Comment 8: Line 136 — Hypothalamic-Pituitary-Adrenal axis**

*Line 136, be sure to spell out Hypothalamic-Pituitary-Adrenal (HPA) axis.*

**Response:**

As this term appeared only once, we replaced the abbreviation with the full term:

...medication known to affect the **hypothalamic-pituitary-adrenal** ~~HPA~~ axis...

**Reviewer 2 — Comment 9: KSS abbreviation (line 163)**

*In line 163 you introduce the term 'Karolinska Sleepiness Scale' but you did not include the abbreviation until 169.*

**Response:**

The abbreviation (KSS) has been introduced at first mention, and PANAS has been similarly added at its first occurrence in the same sentence:

...Karolinska Sleepiness Scale **(KSS)**, and Positive and Negative Affect Schedule **(PANAS)** were administered in both sessions...

**Reviewer 2 — Comment 10: PANAS abbreviation consistency (lines 163–170)**

*Be consistent with your abbreviations and terminology. Line 170 is missing the spelled-out term for PANAS.*

**Response:**

The Positive and Negative Affect Schedule (PANAS) abbreviation has been introduced at first mention, as shown above in Comment 9. All outcome measures with established abbreviations (KSS, PANAS, HRV) now follow the full name at first mention pattern throughout the manuscript.

**Reviewer 2 — Comment 11: Line 197 — WHR**

*In line 197, specify what WHR refers to: Waist-to-Hip Ratio.*

**Response:**

'WHR' has been replaced by the full term 'waist-hip ratio':

...weight, height, **waist-hip ratio**~~WHR~~, use of cardioactive medication...

**Reviewer 2 — Comment 12: Spelling check (line 334)**

*Check spelling throughout the manuscript, example line 334.*

**Response:**

We carefully reviewed the manuscript for spelling errors and ran a thorough spell-check across the full text. We corrected the following issues identified during this process: (1) "Supplmentary" corrected to "Supplementary" in the Data analysis section; (2) "a-priori" corrected to "a priori" (without hyphen) at all occurrences; (3) the title of Table 3 was incorrectly labelled "of positive affect" and has been corrected to "of log-transformed negative affect"; (4) "stress response neither" corrected to "stress response either" in the Discussion; (5) "Akakike" corrected to "Akaike" in the Data analysis section; (6) "drop-outs" corrected to "dropouts" throughout; (7) "is detailed" corrected to "are detailed" in the Interventions section; (8) "Special thanks goes" corrected to "Special thanks go" in the Acknowledgements. The specific issue originally flagged at line 334 was not identifiable in our version of the document as the text of this line was "Table 1. Linear mixed-effects model (LMEM) of log-transformed subjective stress"; if the reviewer can provide further detail, we will be happy to address it directly.

**Reviewer 2 — Comment 13: Future research on light conditions (line 464)**

*In line 464, you mention the potential for comparison against different light conditions. It would be important to expand and be more precise when mentioning this future study. Include a direct comparison for example between blue vs red and dim light affecting cortisol and stress responses. Also include a brief reference of how blue light affects endorphins. See Petrowski et al., 2021 and Albers et al., 2019.*

**Response:**

We thank the reviewer for these suggestions and have reconsidered our initial position. While we maintain that the references on blue light effects on cortisol (Petrowski et al., 2021) and endorphins (Albers et al., 2019) fall outside the direct scope of the present study — which focused on subjective and autonomic stress markers rather than hormonal or biochemical outcomes — we have enriched the future directions paragraph by incorporating two recent studies that have been published since our initial submission and that directly support our research line.

Canazei et al. (2025, Scientific Reports) conducted a controlled crossover study in a simulated office environment showing that light-guided resonant breathing significantly increased RMSSD following acute stress induction, with large effect sizes. This closely mirrors our design and findings. DeGraves et al. (2024, Journal of the American Geriatrics Society) demonstrated that coherent breathing is feasible and effective as a stress management strategy in a long-term care workforce context over an 8-week period, extending the applicability of this approach to occupational settings. Both references strengthen the future research directions we recommend and have been added to the Discussion:

Assessing long-term effects of these in-office interventions and including cognitive tests post-intervention could further elucidate the practical impact of these stress countermeasures. **Notably, two recent studies further support these future directions. Canazei et al. (2025) demonstrated that light-guided resonant breathing significantly enhanced autonomic recovery (RMSSD) following acute stress induction in a simulated office environment, corroborating our findings and underlining the potential of light-guided breathing as an occupational stress recovery strategy [100]. DeGraves et al. (2024) showed that coherent breathing is a feasible and effective stress management strategy for long-term care workers over an 8-week period, suggesting applicability beyond acute stress contexts [101].**

**Reviewer 2 — Comment 14: Acute vs. chronic stress specification**

*Throughout the manuscript you compare managing techniques for acute vs chronic stress, be sure to specify in your methodology what type of stress you are addressing with your technique.*

**Response:**

We thank the reviewer for this important clarification request. The ÉOS device is a tertiary stress management intervention designed to be applicable to both acute and chronic stress contexts. However, the present study specifically tested its effectiveness in the context of acute stress, induced using the Maastricht Acute Stress Test (MAST). This distinction is now made explicit in the newly added 'ÉOS system' subsection of the Methods, which specifies that a single standardized acute stress recovery scenario was used, and that participants did not select session parameters. The relevant passage reads:

**In the present study, all participants underwent both the experimental and control conditions as part of the crossover design. In the experimental condition, a single standardized 15-minute relaxation scenario without sleep was assigned by the researchers, during which coherent breathing was guided at 5.5 cycles per minute (0.1 Hz) through synchronized light pulses and auditory cues. Participants did not select or modify any session parameters.**

This confirms that the intervention was administered in a controlled, standardized manner in response to an acute stress induction (MAST), addressing the reviewer’s concern about the type of stress being targeted.

**Summary of all changes**

All modifications are visible in the tracked-changes version submitted alongside this response. Main additions and revisions:

1. Abstract: sentences restructured and reworded (Reviewer 1 Comment 1, Reviewer 2 Comments 1–2).

2. Introduction: brief description of the ÉOS device expanded at first mention (Reviewer 1 Comment 2, Reviewer 2 Comment 4).

3. Methods > Interventions: KSS and PANAS abbreviations added at first mention (Reviewer 2 Comments 9–10).

4. Methods: new subsection 'ÉOS system' added before Anthropometry, also addressing the acute stress context (Reviewer 1 Comment 2, Reviewer 2 Comments 4 and 14).

5. Methods > ECG preprocessing: quality control procedure for R-peak detection added (Reviewer 1 Comment 3).

6. Abbreviations: all spelled out at first mention throughout — SCN, EEG, ECG, HPA, KSS, PANAS, WHR, a priori (Reviewer 2 Comments 3, 5–11).

7. Discussion: 'isolate and' added to future directions sentence; two new references added (Canazei 2025, DeGraves 2024) (Reviewer 1 Concern 1, Reviewer 2 Comment 13).

8. Figures: resolution improved to 850 pixels width in EPS and TIFF formats (Editor requirement 2).

9. Funding Statement and Competing Interests Statement: updated versions provided in the cover letter (Editor requirements 3–4).

We hope that the revised manuscript fully addresses the concerns raised and meets the standards of PLOS ONE. We remain available for any further clarification.

Yours sincerely,

Emma Chabani, Ph.D., on behalf of all co-authors
