# Supplementary material for: The effects of coherent breathing and blue light after an acute stress on sleepiness and the stress response in a workplace setting: A randomized double-blind cross-over trial
Source: PLoS One. 2026 Sep 30;21(9):e0357293. doi: 10.1371/journal.pone.0357293 (PMC13626309; doi:10.1371/journal.pone.0357293)
Supplement: Peer review history

# Peer review: Decision Letter 0

**DOI:** 10.1371/journal.pone.0357293.r001

**Authors:** Shimaa Yousof

1 May 2026

Dear Dr. Chabani,

Please submit your revised manuscript by Jun 15 2026 11:59PM. If you will need more time than this to complete your revisions, please reply to this message or contact the journal office at plosone@plos.org. When you're ready to submit your revision, log on to https://www.editorialmanager.com/pone/ and select the 'Submissions Needing Revision' folder to locate your manuscript file.

- A letter that responds to each point raised by the academic editor and reviewer(s). You should upload this letter as a separate file labeled 'Response to Reviewers'.
- A marked-up copy of your manuscript that highlights changes made to the original version. You should upload this as a separate file labeled 'Revised Manuscript with Track Changes'.
- An unmarked version of your revised paper without tracked changes. You should upload this as a separate file labeled 'Manuscript'.

If you would like to make changes to your financial disclosure, please include your updated statement in your cover letter. Guidelines for resubmitting your figure files are available below the reviewer comments at the end of this letter.

If applicable, we recommend that you deposit your laboratory protocols in protocols.io to enhance the reproducibility of your results. Protocols.io assigns your protocol its own identifier (DOI) so that it can be cited independently in the future. For instructions see: https://journals.plos.org/plosone/s/submission-guidelines#loc-laboratory-protocols. Additionally, PLOS ONE offers an option for publishing peer-reviewed Lab Protocol articles, which describe protocols hosted on protocols.io. Read more information on sharing protocols at https://plos.org/protocols?utm_medium=editorial-email&utm_source=authorletters&utm_campaign=protocols.

As the corresponding author, your ORCID iD is verified in the submission system and will appear in the published article. PLOS supports the use of ORCID, and we encourage all coauthors to register for an ORCID iD and use it as well. Please encourage your coauthors to verify their ORCID iD within the submission system before final acceptance, as unverified ORCID iDs will not appear in the published article. *Only*  the individual author can complete the verification step; PLOS staff *cannot*  verify ORCID iDs on behalf of authors.

We look forward to receiving your revised manuscript.

Kind regards,

Shimaa Mohammad Yousof, Msc, M.D., Ph.D

Academic Editor

PLOS One

Journal Requirements:

When submitting your revision, we need you to address these additional requirements.

1. Please ensure that your manuscript meets PLOS ONE's style requirements, including those for file naming. The PLOS ONE style templates can be found at

https://journals.plos.org/plosone/s/file?id=wjVg/PLOSOne_formatting_sample_main_body.pdf and

https://journals.plos.org/plosone/s/file?id=ba62/PLOSOne_formatting_sample_title_authors_affiliations.pdf.

2. Thank you for stating in your Funding Statement:

“This research was conducted with institutional support from Spaceship FR at CNES and Human Design Group, who provided salaries for L.B., E.C., M.D, S.B, F.N. and L.R. for an 6 month intership in the context of their professional roles. The Research Infrastructure RECETOX (No. LM2023069) was funded by the Ministry of Education, Youth and Sports of the Czech Republic, and the European Union’s Horizon 2020 programme under grant agreement No. 857560 (CETOCOEN Excellence www.recetox.muni.cz/cetocoen). “

Please provide an amended statement that declares *all* the funding or sources of support (whether external or internal to your organization) received during this study, as detailed online in our guide for authors at http://journals.plos.org/plosone/s/submit-now.  Please also include the statement “There was no additional external funding received for this study.” in your updated Funding Statement.

Please include your amended Funding Statement within your cover letter. We will change the online submission form on your behalf.

3. Thank you for stating the following in the Competing Interests section:

“The authors declare the following interests: Emma Chabani, Federico Nemmi, Stanislas Boyer, and Madison Diamond are employees of Human Design Group, which funded this research. Lucie Ráčková was also employed by Human Design Group during the six-month period of this study, in the context of a research internship; she is no longer affiliated with the company. Human Design Group is involved in the development of the product whose effectiveness in stress management was tested in this study. These affiliations do not alter the authors’ adherence to PLOS ONE policies on sharing data and materials.”

We note that one or more of the authors are employed by a commercial company

a.        Please provide an amended Funding Statement declaring this commercial affiliation, as well as a statement regarding the Role of Funders in your study. If the funding organization did not play a role in the study design, data collection and analysis, decision to publish, or preparation of the manuscript and only provided financial support in the form of authors' salaries and/or research materials, please review your statements relating to the author contributions, and ensure you have specifically and accurately indicated the role(s) that these authors had in your study. You can update author roles in the Author Contributions section of the online submission form.

Please also include the following statement within your amended Funding Statement.

“The funder provided support in the form of salaries for authors [insert relevant initials], but did not have any additional role in the study design, data collection and analysis, decision to publish, or preparation of the manuscript. The specific roles of these authors are articulated in the ‘author contributions’ section.”

If your commercial affiliation did play a role in your study, please state and explain this role within your updated Funding Statement.

b. Please also provide an updated Competing Interests Statement declaring this commercial affiliation along with any other relevant declarations relating to employment, consultancy, patents, products in development, or marketed products, etc.

Within your Competing Interests Statement, please confirm that this commercial affiliation does not alter your adherence to all PLOS ONE policies on sharing data and materials by including the following statement: "This does not alter our adherence to  PLOS ONE policies on sharing data and materials.” (as detailed online in our guide for authors http://journals.plos.org/plosone/s/competing-interests) . If this adherence statement is not accurate and  there are restrictions on sharing of data and/or materials, please state these. Please note that we cannot proceed with consideration of your article until this information has been declared.

Please include both an updated Funding Statement and Competing Interests Statement in your cover letter. We will change the online submission form on your behalf.

4. If the reviewer comments include a recommendation to cite specific previously published works, please review and evaluate these publications to determine whether they are relevant and should be cited. There is no requirement to cite these works unless the editor has indicated otherwise.

5. Please review your reference list to ensure that it is complete and correct. If you have cited papers that have been retracted, please include the rationale for doing so in the manuscript text, or remove these references and replace them with relevant current references. Any changes to the reference list should be mentioned in the rebuttal letter that accompanies your revised manuscript. If you need to cite a retracted article, indicate the article’s retracted status in the References list and also include a citation and full reference for the retraction notice.

[Note: HTML markup is below. Please do not edit.]

Reviewers' comments:

Reviewer's Responses to Questions

**Comments to the Author**

1. Is the manuscript technically sound, and do the data support the conclusions?

Reviewer #1: Yes

Reviewer #2: Yes

**********

2. Has the statistical analysis been performed appropriately and rigorously?

Reviewer #1: I Don't Know

Reviewer #2: Yes

**********

3. Have the authors made all data underlying the findings in their manuscript fully available?

The PLOS Data policy

Reviewer #1: Yes

Reviewer #2: Yes

**********

4. Is the manuscript presented in an intelligible fashion and written in standard English?

Reviewer #1: Yes

Reviewer #2: Yes

**********

Reviewer #1: Many thanks for the opportunity to review this article. The article poses an interesting question does coherent breathing with blue light exposure significantly reduce stress response compared to passive rest alone?

Minor recommendations:

Abstract:

Lines 23 -28 is too long a sentence please consider breaking up to improve readability.

In the methodology or introduction please include a brief line on what the EOS system is as the reader may not be familiar with such a device. Please include information as to whether there was any quality control in place associated with the manual adjustment of adding, removing or adjusting the R peaks on the R-DECO system.

Concerns to maybe consider:

I find a significant limitation of this study is there is no isolation of the effect of coherent breathing compared to the effect of the blue light something the author alludes to in their discussion giving grounds for further research.

I do have a concern; with the way log-transformed data has been used and displayed alongside non-log transformed data for positive affect which is without transformation and negative affect which is log transformed. My thoughts are ideally comparison of positive effect and negative effect should be either log transformed or not as I have concerns that maybe potential for bias in deciding which analysis methodology to display. Perhaps this requires the inclusion of a table with log transformed results and non-log transformed results? Please ignore this concern if this is just related to my unfamiliarity of reporting of transformed data.

Reviewer #2: Comments:

1. In the abstract, line 21 you could re-word this sentence to be a bit clearer and more fluent. For example, instead of saying “The hypothesis was that the intervention will mitigate acute stress reactions and sleepiness more compared to passive rest without external stimulation.” You could rephrase it to say, “We hypothesize that a tertiary intervention can mitigate acute stress reactions and sleepiness more effectively than passive rest without external stimulation.”

2. In the abstract, line 23 could be cleaned up a bit for better understanding. You currently state “Through a randomized-controlled crossover double-blind design in a workplace-based laboratory setting, the effect of intervention after exposition to Maastricht Acute Stress Test on subjective stress (single item scale), sleepiness (Karolinska Sleepiness Scale), positive and negative affect (Positive and Negative Affect Schedule), along with heart rate variability (HRV) and electroencephalography metrics were assessed using linear mixed-effect models on 29 individuals.” Try rewording this to be more direct, for example: “Using a randomized-controlled crossover double-blind design in a workplace-based laboratory setting, we measured the effects of intervention following the exposure to Maastricht Acute Stress Test on subjective stress (single item scale), sleepiness (Karolinska Sleepiness Scale), positive and negative affect (Positive and Negative Affect Schedule), heart rate variability (HRV) and electroencephalography metrics to asses linear mixed-effect models on 29 individuals.”

3. In line 91, be sure to spell out the entire name for the pathway as follows Suprachiasmatic Nucleus (SCN) Pathway.

4. Be sure to explain what an EOS device is in your introduction. See line 104.

5. Line 109, be sure to spell out Electroencephalography (EEG).

6. Line 125, 265 check spelling formats for “a-priori” as it should be “a priori”.

7. Line 133, be sure to spell out Electrocardiography (ECG).

8. Line 136, be sure to spell out Hypothalamic-Pituitary-Adrenal (HPA) axis.

9. Be sure to include appropriate abbreviations early on in your introduction. In line 163 you introduce the term “Karolinska Sleepiness Scale” but you did not include the abbreviation until 169 and later 173.

10. Be consistent with your abbreviations and terminology. You include abbreviations for some terms and not others, for example line 170 is missing the spelled-out term for PANAS but in line 167 you include the appropriate abbreviation. Revise lines 163-164, 169-170.

11. In line 197, specify what WHR refers to Waist-to-Hip Ratio.

12. Check spelling throughout the manuscript, example line 334.

13. In line 464, you mention the potential for comparison against different light conditions. It would be important to expand and be more precise when mentioning this future study. Include a direct comparison for example between blue vs red and dim light study affecting cortisol & stress responses (Blue light increases stress hormones vs other wavelengths, Petrowski et al., 2021 (Stress journal). Also take a look at Bright light vs dim light and its effects on stress, sleepiness, performance. It would also be important to include a brief reference of how blue light affects endorphins based on direct biochemical evidence. See Albers et al., 2019, Free Radical Biology & Medicine.

14. In your abstract you write “This study contributes to stress management research by demonstrating its potential to improve well-being and performance in occupational settings.” (line 34). Throughout the manuscript you compare managing techniques for acute vs chronic stress, be sure to specify in your methodology what type of stress you are addressing with your technique. This clarification will strengthen the validity of you methods.

**********

what does this mean?). If published, this will include your full peer review and any attached files.

If you choose “no”, your identity will remain anonymous but your review may still be made public.

**Do you want your identity to be public for this peer review?**  For information about this choice, including consent withdrawal, please see our Privacy Policy

Reviewer #1: No

Reviewer #2: No

**********

[NOTE: If reviewer comments were submitted as an attachment file, they will be attached to this email and accessible via the submission site. Please log into your account, locate the manuscript record, and check for the action link "View Attachments". If this link does not appear, there are no attachment files.]

To ensure your figures meet our technical requirements, please review our figure guidelines: https://journals.plos.org/plosone/s/figures

You may also use PLOS’s free figure tool, NAAS, to help you prepare publication quality figures: https://journals.plos.org/plosone/s/figures#loc-tools-for-figure-preparation.

NAAS will assess whether your figures meet our technical requirements by comparing each figure against our figure specifications.

# Author comment: Author response to Decision Letter 1

**DOI:** 10.1371/journal.pone.0357293.r002

16 Jun 2026

Please refer to the Response to Reviewers file uploaded with this submission for our detailed point-by-point responses.

Attachment Submitted filename: Response to Reviewers.docx

# Peer review: Decision Letter 1

**DOI:** 10.1371/journal.pone.0357293.r003

**Authors:** Shimaa Yousof

17 Aug 2026

The effects of coherent breathing and blue light after an acute stress on sleepiness and the stress response in a workplace setting: a randomized double-blind cross-over trial

PONE-D-25-23417R1

Dear Dr. Chabani,

We’re pleased to inform you that your manuscript has been judged scientifically suitable for publication and will be formally accepted for publication once it meets all outstanding technical requirements.

Within one week, you’ll receive an e-mail detailing the required amendments. When these have been addressed, you’ll receive a formal acceptance letter and your manuscript will be scheduled for publication.

An invoice will be generated when your article is formally accepted. Please note, if your institution has a publishing partnership with PLOS and your article meets the relevant criteria, all or part of your publication costs will be covered. Please make sure your user information is up-to-date by logging into Editorial Manager at Editorial Manager® and clicking the ‘Update My Information' link at the top of the page. For questions related to billing, please contact billing support.

If your institution or institutions have a press office, please notify them about your upcoming paper to help maximize its impact. If they’ll be preparing press materials, please inform our press team as soon as possible -- no later than 48 hours after receiving the formal acceptance. Your manuscript will remain under strict press embargo until 2 pm Eastern Time on the date of publication. For more information, please contact onepress@plos.org.

Kind regards,

Prof. Shimaa Mohammad Yousof, Msc, M.D., Ph.D

Academic Editor

PLOS One

Additional Editor Comments (optional):

Dear Author

Kindyl, Edit parenthesis and word flow to ensure clarity. In line 114-118: Edit the parenthesis to reflect the following: “In this randomized-controlled cross-over double blinded design, parameters are measured through both subjective and objective markers. These markers include questionnaires and measurements of heart rate variability using root mean square successive difference (rMSSD), low-frequency band (LF), and electroencephalography (EEG) of frontal alpha asymmetry (FAA) and theta/beta ratio (S 1.4) [41]. Rephrase this sentence for clarity. Also, when citing supplemental information only use (S#).

Reviewers' comments:

Reviewer's Responses to Questions

**Comments to the Author**

Reviewer #1: All comments have been addressed

Reviewer #2: All comments have been addressed

**********

2. Is the manuscript technically sound, and do the data support the conclusions?

Reviewer #1: Yes

Reviewer #2: Yes

**********

3. Has the statistical analysis been performed appropriately and rigorously?

Reviewer #1: I Don't Know

Reviewer #2: Yes

**********

4. Have the authors made all data underlying the findings in their manuscript fully available?

The PLOS Data policy

Reviewer #1: Yes

Reviewer #2: Yes

**********

5. Is the manuscript presented in an intelligible fashion and written in standard English?

Reviewer #1: Yes

Reviewer #2: Yes

**********

Reviewer #1: The authors have a detailed response to all questions raised on previous submission. The submission reads well for a technical study.

Reviewer #2: Edits Suggested:

1. Edit parenthesis and word flow to ensure clarity. In line 114-118: Edit the parenthesis to reflect the following: “In this randomized-controlled cross-over double blinded design, parameters are measured through both subjective and objective markers. These markers include questionnaires and measurements of heart rate variability using root mean square successive difference (rMSSD), low-frequency band (LF), and electroencephalography (EEG) of frontal alpha asymmetry (FAA) and theta/beta ratio (S 1.4) [41]. Rephrase this sentence for clarity. Also, when citing supplemental information only use (S#).

**********

what does this mean?). If published, this will include your full peer review and any attached files.

If you choose “no”, your identity will remain anonymous but your review may still be made public.

**Do you want your identity to be public for this peer review?**  For information about this choice, including consent withdrawal, please see our Privacy Policy

Reviewer #1: No

Reviewer #2: No

**********

# Editor report: Acceptance letter

**DOI:** 10.1371/journal.pone.0357293.r004

**Authors:** Shimaa Yousof

PONE-D-25-23417R1

PLOS One

Dear Dr. Chabani,

I'm pleased to inform you that your manuscript has been deemed suitable for publication in PLOS One. Congratulations! Your manuscript is now being handed over to our production team.

At this stage, our production department will prepare your paper for publication. This includes ensuring the following:

* All references, tables, and figures are properly cited

* All relevant supporting information is included in the manuscript submission,

* There are no issues that prevent the paper from being properly typeset

You will receive further instructions from the production team, including instructions on how to review your proof when it is ready. Please keep in mind that we are working through a large volume of accepted articles, so please give us a few days to review your paper and let you know the next and final steps.

Lastly, if your institution or institutions have a press office, please let them know about your upcoming paper now to help maximize its impact. If they'll be preparing press materials, please inform our press team within the next 48 hours. Your manuscript will remain under strict press embargo until 2 pm Eastern Time on the date of publication. For more information, please contact onepress@plos.org.

You will receive an invoice from PLOS for your publication fee after your manuscript has reached the completed accept phase. If you receive an email requesting payment before acceptance or for any other service, this may be a phishing scheme. Learn how to identify phishing emails and protect your accounts at https://explore.plos.org/phishing.

If we can help with anything else, please email us at customercare@plos.org.

Thank you for submitting your work to PLOS One and supporting open access.

Kind regards,

PLOS One Editorial Office Staff

on behalf of

Professor Shimaa Mohammad Yousof

Academic Editor

PLOS One
